# Severity of SARS-CoV-2 infection in children with inborn errors of immunity (primary immunodeficiencies): a systematic review

**DOI:** 10.1186/s13223-023-00831-1

**Published:** 2023-08-09

**Authors:** Saad Alhumaid, Koblan M. Al Mutared, Zainab Al Alawi, Zainah Sabr, Ola Alkhars, Muneera Alabdulqader, Nourah Al Dossary, Fatemah M. ALShakhs, Rabab Abbas Majzoub, Yousef Hassan Alalawi, Khalid Al Noaim, Abdulrahman A. Alnaim, Mohammed A. Al Ghamdi, Abdulaziz A. Alahmari, Sawsan Sami Albattat, Yasin S. Almubarak, Essam Mohammed Al Abdulmohsen, Hanan Al Shaikh, Mortadah Essa Alobaidan, Hadi Hassan Almusallam, Fatimah Mohammed Alhassan, Mohammed Abdulhadi Alamer, Jawad Ali Al-Hajji, Duaa Ali Al-Hajji, Anwar Ahmed Alkadi, Abbas Al Mutair, Ali A. Rabaan

**Affiliations:** 1 School of Pharmacy, University of Tasmania, Hobart, 7000 Australia; 2 Administration of Pharmaceutical Care, Ministry of Health, 66255 Najran, Saudi Arabia; 3 Division of Allergy and Immunology, College of Medicine, King Faisal University, 31982 Hofuf, Al-Ahsa Saudi Arabia; 4 Division of Allergy and Immunology, Pediatric Department, College of Medicine, King Khalid University, 62529 Abha, Saudi Arabia; 5 Pediatric Department, King Faisal General Hospital, Ministry of Health, 36361 Hofuf, Al-Ahsa Saudi Arabia; 6 Pediatric Nephrology Specialty, Pediatric Department, Medical College, King Faisal University, 31982 Hofuf, Al-Ahsa Saudi Arabia; 7 General Surgery Department, Alomran General Hospital, Ministry of Health, 36358 Hofuf, Al-Ahsa Saudi Arabia; 8 Respiratory Therapy Department, Prince Saud Bin Jalawi Hospital, Ministry of Health, 36424 Al Mubarraz, Al-Ahsa Saudi Arabia; 9 Department of Pediatrics, College of Medicine, King Faisal University, 31982 Hofuf, Al-Ahsa Saudi Arabia; 10 Ear, Nose and Throat Department, Al Jabr Hospital for Eye, Ear, Nose and Throat, Ministry of Health, 36422 Al Mubarraz, Al-Ahsa Saudi Arabia; 11 Department of Pediatrics, King Fahad Hospital of the University, College of Medicine, Imam Abdulrahman Bin Faisal University, 34212 Dammam, Saudi Arabia; 12 College of Medicine, King Faisal University, 31982 Hofuf, Al-Ahsa Saudi Arabia; 13 Regional Medical Supply, Al-Ahsa Health Cluster, Ministry of Health, 36361 Hofuf, Al-Ahsa Saudi Arabia; 14 Pharmacy Department, Aljafr General Hospital, Ministry of Health, 7110 Hofuf, Al-Ahsa Saudi Arabia; 15 Infection Prevention and Control Department, Prince Saud Bin Jalawi Hospital, Ministry of Health, 36424 Al Mubarraz, Al-Ahsa Saudi Arabia; 16 Pharmacy Department, King Faisal General Hospital, Ministry of Health, 36361 Hofuf, Al-Ahsa Saudi Arabia; 17 Pharmacy Department, Prince Saud Bin Jalawi Hospital, Ministry of Health, 36424 Al Mubarraz, Al-Ahsa Saudi Arabia; 18 Primary Care Medicine, Al-Ahsa Health Cluster, Ministry of Health, 24231 Hofuf, Al-Ahsa Saudi Arabia; 19 Nursing Department, King Faisal General Hospital, Ministry of Health, 36361 Hofuf, Al-Ahsa Saudi Arabia; 20 Nursing Department, Prince Saud Bin Jalawi Hospital, Ministry of Health, 36424 Al Mubarraz, Al-Ahsa Saudi Arabia; 21 Research Center, Almoosa Specialist Hospital, 36342 Al Mubarraz, Al-Ahsa Saudi Arabia; 22 College of Nursing, Princess Norah Bint Abdul Rahman University, 11564 Riyadh, Saudi Arabia; 23 School of Nursing, University of Wollongong, Wollongong, NSW 2522 Australia; 24 Nursing Department, Prince Sultan Military College of Health Sciences, 33048 Dhahran, Saudi Arabia; 25 Molecular Diagnostic Laboratory, Johns Hopkins Aramco Healthcare, 31311 Dhahran, Saudi Arabia; 26 College of Medicine, Alfaisal University, 11533 Riyadh, Saudi Arabia; 27 Department of Public Health/Nutrition, The University of Haripur, Haripur, 22620 Khyber Pakhtunkhwa Pakistan

**Keywords:** Children, COVID-19, Errors, Immunodeficiency, Immunity, Inborn, Pediatric, Primary, SARS-CoV-2, Systematic review

## Abstract

**Background:**

Inborn errors of immunity (IEIs) are considered significant challenges for children with IEIs, their families, and their medical providers. Infections are the most common complication of IEIs and children can acquire coronavirus disease 2019 (COVID-19) even when protective measures are taken.

**Objectives:**

To estimate the incidence of severe acute respiratory syndrome coronavirus 2 (SARS-CoV-2) infection in children with IEIs and analyse the demographic parameters, clinical characteristics and treatment outcomes in children with IEIs with COVID-19 illness.

**Methods:**

For this systematic review, we searched ProQuest, Medline, Embase, PubMed, CINAHL, Wiley online library, Scopus and Nature through the Preferred Reporting Items for Systematic Reviews and Meta Analyses (PRISMA) guideline for studies on the development of COVID-19 in children with IEIs, published from December 1, 2019 to February 28, 2023, with English language restriction.

**Results:**

Of the 1095 papers that were identified, 116 articles were included in the systematic review (73 case report, 38 cohort 4 case-series and 1 case–control studies). Studies involving 710 children with IEIs with confirmed COVID-19 were analyzed. Among all 710 IEIs pediatric cases who acquired SARS-CoV-2, some children were documented to be admitted to the intensive care unit (ICU) (n = 119, 16.8%), intubated and placed on mechanical ventilation (n = 87, 12.2%), suffered acute respiratory distress syndrome (n = 98, 13.8%) or died (n = 60, 8.4%). Overall, COVID-19 in children with different IEIs patents resulted in no or low severity of disease in more than 76% of all included cases (COVID-19 severity: asymptomatic = 105, mild = 351, or moderate = 88). The majority of children with IEIs received treatment for COVID-19 (n = 579, 81.5%). Multisystem inflammatory syndrome in children (MIS-C) due to COVID-19 in children with IEIs occurred in 103 (14.5%). Fatality in children with IEIs with COVID-19 was reported in any of the included IEIs categories for cellular and humoral immunodeficiencies (n = 19, 18.6%), immune dysregulatory diseases (n = 17, 17.9%), innate immunodeficiencies (n = 5, 10%), bone marrow failure (n = 1, 14.3%), complement deficiencies (n = 1, 9.1%), combined immunodeficiencies with associated or syndromic features (n = 7, 5.5%), phagocytic diseases (n = 3, 5.5%), autoinflammatory diseases (n = 2, 3%) and predominantly antibody deficiencies (n = 5, 2.5%). Mortality was COVID-19-related in a considerable number of children with IEIs (29/60, 48.3%). The highest ICU admission and fatality rates were observed in cases belonging to cellular and humoral immunodeficiencies (26.5% and 18.6%) and immune dysregulatory diseases (35.8% and 17.9%) groups, especially in children infected with SARS-CoV-2 who suffered severe combined immunodeficiency (28.6% and 23.8%), combined immunodeficiency (25% and 15%), familial hemophagocytic lymphohistiocytosis (40% and 20%), X-linked lymphoproliferative diseases-1 (75% and 75%) and X-linked lymphoproliferative diseases-2 (50% and 50%) compared to the other IEIs cases.

**Conclusion:**

Children with IEIs infected with SARS-CoV-2 may experience higher rates of ICU admission and mortality in comparison with the immunocompetent pediatric populations. Underlying immune defects does seem to be independent risk factors for severe SARS-CoV-2 infection in children with IEIs, a number of children with SCID and CID were reported to have prolonged infections–though the number of patients is small–but especially immune dysregulation diseases (XLP1 and XLP2) and innate immunodeficiencies impairing type I interferon signalling (IFNAR1, IFNAR2 and TBK1).

**Supplementary Information:**

The online version contains supplementary material available at 10.1186/s13223-023-00831-1.

## Background

Since our knowledge on the multiple aspects and complications of severe acute respiratory syndrome coronavirus 2 (SARS-CoV-2), such as multisystem inflammatory syndrome in children (MIS-C), has grown gradually during the coronavirus disease 2019 (COVID-19) pandemic, some relevant features of the disease especially in children were not highlighted in early case reports and small series published. Inborn errors of immunity (IEIs), formerly called primary immunodeficiency disorders, are a growing group of hundreds of disorders [1]. IEIs range considerably in severity from mild infections to serious multisystemic disease [2]. A group of nearly 500 IEIs have been described by the expert committee of the International Union of Immunological Societies (IUIS) [1]. While individually rare, IEIs are considered significant challenges for patients with IEIs, their families, and their medical providers; and children with IEIs present clinically as increased susceptibility to infections, autoimmunity, autoinflammatory diseases, allergy, bone marrow failure, and/or malignancy [3]. Very few sporadic cases of IEIs in children with SARS-CoV-2 infection have been reported worldwide [4–10]. Several previous systematic reviews have reported on the association between IEIs and COVID-19; however, these studies included mixed populations of adults and children, and included a smaller number of studies (with most data for adults and very few pediatric patients) [11–19], Moreover, only some of these reviews covered the occurrence of COVID-19 in patients with all categories of IEIs as compiled by the IUIS [11–14, 19]. Few reviews evaluated clinical course of SARS-CoV-2 infection in patients with IEIs with the limitation of focusing on one major category or subcategory of IEIs such as cellular and humoral immunodeficiencies [17], common variable immunodeficiency [16, 18] or DNA repair defects [15]. Due to the lack of comprehensive and updated systematic reviews focusing on the development of those two medical conditions, we aimed to estimate the incidence of SARS-CoV-2 infection in children with IEIs and analyse the demographic parameters, clinical characteristics and treatment outcomes in those IEIs cases with pediatric COVID-19 illness, with larger and better-quality data. We expect our review to provide clinicians with a thorough understanding of the clinical course and outcome of hundreds of children with IEIs infected with SARS-CoV-2 and predisposing factors and immunological mechanisms underlying severe COVID-19.

## Methods

### Design

We performed this systematic review following the recommendations of the Preferred Reporting Items for Systematic Reviews and Meta-Analyses guidelines (PRISMA) [20]. We searched for observational studies published from 1 December 2019 until 28 February 2023, in PROQUEST, MEDLINE, EMBASE, PUBMED, CINAHL, WILEY ONLINE LIBRARY, SCOPUS and NATURE, with a restriction to articles published in the English language. Search terms were based on the IUIS classification of human IEIs [1] (see Additional file 1 for complete search strategies and IEIs in each inborn error of immunity class included in Additional file 1: Tables S1, S2). Articles discussing and reporting the development of COVID-19 in children with IEIs were selected based on the title and abstract.

### Inclusion–exclusion criteria

The eligible studies were included based on the following inclusion criteria: (1) published case reports, case-series, case–control and cohort studies that focused on development of COVID-19 in IEIs patients that included children as a population of interest; (2) studies of an experimental or observational design reporting the incidence of SARS-CoV-2 infection in pediatric patients with IEIs. The exclusion criteria included: (1) editorials, commentaries, reviews and meta-analyses; (2) studies that reported IEIs in children with negative SARS-CoV-2 polymerase chain reaction (PCR) tests; (3) studies that reported IEIs in adult COVID-19 patients.

### Data extraction

The screening of the papers was performed independently by six reviewers (Saad Alhumaid, Zainah Sabr, Ola Alkhars, Muneera Alabdulqader, Fatemah M. ALShakhs, and Rabab Abbas Majzoub) by screening the titles with abstracts using the selection criteria. Disagreements in the study selection after the full-text screening were discussed; if agreement could not be reached, a third reviewer was involved. We categorized articles as case report, case-series, case–control or cohort studies. The following data were extracted from the selected studies: authors; publication year; study location; study design and setting; age; proportion of male patients; patient ethnicity; identified IEIs; main genetic cause of IEIs; other potential modifiers in immunity-related pathways or specific allele change; IEIs mode of inheritance; COVID-19 severity, if patient experienced multisystem inflammatory syndrome in children (MIS-C), and comorbidities; laboratory findings; IEIs treatment at SARS-CoV-2 infection; if patient was admitted to the intensive care unit (ICU), placed on mechanical ventilation and/or suffered acute respiratory distress syndrome (ARDS); assessment of study risk of bias; and final treatment outcome (survived or died); and they are noted in Additional file 2: Table S3 (see Additional file 2 for summary of the characteristics of the included studies with evidence on IEIs and COVID-19 in pediatric patients, n = 116 studies, 2020–2022).

### Quality assessment

Two tools were used appropriately to assess the quality of the studies included in this review: (1) Newcastle–Ottawa Scale (NOS) to evaluate cohort and case–control studies (scoring criteria: > 7 scores = high quality, 5–7 scores = moderate quality, and < 5 scores = low quality) [21]; and (2) modified NOS to evaluate case report and case-series studies (scoring criteria: 5 criteria fulfilled = good, 4 criteria fulfilled = moderate, and 3 criteria fulfilled = low) [22]. Quality assessment was conducted by six co-authors (Yousef Hassan Alalawi, Khalid Al Noaim, Abdulrahman A. Alnaim, Mohammed A. Al Ghamdi, Abdulaziz A. Alahmari, and Sawsan Sami Albattat) who separately evaluated the possibility of bias using these two tools.

### Data analysis

We examined primarily the proportion of confirmed SARS-CoV-2 infection in patients with IEIs. This proportion was further classified based on 2022 updated classification of IEIs (i.e., identified IEIs cases were categorized into 10 Tables with subtables segregating groups of disorders into overlapping phenotypes), as compiled by the expert committee of the IUIS [1]. Clinical Spectrum of SARS-CoV-2 Infection from the National Institutes of Health was applied to define severity of COVID-19 (asymptomatic, mild, moderate, severe and critical) [23]. MIS-C was defined according to the current United States Centers for Disease Control and Prevention case definition in an individual aged < 21 years [24].

Descriptive statistics were used to describe the data. For continuous variables, mean and standard deviation were used to summarize the data; and for categorical variables, frequencies and percentages were reported. Microsoft Excel 2019 (Microsoft Corp., Redmond, USA) was used for all statistical analyses.

## Results

### Study characteristics and quality

**Fig. 1:**
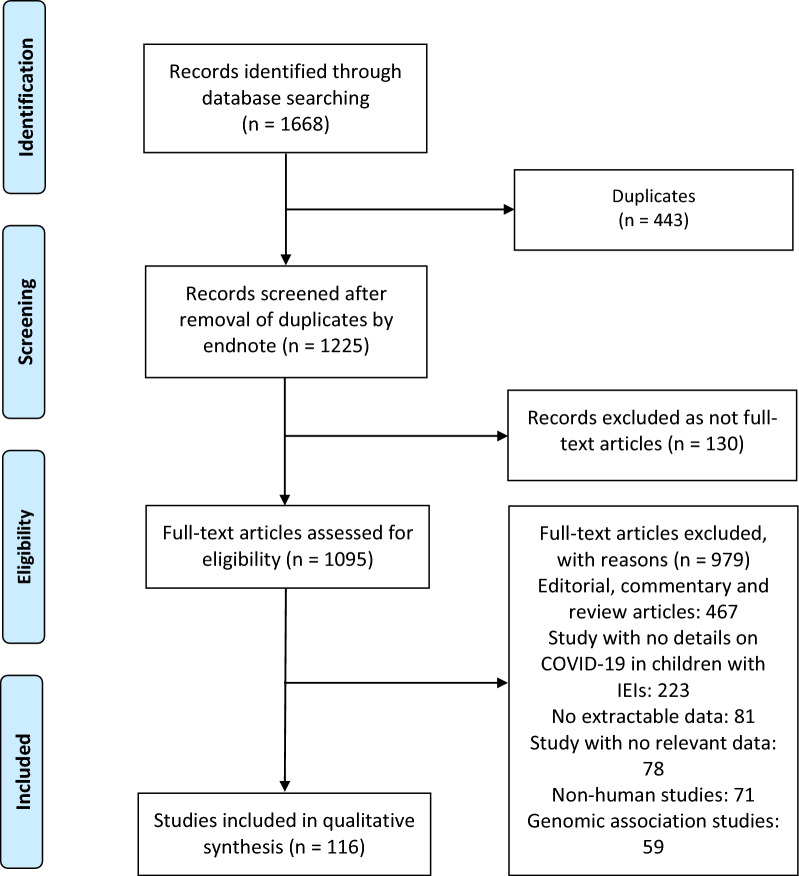
Flow diagram of studies included in the systematic review

A total of 3952 publications were identified (Fig. 1). After exclusion of duplicates and articles that did not fulfill the study inclusion criteria, one hundred and sixteen articles were included in the qualitative synthesis of this systematic review [4–10, 19, 25–132]. The reports of seven hundred and ten cases identified from these articles are presented by groups based on 2022 updated classification of IEIs as described by IUIS [1]. The detailed characteristics of the included studies are shown in Additional file 2: Table S3. There were 73 case report [4–6, 8, 9, 25–32, 37–39, 44, 45, 49, 51, 53–60, 67–69, 72, 76–79, 82–84, 86–88, 90, 91, 93, 94, 96, 99–107, 109–114, 116, 119–123, 126, 127, 129–131], 38 cohort [7, 10, 19, 33–36, 40–43, 46–48, 50, 52, 61–66, 70, 71, 73, 74, 80, 81, 92, 95, 97, 98, 108, 117, 118, 124, 125, 132], 4 case-series [85, 89, 115, 128], and 1 case–control [75] studies. These studies were conducted in United States (n = 27), Iran (n = 15), India (n = 10), United Kingdom (n = 8), Italy (n = 8), Turkey (n = 8), Germany (n = 6), Brazil (n = 4), Mexico (n = 3), Israel (n = 3), Spain (n = 3), Saudi Arabia (n = 2), Poland (n = 2), Greece (n = 2), Belgium (n = 2), Switzerland (n = 1), Sweden (n = 1), Hong Kong (n = 1), Peru (n = 1), United Arab Emirates (n = 1), Tunisia (n = 1), and France (n = 1). Only six studies were made within multi-countries (n = 6) [10, 33, 35, 51, 95, 132]. The majority of the studies were single centre [4–6, 8, 9, 19, 25–32, 34, 37–39, 41, 43–47, 49, 50, 52–60, 62, 64, 66–69, 71, 72, 75–80, 82–84, 86–90, 93, 94, 96, 98–117, 119–131] and only 23 studies were multi-centre [7, 10, 33, 35, 36, 40, 42, 48, 51, 61, 63, 65, 70, 73, 74, 81, 85, 91, 92, 95, 97, 118, 132]. Among all included studies in our systematic review, only one study reported on the other potential modifiers in immunity-related pathways for all diagnosed IEIs in children who were infected with SARS-CoV-2 (n = 1, 0.9%) [19]. All case reports and case-series studies were assessed for bias using the modified NOS. Sixty-seven studies were deemed to have high methodological quality, 7 moderate methodological quality, and 3 low methodological quality. Among the 116 included studies, 38 cohort studies were assessed using the NOS: 31 studies were found to be moderate-quality studies (i.e., NOS scores between 5 and 7) and 7 study demonstrated a relatively high quality (i.e., NOS scores > 7); Additional file 2: Table S3.

### Predominantly antibody deficiencies

Predominantly antibody deficiencies were the first most-common IEIs in children who experienced COVID-19 (n = 197, 27.7%) [8, 19, 27, 28, 34, 36, 40, 42, 45, 46, 49, 50, 52, 55, 62–66, 71, 73, 74, 80, 81, 83, 85, 91, 92, 95, 97, 105, 108, 114, 117, 120, 122, 124, 125, 129, 130] (see Additional file 2: Table S3). Among them, 53 have common variable immunodeficiency (CVID, 26.9% of all predominantly antibody deficiencies) [19, 27, 28, 34, 36, 40, 46, 50, 62–66, 73, 74, 80, 95, 97, 114, 122, 124, 125], 45 have X-linked agammaglobulinemia (XLA, 22.8%) [8, 19, 34, 40, 42, 46, 49, 50, 52, 63, 65, 71, 73, 74, 85, 91, 92, 95, 97, 105, 117, 120, 129, 130], 41 have autosomal recessive or autosomal dominant agammaglobulinemia or hypogammaglobulinemia (20.8%) [45, 52, 55, 63, 65, 80, 83, 124], 28 have isolated IgG subclass deficiencies (14.2%) [81, 108], and 12 have selective IgA deficiencies (6.1%) [46, 48, 52, 65, 81, 124]. The remaining 18 patients have other isotype, light chain, or functional deficiencies with generally normal numbers of B cells [including IgG subclass deficiency with IgA and/or IgM deficiency (n = 2) [108], IgG, IgA and IgM deficiencies (n = 2) [108], partial IgA deficiency (n = 1) [124], and low IgM level (n = 1) [124]]; selective IgM deficiencies (n = 3) [81, 108]; specific antibody deficiency with normal immunoglobulin and B cells levels (n = 4) [40, 65], unspecified predominantly antibody deficiency (n = 3) [65, 95]; UNG deficiency (n = 1) [19] and APRIL deficiency (n = 1) [40]. The most frequent main genetic causes of predominantly antibody deficiencies in children infected with SARS-CoV-2 were BTK (n = 41) [8, 19, 34, 40, 46, 49, 50, 52, 63, 65, 71, 73, 74, 85, 91, 92, 95, 97, 105, 117, 120, 129], NFKB2 (n = 4) [27, 36, 95, 122], TNFRSF13B (n = 3) [62, 114, 125], PIK3CD (n = 3) [19, 65, 97], and PIK3R1 (n = 2) [45, 63]. For patients with predominately antibody deficiencies who acquired SARS-CoV-2, the median interquartile range (IQR) age was 120 months [83–175], with a male predominance [n = 94, 47.7%] [8, 19, 27, 28, 34, 40, 42, 45, 46, 49, 50, 52, 55, 62, 63, 65, 66, 71, 73, 74, 80, 85, 91, 92, 95, 105, 108, 117, 120, 125, 129, 130], and majority of the patients belonged to White (Caucasian) (n = 144, 73.1%) [8, 27, 36, 42, 46, 50, 52, 55, 62–64, 66, 71, 73, 80, 81, 83, 85, 95, 97, 105, 108, 114, 120, 122, 124, 129], Hispanic (n = 30, 15.2%) [40, 65, 95, 125] and Persian (n = 15, 7.6%) [19, 28, 34, 48, 74, 117] ethnicity. In those predominantly antibody deficiencies patients, few studies reported on specific allele changes (n = 9, 4.6%) [27, 36, 49, 62, 114, 120, 122, 125, 129]. Reported modes of inheritance for the predominantly antibody deficiencies in children were autosomal recessive (n = 62, 31.5%) [19, 28, 34, 40, 46, 50, 63, 73], X-linked (n = 46, 23.3%) [8, 19, 34, 40, 42, 46, 49, 50, 52, 63, 65, 71, 73, 74, 85, 91, 92, 95, 97, 105, 117, 120, 129, 130], or autosomal dominant (n = 15, 7.6%) [19, 27, 36, 45, 52, 62, 63, 65, 83, 95, 97, 114, 122, 125], however, mode of inheritance in these predominantly antibody deficiencies cases was unknown in a high percentage of patients (n = 120, 60.9%) [40, 46, 48, 52, 55, 63–66, 74, 80, 81, 95, 97, 108, 124]. COVID-19 in children with predominately antibody deficiencies was asymptomatic (32/197 = 16.2%) [50, 63, 65, 73, 81, 92, 95, 97, 108, 124], mild (120/197 = 61%) [28, 34, 40, 42, 46, 48, 49, 52, 55, 63, 65, 66, 71, 73, 74, 80, 81, 83, 85, 91, 92, 95, 97, 108, 120, 124, 130], moderate (21/197 = 10.6%) [40, 46, 52, 64, 74, 81, 85, 91, 95, 117, 122, 124, 129], severe (14/197 = 7.1%) [8, 19, 27, 36, 45, 65, 81, 91, 105, 114] or critical (2/197 = 1%) [19, 65]. Most children with predominately antibody deficiencies did not get MIS-C due to COVID-19 (178/197, 90.3%) [8, 19, 28, 34, 40, 42, 46, 48–50, 52, 55, 63–66, 71, 73, 80, 81, 83, 85, 92, 95, 97, 105, 108, 117, 120, 122, 124, 129, 130], however, few children with predominately antibody deficiencies were reported to experience MIS-C (9/197, 4.6%) [19, 27, 36, 45, 62, 108, 114, 125]. Few of those predominantly antibody deficiencies cases presented with a previous known history of chronic lung disease (n = 5) [40, 46, 64, 74, 95], chronic heart disease (n = 4) [55, 63, 65, 95], arthritis (n = 4) [49, 52, 114, 122], hypertension (n = 3) [55, 124], Down syndrome (n = 3) [55, 65, 108], autoimmune haemolytic anaemia (n = 2) [40, 52], asthma (n = 2) [52, 124], immune thrombocytopenic purpura (n = 2) [46, 124], hypercholesterolemia (n = 2) [63, 95], hereditary spherocytosis (n = 2) [8, 95], Chromosome 18q deletion (n = 2) [64, 66], seizures (n = 2) [40, 114], psoriasis (n = 2) [27, 95], hemophagocytic lymphohistiocytosis (n = 2) [95], celiac disease (n = 2) [46, 80] or obesity (n = 2) [65, 124]. Patients who suffered predominantly antibody deficiencies and experienced COVID-19 were maybe more likely to have low serum immunoglobulin G levels (n = 29) [19, 36, 66, 108], low serum immunoglobulin A levels (n = 10) [19, 28, 36, 49, 66, 80, 85], high C-reactive protein (n = 8) [8, 27, 48, 55, 64, 71, 73, 105], low serum immunoglobulin M levels (n = 5) [19, 28, 66, 85], lymphopenia (n = 5) [27, 45, 64, 73], thrombocytopenia (n = 5) [8, 40, 45, 55], high ESR (n = 4) [8, 45, 48, 105], raised liver enzymes (n = 3) [27, 55, 114], hypogammaglobulinemia (n = 3) [27, 36, 80], and high D-dimer (n = 3) [27, 55, 105]. As expected, most prescribed therapeutic agents in these predominantly antibody deficiencies cases were intravenous immunoglobulin (n = 49, 24.9%) [8, 19, 27, 28, 34, 40, 42, 45, 48, 49, 52, 63, 65, 73, 74, 80, 85, 91, 92, 95, 105, 129], antibiotics (n = 38, 19.3%) [8, 19, 27, 28, 34, 42, 45, 48, 49, 52, 55, 63, 73, 74, 85, 91, 95, 105, 117, 129], oxygen supplementation (n = 14, 7.1%) [8, 19, 27, 28, 45, 52, 55, 64, 71, 95, 108, 117, 122, 124], steroids (n = 16, 8.1%) [19, 27, 34, 40, 45, 52, 55, 64, 95, 114, 117, 122], convalescent plasma (n = 12, 6.1%) [8, 19, 27, 71, 85, 91, 95, 108, 122], and remdesivir (n = 12, 6.1%) [8, 27, 42, 45, 64, 85, 95, 105, 117, 122], however, treatment was not necessary in a high number of these predominantly antibody deficiencies patients (n = 59, 29.9%) [40, 46, 50, 63, 65, 66, 80, 83, 108, 120, 124, 130]. There were predominantly antibody deficiencies patients who were admitted to the intensive care units (n = 16, 8.1%) [19, 27, 36, 40, 45, 55, 64, 65, 95, 114], intubated and placed on mechanical ventilation (n = 9, 4.6%) [19, 27, 36, 40, 45, 65, 114] and suffered acute respiratory distress syndrome (n = 13, 6.6%) [19, 27, 36, 40, 45, 65, 114, 117, 122]. Clinical outcomes of the predominantly antibody deficiencies patients with mortality were documented in 5 (2.5%) [19, 40, 65, 114], while 190 (96.4%) of the predominantly antibody deficiencies cases recovered [8, 19, 27, 28, 34, 36, 40, 42, 45, 46, 48–50, 52, 55, 63–66, 71, 73, 74, 80, 81, 83, 85, 91, 92, 95, 97, 105, 108, 117, 120, 122, 124, 129, 130] and final treatment outcome was not reported in two predominantly antibody deficiencies patients (n = 2, 1%) [62, 125]. Mortality was COVID-19-related in two children with predominately antibody deficiencies (2/197, 1%) [65, 114], however, COVID-19 was not attributable to death in one child with the reported predominately antibody deficiencies (1/197, 0.5%) [40] and one study failed to report if COVID-19 was a leading or an underlying cause of death in two children with predominately antibody deficiencies (2/197, 1%) [19] (see Table 1).

**Table 1:** Pediatric patients with IEIs affected by COVID-19, stratified by type of immune defect and treatment outcome (n = 116 studies), 2020–2022

Type of IEIs	Number of patients^a^	ICU admission rate^a^	Use of MV^a^	Suffered ARDS^a^	Case fatality rate^a^
Predominantly antibody deficiencies	197 (27.7)	16 (8.1)	9 (4.6)	13 (6.6)	5 (2.5)
CVID	53 (26.9)	9 (17)	5 (9.4)	7 (13.2)	3 (5.7)
XLA	45 (22.8)	2 (4.4)	2 (4.4)	4 (8.9)	2 (4.4)
AR or AD agammaglobulinemia/hypogammaglobulinemia	41 (20.8)	2 (4.9)	1 (2.4)	1 (2.4)	0
Isolated IgG subclass deficiencies	28 (14.2)	0	0	0	0
Selective IgA deficiencies	12 (6.1)	0	0	0	0
Specific antibody deficiency with normal Ig and B cells levels	4 (2)	1 (25)	1 (25)	1 (25)	0
Selective IgM deficiencies	3 (1.5)	0	0	0	0
Unspecified predominantly antibody deficiency	3 (1.5)	0	0	0	0
IgG subclass deficiency with IgA and/or IgM deficiency	2 (1)	0	0	0	0
IgG, IgA and IgM deficiencies	2 (1)	0	0	0	0
Partial IgA deficiency	1 (0.5)	0	0	0	0
Low IgM level	1 (0.5)	0	0	0	0
UNG deficiency	1 (0.5)	1 (100)	0	0	0
APRIL deficiency	1 (0.5)	1 (100)	0	0	0
Combined immunodeficiencies with associated or syndromic features	126 (17.7)	11 (8.7)	9 (7.1)	9 (7.1)	7 (5.5)
DiGeorge syndromes	40 (31.7)	1 (2.5)	0	0	0
Immunodeficiency with ataxia-telangiectasia	25 (19.8)	0	0	0	0
Wiskott-Aldrich syndromes	14 (11.1)	2 (14.3)	2 (14.3)	2 (14.3)	1 (7.1)
Nijmegen breakage syndromes	9 (7.1)	0	0	0	0
Unspecified hyper IgM syndromes	7 (5.5)	1 (14.3)	1 (14.3)	1 (14.3)	1 (14.3)
Immunodeficiencies with centromeric instability and facial anomalies	6 (4.8)	2 (33.3)	2 (33.3)	2 (33.3)	1 (16.7)
Unspecified hyper IgE syndromes	5 (4)	1 (20)	1 (20)	1 (20)	1 (20)
ARPC1B deficiency	3 (2.4)	0	0	0	0
Hypoparathyroidism-retardation-dysmorphism syndromes	3 (2.4)	2 (66.7)	2 (66.7)	2 (66.7)	2 (66.7)
STIM1 deficiencies	2 (1.6)	1 (50)	1 (50)	1 (50)	1 (50)
EDA-ID caused by hypomorphic mutations in encoding the nuclear factor κB essential modulator (NEMO) protein	2 (1.6)	0	0	0	0
MCM4 deficiencies	2 (1.6)	nr	nr	nr	nr
Kabuki syndrome	2 (1.6)	0	0	0	0
PGM3 deficiency	2 (1.6)	0	0	0	0
ORAI-1 deficiency	1 (0.8)	nr	nr	nr	nr
TBX1 deficiency	1 (0.8)	1 (100)	0	0	0
Bloom syndrome	1 (0.8)	0	0	0	0
Schimke immuno-osseous dysplasia	1 (0.8)	0	0	0	0
Cellular and humoral immunodeficiencies	102 (14.4)	27 (26.5)	23 (22.5)	23 (22.5)	19 (18.6)
CID	60 (58.8)	15 (25)	12 (20)	12 (20)	9 (15)
SCID	42 (41.2)	12 (28.6)	11 (26.2)	11 (26.2)	10 (23.8)
Immune dysregulatory diseases	95 (13.4)	34 (35.8)	25 (26.3)	27 (28.4)	17 (17.9)
FHL syndromes	25 (26.3)	10 (40)	7 (28)	6 (24)	5 (20)
APS-1	19 (20)	8 (42.1)	6 (31.6)	9 (47.4)	1 (5.3)
ALPS	9 (9.5)	0	0	0	0
LRBA deficiency	6 (6.3)	2 (33.3)	2 (33.3)	2 (33.3)	1 (16.7)
TPP2 deficiency	5 (5.3)	0	0	0	0
Unspecified immune dysregulatory disease	5 (5.3)	1 (20)	1 (20)	1 (20)	1 (20)
XLP1	4 (4.2)	3 (75)	3 (75)	3 (75)	3 (75)
XLP2	4 (4.2)	2 (50)	2 (50)	2 (50)	2 (50)
SOCS1 deficiency	2 (2.1)	0	0	0	0
CTLA4 deficiency	2 (2.1)	0	0	0	0
Interleukin-10Ra deficiency	2 (2.1)	0	0	0	0
BACH2 deficiency	2 (2.1)	2 (100)	0	0	0
RLTPR deficiency	2 (2.1)	1 (50)	1 (50)	1 (50)	1 (50)
NOTCH1 mutation	1 (1)	1 (100)	0	0	0
ALPS-Caspase10	1 (1)	1 (100)	0	0	0
CD137 deficiency	1 (1)	1 (100)	1 (100)	1 (100)	1 (100)
Interleukin-37 deficiency	1 (1)	1 (100)	1 (100)	1 (100)	1 (100)
IPEX syndrome	1 (1)	1 (100)	1 (100)	1 (100)	1 (100)
Prolidase deficiency	1 (1)	nr	nr	nr	nr
PRKCD deficiency	1 (1)	0	0	0	0
MAGT1 deficiency	1 (1)	0	0	0	0
Autoinflammatory diseases	67 (9.4)	3 (4.5)	2 (3)	2 (3)	2 (3)
FMF	36 (53.7)	0	0	0	0
Blau syndrome	4 (6)	nr	nr	nr	nr
Aicardi-Goutières syndrome	3 (4.5)	0	0	0	0
Familial cold autoinflammatory syndromes 1	3 (4.5)	0	0	0	0
ADA2 deficiency	2 (3)	0	0	0	0
NLRP1 deficiency	2 (3)	1 (50)	0	0	0
TNF receptor-associated periodic syndrome	2 (3)	0	0	0	0
Hyperpigmentation hypertrichosis, histiocytosis-lymphadenopathy plus syndrome SLC29A3 mutation	2 (3)	0	0	0	0
RNASEH2B deficiency	2 (3)	0	0	0	0
Unspecified autoinflammatory diseases	2 (3)	1 (50)	1 (50)	1 (50)	1 (50)
Familial cold autoinflammatory syndrome 4	1 (1.5)	0	0	0	0
Deficiency of the interleukin 1 receptor antagonist	1 (1.5)	1 (100)	1 (100)	1 (100)	1 (100)
PAPA syndrome, hyperzincemia and hypercalprotectinemia	1 (1.5)	nr	nr	nr	nr
Mevalonate kinase deficiency	1 (1.5)	0	0	0	0
SAMHD1 deficiency	1 (1.5)	0	0	0	0
A20 deficiency	1 (1.5)	0	0	0	0
Majeed syndrome	1 (1.5)	nr	nr	nr	nr
STING-like disease	1 (1.5)	nr	nr	nr	nr
CARD14 mediated psoriasis	1 (1.5)	nr	nr	nr	nr
Phagocytic diseases	54 (7.6)	4 (7.4)	3 (5.5)	5 (9.2)	3 (5.5)
CGD	26 (48.1)	3 (11.5)	2 (7.7)	4 (15.4)	2 (7.7)
Shwachman-Diamond syndromes	8 (14.8%)	0	0	0	0
HAX1 deficiencies	6 (11.1%)	0	0	0	0
Unspecified phagocytic diseases	4 (7.4)	0	0	0	0
Glycogen storage diseases type 1b	2 (3.7)	nr	nr	nr	nr
Elastase deficiency	2 (3.7)	0	0	0	0
JAGN1 deficiency	1 (1.8)	0	0	0	0
Poikiloderma with neutropenia	1 (1.8)	0	0	0	0
Cystic fibrosis	1 (1.8)	1 (100)	1 (100)	1 (100)	1 (100)
Leukocyte adhesion deficiency type 3	1 (1.8)	0	0	0	0
GATA2 deficiency	1 (1.8)	0	0	0	0
Undefined leukopenia	1 (1.8)	0	0	0	0
Innate immunodeficiencies	50 (7)	19 (38)	14 (28)	17 (34)	5 (10)
TLR7 deficiency	8 (16)	6 (75)	5 (62.5)	5 (62.5)	0
MyD88 deficiency	7 (14)	3 (42.8)	2 (28.6)	2 (28.6)	0
STAT1 deficiency	7 (14)	0	0	0	0
Unspecified innate immunodeficiencies	5 (10)	0	0	0	0
IFNAR1 deficiency	3 (6)	3 (100)	2 (66.7)	3 (100)	2 (66.7)
IFNAR2 deficiency	3 (6)	3 (100)	3 (100)	3 (100)	2 (66.7)
TYK2 deficiency	3 (6)	2 (66.7)	0	2 (66.7)	0
TBK1 deficiency	2 (4)	1 (50)	1 (50)	1 (50)	1 (50)
Isolated congenital asplenia	2 (4)	0	0	0	0
IFN-g receptor 2 deficiency	2 (4)	1 (50)	1 (50)	1 (50)	0
MDA5 deficiency	2 (4)	nr	nr	nr	nr
TRIF deficiency	1 (2)	0	0	0	0
WHIM syndrome	1 (2)	0	0	0	0
IRAK4 deficiency	1 (2)	0	0	0	0
IRF9 deficiency	1 (2)	0	0	0	0
STAT2 deficiency	1 (2)	0	0	0	0
Interleukin-12 and interleukin-23 receptor b1 chain deficiency	1 (2)	0	0	0	0
Complement deficiencies	11 (1.5)	3 (27.3)	1 (9.1)	1 (9.1)	1 (9.1)
Factor H deficiency	3 (27.3)	2 (66.7)	1 (33.3)	1 (33.3)	1 (33.3)
C1 inhibitor deficiency	3 (27.3)	0	0	0	0
Ficolin 3 deficiency	2 (18.2)	nr	nr	nr	nr
Factor H –related protein deficiency	1 (9.1)	1 (100)	0	0	0
Factor I deficiency	1 (9.1)	nr	nr	nr	nr
C3 deficiency	1 (9.1)	0	0	0	0
Bone marrow failure	7 (1)	2 (28.6)	1 (14.3)	1 (14.3)	1 (14.3)
Fanconi anaemia	5 (71.4)	1 (20)	1 (20)	1 (20)	1 (20)
SAMD9 deficiency	1 (14.3)	0	0	0	0
DKCA1 deficiency	1 (14.3)	1 (100)	0	0	0
Phenocopies of primary immunodeficiencies	1 (0.1)	0	0	0	0
RAS-associated autoimmune leukoproliferative disease	1 (100)	0	0	0	0
Total	**710**	**119 (16.8)**	**87 (12.2)**	**98 (13.8)**	**60 (8.4)**

*AD* autosomal dominant, *ALPS* autoimmune lymphoproliferative syndrome, *APS-1* autoimmune polyendocrine syndromes type-1, *AR* autosomal recessive; *ARDS* acute respiratory distress syndrome; *CGD* chronic granulomatous disease; *CID* combined immunodeficiency; *COVID-19* coronavirus disease 2019, *CVID* common variable immunodeficiency, *EDA-ID* anhidrotic ectodermodysplasia with immunodeficiency, *FHL* familial hemophagocytic lymphohistiocytosis, *FMF* familial Mediterranean fever, *ICU* intensive care unit, *IEIs* inborn errors of immunity, *IFN* interferon, *IFNAR* interferon alpha/beta receptor subunit, *Ig* immunoglobulin, *IgA* immunoglobulin A, *IgE* immunoglobulin E, *IgG* immunoglobulin G, *IgM* immunoglobulin M, *IPEX* immune-dysregulation polyendocrinopathy X-linked, *MV* mechanical ventilation, *nr* not reported, *PAPA* pyogenic sterile arthritis, pyoderma gangrenosum, acne, *SCID* severe combined immunodeficiencies, *TLR* toll-like receptors, *TNF* tumor necrosis factor, *TPP2* tripeptidyl-Peptidase II, *WHIM. warts* hypogammaglobulinemia, infections, and myelokathexis, *XLA* X-linked agammaglobulinemia, *XLP* X-linked lymphoproliferative disease

^a^Data are presented as number (%). Data was calculated on patients for whom the information was available

Percentages do not total 100% owing to missing data

### Combined immunodeficiencies with associated or syndromic features

Combined immunodeficiencies with associated or syndromic features were the second most-common IEIs in children who experienced COVID-19 (n = 126, 17.7%) [5, 19, 30, 34, 39, 40, 46–48, 52, 56, 62–65, 73, 74, 76, 81, 92, 95, 97, 99, 108, 112, 117, 118, 123–125] (see Additional file 2: Table S3). Among them, 40 have DiGeorge syndromes (31.7% of all syndromic combined immunodeficiencies) [46, 56, 63, 65, 81, 92, 95, 97, 108], 25 have immunodeficiency with ataxia-telangiectasia (19.8%) [34, 46, 48, 52, 62–64, 73, 74, 81, 97, 108, 117, 118, 124], and 14 have Wiskott-Aldrich syndromes (WAS) (11.1%) [5, 40, 46, 48, 63, 65, 81, 95, 99, 124]. The remaining 47 patients have Nijmegen breakage syndromes (n = 9) [81, 108]; immunodeficiencies with centromeric instability and facial anomalies (n = 6) [19, 34, 48, 73]; ARPC1B deficiency (n = 3) [39, 40, 95]; STIM1 deficiencies (n = 2) [34, 74]; anhidrotic ectodermodysplasia with immunodeficiency caused by hypomorphic mutations in encoding the nuclear factor κB essential modulator (NEMO) protein (n = 2) [30, 73]; hypoparathyroidism-retardation-dysmorphism syndromes (n = 3) [47]; MCM4 deficiencies (n = 2) [62]; Kabuki syndrome (n = 2) [63, 108]; PGM3 deficiency (n = 2) [76, 95]; ORAI-1 deficiency (n = 1) [125]; TBX1 deficiency (n = 1) [19]; Bloom syndrome (n = 1) [46]; Schimke immuno-osseous dysplasia (n = 1) [46]; unspecified hyper IgM syndromes (n = 7) [48, 65, 74, 92, 112, 123] and unspecified hyper IgE syndromes (n = 5) [34, 46]. The most frequent main genetic causes of combined immunodeficiencies with associated or syndromic features in children infected with SARS-CoV-2 were large (3 Mb) deletion of 22q11.2 (n = 40) [46, 56, 63, 65, 81, 92, 95, 97, 108], ATM deficiency (n = 24) [34, 46, 48, 52, 62–64, 73, 74, 81, 97, 108, 117, 118, 124], Wiskott-Aldrich syndrome protein deficiency (n = 14) [5, 40, 46, 48, 63, 65, 81, 95, 99, 124], NBS1 (n = 9) [81, 108], DNMT3B (n = 5) [19, 48, 73], ARPC1B (n = 3) [39, 40, 95], STIM1 (n = 2) [34, 74], IKBKG (n = 2) [30, 73], and PGM3 (n = 2) [76, 95]. For patients with combined immunodeficiencies with associated or syndromic features who acquired SARS-CoV-2, the median interquartile range (IQR) age was 90 months [25.7 to 142.5], with a male predominance [n = 61, 48.4%] [34, 46–48, 52, 56, 62, 64, 65, 73, 74, 108, 112, 117, 118, 125], and majority of the patients belonged to White (Caucasian) (n = 91, 72.2%) [5, 30, 46, 52, 62–64, 73, 76, 81, 95, 97, 108, 118, 123, 124], Persian (n = 18, 14.3%) [19, 34, 48, 74, 112, 117] and Hispanic (n = 11, 8.7%) [39, 40, 56, 65, 95, 125] ethnicity. In those combined immunodeficiencies with associated or syndromic features patients, few studies reported on specific allele changes (n = 5, 4%) [39, 47, 62, 99, 125]. Reported modes of inheritance for the combined immunodeficiencies with associated or syndromic features in children were autosomal recessive (n = 61, 48.4%) [19, 34, 39, 40, 46–48, 52, 62–64, 73, 74, 76, 81, 95, 97, 108, 117, 118, 124, 125], autosomal dominant (n = 41, 32.5%) [19, 46, 56, 63, 65, 81, 92, 95, 97, 108], or X-linked (n = 18, 14.3%) [5, 30, 40, 46, 48, 63, 65, 73, 81, 95, 99, 108, 124], however, mode of inheritance in these combined immunodeficiencies with associated or syndromic features cases was unknown in a low percentage of patients (n = 6, 4.8%) [65, 74, 92, 112, 123]. COVID-19 in children with combined immunodeficiencies with associated or syndromic features was asymptomatic (20/126 = 15.9%) [34, 56, 63, 65, 81, 92, 95, 97, 123, 124], mild (66/126 = 52.4%) [5, 34, 40, 46, 48, 52, 63–65, 73, 74, 76, 81, 92, 95, 97, 99, 108, 117], moderate (14/126 = 11.1%) [34, 40, 46, 48, 63, 74, 81, 112, 124], severe (9/126 = 7.1%) [19, 30, 34, 39, 48, 65, 97] or critical (1/126 = 0.8%) [65]. Most children with combined immunodeficiencies with associated or syndromic features did not get MIS-C due to COVID-19 (105/126, 83.3%) [5, 19, 34, 39, 40, 46, 48, 52, 56, 63–65, 73, 76, 81, 92, 95, 97, 99, 108, 112, 117, 123, 124], however, few children with combined immunodeficiencies with associated or syndromic features were reported to experience MIS-C (11/126, 8.7%) [19, 30, 34, 48, 62, 125]. Few of those combined immunodeficiencies with associated or syndromic features cases presented with a previous known history of cardiopathy (n = 7) [46], chronic heart disease (n = 5) [56, 63, 65], chronic lung disease (n = 3) [40, 64, 124], post hematopoietic stem cell transplant (n = 3) [40, 46, 95], inflammatory bowel disease (n = 3) [40, 73], cognitive disability (n = 3) [63, 95], obesity (n = 3) [56, 63, 65], hypoparathyroidism (n = 2) [63], hypothyroidism (n = 2) [63], developmental delay (n = 2) [46, 47, 63], autoimmune haemolytic anaemia (n = 2) [34, 74], vasculitis (n = 2) [63, 74], gastroesophageal reflux disease (n = 2) [46, 52], hypertension (n = 2) [65], myopathy (n = 2) [34, 74], sepsis or septic shock (n = 2) [39, 95] or nephrotic syndrome (n = 2) [34, 74]. Patients who suffered combined immunodeficiencies with associated or syndromic features and experienced COVID-19 were maybe more likely to have high C-reactive protein (n = 9) [30, 48, 73], high erythrocyte sedimentation rate (n = 8) [30, 48, 73, 112], low serum immunoglobulin M level (n = 5) [19, 52, 56], low serum immunoglobulin A level (n = 5) [19, 47, 52, 99], neutropenia (n = 5) [39, 64, 73, 76], low serum immunoglobulin G level (n = 4) [19, 52, 108], low haemoglobin (n = 4) [30, 48, 112], high D-dimer (n = 3) [73], hypogammaglobulinemia (n = 2) [56, 92], lymphopenia (n = 2) [56, 76], anaemia (n = 2) [62, 112], low white blood cells (n = 2) [30, 64, 123], high lactate dehydrogenase (n = 2) [73], and high interleukin-6 (n = 2) [30, 73]. As expected, most prescribed therapeutic agents in these combined immunodeficiencies with associated or syndromic features cases were antibiotics (n = 37, 29.4%) [5, 19, 30, 34, 39, 46–48, 52, 63, 73, 74, 92, 95, 112], intravenous immunoglobulin (n = 32, 25.4%) [5, 19, 30, 34, 39, 40, 46, 48, 52, 65, 73, 74, 92, 95, 123], oxygen supplementation (n = 7, 5.5%) [19, 30, 48, 95, 124], and hydroxychloroquine (n = 5, 4%) [5, 30, 48, 117], however, treatment was not necessary in a considerable number of these combined immunodeficiencies with associated or syndromic features patients (n = 18, 14.3%) [46, 56, 63–65, 76, 92, 99, 108, 124]. There were combined immunodeficiencies with associated or syndromic features patients who were admitted to the intensive care units (n = 11, 8.7%) [19, 34, 40, 47, 48, 65, 95], intubated and placed on mechanical ventilation (n = 9, 7.1%) [34, 40, 47, 48, 65, 95] and suffered acute respiratory distress syndrome (n = 9, 7.1%) [34, 40, 47, 48, 65, 95]. Clinical outcomes of the combined immunodeficiencies with associated or syndromic features patients with mortality were documented in 7 (5.5%) [34, 40, 47, 48, 65], while 112 (88.9%) of the combined immunodeficiencies with associated or syndromic features cases recovered [5, 19, 30, 34, 39, 40, 46–48, 52, 56, 63–65, 73, 74, 76, 81, 92, 95, 97, 99, 108, 112, 117, 123, 124], final treatment outcome was not reported in few combined immunodeficiencies with associated or syndromic features patients (n = 6, 4.8%) [62, 125], and one case was still in the intensive care unit (n = 1, 0.8%) [95]. Mortality was COVID-19-related in two cases with combined immunodeficiencies with associated or syndromic features (2/126, 1.6%) [34, 65], however, COVID-19 was not attributable to death in four of the children with reported combined immunodeficiencies with associated or syndromic features (4/126, 3.2%) [34, 40, 47] and one study failed to report if COVID-19 was a leading or an underlying cause of death in one child with combined immunodeficiencies with associated or syndromic features (1/126, 0.8%) [48] (see Table 1).

### Cellular and humoral immunodeficiencies

Cellular and humoral immunodeficiencies were the third most-common IEIs in children who experienced COVID-19 (n = 102, 14.4%) [19, 31, 34, 40, 46, 48, 60, 62, 63, 65, 69, 73, 74, 81, 91, 92, 95, 98, 99, 104, 108, 111, 125, 126, 131] (see Additional file 2: Table S3). Among them, 60 have combined immunodeficiency (CID, 58.8% of all cellular and humoral immunodeficiencies) [19, 46, 48, 62, 63, 65, 73, 74, 91, 92, 95, 98, 99, 104, 111, 125], and 42 have severe combined immunodeficiency (SCID) (41.2%) [19, 31, 34, 40, 46, 48, 60, 63, 65, 69, 73, 74, 81, 98, 108, 125, 126, 131]. The most frequent main genetic causes of cellular and humoral immunodeficiencies in children infected with SARS-CoV-2 were DOCK8 deficiency (n = 6) [62, 125], IL2RG (n = 5) [46, 65, 108, 126], RelB deficiency (n = 3) [91, 92], JAK3 deficiency (n = 3) [31, 46, 131], and IL7Ra deficiency (n = 3) [19, 69, 73]. For patients with cellular and humoral immunodeficiencies who acquired SARS-CoV-2, the median interquartile range (IQR) age was 48 months [13.5 to 122], with a male predominance [n = 61, 59.8%] [19, 31, 40, 46, 48, 62, 63, 65, 73, 74, 92, 95, 98, 99, 108, 125, 126], and majority of the patients belonged to White (Caucasian) (n = 47, 46.1%) [46, 60, 62, 63, 73, 81, 95, 104, 108, 111, 125, 126, 131], Persian (n = 28, 27.4%) [19, 34, 48, 74, 98] and Hispanic (n = 18, 17.6%) [40, 65, 95, 125] ethnicity. In those cellular and humoral immunodeficiencies patients, few studies reported on specific allele changes (n = 8, 7.8%) [31, 60, 62, 69, 99, 111, 125, 126]. Reported modes of inheritance for the cellular and humoral immunodeficiencies in children were autosomal recessive (n = 61, 59.8%) [19, 31, 34, 40, 46, 48, 60, 62, 63, 65, 69, 73, 74, 81, 91, 92, 95, 98, 111, 125, 131], X-linked (n = 8, 7.8%) [19, 46, 65, 92, 99, 108, 126], or autosomal dominant (n = 1, 1%) [19], however, mode of inheritance in these cellular and humoral immunodeficiencies cases was unknown in a high percentage of patients (n = 32, 31.4%) [19, 46, 48, 63, 65, 73, 95, 98, 104]. COVID-19 in children with cellular and humoral immunodeficiencies was asymptomatic (16/102 = 15.7%) [63, 65, 73, 81, 92, 95, 99], mild (45/102 = 44.1%) [46, 60, 63, 65, 69, 73, 74, 81, 91, 92, 95, 98, 108, 111, 131], moderate (20/102 = 20%) [31, 34, 40, 46, 48, 63, 65, 73, 74, 81, 98, 126], severe (6/102 = 5.7%) [19, 48, 74, 104] or critical (6/102 = 5.7%) [19, 48, 73]. Most children with cellular and humoral immunodeficiencies did not get MIS-C due to COVID-19 (69/102, 67.6%) [19, 31, 34, 40, 46, 60, 63, 65, 73, 81, 92, 95, 98, 99, 108, 111, 126, 131], however, some children with cellular and humoral immunodeficiencies were reported to experience MIS-C (21/102, 20.6%) [48, 62, 69, 73, 81, 104, 125]. Few of those cellular and humoral immunodeficiencies cases presented with a previous known history of chronic lung disease (n = 7) [46, 63, 95, 98], chronic heart disease (n = 6) [63, 65, 73, 74, 95], epilepsy or seizures (n = 5) [34, 48, 63, 74, 98, 104], Down syndrome (n = 4) [65, 95], hypothyroidism (n = 4) [46, 48, 63, 104], post haematopoietic stem cell transplantation (n = 4) [46, 73, 95], lymphoma (n = 3) [46, 95], cognitive disability (n = 3) [63, 95], developmental delay (n = 3) [46, 63], hemophagocytic lymphohistiocytosis (n = 3) [95], adenitis due to tuberculosis vaccine (n = 3) [34, 48, 131], obesity (n = 3) [65], atrial or ventricular septal defects (n = 3) [34, 74, 104], neurological disorder (n = 2) [48, 74], hypogammaglobulinemia (n = 2) [92], colitis (n = 2) [63, 74], autoimmune haemolytic anaemia (n = 2) [46, 48], eczema (n = 2) [65, 95], hepatitis (n = 2) [63, 126] or dilated cardiomyopathy (n = 2) [34, 74]. Patients who suffered cellular and humoral immunodeficiencies and experienced COVID-19 were maybe more likely to have lymphopenia (n = 11) [31, 48, 60, 81, 104, 126], high C-reactive protein (n = 8) [48, 73, 98], neutropenia (n = 7) [73, 95, 126], high erythrocyte sedimentation rate (n = 7) [48, 69], high D-dimer (n = 5) [73, 104, 126], low serum immunoglobulin A levels (n = 5) [19, 60, 69, 99], low serum immunoglobulin M levels (n = 4) [19, 69, 99], thrombocytopenia (n = 4) [48, 98], high lactate dehydrogenase (n = 4) [73, 104], low serum immunoglobulin G levels (n = 3) [19, 60, 99], raised liver enzymes (n = 3) [69, 104, 108], low white blood cells (n = 3) [48], hypoalbuminemia (n = 2) [69, 73], high ferritin (n = 2) [104, 126], elevated partial thromboplastin time (n = 2) [104, 126], and high interleukin-10 (n = 2) [104, 126]. As expected, most prescribed therapeutic agents in these cellular and humoral immunodeficiencies cases were antibiotics (n = 50, 49%) [19, 31, 34, 46, 48, 73, 74, 95, 98, 104, 111, 131], intravenous immunoglobulin (n = 47, 46.1%) [19, 31, 34, 40, 46, 48, 63, 65, 69, 73, 74, 92, 95, 98, 99, 126, 131], (n = 11, 10.8%), hydroxychloroquine or chloroquine (n = 9, 8.8%) [48, 74, 95, 104, 131], antifungals (n = 6, 5.9%) [31, 34, 73, 74, 95], and oxygen supplementation (n = 6, 5.9%) [31, 95, 104], however, treatment was not necessary in a considerable number of these cellular and humoral immunodeficiencies patients (n = 21, 20.6%) [46, 60, 63, 65, 73, 95, 108]. There were cellular and humoral immunodeficiencies patients who were admitted to the intensive care units (n = 27, 26.5%) [19, 31, 34, 48, 65, 73, 74, 95, 98, 104], intubated and placed on mechanical ventilation (n = 23, 22.5%) [19, 31, 34, 48, 73, 74, 95, 98, 104] and suffered acute respiratory distress syndrome (n = 23, 22.5%) [19, 31, 34, 48, 73, 74, 95, 98, 104]. Clinical outcomes of the cellular and humoral immunodeficiencies patients with mortality were documented in 19 (18.6%) [19, 34, 48, 73, 74, 98], while 72 (70.6%) of the cellular and humoral immunodeficiencies cases recovered [19, 31, 40, 46, 48, 60, 63, 65, 69, 73, 74, 81, 91, 92, 95, 98, 99, 104, 108, 111, 126, 131], and final treatment outcome was not reported in few cellular and humoral immunodeficiencies patients (n = 9, 8.8%) [62, 125], and two cases were still in the intensive care unit (n = 2, 2%) [95]. Mortality was COVID-19-related in twelve cases with cellular and humoral immunodeficiencies (12/102, 11.8%) [48, 73, 74, 98], however, COVID-19 was not attributable to death in three of the children with reported cellular and humoral immunodeficiencies (3/102, 2.9%) [34, 48, 98] and one study failed to report if COVID-19 was a leading or an underlying cause of death in four children with cellular and humoral immunodeficiencies (4/102, 3.9%) [19] (see Table 1).

### Immune dysregulatory diseases

Immune dysregulatory diseases were the fourth most-common IEIs in children who experienced COVID-19 (n = 95, 13.4%) [6, 9, 19, 29, 32, 35, 38, 40, 43, 44, 46, 48, 50, 52, 54, 57, 58, 62, 63, 65, 67, 72–74, 79, 81, 82, 84, 86, 88, 93–97, 99, 100, 102, 109, 110, 113, 115, 118, 121, 125, 130] (see Additional file 2: Table S3). Among them, 25 have familial hemophagocytic lymphohistiocytosis (26.3% of all immune dysregulatory diseases) [6, 19, 29, 48, 62, 65, 67, 72, 73, 79, 81, 82, 84, 88, 99, 110, 125], 19 have autoimmune polyendocrine syndromes type-1 (APS-1) (20%) [35, 38, 73, 86, 94, 102, 113], 9 have autoimmune lymphoproliferative syndrome (ALPS) (9.5%) [50, 81, 95, 97, 130], 6 have LRBA deficiency (6.3%) [57, 58, 115, 125], 5 have TPP2 deficiency (5.3%) [118, 121, 125], 4 have XLP1 (4.2%) [44, 54, 81, 109], 4 have XLP2 (4.2%) [43, 65, 95, 100], 2 have SOCS1 deficiency (2.1%) [9, 96], 2 have CTLA4 deficiency (2.1%) [32, 95], 2 have IL-10Ra deficiency (2.1%) [74, 125], 2 have BACH2 deficiency (2.1%) [19] and 2 have RLTPR deficiency (2.1%) [73]. The remaining 13 patients have NOTCH1 mutation (n = 1) [19]; ALPS-Caspase10 (n = 1) [19]; CD137 deficiency (n = 1) [73]; interleukin-37 deficiency (n = 1) [19]; IPEX syndrome (n = 1) [93]; prolidase deficiency (n = 1) [62]; PRKCD deficiency (n = 1) [95]; MAGT1 deficiency (n = 1) [99]; and unspecified immune dysregulatory disease (n = 5) [40, 46, 52, 63]. The most frequent main genetic causes of immune dysregulatory diseases in children infected with SARS-CoV-2 were AIRE (n = 19) [35, 38, 73, 86, 94, 102, 113], LRBA deficiency (n = 6) [57, 58, 115, 125], PRF1 (n = 6) [74, 125], TPP2 (n = 5) [118, 121, 125], LYST (n = 4) [65, 84, 99, 125], XIAP deficiency (n = 4) [43, 65, 95, 100], SH2D1A deficiency (n = 4) [44, 54, 81, 109], STXBP2 (n = 3) [19, 73, 125], UNC13D (n = 2) [19, 125], SOCS1 deficiency (n = 2) [9, 96], CTLA4 deficiency (n = 2) [32, 95], and IL10RA deficiency (n = 2) [74, 125]. For patients with immune dysregulatory diseases who acquired SARS-CoV-2, the median interquartile range (IQR) age was 108 months [60 to 168], with a male predominance [n = 55, 57.9%] [6, 9, 19, 32, 35, 40, 43, 44, 46, 50, 54, 57, 58, 62, 63, 65, 72, 73, 79, 82, 88, 93–95, 99, 100, 102, 109, 113, 115, 125, 130], and majority of the patients belonged to White (Caucasian) (n = 53, 55.8%) [6, 9, 32, 35, 38, 43, 46, 50, 52, 62, 63, 67, 73, 81, 82, 84, 86, 94, 95, 97, 100, 109, 110, 113, 115, 118], Black (n = 12, 12.6%) [79, 93, 125] and Persian (n = 10, 10.5%) [19, 48, 54, 74, 88] ethnicity. In those immune dysregulatory diseases patients, few studies reported on specific allele changes (n = 15, 15.8%) [32, 35, 38, 58, 62, 84, 86, 94, 96, 99, 100, 109, 113, 121, 125]. Reported modes of inheritance for the immune dysregulatory diseases in children were autosomal recessive (n = 64, 67.4%) [6, 19, 29, 35, 38, 48, 57, 58, 62, 65, 67, 72–74, 79, 81, 82, 84, 86, 88, 94, 95, 99, 102, 110, 113, 115, 118, 121, 125], X-linked (n = 10, 10.5%) [43, 44, 54, 65, 81, 93, 95, 99, 100, 109], or autosomal dominant (n = 7, 7.4%) [9, 19, 32, 35, 95, 96], however, mode of inheritance in these immune dysregulatory diseases cases was unknown in a high percentage of patients (n = 14, 14.7%) [40, 46, 50, 52, 63, 81, 95, 97, 130]. COVID-19 in children with immune dysregulatory diseases was asymptomatic (11/95 = 11.6%) [32, 38, 58, 63, 65, 73, 74, 81, 95, 99], mild (34/95 = 35.8%) [6, 35, 43, 50, 52, 63, 65, 67, 72, 73, 81, 82, 88, 94–97, 99, 100, 110, 115, 121, 130], moderate (11/95 = 11.6%) [40, 46, 54, 73, 84, 95, 109, 133], severe (19/95 = 20%) [9, 19, 29, 44, 48, 57, 79, 86, 93, 102, 113, 133] or critical (2/95 = 2.1%) [19, 65]. Most children with immune dysregulatory diseases did not get MIS-C due to COVID-19 (71/95, 74.7%) [6, 9, 19, 32, 35, 38, 40, 43, 44, 46, 50, 52, 54, 57, 58, 63, 65, 67, 72–74, 79, 81, 82, 84, 86, 88, 93–97, 99, 100, 102, 109, 110, 113, 121, 130], however, some children with immune dysregulatory diseases were reported to experience MIS-C (23/95, 24.2%) [29, 48, 62, 73, 115, 125]. Few of those immune dysregulatory diseases cases presented with a previous known history of hypoparathyroidism (n = 13) [35, 38, 86, 94, 102], adrenal insufficiency (n = 12) [35, 67, 94, 102], cutaneous mucocutaneous candidiasis (n = 11) [35, 38, 94], inflammatory bowel disease (n = 8) [32, 58, 63, 65, 73, 74, 100, 115], arthritis (n = 6) [32, 58, 96, 109, 115], post hematopoietic stem cell transplants (n = 6) [54, 73, 93, 95, 100], grafts rejection (stem cell, gut or heart) (n = 6) [88, 93, 95, 100], hemophagocytic lymphohistiocytosis (n = 5) [62, 73, 95, 109], coagulopathy (n = 5) [9, 46, 82, 88, 109], sepsis (n = 5) [6, 29, 82, 95, 110], autoimmune haemolytic anaemia (n = 4) [9, 40, 48, 58], hepatitis (n = 4) [35, 102], hypogonadism (n = 4) [35, 38, 94], hypertension (n = 4) [65, 73, 79, 102], diabetes mellitus type 1 (n = 3) [32, 63, 102], hypothyroidism (n = 3) [35, 58, 113], immune thrombocytopenic purpura (n = 3) [9, 58, 121], chronic lung disease (n = 3) [58, 95, 110], asthma (n = 3) [35, 113, 121], vitiligo (n = 3) [35, 38, 86], organ failure (heart, liver and respiratory system) (n = 3) [88, 109, 110], gastrointestinal or rectal bleeding (n = 2) [35, 58], ascites (n = 2) [67, 109] or jaundice (n = 2) [82, 95]. Patients who suffered immune dysregulatory diseases and experienced COVID-19 were maybe more likely to have high C-reactive protein (n = 19) [6, 9, 35, 57, 67, 72, 73, 79, 88, 93, 113, 115], thrombocytopenia (n = 15) [6, 9, 48, 58, 72, 79, 82, 84, 88, 96, 100, 109, 115, 121, 130], high D-dimer (n = 14) [6, 35, 57, 72, 73, 82, 88, 93, 109, 113], high ferritin (n = 14) [6, 29, 35, 67, 72, 73, 79, 82, 84, 88, 93, 109, 115], raised liver enzymes (n = 14) [6, 35, 38, 57, 67, 72, 79, 82, 95, 102, 109, 113], high lactate dehydrogenase (n = 12) [9, 35, 57, 73, 88, 93, 109], low haemoglobin (n = 12) [9, 48, 67, 72, 79, 82, 84, 88, 102, 109, 115, 130], lymphopenia (n = 11) [9, 32, 35, 43, 57, 58, 88, 113], low serum immunoglobulin A level (n = 8) [9, 19, 44, 58], low serum immunoglobulin G level (n = 8) [9, 19, 44, 58], high interleukin-6 (n = 7) [29, 35, 43, 72, 73, 79, 115], leukopenia (n = 7) [9, 48, 72, 73, 130], anaemia (n = 7) [58, 82, 84, 88, 95, 109], high erythrocyte sedimentation rate (n = 6) [67, 73, 88, 109, 115], raised procalcitonin (n = 6) [9, 67, 73, 79, 93], high triglycerides (n = 6) [6, 67, 79, 84, 88, 109], high fibrinogen (n = 5) [6, 57, 72, 73, 79, 82, 84, 109], low serum immunoglobulin M level (n = 5) [19, 44, 58], low natural killer cells (n = 4) [44, 67, 84, 96], and high NT-proBNP (n = 3) [29, 88, 115]. As expected, most prescribed therapeutic agents in these immune dysregulatory diseases cases were steroids (n = 36, 37.9%) [9, 19, 29, 32, 35, 40, 43, 44, 54, 57, 67, 72, 74, 79, 82, 84, 86, 88, 95, 96, 100, 102, 109, 110, 113, 115, 121], antibiotics (n = 33, 34.7%) [6, 19, 29, 35, 44, 48, 54, 57, 58, 67, 72–74, 79, 82, 88, 95, 102, 109, 110, 113], intravenous immunoglobulin (n = 27, 28.4%) [6, 9, 19, 35, 40, 43, 44, 52, 57, 58, 65, 73, 79, 82, 86, 88, 95, 96, 100, 109, 110, 115, 121], hydroxychloroquine or chloroquine (n = 7, 7.4%) [48, 52, 54, 95, 109], oxygen supplementation (n = 6, 6.3%) [57, 79, 102, 109, 113], total parenteral nutrition (n = 6, 6.3%) [19], convalescent plasma (n = 6, 6.3%) [19, 35, 93, 113], tocilizumab (n = 6, 6.3%) [29, 35, 93, 95, 109], remdesivir (n = 5, 5.3%) [43, 79, 93, 102, 109], heparin (n = 5, 5.3%) [88, 102, 113], rituximab (n = 5, 5.3%) [43, 58, 88, 96, 109], antifungals (n = 5, 5.3%) [35, 54, 57, 73], and anakinra (n = 5, 5.3%) [6, 29, 43, 109, 115], however, treatment was not necessary in a considerable number of these immune dysregulatory diseases patients (n = 12, 12.6%) [38, 50, 63, 65, 94, 99, 130]. There were immune dysregulatory diseases patients who were admitted to the intensive care units (n = 34, 35.8%) [19, 29, 35, 44, 46, 48, 54, 57, 58, 65, 67, 72, 73, 79, 82, 84, 86, 88, 93, 95, 102, 109, 110, 113], intubated and placed on mechanical ventilation (n = 25, 26.3%) [19, 29, 35, 44, 46, 48, 54, 57, 58, 65, 67, 73, 79, 82, 86, 88, 93, 95, 109, 110, 113] and suffered acute respiratory distress syndrome (n = 27, 28.4%) [19, 29, 35, 44, 46, 48, 54, 57, 58, 65, 73, 79, 82, 86, 88, 93, 95, 109, 110, 113]. Clinical outcomes of the immune dysregulatory diseases patients with mortality were documented in 17 (17.9%) [19, 29, 44, 46, 48, 54, 58, 65, 73, 82, 88, 93, 95, 109, 110], while 60 (63.1%) of the immune dysregulatory diseases cases recovered [6, 9, 19, 32, 35, 38, 40, 43, 50, 52, 57, 63, 65, 67, 73, 74, 79, 81, 84, 86, 94–97, 99, 100, 102, 113, 115, 118, 121, 130] and final treatment outcome was not reported in many immune dysregulatory diseases patients (n = 18, 18.9%) [62, 72, 125]. Mortality was COVID-19-related in six cases with immune dysregulatory diseases (6/95, 6.3%) [29, 44, 48, 65, 73], however, COVID-19 was not attributable to death in ten of the children with reported immune dysregulatory diseases (10/95, 10.5%) [46, 54, 58, 73, 82, 88, 93, 95, 109, 110] and one study failed to report if COVID-19 was a leading or an underlying cause of death in one child with immune dysregulatory diseases (1/95, 1%) [19] (see Table 1).

### Autoinflammatory diseases

Autoinflammatory diseases were the fifth most-common IEIs in children who experienced COVID-19 (n = 67, 9.4%) [7, 19, 40, 48, 62, 65, 70, 73, 75, 81, 95, 97, 103, 115, 118, 119, 125, 128] (see Additional file 2: Table S3). Among them, 36 have familial Mediterranean fever (53.7% of all autoinflammatory diseases) [7, 62, 65, 70, 75, 115, 128], 4 have Blau syndrome (6%) [62, 125], 3 have Aicardi-Goutières syndrome (4.5%) [97, 103, 118], 3 have familial cold autoinflammatory syndromes 1 (4.5%) [65, 128], 2 have ADA2 deficiency (3%) [73, 125], 2 have NLRP1 deficiency (3%) [19, 62], 2 have TNF receptor-associated periodic syndrome (%) [81, 125], 2 have hyperpigmentation hypertrichosis, histiocytosis-lymphadenopathy plus syndrome SLC29A3 mutation (3%) [119, 125], and 2 have RNASEH2B deficiency (3%) [95]. The remaining 11 patients have familial cold autoinflammatory syndrome 4 (n = 1) [81]; deficiency of the interleukin 1 receptor antagonist (n = 1) [48]; pyogenic sterile arthritis, pyoderma gangrenosum, acne (PAPA) syndrome, hyperzincemia and hypercalprotectinemia (n = 1) [62]; mevalonate kinase deficiency (n = 1) [81]; SAMHD1 deficiency (n = 1) [95]; A20 deficiency (n = 1) [118]; Majeed syndrome (n = 1) [125]; STING-like disease (n = 1) [125]; CARD14 mediated psoriasis (n = 1) [125]; and unspecified autoinflammatory diseases (n = 2) [19, 40]. The most frequent main genetic causes of autoinflammatory diseases in children infected with SARS-CoV-2 were MEFV (n = 36) [7, 62, 65, 70, 75, 115, 128], NOD2 (n = 4) [62, 125], NLRP3 (n = 3) [65, 128], ADA2 deficiency (n = 2) [73, 125], NLRP1 deficiency (n = 2) [19, 62], TNFRSF1A (n = 2) [81, 125], and SLC29A3 (n = 2) [119, 125]. For patients with autoinflammatory diseases who acquired SARS-CoV-2, the median interquartile range (IQR) age was 108 months [78 to 168], with a male predominance [n = 32, 47.8%] [7, 19, 40, 62, 65, 70, 75, 95, 115, 125, 128], and majority of the patients belonged to White (Caucasian) (n = 50, 74.6%) [7, 62, 70, 73, 75, 81, 95, 97, 115, 118, 128], Hispanic (n = 6, 8.9%) [40, 65, 95, 125] and Black (n = 5, 7.5%) [125] ethnicity. In those autoinflammatory diseases patients, few studies reported on specific allele changes (n = 4, 6%) [62, 103, 125, 128]. Reported modes of inheritance for the autoinflammatory diseases in children were autosomal recessive (n = 50, 74.6%) [7, 19, 48, 62, 65, 70, 73, 75, 81, 95, 103, 115, 119, 125, 128], or autosomal dominant (n = 13, 19.4%) [62, 65, 81, 118, 125, 128], however, mode of inheritance in these autoinflammatory diseases cases was unknown in a few percentage of patients (n = 4, 6%) [19, 40, 97, 118]. COVID-19 in children with autoinflammatory diseases was asymptomatic (12/67 = 17.9%) [7, 65, 70, 75, 95, 103], mild (35/67 = 52.2%) [40, 65, 73, 75, 81, 97, 115, 119, 128], moderate (2/67 = 3%) [81, 115] or severe (3/67 = 4.5%) [19, 48]. Most children with autoinflammatory diseases did not get MIS-C due to COVID-19 (47/67, 70.1%) [7, 19, 40, 65, 70, 75, 81, 95, 97, 103, 119, 128], however, some children with autoinflammatory diseases were reported to experience MIS-C (18/67, 26.9%) [48, 62, 73, 115, 125]. Few of those autoinflammatory diseases cases presented with a previous known history of mental disability (n = 3) [95], epilepsy (n = 3) [75, 95], arthralgia or arthritis (n = 2) [128], cryopyrin-associated periodic syndrome (n = 2) [128] or asthma (n = 2) [75]. Patients who suffered autoinflammatory diseases and experienced COVID-19 were maybe more likely to have leukocytosis (n = 19) [75, 115], high erythrocyte sedimentation rate (n = 17) [48, 75, 115], high C-reactive protein (n = 17) [48, 73, 75, 115, 119, 128], high D-dimer (n = 6) [73, 103, 115, 119], high ferritin (n = 5) [73, 115, 119], low haemoglobin (n = 5) [48, 115, 119], high interleukin-6 (n = 4) [115, 119], thrombocytopenia (n = 2) [115, 119], high NT-proBNP (n = 2) [115], raised liver enzymes (n = 2) [103, 119], high lactate dehydrogenase (n = 2) [73, 103], neutropenia (n = 2) [73, 119], high fibrinogen (n = 2) [73, 119], and anaemia (n = 2) [48, 119]. As expected, most prescribed therapeutic agents in these autoinflammatory diseases cases were favipiravir (n = 12) [73, 75], hydroxychloroquine or chloroquine (n = 5) [7, 48, 75, 95], antibiotics (n = 8) [7, 19, 48, 73, 75, 119], steroids (n = 7) [19, 95, 103, 115, 119], intravenous immunoglobulin (n = 6) [40, 73, 115, 119], anakinra (n = 5) [115, 128], and colchicine (n = 3) [70, 128], however, treatment was not necessary in a few number of these autoinflammatory diseases patients (n = 4, 6%) [65, 95]. There were autoinflammatory diseases patients who were admitted to the intensive care units (n = 3, 4.5%) [19, 48], intubated and placed on mechanical ventilation (n = 2, 3%) [40, 48] and suffered acute respiratory distress syndrome (n = 2, 3%) [40, 48]. Clinical outcomes of the autoinflammatory diseases patients with mortality were documented in 2 (3%) [40, 48], while 52 (77.6%) of the autoinflammatory diseases cases recovered [7, 19, 65, 70, 73, 75, 81, 95, 97, 103, 115, 118, 119, 128] and final treatment outcome was not reported in many autoinflammatory diseases patients (n = 13, 19.4%) [48, 65]. Mortality was COVID-19-related in one case with autoinflammatory diseases (1/67, 1.5%) [48] and one study failed to report if COVID-19 was a leading or an underlying cause of death in one child with autoinflammatory diseases (1/67, 1.5%) [40] (see Table 1).

### Phagocytic diseases

Phagocytic diseases were the sixth most-common IEIs in children who experienced COVID-19 (n = 54, 7.6%) [19, 40, 43, 46, 48, 52, 53, 61–63, 65, 74, 77, 81, 90, 92, 95, 98, 101, 107, 117, 124, 127] (see Additional file 2: Table S3). Among them, 26 have chronic granulomatous disease (48.1% of all phagocytic diseases) [40, 43, 46, 48, 53, 62, 65, 74, 81, 90, 92, 95, 98, 124], 8 have Shwachman-Diamond syndromes (14.8%) [61, 95, 101], 6 have HAX1 deficiencies (11.1%) [74, 77, 124], 2 have Glycogen storage diseases type 1b (3.7%) [62], and 2 have Elastase deficiency (3.7%) [81, 127]. The remaining 10 patients have JAGN1 deficiency (n = 1) [117]; poikiloderma with neutropenia (n = 1) [107]; cystic fibrosis (n = 1) [19]; leukocyte adhesion deficiency type 3 (n = 1) [65]; GATA2 deficiency (n = 1) [95]; undefined leukopenia (n = 1) [46]; and unspecified phagocytic diseases (n = 4) [52, 63, 101, 124]. The most frequent main genetic causes of phagocytic diseases in children infected with SARS-CoV-2 were CYBB (n = 9) [40, 43, 46, 90, 92, 95], HAX1 deficiency (n = 6) [74, 77, 124], SDBS deficiency (n = 5) [61, 101], NCF1 (n = 3) [62, 92], ELANE (n = 2) [81, 127], and SLC37A4 (n = 2) [62]. For patients with phagocytic diseases who acquired SARS-CoV-2, the median interquartile range (IQR) age was 96 months [22.5 to 180], with a male predominance [n = 26, 48.1%] [19, 40, 43, 46, 48, 52, 53, 62, 65, 74, 90, 92, 95, 98, 101, 107, 127], and majority of the patients belonged to White (Caucasian) (n = 28, 51.8%) [43, 46, 52, 61–63, 81, 95, 107, 124], Persian (n = 11, 20.4%) [19, 48, 53, 74, 77, 98, 117] and Hispanic (n = 9, 16.7%) [40, 65, 95] ethnicity. In those phagocytic diseases patients, few studies reported on specific allele changes (n = 5, 9.2%) [62, 77, 107, 124, 127]. Reported modes of inheritance for the phagocytic diseases in children were autosomal recessive (n = 32, 59.2%) [19, 53, 61, 62, 65, 74, 77, 81, 92, 101, 107, 117, 124], X-linked (n = 12, 22.2%) [40, 43, 46, 48, 90, 92, 95] or autosomal dominant (n = 4, 7.4%) [81, 95, 101, 127], however, mode of inheritance in these phagocytic diseases cases was unknown in a few percentage of patients (n = 5, 9.2%) [46, 52, 63, 98, 124]. COVID-19 in children with phagocytic diseases was asymptomatic (7/54 = 13%) [65, 92, 95, 98, 124], mild (29/54 = 53.7%) [40, 43, 46, 48, 52, 61, 63, 65, 74, 77, 81, 90, 92, 95, 101, 107, 124], moderate (10/54 = %) [40, 48, 61, 65, 74, 81, 117, 127], severe (2/54 = 3.7%) [53] or critical (1/54 = 1.8%) [19]. Most children with phagocytic diseases did not get MIS-C due to COVID-19 (45/54, 83.3%) [19, 40, 43, 46, 48, 52, 53, 61, 63, 65, 74, 77, 81, 90, 92, 95, 98, 101, 107, 117, 124, 127], however, few children with phagocytic diseases were reported to experience MIS-C (7/54, 13%) [40, 48, 53, 62]. Few of those phagocytic diseases cases presented with a previous known history of sepsis or septic, cardiogenetic or compensated shock (n = 4) [40, 43, 95, 124], liver abscess or disorder (n = 3) [74, 98, 124], chronic lung disease (n = 3) [74, 95], post haematopoietic stem cell transplantation (n = 3) [90, 101], dermatitis (n = 2) [107, 124], immune thrombocytopenic purpura (n = 2) [92, 124], hypotension (n = 2) [40, 95] and Gaucher disease (n = 2) [74, 77]. Patients who suffered phagocytic diseases and experienced COVID-19 were maybe more likely to have lymphopenia (n = 5) [43, 77, 90, 101], high C-reactive protein (n = 5) [43, 53, 77, 90, 107], neutropenia (n = 4) [77, 101, 107, 127], high erythrocyte sedimentation rate (n = 4) [48, 77, 90], low haemoglobin (n = 3) [48, 53], high lactate dehydrogenase (n = 3) [53, 90], thrombocytopenia (n = 3) [40, 95], neutrophilia (n = 3) [43, 90, 107], anaemia (n = 3) [53, 95], thrombocytosis (n = 2) [48, 53], elevated prothrombin time (n = 2) [53] and high ferritin (n = 2) [53, 107]. As expected, most prescribed therapeutic agents in these phagocytic diseases cases were antibiotics (n = 27) [43, 46, 48, 52, 53, 74, 77, 90, 92, 95, 98, 101, 107, 117, 124, 127], antifungals (n = 9) [46, 53, 74, 92, 101], steroids (n = 7) [40, 43, 53, 61, 74, 95], intravenous immunoglobulin (n = 6) [19, 40, 43, 53, 95], oxygen supplementation (n = 4) [48, 53, 127], acyclovir (n = 4) [98, 101, 124], hydroxychloroquine (n = 3) [48, 77, 117], and granulocyte colony-stimulating factor (n = 3) [52, 77, 127], however, treatment was not necessary in a few number of these phagocytic diseases patients (n = 8, 14.8%) [63, 65, 124]. There were phagocytic diseases patients who were admitted to the intensive care units (n = 4, 7.4%) [19, 40, 90, 95], intubated and placed on mechanical ventilation (n = 3, 5.5%) [19, 40, 95] and suffered acute respiratory distress syndrome (n = 5, 9.2%) [19, 40, 53, 95]. Clinical outcomes of the phagocytic diseases patients with mortality were documented in 3 (5.5%) [19, 40, 95], while 47 (87%) of the autoinflammatory diseases cases recovered [40, 43, 46, 48, 52, 53, 61, 63, 65, 74, 77, 81, 90, 92, 95, 98, 101, 107, 117, 124, 127] and final treatment outcome was not reported in four phagocytic diseases patients (n = 4, 7.4%) [62]. COVID-19 was not attributable to death in two of the children with reported phagocytic diseases (2/54, 3.7%) [40, 95] and one study failed to report if COVID-19 was a leading or an underlying cause of death in one child with phagocytic diseases (1/54, 1.8%) [19] (see Table 1).

### Innate immunodeficiencies

Innate immunodeficiencies were the seventh most-common IEIs in children who experienced COVID-19 (n = 50, 7%) [4, 10, 19, 25, 26, 33, 37, 50–52, 63, 65, 68, 71, 73, 78, 81, 87, 89, 95, 97, 98, 106, 108, 114, 125, 132] (see Additional file 2: Table S3). Among them, 8 have TLR7 deficiency (16% of all innate immunodeficiencies) [19, 26, 33, 106, 132], 7 have MyD88 deficiency (14%) [37, 50, 63, 89, 97], 7 have STAT1 deficiency (14%) [52, 65, 68, 73, 95, 125], 3 have IFNAR1 deficiency (6%) [19, 25, 78], 3 have IFNAR2 deficiency (6%) [51], 3 have TYK2 deficiency (6%) [132], 2 have TBK1 deficiency (4%) [10, 114], 2 have isolated congenital asplenia (4%) [81, 108], 2 have IFN-g receptor 2 deficiency (4%) [71, 95], and 2 have MDA5 deficiency (4%) [125]. The remaining 11 patients have TRIF deficiency (n = 1) [19]; warts, hypogammaglobulinemia, infections, myelokathexis syndrome (n = 1) [65]; IRAK4 deficiency (n = 1) [65]; IRF9 deficiency (n = 1) [87]; STAT2 deficiency (n = 1) [132]; interleukin-12 and interleukin-23 receptor b1 chain deficiency (n = 1) [4]; and unspecified innate immunodeficiencies (n = 5) [19, 65, 97, 98]. The most frequent main genetic causes of innate immunodeficiencies in children infected with SARS-CoV-2 were TLR7 deficiency (n = 8) [19, 26, 33, 106, 132], MYD88 (n = 7) [37, 50, 63, 89, 97], STAT1-GOF (n = 6) [52, 65, 68, 73, 95, 125], IFNAR1 deficiency (n = 3) [19, 25, 78], IFNAR2 deficiency (n = 3) [51], TYK2 deficiency (n = 3) [132], TBK1 deficiency (n = 2) [10, 114], IFNGR2 (n = 2) [51], and IFIH1 (n = 2) [125]. For patients with innate immunodeficiencies who acquired SARS-CoV-2, the median interquartile range (IQR) age was 96 months [48 to 153], with a male predominance [n = 23, 46%] [10, 19, 26, 33, 37, 51, 52, 63, 65, 68, 71, 73, 78, 89, 95, 106, 132], and majority of the patients belonged to White (Caucasian) (n = 26, 52%) [10, 33, 37, 50–52, 63, 68, 71, 73, 81, 89, 95, 97, 108, 114, 132], Persian (n = 9, 18%) [19, 25, 26, 33, 78, 98] and Hispanic (n = 8, 16%) [4, 65, 95, 106, 125] ethnicity. In those innate immunodeficiencies patients, few studies reported on specific allele changes (n = 14, 28%) [4, 10, 25, 26, 33, 37, 51, 68, 78, 87, 106, 114, 125, 132]. Reported modes of inheritance for the innate immunodeficiencies in children were autosomal recessive (n = 27, 54%) [4, 10, 19, 25, 37, 50, 51, 63, 71, 73, 78, 87, 89, 95, 97, 106, 114, 125, 132], X-linked (n = 7, 14%) [19, 26, 33, 132] or autosomal dominant (n = 9, 18%) [19, 52, 65, 68, 95, 125], however, mode of inheritance in these innate immunodeficiencies cases was unknown in a few percentage of patients (n = 7, 14%) [19, 65, 81, 97, 98, 108]. COVID-19 in children with innate immunodeficiencies was asymptomatic (3/50 = 6%) [63, 65, 95], mild (13/50 = 26%) [4, 51, 52, 68, 71, 87, 95, 97, 98, 106, 108], moderate (8/50 = 16%) [50, 51, 65, 73, 81, 89, 132], severe (16/50 = 32%) [10, 19, 33, 37, 51, 65, 78, 89, 114, 132] or critical (6/50 = 12%) [19, 25, 26, 132]. Most children with innate immunodeficiencies did not get MIS-C due to COVID-19 (41/50, 82%) [4, 10, 19, 33, 37, 50–52, 63, 65, 68, 71, 78, 81, 87, 89, 95, 97, 98, 106, 108, 132], however, few children with innate immunodeficiencies were reported to experience MIS-C (8/50, 16%) [19, 25, 65, 73, 114, 125]. Few of those innate immunodeficiencies cases presented with a previous known history of autoimmune haemolytic anaemia (n = 4) [52, 73, 95, 132], epilepsy or seizures (n = 4) [26, 33, 52, 114], renal, heart or multi-organ failure (n = 4) [26, 33, 114], respiratory failure (n = 3) [51], hypertension (n = 2) [26, 33], hepatitis (n = 2) [52, 106], diarrhoea (n = 2) [65, 106], sepsis (n = 2) [132], asthma (n = 2) [51, 106], bradycardia (n = 2) [25, 78] or Kawasaki disease (n = 2) [132]. Patients who suffered innate immunodeficiencies and experienced COVID-19 were maybe more likely to have lymphopenia (n = 10) [26, 33, 37, 50, 52, 68, 89, 108], high C-reactive protein (n = 9) [25, 33, 37, 50, 68, 73, 87, 106], low IgA level (n = 5) [19, 26, 37, 52], anaemia (n = 4) [25, 26, 33, 50], thrombocytopenia (n = 4) [26, 33, 37, 50], high ESR (n = 3) [25, 26, 78], low serum immunoglobulin M level (n = 3) [19, 52], low serum immunoglobulin G level (n = 3) [19, 26], low haemoglobin (n = 3) [26, 33, 78], high D-dimer (n = 3) [50, 73, 106], high white blood cells (n = 3) [37, 78, 114], elevated partial thromboplastin time (n = 2) [78, 106], neutrophilia (n = 2) [37, 71], high ferritin (n = 2) [50, 73], leukocytosis (n = 2) [25, 95], raised liver enzymes (n = 2) [25, 114], metabolic acidosis (n = 2) [25, 114], high interleukin-6 (n = 2) [68, 78], and low memory B cells (n = 2) [52, 68]. As expected, most prescribed pharmacotherapy agents in these innate immunodeficiencies cases were antibiotics (n = 17) [19, 25, 33, 37, 50–52, 63, 68, 73, 78, 98], intravenous immunoglobulin (n = 12) [19, 25, 37, 52, 63, 65, 68, 73, 78], steroids (n = 11) [19, 25, 50, 51, 71, 78, 95, 114], oxygen supplementation (n = 10) [19, 33, 37, 51, 78, 89], antiplatelets (n = 6) [19, 25, 78], chloroquine or hydroxychloroquine (n = 5) [33, 89], remdesivir (n = 4) [51, 78, 89], favipiravir (n = 3) [33, 52, 78], angiotensin-converting enzyme inhibitors (n = 3) [19, 25, 33], total parenteral nutrition (n = 3) [19, 26], and biological agents (n = 3) [19], however, treatment was not necessary in a few number of these innate immunodeficiencies patients (n = 4, 8%) [65, 95, 108]. There were innate immunodeficiencies patients who were admitted to the intensive care units (n = 19, 38%) [19, 25, 26, 33, 51, 71, 78, 89, 114, 132], intubated and placed on mechanical ventilation (n = 14, 28%) [19, 25, 26, 33, 51, 71, 89, 114] and suffered acute respiratory distress syndrome (n = 17, 34%) [19, 25, 26, 33, 51, 71, 78, 89, 114, 132]. Clinical outcomes of the innate immunodeficiencies patients with mortality were documented in 5 (10%) [19, 25, 51, 114], while 42 (84%) of the innate immunodeficiencies cases recovered [4, 10, 19, 26, 33, 37, 50–52, 63, 65, 68, 71, 73, 78, 81, 87, 89, 95, 97, 98, 106, 108, 132] and final treatment outcome was not reported in three innate immunodeficiencies patients (n = 3, 6%) [125]. Mortality was COVID-19-related in four cases with innate immunodeficiencies (4/50, 8%) [19, 25, 51, 114] and COVID-19 was not attributable to death in one of the children with reported innate immunodeficiencies (1/50, 2%) (see Table 1).

### Complement deficiencies

Complement deficiencies were the eighth most-common IEIs in children who experienced COVID-19 (n = 11, 1.5%) [19, 62, 65] (see Additional file 2: Table S3). Among them, 3 have factor H deficiency (27.3% of all complement deficiencies) [19, 62], 3 have C1 inhibitor deficiency (27.3%) [65], and 2 have ficolin 3 deficiency (18.2%) [62]. The remaining 3 patients have factor H –related protein deficiency (n = 1) [19]; factor I deficiency (n = 1) [62]; and C3 deficiency (n = 1) [65]. The most frequent main genetic causes of complement deficiencies in children infected with SARS-CoV-2 were CFH (n = 3) [19, 62], SERPING1 (n = 3) [65], and FCN3 (n = 2) [62]. For patients with complement deficiencies who acquired SARS-CoV-2, the median interquartile range (IQR) age was 168 months [72 to 180], with a male predominance [n = 5, 45.4%] [19, 62, 65], and majority of the patients belonged to White (Caucasian) (n = 4, 36.4%) [62], Hispanic (n = 4, 36.4%) [65] and Persian (n = 3, 27.3%) [19] ethnicity. Reported modes of inheritance for the complement deficiencies in children were autosomal recessive (n = 7, 63.6%) [19, 62, 65] or autosomal dominant (n = 4, 36.4%) [19, 65]. COVID-19 in children with complement deficiencies was asymptomatic (1/11 = 9.1%) [65], mild (3/11 = 27.3%) [65], severe (2/11 = 18.2%) [19] or critical (1/11 = 9.1%) [19]. Four children with complement deficiencies did not get MIS-C due to COVID-19 (4/11, 36.4%) [65], however, four children with complement deficiencies were reported to experience MIS-C (4/11, 36.4%) [19, 62]. Few of those complement deficiencies cases presented with a previous known history of hereditary angioedema (n = 3) [65]. Patients who suffered complement deficiencies and experienced COVID-19 were maybe more likely to have low serum immunoglobulin A, immunoglobulin M and immunoglobulin G levels [19], however, laboratory findings were not reported in most complement deficiencies patients (n = 10) [19, 62, 65]. As expected, most prescribed therapeutic agents in these complement deficiencies cases were antibiotics (n = 3) [19] and total parenteral nutrition (n = 3) [19]. There were complement deficiencies patients who were admitted to the intensive care units (n = 3, 27.3%) [19], intubated and placed on mechanical ventilation (n = 1, 9.1%) [19] and suffered acute respiratory distress syndrome (n = 1, 9.1%) [19]. Among these complement deficiencies patients, one patient died (9.1%) [19] and six patients survived (54.5%) [19, 65]. Mortality was COVID-19-related in one case with complement deficiencies (1/11, 9.1%) [19] (see Table 1).

### Bone marrow failure

Bone marrow failure was the ninth most-common IEIs in children who experienced COVID-19 (n = 7, 1%) [19, 41, 54, 59, 74, 101, 116] (see Additional file 2: Table S3). Among them, 5 have Fanconi anaemia (71.4% of all bone marrow failure) [41, 54, 59, 74, 116], 1 has SAMD9 deficiency (14.3%) [101], and 1 has DKCA1 deficiency (14.3%) [19]. For patients with bone marrow failure who acquired SARS-CoV-2, the median interquartile range (IQR) age was 60 months [48 to 84], with a female predominance [n = 5, 71.4%] [19, 59, 74, 101, 116] except 1 patient was male (14.3%) [54], and majority of the patients belonged to Persian (n = 3, 42.8%) [19, 54, 74] and Indian (n = 2, 28.6%) [41, 116] ethnicity. Reported modes of inheritance for the bone marrow failure in children were autosomal recessive (n = 5, 71.4%) [41, 54, 59, 74, 116] or autosomal dominant (n = 2, 28.6%) [19, 101]. COVID-19 in children with bone marrow failure was asymptomatic (1/7 = 14.3%) [59], mild (2/7 = 28.6%) [41, 74], moderate (2/7 = 28.6%) [54, 101] or severe (1/7 = 14.3%) [19]. Almost all children with bone marrow failure did not get MIS-C due to COVID-19 (5/7, 71.4%) [19, 41, 54, 59, 101]. Few of those bone marrow failure cases presented with a previous known history of post haematopoietic stem cell transplantation (n = 2) [41, 54] and posterior reversible encephalopathy syndrome (n = 2) [41, 54]. The most prescribed therapeutic agents in children with bone marrow failure who suffered COVID-19 was the intravenous immunoglobulin (n = 2) [54, 74] and tacrolimus (n = 2) [41, 54]. There were bone marrow failure patients who were admitted to the intensive care units (n = 2, 28.6%) [19, 54], intubated and placed on mechanical ventilation (n = 1, 14.3%) [54] and suffered acute respiratory distress syndrome (n = 1, 14.3%) [54]. Among these bone marrow failure patients, one patient died (14.3%) [54] and six patients survived (85.7%) [19, 41, 59, 74, 101, 116]. COVID-19 was not attributable to death in one of the children with reported bone marrow failure (1/7, 14.3%) [54] (see Table 1).

### Phenocopies of primary immunodeficiencies

RAS-associated autoimmune leukoproliferative disease was reported in a 120 month-old white child following SARS-CoV-2 infection, with development of hypertelorism, secondary hemophagocytic lymphohistiocytosis and aplastic anaemia [124]. Patient never needed intensive care unit admission or mechanical ventilation, suffered no acute respiratory distress syndrome and survived without treatment (see Additional file 2: Table S3).

## Discussion

This systematic review included 710 children with IEIs with laboratory-confirmed COVID-19 from 116 observational studies to provide an insight into the clinical course and treatment outcomes in children with IEIs who were infected with SARS-CoV-2. To the best of our knowledge, this is the first and largest systematic review to report exclusively on development of SARS-CoV-2 infection in children with IEIs, in an attempt to avoid measurement bias. Of all the IEIs categories, we found predominantly antibody deficiencies were the most common IEIs (n = 197, 27.7%) and phenocopies of primary immunodeficiencies were the least common IEIs (n = 1, 0.1%) in children who experienced COVID-19, in line with findings of three previous systematic reviews [13, 14, 19], which reported that predominantly antibody deficiencies constituted the majority of IEIs and phenocopies of primary immunodeficiencies constituted the minority of IEIs in a mixed population with inborn errors of immunity and SARS-CoV-2 infection (mostly adults and few children). Our finding is also in parallel to the findings reported by Jeffrey Modell Centres Network registry in 2018 that found global rate of IEIs by category was highest for predominantly antibody deficiencies (n = 46,077) and lowest for phenocopies of primary immunodeficiencies (n = 114) [134].

We report identical ICU admission for children with IEIs infected with SARS-CoV-2 percentage to the rates reported in two previous systematic reviews (16%) [13, 14], and the fatality rate in our study (8.4%) was very similar to the rates reported in three reviews made in Iran (8.7%) [19], United States (9%) [14], and Belgium (9%) [13]. However, we report a much lower fatality rate in children with IEIs with COVID-19 than a previous review that included lower number of studies and fewer pediatric cases (8.4% vs 23.6%) [19]. Across the studies we included in our review, rates of ICU admission in children with IEIs with COVID-19 differ due to different healthcare systems, medical practice and admission criteria as well as differences in predisposing factors such as age, comorbidities and testing availability in the patients served. Moreover, there was a large variation in fatality rates in those children with IEIs infected with SARS-CoV-2, which could be explained by differences in child’s baseline characteristics and severity of IEIs illness and the result of a better clinical management of COVID-19. It is worth to mention that although most cases of COVID-19 in the pediatric population are mild or asymptomatic [135, 136], the overall rate of ICU admission and mortality rate we report in children with IEIs who were infected with SARS-CoV-2 suggests that the risk of severe disease and mortality from SARS-CoV-2 is much higher in children with IEIs compared to the general healthy children. For example, COVID-19-related ICU admission among healthy children was very low (141 per 20,458 (0.7%) children age ≤ 9 years and 216 per 49,245 (0.4%) children age 10 to 19 years) [137] and the pooled analysis from seven countries shown the COVID-19-related death rate among healthy children (age 0–19 years) was 0.17 per 100,000 population (0.48% of the estimated total mortality from all causes) [138].

In our review, COVID-19 in children with different IEIs patents resulted in a mild disease in more than 76% of all included cases. At the beginning of the COVID-19 pandemic, children with IEIs were thought to be at risk for severe COVID-19. Recent studies however report that most children with IEIs had asymptomatic infection with SARS-CoV-2 or mild COVID-19, as seen in the general population [46, 63, 65, 81, 94, 95, 97, 108, 124, 130]. However, some children with IEIs may experience more severe COVID-19 and the COVID-19-related mortality rates among children IEIs were higher than those of the general population [19, 25, 29, 44, 48, 51, 65, 73, 74, 98]. In this context, the highest ICU admission and fatality rates were observed in cases belonging to cellular and humoral immunodeficiencies (26.5% and 18.6%) and immune dysregulatory diseases (35.8% and 17.9%) groups, especially in children infected with SARS-CoV-2 who suffered severe combined immunodeficiency (28.6% and 23.8%), combined immunodeficiency (25% and 15%), familial hemophagocytic lymphohistiocytosis (40% and 20%), X-linked lymphoproliferative diseases-1 (75% and 75%) and X-linked lymphoproliferative diseases-2 (50% and 50%) compared to the other IEIs cases. Specific subset of IEIs entities, especially those that are younger and those with reduced type I interferon signalling, have been reported to experience severe COVID-19 [10, 95]. Type I interferons are critical to controlling certain viruses during the earliest stages of infections and type I interferons production plays an essential role in host defence against COVID-19 [139]. IEIs of TLR3-, IRF7-, UNC93B1-, TICAM1-, TBK1-, and interferon alpha and beta receptor subunit 1 (IFNAR1)-dependent type I interferon immunity have been described to underlie life-threatening COVID-19 pneumonia in patients with no prior severe infection [10, 133]. Therefore, defects in the pathways generating type I interferons, inhibition of type I interferon production, and autoantibodies that neutralize interferons predispose to severe COVID-19. Moreover, patients with XLP1 and XLP2 who have dysregulated T-cell activation were suggested to be at risk of severe COVID-19 infection because of adverse unregulated inflammatory responses [48]. And most of the children with IEIs who developed COVID-19 in the cellular and humoral immunodeficiencies and immune dysregulatory diseases groups were likely to have lymphopenia as well as low serum immunoglobulins which was previously described to correlate with risk of ICU admission and severe SARS-CoV-2 infection [83, 95].

We found male IEIs pediatric patients with COVID-19 were predominant among all major IEIs categories except the bone marrow failure group and COVID-19–related fatality was higher in male patients (71.7% of deceased patients). The large predominance of males among IEIs cases is certainly due to the presence of the severe X-linked IEIs, such as SCID due to IL2RG mutations, IPEX, WAS, hyper-IgM due to CD40L deficiency, nuclear factor κB essential modulator (NEMO), and most chronic granulomatous disease and agammaglobulinemia cases which tend to have a severe clinical phenotype with symptoms manifesting in the first year of life [140], and in agreement to the Jeffrey Modell Foundation global report and a systematic review that included 104,614 IEIs patients registered in 80 countries [134, 141]. We found development of COVID-19 in children with IEIs was highest in people of White (Caucasian), Persian and Hispanic ethnicity (62.5%, 14.1% and 13.1%, respectively). Besides, we found fatality rate in children with IEIs infected with SARS-CoV-2 was the highest in patients with Persian ethnicity (n = 32, 53.3%). Iran reported 3056 IEIs patients with updated data in 2018 to the European Society for Immunodeficiencies registry, thus describing the largest cohort of IEIs patients in Asia and the fifth-largest national cohort of IEIs patients globally [142]. Largest worldwide cohorts of patients with IEIs have been reported from European countries like France (n = 5426), Turkey (n = 6392), Spain (n = 2211), Germany (n = 1981), Italy (n = 1275), Poland (n = 690), Switzerland (n = 352), Greece (n = 202), and Sweden (n = 92) [143]. Moreover, from 2013 to 2021, total IEIs patients identified with a specific IEIs defect increased by 45.2% in Western Europe, 25.7% in Eastern Europe, and 92.4% in the Middle East [144]. Still, representation of children with IEIs with other ethnicities at risk to develop COVID-19 can be misleading as most studies we included in our review have been done with pediatric populations of a European and Persian background (study locations: Iran, Germany, Turkey, Spain, Italy, Switzerland and Greece), therefore, there is less information about the development and health outcomes of COVID-19 in children with IEIs in different races or ethnic groups.

We report the most common mode of inheritance for IEIs in children infected with SARS-CoV-2 was autosomal recessive (n = 369, 52%), a finding which can be explained by the high percentages of consanguineous marriages in the areas for the IEIs pediatric cases included in our review. Majority of genes are associated with autosomal recessive diseases and consanguinity increases the IEIs genetic diagnostic yield [145]. Globally, consanguineous marriages have been reported to be a very common practice in many of this review’s included study locations such as Saudi Arabia (75%) [146], Iran (60%) [141], Tunisia (58%) [147], India (38%) [148], France (15%) [149] and Mexico (11%) [150], and much higher compared to the global rate (6.1%) [141]. The relative higher fatality rate in children with IEIs and COVID-19 in this study may be related to the autosomal recessive mode of inheritance (n = 40) because children born to consanguineous parents have been reported to experience more severe forms of IEIs compared to other regions and increased rates of morbidity and mortality compared to other patients [151].

The fatality rate was potentially high in children who developed COVID-19 with PAX (2/2 cases), STIM1 (1/2 cases), PIGA (1/2 cases), UNC13D (1/2 cases), CARMIL2 (1/2 cases), TBK1 (1/2 cases), IFNAR1 (2/3 cases), IFNAR2 (2/3 cases), TBCE (2/3 cases), CFH (1/3 cases), IL7Ra (1/3 cases), TNFRSF13B (1/3 cases), SH2D1A (3/4 cases), XIAP (2/4 cases), DNMT3B (1/5 cases), LRBA (1/6 cases), WAS (1/14 cases), AIRE (1/19 cases), and BTK (2/41 cases) deficiencies, although this needs to be confirmed by evaluation of additional pediatric patients with these rare IEIs. However, the main genetic causes of IEIs were not identified in 269 (38%) of the children with IEIs with COVID-19 reported to date, and a high proportion of non-severe IEIs pediatric cases might not be reported or were not genetically evaluated, which is required to make a more accurate evaluation of the molecular defects underlying different types of COVID-19 severity in children with IEIs. Nevertheless, the varying number and level of details for reported SARS-CoV-2 infection in children with many IEIs types limit our ability to compare the severity of COVID-19 between different IEIs categories and subcategories.

Hence infectious diseases are the most common complication of IEIs and occur even when protective measures are taken [152], efforts to prevent infections like COVID-19 are critical for children especially those with severe forms IEIs, such as SCID, and important for patients of any age. In addition to IEIs targeted pharmacotherapy, biologic use, and consideration of curative therapies such as bone marrow transplantation or gene therapy [153, 154], early treatment with polyclonal or monoclonal anti–SARS-CoV-2 antibodies [139, 155, 156], treatment with antiviral medications [153, 154], vaccination prioritization [153, 154], and potentially including interferon and anti-inflammatory drugs [153, 154], might improve COVID-19 management, resulting in a lower mortality in children with IEIs.

## Limitations

We acknowledge that our study was not without some limitations. First, for case studies, the more severe cases with worse outcomes may be more likely to be published and children with IEIs who were diagnosed with COVID-19 and remained asymptomatic or had mild disease courses that did not require hospitalization were less likely to be included in the published literature. Therefore, treatment outcomes such as hospitalization, ICU admission, oxygen requirement, and death are likely overestimated. Second, the heterogeneity across the large number of IEIs conditions makes it challenging to draw overall conclusions about COVID-19 in children with IEIs. Third, the low number of cases in most IEIs major categories and subcategories could mean that the cases included in this review are not representative of those groups. Last, potential limitation is the exclusion of non-English articles. As a result, important findings for COVID-19 outcomes in IEIs pediatric cases may have been missed.

## Conclusion

Globally, predominantly antibody deficiencies were the most prevalent and phenocopies of primary immunodeficiencies were the least prevalent IEIs in children who developed COVID-19. Children with IEIs infected with SARS-CoV-2 may experience higher rates of ICU admission and mortality in comparison with the immunocompetent pediatric populations Underlying immune defects does seem to be independent risk factors for severe SARS-CoV-2 infection in children with IEIs, a number of children with SCID and CID were reported to have prolonged infections–though the number of patients is small–but especially immune dysregulation diseases (XLP1 and XLP2) and innate immunodeficiencies impairing type I interferon signalling (IFNAR1, IFNAR2 and TBK1). Efforts to prevent infections like COVID-19 are critical for children especially those with severe forms IEIs.

### Supplementary Information

**Additional file 1: Table S1.** Search strategy. **Table S2.** Inborn errors of immunity in each inborn error of immunity class.

**Additional file 2: Table S3.** Summary of the characteristics of the included studies with evidence on IEIs and COVID-19 in pediatric patients (n = 116 studies), 2020-2022.

## Data Availability

All data generated or analyzed during this study are included in this published article.

## Abbreviations

AD: Autosomal dominant
AR: Autosomal recessive
ARDS: Acute respiratory distress syndrome
CID: Combined immunodeficiency
COVID-19: Coronavirus disease 2019
CVID: Common variable immunodeficiency
HSCT: Hematopoietic stem cell transplant
IEIs: Inborn errors of immunity
IUIS: International union of immunologic societies
IVIG: Intravenous immunoglobulin
NOS: Newcastle–Ottawa scale
PRISMA: Preferred Reporting Items for systematic reviews and meta-Analyses
SARS-CoV-2: Severe acute respiratory syndrome coronavirus 2
SCID: Severe combined immunodeficiencies
SCIg: Subcutaneous immunoglobulin
XLP: X-linked lymphoproliferative disease
