# Supplementary material for: Severity of SARS-CoV-2 infection in children with inborn errors of immunity (primary immunodeficiencies): a systematic review
Source: Allergy Asthma Clin Immunol. 2023 Aug 9;19:69. doi: 10.1186/s13223-023-00831-1 (PMC10413516; doi:10.1186/s13223-023-00831-1)
Supplement: Supplementary file 1 — Additional file 1: Table S1. Search strategy. Table S2. Inborn errors of immunity in each inborn error of immunity class. [file 13223_2023_831_MOESM1_ESM.docx]

Table Supplementary 1: Search strategy

| Database | Strategy | Run Date | Records |
| --- | --- | --- | --- |
| ProQuest Central | [((((((11q23 OR 11q23del OR acd OR ace OR "angiotensin converting enzyme" OR acp5 OR "acid phosphate 5" OR actb OR ada OR ada2 OR adam17 OR tace OR "adenosine deaminase" OR adar1 OR aicda OR "AID enzyme" OR "activation induced cytidine deaminase" OR "activation-induced cytidine deaminase" OR apeced OR "aire autoimmune regulator" OR ak2 OR "Adenylate kinase 2" OR "Adenylate kinase-2" OR alpi OR "intestinal alkaline phosphatase" OR ap1s3 OR "adaptor related protein complex 1" OR ap3b1 "adaptor related protein complex 3" OR ap3d1 OR "APOL-I" OR "APOL I" OR "Apolipoprotein L" OR arhgef1 OR arpc1b OR atm OR "ataxia telangiectasia mutated" OR atp6ap1 OR b2m OR "beta 2-Microglobulin" OR "beta 2 Microglobulin" OR bach2 OR baff OR "B-Cell Activation factor" OR "B Cell Activation factor" OR "B-Cell Activating factor" OR "B Cell Activating factor" OR bcl10 OR bcl11 OR bcl11b OR bcli10 OR blm OR blnk OR "B Cell linker" OR bloc1s6 OR "biogenesis of lysosomal organelles complex 1" OR brca1 OR brca2 OR brip1 OR btk OR "B Cell linker" OR "Bruton tyrosine kinase" OR c1qa OR c1qb OR c1qc OR "Complement C1q" OR "Complement C1s" OR "Complement activating enzyme" OR "Complement system protein" OR c1r OR c1s OR c2 OR c3 OR c4 OR c4a OR c4b OR c5 OR c6 OR c7 OR c8 OR c8a OR c8b OR c8g OR c9 OR card11 OR "Caspase recruitment domain" OR card14 OR card15 OR card9 OR carmil1 OR carmil2 OR casp8 OR "Caspase Eight" OR "Caspase 8" OR "Caspase-8" OR casp10 OR "Caspase ten" OR "Caspase 10" OR "Caspase-10" OR ccbe1 OR "calcium-binding EGF domains" OR "calcium binding EGF domains" OR cd16 OR cd19 OR cd20 OR cd21 OR cd25 OR cd27 OR cd3* OR cd3d OR cd3e OR cd3g OR cd3z OR cd40 OR cd40lg OR cd45 OR cd46 OR cd55 OR cd59 OR cd70 OR cd79a OR cd79b OR cd79 OR cd8 OR cd81 OR cd8a OR cdca7 OR cebpe OR "C-EBP epsilon" OR "CEBP epsilon" OR "C EBP epsilon" OR "CCAAT enhancer binding protein" OR cfb OR cfd OR cfh OR cfhr1 OR cfhr2 OR cfhr3 OR cfhr4 OR cfhr5 OR cfi OR cfp OR cftr OR "complement factor b" OR "complement factor d" OR "complement factor h" OR "complement factor i" OR "complement factor p" OR "cystic fibrosis transmembrane conductance regulator" OR chd7 OR "chromodomain helicase DNA binding protein 7" OR cib1 OR ciita OR "class II transactivator" OR "Class II Major Histocompatibility Complex Transactivator" OR clbp OR clcn7 OR "chloride channel 7" OR clpb OR coh1 OR copa OR "COPI Coat Complex Subunit Alpha" OR "Coronin-1A" OR "Coronin 1A" OR coro1a OR csf2ra OR csf3r OR csf2rb OR "colony stimulating factor 2" OR "colony stimulating factor 3" OR ctc1 "conserved telomere maintenance component 1" OR ctla4 OR "cytotoxic T lymphocyte associated protein 4" OR ctps1 OR "ctp synthase 1" OR ctsc OR "cathepsin C" OR cxcr4 OR cxcra OR cyba OR cybb OR cybc1 OR "cytochrome b-245" OR dbr1 OR dclre1b OR dclre1c OR "DNA cross-link repair" OR "DNA cross link repair" OR def6 OR "Del1op13-p14" OR del1op13 OR dkc1 OR "Dyskerin Pseudouridine Synthase" OR dnajc21 OR dnase1l3 OR dnase2 OR dnmt3b OR "DNA methyltransferase 3B" OR "DNA methyltransferase 3 beta" OR dock2 OR dock8 OR "dedicator of cytokinesis" OR efl1 OR "euphorbia factor L1" OR elane OR "elastase neutrophil expressed" OR epg5 OR "ectopic P-granules autophagy protein 5" OR "ectopic P granules autophagy protein 5" OR erbb2ip OR ercc4 OR ercc6l2 OR extl3 OR faap24 OR fadd OR "Fas-Associated Death Domain" OR "Fas Associated Death Domain" OR fanca OR fancb OR fancc OR fancd2 OR fance OR fanf OR fanci OR fancl OR fancm OR "Fanconi Anemia Complementation Group" OR DKCA6 OR "Coats plus syndrome due to CTC1 deficiency" OR DKCX1 OR EVI1 OR "MECOM deficiency" OR DKCB2 OR DKCB1 OR DKCB6 OR DKCA4 OR DKCB5 OR "Ataxia Pancytopenia Syndrome" OR BMFS1 OR "SRP72-deficiency" OR "Coats plus syndrome due to STN1 deficiency" OR DKCA1 OR DKCA2 OR DKCB4 OR DKCA3 OR BMFS5 OR DKCB3 OR fas OR faslg OR fat4 OR fcgr3a OR "Fc gamma receptor" OR fcho1 OR fcn3 OR "ficolin 3" OR fremt1 OR "fermitin 1" OR fermt3 OR "fermitin 3" OR foxn1 OR foxp3 OR "forkhead box P3" OR fpr1 OR "Formyl Peptide Receptor 1" OR g6pc3 OR "Glucosephosphate Dehydrogenase" OR "glucose 6 phosphatase catalytic" OR g6pt1 OR "gata-2" OR "gata 2" OR gata2 OR "gata binding protein 2" OR gfi1 OR "growth factor independent 1" OR gins1 OR havcr2 OR hax1 OR "HS1-associated protein X-1" OR "HS1 associated protein X 1" OR "HS1 associated protein X1" OR "HCLS1-associated protein X-1" OR " HCLS1 associated protein X 1" OR " HCLS1 associated protein X1" OR hells OR homx OR hmox1 OR hyou1 OR icos OR "Inducible T-Cell Co-Stimulator" OR "Inducible T Cell Costimulator" OR icoslg OR ifih1 OR "Interferon Induced Helicase c domain" OR ifnar1 OR ifnar2 OR ifngr1 OR ifngr2 OR ifnyr1 OR ifnyr2 OR "interferon gamma receptor" OR "inf gamma r1" OR "inf gamma r2" OR ighm OR igkc OR "immunoglobulin kappa constant" OR igll1 OR "immunoglobulin lambda-like polypeptide 1" OR "immunoglobulin lambda like polypeptide 1" OR ikbkb OR ikbkg OR "I kappa b kinase" OR "inhibitor of nuclear factor b kinase" OR ikzf1 OR il10 OR "IL-10" OR "IL 10" OR "interleukin 10" OR "interleukin-10" OR il10ra OR "IL-10RA" OR "IL 10RA" OR "interleukin 10RA" OR "interleukin-10RA" OR il10rb OR "IL-10RB" OR "IL 10RB" OR "interleukin 10RB" OR "interleukin-10RB" OR il12r OR "IL-12R" OR "IL 12R" OR "interleukin 12R" OR "interleukin-12R" OR il12rb OR "IL-12RB" OR "IL 12RB" OR "interleukin 12RB" OR "interleukin-12RB" OR il12rb1 OR "IL-12RB1" OR "IL 12RB1" OR "interleukin 12RB1" OR "interleukin-12RB1" OR il12rb2 OR "IL-12RB2" OR "IL 12RB2" OR "interleukin 12RB2" OR "interleukin-12RB2" OR il17f OR "IL-17F" OR "IL 17F" OR "interleukin 17F" OR "interleukin-17F" OR il17ra OR "IL-17RA" OR "IL 17RA" OR "interleukin 17RA" OR "interleukin-17RA" OR il17rc OR "IL-17RC" OR "IL 17RC" OR "interleukin 17RC" OR "interleukin-17RC" OR il18bp OR "IL-18BP" OR "IL 18BP" OR "interleukin 18BP" OR "interleukin-18BP" OR il1rn OR "IL-1RN" OR "IL 1RN" OR "interleukin 1RN" OR "interleukin-1RN" OR il23r OR "IL-23R" OR "IL 23R" OR "interleukin 23R" OR "interleukin-23R" OR il2i OR "IL-2I" OR "IL 2I" OR "interleukin 2I" OR "interleukin-2I" OR il2ir OR "IL-2IR" OR "IL 2IR" OR "interleukin 2IR" OR "interleukin-2IR" OR il2ira OR "IL-2IRA" OR "IL 2IRA" OR "interleukin 2IRA" OR "interleukin-2IRA" OR il2irb OR "IL-2IRB" OR "IL 2IRB" OR "interleukin 2IRB" OR "interleukin-2IRB" OR il2irg OR "IL-2IRG" OR "IL 2IRG" OR "interleukin 2IRG" OR "interleukin-2IRG" OR il36rn OR "IL-36RN" OR "IL 36RN" OR "interleukin 36RN" OR "interleukin-36RN" OR il6r OR "IL-6R" OR "IL 6R" OR "interleukin 6R" OR "interleukin-6R" OR il6st OR "IL-6ST" OR "IL 6ST" OR "interleukin 6ST" OR "interleukin-6ST" OR ilr7 OR "IL-R7" OR "IL R7" OR "interleukin R7" OR "interleukin- R7" OR ino80 OR irak1 OR "IRAK-1" OR "IRAK 1" OR "interleukin 1" OR "interleukin-1" OR irak4 OR "IRAK-4" OR "IRAK 4" OR "interleukin 4" OR "interleukin-4" OR irf2bp2 OR irf3 OR irf4 OR irf7 OR irf8 OR irf9 OR "interferon regulatory factor 2" OR "interferon regulatory factor 3" OR "interferon regulatory factor 4" OR "interferon regulatory factor 7" OR "interferon regulatory factor 8" OR "interferon regulatory factor 9" OR isg15 OR "itchy homolog e3" OR "itchy e3" OR itgam OR "integrin subunit alpha" OR "integrin subunit beta" OR itgb2 OR itk OR "il2 inducible t cell kinase" OR jagn1 OR "jagunal homolg 1" OR jak1 OR jak3 OR "janus kinase 1" OR "janus kinase 3" OR kdm6a OR kindlin3 OR kmt2a OR kmt2d OR kras OR lamtor2 OR lat OR lck OR "leukocyte c terminal src kinase" OR "leukocyte c-terminal src kinase" OR lig1 OR "ligase I" OR lig4 OR "ligase IV" OR lpin2 OR "lipin 2" OR lrba OR "lipopolysaccharide responsive beige like anchor protein " OR "lps responsive beige like anchor protein " OR lyst OR "lysosomal trafficking regulator" OR mad2l2 OR magt1 OR "magnesium transporter 1" OR malt1 OR "Mannan Binding Lectin" OR "Mannose Binding Lectin" OR map3k14 OR "mitogen-activated protein kinase 14" OR masp2 OR "MBL-associated serine protease 2" OR "MBL associated serine protease 2" OR mbs1 OR mcm4 OR "minichromosome maintenance complex component 4" OR mefv OR mkl1 OR mogs OR "mannosyl-oligosaccharide glucosidase" OR "mannosyl oligosaccharide glucosidase" OR msh6 OR "muts homolog 6" OR msn OR moesin OR mst1 OR "macrophage stimulating 1" OR mthfd1 OR "methylenetetrahydrofolate dehydrogenase" OR mvk OR "Mevalonate Kinase" OR myd88 OR mysm1 OR nbas OR nbs1 OR ncf1 OR ncf2 OR ncf4 OR "neutrophil cytosolic factor 1" OR "neutrophil cytosolic factor 2" OR "neutrophil cytosolic factor 4" OR ncstn OR nicastrin OR nfat5 OR "nuclear factor of activated T-cells 5" OR "nuclear factor of activated T cells 5" OR nfe2l2 OR nfkb1 OR nfkb2 OR "nuclear factor kappa b subunit 1" OR "nuclear factor kappa b subunit 2" OR nfkbia OR "nfkb inhibitor alpha" OR nhej1 OR "nonhomologous end-joining factor 1" OR "nonhomologous end joining factor 1" OR nik OR "nfkb inducing kinase" OR "nf-kb inducing kinase" OR "nf kb inducing kinase" OR nlrc4 OR "nlf family CARD domain containing 4" OR nlrp1 OR nlrp12 OR nlrp3 OR "nlr family pyrin domain containing 12" OR "nlr family pyrin domain containing 3" OR "nlr family pyrin domain containing 1" OR nod2 OR "Nucleotide Binding Oligomerization Domain Containing 2" OR nola2 OR nola3 OR "nucleolar protein family A member 2" OR "nucleolar protein family A member 3" OR nras OR nsmce3 OR oas1 OR "ORAI-I" OR "ORAI I" OR ostm1 OR otulin OR ox40 OR "tumor necrosis factor superfamily member 4 " OR "TNF receptor superfamily member 4 " OR "TNF superfamily member 4" OR p22phox OR p40phox OR p47phox OR p67phox OR "Neutrophil cytosol factor P22" OR "Neutrophil cytosol factor P40" OR "Neutrophil cytosol factor P47" OR "Neutrophil cytosol factor P67" OR palb2 OR parn OR "poly A-specific ribonuclease" OR "poly A specific ribonuclease" OR "polyA specific ribonuclease" OR pepd OR pgm3 OR "Phosphoglucomutase 3" OR pi3kr1 OR "Phosphoinositide-3-Kinase Regulatory Subunit 1" OR "Phosphoinositide 3 Kinase Regulatory Subunit 1" OR pik3cd OR pik3r1 OR plcg2 OR "phospholipase C gamma 2" OR plekhm1 OR pms2 OR "PMS1 Homolog 2" OR pnp OR "Purine Nucleoside Phosphorylase" OR pola1 OR pold1 OR pold2 OR pole1 OR pole2 OR "DNA polymerase epsilon" OR polr31 OR polr3c OR polr3f OR prf1 OR "perforin 1" OR "PRKC Delta" OR "protein kinase c delta" OR prkcd OR prkcdc OR psen OR psenen OR psmb8 OR "Proteasome 20S Subunit Beta 8" OR psmg2 OR pstpip1 OR "Proline Serine Threonine Phosphatase Interacting Protein 1 " OR pten OR ptprc OR rab27a OR rac2 OR "Rac 2" OR "Rac Family Small GTPase 2" OR rad51 OR rag OR rag1 OR rag2 OR "recombination activating gene 1" OR "recombination activating 1" OR "recombination activating gene 2" OR "recombination activating 2" OR ranbp2 OR rand51c OR rasgrp1 OR rbck1 OR "RANBP2-type" OR "RANBP2 type" OR recql3 OR "RECQ Protein-Like 3" OR "RECQ Protein Like 3" OR rel OR rela OR relb OR rfwd3 OR rfx5 OR "Regulatory Factor X5" OR rfxank OR "Regulatory Factor X Associated" OR rfxap OR rhoh OR ripk1 OR rmrp OR "RNA component of mitochondrial RNA processing endoribonuclease" OR rnaseh2a OR rnaseh2b OR rnaseh2c OR "Ribonuclease H2 Subunit A" OR "Ribonuclease H2 Subunit B" OR "Ribonuclease H2 Subunit C" OR rnf168 OR rnf31 OR "ring finger protein 168" OR "ring finger protein 31" OR rnu4atac OR robld3 OR "Roadblock domain containing 3" OR rorc OR "RAR Related Orphan Receptor C" OR rpsa OR "Ribosomal Protein SA" OR rtel1 OR "Regulator of Telomere Elongation Helicase 1" OR samd9 OR samd9l OR samhd1 OR sbds OR sec61a1 OR sema3e OR "semaphorin 3E" OR serping1 OR sh2d1a OR "SH2 Domain Containing 1A" OR sh3bp2 OR "SH3 Domain Binding Protein 2" OR sh3kbp1 OR skiv2l OR slc29a3 OR slc35c1 OR slc37a4 OR "Solute Carrier Family 29 Member 3" OR "Solute Carrier Family 35 Member C1" OR "Solute Carrier Family 37 Member A4" OR slc39a7 OR slc46a1 OR "Solute Carrier Family 39 Member A7" OR "Solute Carrier Family 46 Member A1" OR slc7a7 OR "Solute Carrier Family 7 Member A7" OR slx4 OR smarcal1 OR smarcd2 OR snx10 OR sp110 OR spink5 OR "Serine Peptidase Inhibitor Kazal Type 5" OR sppl2a OR srp54 OR srp72 OR "STAT 1" OR stat1 OR "STAT 2" OR stat2 OR "STAT 3" OR stat3 OR "STAT 5b" OR stat5b OR "STAT 5" OR stat5 OR "Signal Transducer and Activator of Transcription 5b" OR "Signal Transducer and Activator of Transcription 5" OR "Signal Transducer and Activator of Transcription 1" OR "Signal Transducer and Activator of Transcription 2" OR "Signal Transducer and Activator of Transcription 3" OR "stim-1" OR stim1 OR "Stromal Interaction Molecule 1" OR stk4 OR stn1 OR stx11 OR "Syntaxin 11" OR stxbp2 OR "syntaxin binding protein 2" OR tap1 OR tap2 OR "transporter 1" OR "transporter 2" OR tapbp OR "tap binding protein" OR taz OR tafazzin OR tbk1 OR "tank binding kinase 1" OR tbx1 OR "t box transcription factor 1" OR tcf3 OR "transcription factor 3" OR tcirg1 OR tcn2 OR "Transcobalamin 2" OR "tcr-alpha" OR "tcr alpha" OR "t cell receptor alpha" OR terc OR "Telomerase RNA Component" OR tert OR "Telomerase Reverse Transcriptase" OR tfrc OR tgfb1 OR tgfbr1 OR tgfbr2 OR thbd OR thrombomodulin OR ticam1 OR tinf2 OR "TRF1-interacting nuclear factor 2" OR "TRF1 interacting nuclear factor 2" OR "TERF1-interacting nuclear factor 2" OR "TERF1 interacting nuclear factor 2" OR thymotaxin OR tirap OR tlr3 OR "toll like receptor 3" OR tmc6 OR tmc8 OR "Transmembrane Channel Like 6" OR "Transmembrane Channel Like 8" OR tmem173 OR "Transmembrane Protein 173" OR tnfaip3 OR tnfrsf1 OR "TNF Receptor Superfamily Member" OR tnfrsf11a OR tnfrsf13b OR tnfrsf13c OR tnfrsf1a OR tnfrsf4 OR tnfrsf6 OR tnfrsf9 OR tnfsf11 OR tnfsf12 OR tnfsf6 OR top2b OR tp53 OR tpp1 OR tpp2 OR "Tripeptidyl Peptidase 1" OR "Tripeptidyl Peptidase 2" OR trac OR traf3 OR "TNF Receptor Associated Factor 3" OR traf3ip2 OR trex1 OR "Three Prime Repair Exonuclease 1" OR trim22 OR trnt1 OR "TRNA Nucleotidyl Transferase 1" OR ttc37 OR "Tetratricopeptide Repeat Domain 37" OR ttc7a OR "Tetratricopeptide Repeat Domain 7A" OR txb1 OR tyk2 OR "Tyrosine Kinase 2" OR ube2t OR unc13d OR "unc-13 homolog D" OR "unc 13 homolog D" OR unc93b1 OR "unc-93 homolog b1" OR "unc 93 homolog b1" OR ung OR "Uracil DNA Glycosylase" OR usb1 OR usp18 OR vps13b OR "Vacuolar Protein Sorting 13 Homolog B" OR vps45 OR vps45a OR "Vacuolar Protein Sorting 45 A" OR "was gene" OR "was protein" OR "WASP Actin Nucleation Promoting Factor" OR wdr1 OR wipf1 OR wrap53 OR xiap OR xrcc1 OR xrcc9 OR "ZAP-70" OR zap70 OR "Zeta Chain Of T Cell Receptor Associated Protein Kinase 70" OR zbtb24 OR "BTB Domain Containing 24" OR znf341 OR "interleukin-2 receptor subunit gamma" OR "interleukin 2 receptor subunit gamma" OR "moebius syndrome" OR "X-Linked" OR "X Linked") AND PEER(yes)) AND ((autoimmun* OR immunodeficien* OR deficien*) AND PEER(yes))) OR "3") AND ((coronavir* OR "corona virus" OR "corona pandemic" OR betacoronavir* OR covid19 OR covid OR ncov OR "CoV 2" OR cov2 OR sarscov2 OR sars2 OR 2019ncov OR "novel CoV" OR "wuhan virus" OR NCOV19 OR "solidarity trial" OR "operation warp speed" OR COVAX OR "ACT-Accelerator" OR BNT162b2 OR comirnaty OR "mRNA-1273" OR CoviShield OR AZD1222 OR "Sputnik V" OR CoronaVac OR "BBIBP-CorV" OR "Ad26.CoV2.S" OR "JNJ-78436735" OR Ad26COVS1 OR VAC31518 OR EpiVacCorona OR Convidicea OR "Ad5-nCoV" OR Covaxin OR CoviVac OR ZF2001 OR "NVX-CoV2373" OR "ZyCoV-D" OR "CIGB 66" OR CVnCoV OR "INO-4800" OR "VIR-7831" OR "UB-612" OR "BNT162" OR "Soberana 1" OR "Soberana 2 " OR "B.1.1.7" OR "VOC 202012/01" OR "VOC202012/01" OR "VUI 202012/01" OR "VUI202012/01" OR "501Y.V1" OR "UK Variant" OR "Kent Variant" OR "VOC 202102/02" OR "VOC202102/02" OR "B.1.351" OR "VOC 202012/02" OR "VOC202012/02" OR "20H/501.V2" OR "20H/501Y.V2" OR "501Y.V2" OR "501.V2" OR "South African Variant" OR "B.1.1.28.1" OR "B.1.1.28" OR "B.1.1.248" OR "VOC 202101/02" OR "VOC202101/02" OR "VUI202101/02" OR "VUI 202101/02" OR "501Y.V3" OR "brazil Variant" OR "P.1 variant" OR "P.1 lineage" OR "Lineage P.1" OR "B.1.427" OR "B.1.429" OR "20C/S:452R" OR "CAL.20C" OR "CAL.20C/L452R" OR "20C/L452R" OR "20-C variant" OR CAVUI1 OR "GH/451R.V1" OR "B.1.526" OR "20C/S:484K" OR "B.1.1.28.3" OR "P3 variant" OR "P.3 Strain" OR "Lineage P.3" OR "P.3 Lineage" OR "PHL-B.1.1.28" OR "VUI-21MAR-02" OR "B.1.1.28.2" OR "20B/S.484K" OR "P.2 variant" OR "P.2 strain" OR "P.2 Lineage" OR "GR clade" OR "VUI 202101/01" OR "VUI202101/01" OR "B.1.177" OR "20A.EU1" OR "20A.EU2" OR "Variant 20A" OR "B.1.525" OR "G/484K.V3" OR "20A/S:484K" OR UK1188 OR "B.1.616" OR "20C/ B1" OR "clade 20C" OR "B.1.617" OR "VUI-21APR-01" OR "B.1.618" OR "VUI-202102/01" OR "VUI-21FEB-01" OR D614G OR N501Y OR A570D OR P681H OR K417N OR E484K OR "K417N/T" OR L452R OR S477N OR D253G OR T951 OR A701V OR 8477H OR V30L OR A220V OR T445C OR C6286T OR C26801G OR E484L OR D66H OR Y144V OR Q677H OR D215G OR 484K OR P681R OR D651G OR E484Q OR F888L OR F565L OR V1176F OR F157S OR L452R OR D614G OR T781I OR T859N OR D950H OR L5F OR K417T OR L18F OR T20N OR P26S OR R190S OR H655Y OR T1027I OR A701V OR K417N OR S494P OR T716I OR S982A OR D1118H OR K1191N) AND PEER(yes))) OR "5" OR "6") AND (("severe acute respiratory" OR pneumonia*) AND PEER(yes)) AND (outbreak* AND PEER(yes))](https://www.proquest.com/recentsearches.recentsearchtabview.recentsearchesgridview.scrolledrecentsearchlist.checkdbssearchlink:rerunsearch/27FDD4DC881C4115PQ/None?site=central&t:ac=RecentSearches)  Limits: English, 2019-, peer-reviewed | 02/28/2022 | 268 articles  -58 duplicates  =210 articles |
| Medline DATE 1946 | ((  ((11q23 OR 11q23del OR acd OR ace OR "angiotensin converting enzyme" OR acp5 OR "acid phosphate 5" OR actb OR ada OR ada2 OR adam17 OR tace OR "adenosine deaminase" OR adar1 OR aicda OR "AID enzyme" OR "activation induced cytidine deaminase" OR "activation-induced cytidine deaminase" OR apeced OR "aire autoimmune regulator" OR ak2 OR "Adenylate kinase 2" OR "Adenylate kinase-2" OR alpi OR "intestinal alkaline phosphatase" OR AP1S3 OR "adaptor related protein complex 1" OR AP3B1 "adaptor related protein complex 3" OR AP3D1 OR "APOL-I" OR "APOL I" OR "Apolipoprotein L" OR ARHGEF1 OR ARPC1B OR atm OR "ataxia telangiectasia mutated" OR ATP6AP1 OR b2m OR "beta 2-Microglobulin" OR "beta 2 Microglobulin" OR bach2 OR baff OR "B-Cell Activation factor" OR "B Cell Activation factor" OR "B-Cell Activating factor" OR "B Cell Activating factor" OR BCL10 OR BCL11 OR BCL11B OR BCLI10 OR blm OR blnk OR "B Cell linker" OR BLOC1S6 OR "biogenesis of lysosomal organelles complex 1" OR brca1 OR brca2 OR brip1 OR btk OR "B Cell linker" OR "Bruton tyrosine kinase" OR C1QA OR C1QB OR C1QC OR "Complement C1q" OR "Complement C1s" OR "Complement activating enzyme" OR "Complement system protein" OR c1r OR c1s OR c2 OR c3 OR c4 OR c4a OR c4b OR c5 OR c6 OR c7 OR c8 OR c8a OR c8b OR c8g OR c9 OR card11 OR "Caspase recruitment domain" OR card14 OR card15 OR card9 OR CARMIL1 OR CARMIL2 OR casp8 OR "Caspase Eight" OR "Caspase 8" OR "Caspase-8" OR casp10 OR "Caspase ten" OR "Caspase 10" OR "Caspase-10" OR ccbe1 OR "calcium-binding EGF domains" OR "calcium binding EGF domains" OR cd16 OR cd19 OR cd20 OR cd21 OR cd25 OR cd27 OR cd3* OR cd3d OR cd3e OR cd3g OR cd3z OR cd40 OR cd40lg OR cd45 OR cd46 OR cd55 OR cd59 OR cd70 OR cd79a OR cd79b OR cd79 OR cd8 OR cd81 OR cd8a OR cdca7 OR cebpe OR "C-EBP epsilon" OR "CEBP epsilon" OR "C EBP epsilon" OR "CCAAT enhancer binding protein" OR cfb OR cfd OR cfh OR cfhr1 OR cfhr2 OR cfhr3 OR cfhr4 OR cfhr5 OR cfi OR cfp OR cftr OR "complement factor b" OR "complement factor d" OR "complement factor h" OR "complement factor i" OR "complement factor p" OR "cystic fibrosis transmembrane conductance regulator" OR chd7 OR "chromodomain helicase DNA binding protein 7" OR CIB1 OR ciita OR "class II transactivator" OR "Class II Major Histocompatibility Complex Transactivator" OR clbp OR clcn7 OR "chloride channel 7" OR clpb OR coh1 OR copa OR "COPI Coat Complex Subunit Alpha" OR "Coronin-1A" OR "Coronin 1A" OR CORO1A OR CSF2RA OR CSF3R OR CSF2RB OR "colony stimulating factor 2" OR "colony stimulating factor 3" OR ctc1 "conserved telomere maintenance component 1" OR CTLA4 OR "cytotoxic T lymphocyte associated protein 4" OR CTPS1 OR "ctp synthase 1" OR CTSC OR "cathepsin C" OR CXCR4 OR CXCRA OR CYBA OR CYBB OR CYBC1 OR "cytochrome b-245" OR DBR1 OR DCLRE1B OR DCLRE1C OR "DNA cross-link repair" OR "DNA cross link repair" OR def6 OR "Del1op13-p14" OR Del1op13 OR dkc1 OR "Dyskerin Pseudouridine Synthase" OR DNAJC21 OR DNASE1L3 OR DNASE2 OR DNMT3B OR "DNA methyltransferase 3B" OR "DNA methyltransferase 3 beta" OR dock2 OR dock8 OR "dedicator of cytokinesis" OR efl1 OR "euphorbia factor L1" OR elane OR "elastase neutrophil expressed" OR epg5 OR "ectopic P-granules autophagy protein 5" OR "ectopic P granules autophagy protein 5" OR erbb2ip OR ercc4 OR ercc6L2 OR extl3 OR faap24 OR fadd OR "Fas-Associated Death Domain" OR "Fas Associated Death Domain" OR fanca OR fancb OR fancc OR fancd2 OR fance OR fanf OR fanci OR fancl OR fancm OR "Fanconi Anemia Complementation Group" OR DKCA6 OR "Coats plus syndrome due to CTC1 deficiency" OR DKCX1 OR EVI1 OR "MECOM deficiency" OR DKCB2 OR DKCB1 OR DKCB6 OR DKCA4 OR DKCB5 OR "Ataxia Pancytopenia Syndrome" OR BMFS1 OR "SRP72-deficiency" OR "Coats plus syndrome due to STN1 deficiency" OR DKCA1 OR DKCA2 OR DKCB4 OR DKCA3 OR BMFS5 OR DKCB3 OR fas OR faslg OR fat4 OR fcgr3a OR "Fc gamma receptor" OR fcho1 OR fcn3 OR "ficolin 3" OR fremt1 OR "fermitin 1" OR fermt3 OR "fermitin 3" OR foxn1 OR foxp3 OR "forkhead box P3" OR fpr1 OR "Formyl Peptide Receptor 1" OR G6PC3 OR "Glucosephosphate Dehydrogenase" OR "glucose 6 phosphatase catalytic" OR G6PT1 OR "gata-2" OR "gata 2" OR GATA2 OR "gata binding protein 2" OR GFI1 OR "growth factor independent 1" OR GINS1 OR HAVCR2 OR hax1 OR "HS1-associated protein X-1" OR "HS1 associated protein X 1" OR "HS1 associated protein X1" OR "HCLS1-associated protein X-1" OR " HCLS1 associated protein X 1" OR " HCLS1 associated protein X1" OR hells OR homx OR hmox1 OR hyou1 OR ICOS OR "Inducible T-Cell Co-Stimulator" OR "Inducible T Cell Costimulator" OR ICOSLG OR IFIH1 OR "Interferon Induced Helicase c domain" OR IFNAR1 OR IFNAR2 OR IFNGR1 OR IFNGR2 OR IFNyR1 OR IFNyR2 OR "interferon gamma receptor" OR "inf gamma r1" OR "inf gamma r2" OR ighm OR igkc OR "immunoglobulin kappa constant" OR igll1 OR "immunoglobulin lambda-like polypeptide 1" OR "immunoglobulin lambda like polypeptide 1" OR IKBKB OR ikbkg OR "I kappa b kinase" OR "inhibitor of nuclear factor b kinase" OR ikzf1 OR IL10 OR "IL-10" OR "IL 10" OR "interleukin 10" OR "interleukin-10" OR IL10RA OR "IL-10RA" OR "IL 10RA" OR "interleukin 10RA" OR "interleukin-10RA" OR IL10RB OR "IL-10RB" OR "IL 10RB" OR "interleukin 10RB" OR "interleukin-10RB" OR IL12R OR "IL-12R" OR "IL 12R" OR "interleukin 12R" OR "interleukin-12R" OR IL12RB OR "IL-12RB" OR "IL 12RB" OR "interleukin 12RB" OR "interleukin-12RB" OR IL12RB1 OR "IL-12RB1" OR "IL 12RB1" OR "interleukin 12RB1" OR "interleukin-12RB1" OR IL12RB2 OR "IL-12RB2" OR "IL 12RB2" OR "interleukin 12RB2" OR "interleukin-12RB2" OR IL17F OR "IL-17F" OR "IL 17F" OR "interleukin 17F" OR "interleukin-17F" OR IL17RA OR "IL-17RA" OR "IL 17RA" OR "interleukin 17RA" OR "interleukin-17RA" OR IL17RC OR "IL-17RC" OR "IL 17RC" OR "interleukin 17RC" OR "interleukin-17RC" OR IL18BP OR "IL-18BP" OR "IL 18BP" OR "interleukin 18BP" OR "interleukin-18BP" OR IL1RN OR "IL-1RN" OR "IL 1RN" OR "interleukin 1RN" OR "interleukin-1RN" OR IL23R OR "IL-23R" OR "IL 23R" OR "interleukin 23R" OR "interleukin-23R" OR IL2I OR "IL-2I" OR "IL 2I" OR "interleukin 2I" OR "interleukin-2I" OR IL2IR OR "IL-2IR" OR "IL 2IR" OR "interleukin 2IR" OR "interleukin-2IR" OR IL2IRA OR "IL-2IRA" OR "IL 2IRA" OR "interleukin 2IRA" OR "interleukin-2IRA" OR IL2IRB OR "IL-2IRB" OR "IL 2IRB" OR "interleukin 2IRB" OR "interleukin-2IRB" OR IL2IRG OR "IL-2IRG" OR "IL 2IRG" OR "interleukin 2IRG" OR "interleukin-2IRG" OR IL36RN OR "IL-36RN" OR "IL 36RN" OR "interleukin 36RN" OR "interleukin-36RN" OR IL6R OR "IL-6R" OR "IL 6R" OR "interleukin 6R" OR "interleukin-6R" OR IL6ST OR "IL-6ST" OR "IL 6ST" OR "interleukin 6ST" OR "interleukin-6ST" OR ILR7 OR "IL-R7" OR "IL R7" OR "interleukin R7" OR "interleukin- R7" OR ino80 OR IRAK1 OR "IRAK-1" OR "IRAK 1" OR "interleukin 1" OR "interleukin-1" OR IRAK4 OR "IRAK-4" OR "IRAK 4" OR "interleukin 4" OR "interleukin-4" OR IRF2BP2 OR IRF3 OR IRF4 OR IRF7 OR IRF8 OR IRF9 OR "interferon regulatory factor 2" OR "interferon regulatory factor 3" OR "interferon regulatory factor 4" OR "interferon regulatory factor 7" OR "interferon regulatory factor 8" OR "interferon regulatory factor 9" OR ISG15 OR "itchy homolog e3" OR "itchy e3" OR ITGAM OR "integrin subunit alpha" OR "integrin subunit beta" OR ITGB2 OR ITK OR "il2 inducible t cell kinase" OR JAGN1 OR "jagunal homolg 1" OR JAK1 OR JAK3 OR "janus kinase 1" OR "janus kinase 3" OR KDM6A OR KINDLIN3 OR KMT2A OR KMT2D OR KRAS OR LAMTOR2 OR LAT OR LCK OR "leukocyte c terminal src kinase" OR "leukocyte c-terminal src kinase" OR LIG1 OR "ligase I" OR LIG4 OR "ligase IV" OR LPIN2 OR "lipin 2" OR LRBA OR "lipopolysaccharide responsive beige like anchor protein " OR "lps responsive beige like anchor protein " OR LYST OR "lysosomal trafficking regulator" OR MAD2L2 OR MAGT1 OR "magnesium transporter 1" OR malt1 OR "Mannan Binding Lectin" OR "Mannose Binding Lectin" OR MAP3K14 OR "mitogen-activated protein kinase 14" OR masp2 OR "MBL-associated serine protease 2" OR "MBL associated serine protease 2" OR mbs1 OR mcm4 OR "minichromosome maintenance complex component 4" OR mefv OR mkl1 OR mogs OR "mannosyl-oligosaccharide glucosidase" OR "mannosyl oligosaccharide glucosidase" OR msh6 OR "muts homolog 6" OR msn OR moesin OR mst1 OR "macrophage stimulating 1" OR mthfd1 OR "methylenetetrahydrofolate dehydrogenase" OR mvk OR "Mevalonate Kinase" OR myd88 OR mysm1 OR nbas OR nbs1 OR ncf1 OR ncf2 OR ncf4 OR "neutrophil cytosolic factor 1" OR "neutrophil cytosolic factor 2" OR "neutrophil cytosolic factor 4" OR ncstn OR nicastrin OR nfat5 OR "nuclear factor of activated T-cells 5" OR "nuclear factor of activated T cells 5" OR NFE2L2 OR nfkb1 OR nfkb2 OR "nuclear factor kappa b subunit 1" OR "nuclear factor kappa b subunit 2" OR NFKBIA OR "nfkb inhibitor alpha" OR NHEJ1 OR "nonhomologous end-joining factor 1" OR "nonhomologous end joining factor 1" OR nik OR "nfkb inducing kinase" OR "nf-kb inducing kinase" OR "nf kb inducing kinase" OR NLRC4 OR "nlf family CARD domain containing 4" OR nlrp1 OR nlrp12 OR nlrp3 OR "nlr family pyrin domain containing 12" OR "nlr family pyrin domain containing 3" OR "nlr family pyrin domain containing 1" OR nod2 OR "Nucleotide Binding Oligomerization Domain Containing 2" OR nola2 OR nola3 OR "nucleolar protein family A member 2" OR "nucleolar protein family A member 3" OR nras OR nsmce3 OR oas1 OR "ORAI-I" OR "ORAI I" OR ostm1 OR otulin OR ox40 OR "tumor necrosis factor superfamily member 4 " OR "TNF receptor superfamily member 4 " OR "TNF superfamily member 4" OR p22phox OR p40phox OR p47phox OR p67phox OR "Neutrophil cytosol factor P22" OR "Neutrophil cytosol factor P40" OR "Neutrophil cytosol factor P47" OR "Neutrophil cytosol factor P67" OR palb2 OR parn OR "poly A-specific ribonuclease" OR "poly A specific ribonuclease" OR "polyA specific ribonuclease" OR pepd OR pgm3 OR "Phosphoglucomutase 3" OR PI3KR1 OR "Phosphoinositide-3-Kinase Regulatory Subunit 1" OR "Phosphoinositide 3 Kinase Regulatory Subunit 1" OR PIK3CD OR PIK3R1 OR PLCG2 OR "phospholipase C gamma 2" OR PLEKHM1 OR PMS2 OR "PMS1 Homolog 2" OR pnp OR "Purine Nucleoside Phosphorylase" OR pola1 OR pold1OR pold2 OR pole1 OR pole2 OR "DNA polymerase epsilon" OR polr31 OR polr3c OR polr3f OR prf1 OR "perforin 1" OR "PRKC Delta" OR "protein kinase c delta" OR PRKCD OR PRKCDC OR psen OR psenen OR PSMB8 OR "Proteasome 20S Subunit Beta 8" OR psmg2 OR PSTPIP1 OR "Proline Serine Threonine Phosphatase Interacting Protein 1 " OR pten OR ptprc OR RAB27A OR rac2 OR "Rac 2" OR "Rac Family Small GTPase 2" OR rad51 OR rag OR rag1 OR rag2 OR "recombination activating gene 1" OR "recombination activating 1" OR "recombination activating gene 2" OR "recombination activating 2" OR RANBP2 OR RAND51C OR RASGRP1 OR RBCK1 OR "RANBP2-type" OR "RANBP2 type" OR RECQL3 OR "RECQ Protein-Like 3" OR "RECQ Protein Like 3" OR rel OR rela OR RelB OR RFWD3 OR RFX5 OR "Regulatory Factor X5" OR RFXANK OR "Regulatory Factor X Associated" OR RFXAP OR rhoh OR RIPK1 OR RMRP OR "RNA component of mitochondrial RNA processing endoribonuclease" OR RNASEH2A OR RNASEH2B OR RNASEH2C OR "Ribonuclease H2 Subunit A" OR "Ribonuclease H2 Subunit B" OR "Ribonuclease H2 Subunit C" OR RNF168 OR RNF31 OR "ring finger protein 168" OR "ring finger protein 31" OR RNU4ATAC OR ROBLD3 OR "Roadblock domain containing 3" OR rorc OR "RAR Related Orphan Receptor C" OR rpsa OR "Ribosomal Protein SA" OR rtel1 OR "Regulator of Telomere Elongation Helicase 1" OR samd9 OR samd9l OR samhd1 OR sbds OR SEC61A1 OR SEMA3E OR "semaphorin 3E" OR SERPING1 OR SH2D1A OR "SH2 Domain Containing 1A" OR SH3BP2 OR "SH3 Domain Binding Protein 2" OR SH3KBP1 OR SKIV2L OR SLC29A3 OR SLC35C1 OR SLC37A4 OR "Solute Carrier Family 29 Member 3" OR "Solute Carrier Family 35 Member C1" OR "Solute Carrier Family 37 Member A4" OR SLC39A7 OR SLC46A1 OR "Solute Carrier Family 39 Member A7" OR "Solute Carrier Family 46 Member A1" OR SLC7A7 OR "Solute Carrier Family 7 Member A7" OR slx4 OR SMARCAL1 OR SMARCD2 OR snx10 OR SP110 OR SPINK5 OR "Serine Peptidase Inhibitor Kazal Type 5" OR SPPL2a OR SRP54 OR SRP72 OR "STAT 1" OR stat1 OR "STAT 2" OR stat2 OR "STAT 3" OR stat3 OR "STAT 5b" OR stat5b OR "STAT 5" OR stat5 OR "Signal Transducer and Activator of Transcription 5b" OR "Signal Transducer and Activator of Transcription 5" OR "Signal Transducer and Activator of Transcription 1" OR "Signal Transducer and Activator of Transcription 2" OR "Signal Transducer and Activator of Transcription 3" OR "stim-1" OR stim1 OR "Stromal Interaction Molecule 1" OR stk4 OR stn1 OR stx11 OR "Syntaxin 11" OR stxbp2 OR "syntaxin binding protein 2" OR tap1 OR tap2 OR "transporter 1" OR "transporter 2" OR tapbp OR "tap binding protein" OR taz OR tafazzin OR tbk1 OR "tank binding kinase 1" OR tbx1 OR "t box transcription factor 1" OR tcf3 OR "transcription factor 3" OR TCIRG1 OR tcn2 OR "Transcobalamin 2" OR "tcr-alpha" OR "tcr alpha" OR "t cell receptor alpha" OR terc OR "Telomerase RNA Component" OR tert OR "Telomerase Reverse Transcriptase" OR TFRC OR TGFB1 OR TGFBR1 OR TGFBR2 OR thbd OR Thrombomodulin OR ticam1 OR tinf2 OR "TRF1-interacting nuclear factor 2" OR "TRF1 interacting nuclear factor 2" OR "TERF1-interacting nuclear factor 2" OR "TERF1 interacting nuclear factor 2" OR Thymotaxin OR tirap OR tlr3 OR "toll like receptor 3" OR tmc6 OR tmc8 OR "Transmembrane Channel Like 6" OR "Transmembrane Channel Like 8" OR tmem173 OR "Transmembrane Protein 173" OR TNFAIP3 OR TNFRSF1 OR "TNF Receptor Superfamily Member" OR TNFRSF11A OR TNFRSF13B OR TNFRSF13C OR TNFRSF1A OR TNFRSF4 OR TNFRSF6 OR TNFRSF9 OR TNFSF11 OR TNFSF12 OR TNFSF6 OR TOP2B OR TP53 OR tpp1 OR tpp2 OR "Tripeptidyl Peptidase 1" OR "Tripeptidyl Peptidase 2" OR trac OR traf3 OR "TNF Receptor Associated Factor 3" OR TRAF3IP2 OR trex1 OR "Three Prime Repair Exonuclease 1" OR trim22 OR trnt1 OR "TRNA Nucleotidyl Transferase 1" OR ttc37 OR "Tetratricopeptide Repeat Domain 37" OR ttc7a OR "Tetratricopeptide Repeat Domain 7A" OR txb1 OR tyk2 OR "Tyrosine Kinase 2" OR ube2t OR unc13d OR "unc-13 homolog D" OR "unc 13 homolog D" OR UNC93B1 OR "unc-93 homolog b1" OR "unc 93 homolog b1" OR ung OR "Uracil DNA Glycosylase" OR usb1 OR usp18 OR VPS13B OR "Vacuolar Protein Sorting 13 Homolog B" OR vps45 OR vps45a OR "Vacuolar Protein Sorting 45 A" OR "was gene" OR "was protein" OR "WASP Actin Nucleation Promoting Factor" OR wdr1 OR WIPF1 OR WRAP53 OR XIAP OR XRCC1 OR XRCC9 OR "ZAP-70" OR zap70 OR "Zeta Chain Of T Cell Receptor Associated Protein Kinase 70" OR ZBTB24 OR "BTB Domain Containing 24" OR ZNF341 OR "interleukin-2 receptor subunit gamma" OR "interleukin 2 receptor subunit gamma") adj2 (delet* OR mutat* OR gene* OR autoimmun* OR deficien* OR immunodeficien* )).ti,ab. OR ((moebius) adj2 (syndrom*)).ti,ab. OR (("X-Linked" OR "X Linked") adj2 (Agammaglobulinemia OR inhibitor OR cgd OR Lymphoproliferative OR myelodysplasia OR neutropenia OR "reticulate pigmentary" OR autoimmun* OR deficien* OR immunodeficien*)).ti,ab. OR ((gene* OR primary OR inborn* OR congenital*) adj2 (autoimmun* OR deficien* OR immunodeficien*)).ti,ab.    AND  (autoimmun* OR immunodeficien* OR deficien* ).ti,ab.  )  OR  (("inborn errors" OR "inborn error").ti,ab. AND (immun* OR autoimmun* OR deficien* OR immunodeficien*).ti,ab.)  )  AND  (coronavir* OR corona virus* OR corona pandemic* OR betacoronavir* OR covid19 OR covid OR nCoV OR novel CoV OR CoV 2 OR CoV2 OR sarscov2 OR sars2 OR 2019nCoV OR wuhan virus* OR NCOV19 OR solidarity trial OR operation warp speed OR COVAX OR "ACT-Accelerator" OR BNT162b2 OR comirnaty OR "mRNA-1273" OR CoviShield OR AZD1222 OR Sputnik V OR CoronaVac OR "BBIBP-CorV" OR "Ad26.CoV2.S" OR "JNJ-78436735" OR Ad26COVS1 OR VAC31518 OR EpiVacCorona OR Convidicea OR "Ad5-nCoV" OR Covaxin OR CoviVac OR ZF2001 OR "NVX-CoV2373" OR "ZyCoV-D" OR CIGB 66 OR CVnCoV OR "INO-4800" OR "VIR-7831" OR "UB-612" OR BNT162 OR Soberana 1 OR Soberana 2 OR "B.1.1.7" OR "VOC 202012/01" OR "VOC202012/01" OR "VUI 202012/01" OR "VUI202012/01" OR "501Y.V1" OR UK Variant OR Kent Variant OR "VOC 202102/02" OR "VOC202102/02" OR "B.1.351" OR "VOC 202012/02" OR "VOC202012/02" OR "20H/501.V2" OR "20H/501Y.V2" OR "501Y.V2" OR "501.V2" OR South African Variant OR "B.1.1.28.1" OR "B.1.1.28" OR "B.1.1.248" OR "VOC 202101/02" OR "VOC202101/02" OR "VUI202101/02" OR "VUI 202101/02" OR "501Y.V3" OR brazil Variant OR "P.1 variant" OR "P.1 lineage" OR "Lineage P.1" OR "B.1.427" OR "B.1.429" OR "20C/S:452R" OR "CAL.20C" OR "CAL.20C/L452R" OR "20C/L452R" OR "20-C variant" OR "CAVUI1" OR "GH/451R.V1" OR "B.1.526" OR "20C/S:484K" OR "B.1.1.28.3" OR P3 variant OR "P.3 Strain" OR "Lineage P.3" OR "P.3 Lineage" OR "PHL-B.1.1.28" OR "VUI-21MAR-02" OR "B.1.1.28.2" OR "20B/S.484K" OR "P.2 variant" OR "P.2 strain" OR "P.2 Lineage" OR GR clade OR "VUI 202101/01" OR "VUI202101/01" OR "B.1.177" OR "20A.EU1" OR "20A.EU2" OR Variant 20A OR "B.1.525" OR "G/484K.V3" OR "20A/S:484K" OR "UK1188" OR "B.1.616" OR "20C/ B1" OR clade 20C OR "B.1.617" OR "VUI-21APR-01" OR "B.1.618" OR "VUI-202102/01" OR "VUI-21FEB-01" OR D614G OR N501Y OR A570D OR P681H OR K417N OR E484K OR "K417N/T" OR L452R OR S477N OR D253G OR T951 OR A701V OR 8477H OR V30L OR A220V OR T445C OR C6286T OR C26801G OR E484L OR D66H OR Y144V OR Q677H OR D215G OR 484K OR P681R OR D651G OR E484Q OR F888L OR F565L OR V1176F OR F157S OR L452R OR D614G OR T781I OR T859N OR D950H OR L5F OR K417T OR L18F OR T20N OR P26S OR R190S OR H655Y OR T1027I OR A701V OR K417N OR S494P OR T716I OR S982A OR D1118H OR K1191N).mp. OR (sars AND cov).ti,ab. OR ((wuhan OR hubei OR huanan) AND (severe acute respiratory OR pneumonia*) AND outbreak*).mp. OR Coronavirus Infections/ OR Coronavirus/ OR betacoronavirus/ OR exp COVID-19 Vaccines/ OR exp COVID-19 Testing  Limits: English, humans, 2019 | 02/28/2022 | 362 articles  -73 duplicates  =289 articles |
| Embase  (Ovid)  1947- | ((  ((11q23 OR 11q23del OR acd OR ace OR "angiotensin converting enzyme" OR acp5 OR "acid phosphate 5" OR actb OR ada OR ada2 OR adam17 OR tace OR "adenosine deaminase" OR adar1 OR aicda OR "AID enzyme" OR "activation induced cytidine deaminase" OR "activation-induced cytidine deaminase" OR apeced OR "aire autoimmune regulator" OR ak2 OR "Adenylate kinase 2" OR "Adenylate kinase-2" OR alpi OR "intestinal alkaline phosphatase" OR AP1S3 OR "adaptor related protein complex 1" OR AP3B1 "adaptor related protein complex 3" OR AP3D1 OR "APOL-I" OR "APOL I" OR "Apolipoprotein L" OR ARHGEF1 OR ARPC1B OR atm OR "ataxia telangiectasia mutated" OR ATP6AP1 OR b2m OR "beta 2-Microglobulin" OR "beta 2 Microglobulin" OR bach2 OR baff OR "B-Cell Activation factor" OR "B Cell Activation factor" OR "B-Cell Activating factor" OR "B Cell Activating factor" OR BCL10 OR BCL11 OR BCL11B OR BCLI10 OR blm OR blnk OR "B Cell linker" OR BLOC1S6 OR "biogenesis of lysosomal organelles complex 1" OR brca1 OR brca2 OR brip1 OR btk OR "B Cell linker" OR "Bruton tyrosine kinase" OR C1QA OR C1QB OR C1QC OR "Complement C1q" OR "Complement C1s" OR "Complement activating enzyme" OR "Complement system protein" OR c1r OR c1s OR c2 OR c3 OR c4 OR c4a OR c4b OR c5 OR c6 OR c7 OR c8 OR c8a OR c8b OR c8g OR c9 OR card11 OR "Caspase recruitment domain" OR card14 OR card15 OR card9 OR CARMIL1 OR CARMIL2 OR casp8 OR "Caspase Eight" OR "Caspase 8" OR "Caspase-8" OR casp10 OR "Caspase ten" OR "Caspase 10" OR "Caspase-10" OR ccbe1 OR "calcium-binding EGF domains" OR "calcium binding EGF domains" OR cd16 OR cd19 OR cd20 OR cd21 OR cd25 OR cd27 OR cd3* OR cd3d OR cd3e OR cd3g OR cd3z OR cd40 OR cd40lg OR cd45 OR cd46 OR cd55 OR cd59 OR cd70 OR cd79a OR cd79b OR cd79 OR cd8 OR cd81 OR cd8a OR cdca7 OR cebpe OR "C-EBP epsilon" OR "CEBP epsilon" OR "C EBP epsilon" OR "CCAAT enhancer binding protein" OR cfb OR cfd OR cfh OR cfhr1 OR cfhr2 OR cfhr3 OR cfhr4 OR cfhr5 OR cfi OR cfp OR cftr OR "complement factor b" OR "complement factor d" OR "complement factor h" OR "complement factor i" OR "complement factor p" OR "cystic fibrosis transmembrane conductance regulator" OR chd7 OR "chromodomain helicase DNA binding protein 7" OR CIB1 OR ciita OR "class II transactivator" OR "Class II Major Histocompatibility Complex Transactivator" OR clbp OR clcn7 OR "chloride channel 7" OR clpb OR coh1 OR copa OR "COPI Coat Complex Subunit Alpha" OR "Coronin-1A" OR "Coronin 1A" OR CORO1A OR CSF2RA OR CSF3R OR CSF2RB OR "colony stimulating factor 2" OR "colony stimulating factor 3" OR ctc1 "conserved telomere maintenance component 1" OR CTLA4 OR "cytotoxic T lymphocyte associated protein 4" OR CTPS1 OR "ctp synthase 1" OR CTSC OR "cathepsin C" OR CXCR4 OR CXCRA OR CYBA OR CYBB OR CYBC1 OR "cytochrome b-245" OR DBR1 OR DCLRE1B OR DCLRE1C OR "DNA cross-link repair" OR "DNA cross link repair" OR def6 OR "Del1op13-p14" OR Del1op13 OR dkc1 OR "Dyskerin Pseudouridine Synthase" OR DNAJC21 OR DNASE1L3 OR DNASE2 OR DNMT3B OR "DNA methyltransferase 3B" OR "DNA methyltransferase 3 beta" OR dock2 OR dock8 OR "dedicator of cytokinesis" OR efl1 OR "euphorbia factor L1" OR elane OR "elastase neutrophil expressed" OR epg5 OR "ectopic P-granules autophagy protein 5" OR "ectopic P granules autophagy protein 5" OR erbb2ip OR ercc4 OR ercc6L2 OR extl3 OR faap24 OR fadd OR "Fas-Associated Death Domain" OR "Fas Associated Death Domain" OR fanca OR fancb OR fancc OR fancd2 OR fance OR fanf OR fanci OR fancl OR fancm OR "Fanconi Anemia Complementation Group" OR DKCA6 OR "Coats plus syndrome due to CTC1 deficiency" OR DKCX1 OR EVI1 OR "MECOM deficiency" OR DKCB2 OR DKCB1 OR DKCB6 OR DKCA4 OR DKCB5 OR "Ataxia Pancytopenia Syndrome" OR BMFS1 OR "SRP72-deficiency" OR "Coats plus syndrome due to STN1 deficiency" OR DKCA1 OR DKCA2 OR DKCB4 OR DKCA3 OR BMFS5 OR DKCB3 OR fas OR faslg OR fat4 OR fcgr3a OR "Fc gamma receptor" OR fcho1 OR fcn3 OR "ficolin 3" OR fremt1 OR "fermitin 1" OR fermt3 OR "fermitin 3" OR foxn1 OR foxp3 OR "forkhead box P3" OR fpr1 OR "Formyl Peptide Receptor 1" OR G6PC3 OR "Glucosephosphate Dehydrogenase" OR "glucose 6 phosphatase catalytic" OR G6PT1 OR "gata-2" OR "gata 2" OR GATA2 OR "gata binding protein 2" OR GFI1 OR "growth factor independent 1" OR GINS1 OR HAVCR2 OR hax1 OR "HS1-associated protein X-1" OR "HS1 associated protein X 1" OR "HS1 associated protein X1" OR "HCLS1-associated protein X-1" OR " HCLS1 associated protein X 1" OR " HCLS1 associated protein X1" OR hells OR homx OR hmox1 OR hyou1 OR ICOS OR "Inducible T-Cell Co-Stimulator" OR "Inducible T Cell Costimulator" OR ICOSLG OR IFIH1 OR "Interferon Induced Helicase c domain" OR IFNAR1 OR IFNAR2 OR IFNGR1 OR IFNGR2 OR IFNyR1 OR IFNyR2 OR "interferon gamma receptor" OR "inf gamma r1" OR "inf gamma r2" OR ighm OR igkc OR "immunoglobulin kappa constant" OR igll1 OR "immunoglobulin lambda-like polypeptide 1" OR "immunoglobulin lambda like polypeptide 1" OR IKBKB OR ikbkg OR "I kappa b kinase" OR "inhibitor of nuclear factor b kinase" OR ikzf1 OR IL10 OR "IL-10" OR "IL 10" OR "interleukin 10" OR "interleukin-10" OR IL10RA OR "IL-10RA" OR "IL 10RA" OR "interleukin 10RA" OR "interleukin-10RA" OR IL10RB OR "IL-10RB" OR "IL 10RB" OR "interleukin 10RB" OR "interleukin-10RB" OR IL12R OR "IL-12R" OR "IL 12R" OR "interleukin 12R" OR "interleukin-12R" OR IL12RB OR "IL-12RB" OR "IL 12RB" OR "interleukin 12RB" OR "interleukin-12RB" OR IL12RB1 OR "IL-12RB1" OR "IL 12RB1" OR "interleukin 12RB1" OR "interleukin-12RB1" OR IL12RB2 OR "IL-12RB2" OR "IL 12RB2" OR "interleukin 12RB2" OR "interleukin-12RB2" OR IL17F OR "IL-17F" OR "IL 17F" OR "interleukin 17F" OR "interleukin-17F" OR IL17RA OR "IL-17RA" OR "IL 17RA" OR "interleukin 17RA" OR "interleukin-17RA" OR IL17RC OR "IL-17RC" OR "IL 17RC" OR "interleukin 17RC" OR "interleukin-17RC" OR IL18BP OR "IL-18BP" OR "IL 18BP" OR "interleukin 18BP" OR "interleukin-18BP" OR IL1RN OR "IL-1RN" OR "IL 1RN" OR "interleukin 1RN" OR "interleukin-1RN" OR IL23R OR "IL-23R" OR "IL 23R" OR "interleukin 23R" OR "interleukin-23R" OR IL2I OR "IL-2I" OR "IL 2I" OR "interleukin 2I" OR "interleukin-2I" OR IL2IR OR "IL-2IR" OR "IL 2IR" OR "interleukin 2IR" OR "interleukin-2IR" OR IL2IRA OR "IL-2IRA" OR "IL 2IRA" OR "interleukin 2IRA" OR "interleukin-2IRA" OR IL2IRB OR "IL-2IRB" OR "IL 2IRB" OR "interleukin 2IRB" OR "interleukin-2IRB" OR IL2IRG OR "IL-2IRG" OR "IL 2IRG" OR "interleukin 2IRG" OR "interleukin-2IRG" OR IL36RN OR "IL-36RN" OR "IL 36RN" OR "interleukin 36RN" OR "interleukin-36RN" OR IL6R OR "IL-6R" OR "IL 6R" OR "interleukin 6R" OR "interleukin-6R" OR IL6ST OR "IL-6ST" OR "IL 6ST" OR "interleukin 6ST" OR "interleukin-6ST" OR ILR7 OR "IL-R7" OR "IL R7" OR "interleukin R7" OR "interleukin- R7" OR ino80 OR IRAK1 OR "IRAK-1" OR "IRAK 1" OR "interleukin 1" OR "interleukin-1" OR IRAK4 OR "IRAK-4" OR "IRAK 4" OR "interleukin 4" OR "interleukin-4" OR IRF2BP2 OR IRF3 OR IRF4 OR IRF7 OR IRF8 OR IRF9 OR "interferon regulatory factor 2" OR "interferon regulatory factor 3" OR "interferon regulatory factor 4" OR "interferon regulatory factor 7" OR "interferon regulatory factor 8" OR "interferon regulatory factor 9" OR ISG15 OR "itchy homolog e3" OR "itchy e3" OR ITGAM OR "integrin subunit alpha" OR "integrin subunit beta" OR ITGB2 OR ITK OR "il2 inducible t cell kinase" OR JAGN1 OR "jagunal homolg 1" OR JAK1 OR JAK3 OR "janus kinase 1" OR "janus kinase 3" OR KDM6A OR KINDLIN3 OR KMT2A OR KMT2D OR KRAS OR LAMTOR2 OR LAT OR LCK OR "leukocyte c terminal src kinase" OR "leukocyte c-terminal src kinase" OR LIG1 OR "ligase I" OR LIG4 OR "ligase IV" OR LPIN2 OR "lipin 2" OR LRBA OR "lipopolysaccharide responsive beige like anchor protein " OR "lps responsive beige like anchor protein " OR LYST OR "lysosomal trafficking regulator" OR MAD2L2 OR MAGT1 OR "magnesium transporter 1" OR malt1 OR "Mannan Binding Lectin" OR "Mannose Binding Lectin" OR MAP3K14 OR "mitogen-activated protein kinase 14" OR masp2 OR "MBL-associated serine protease 2" OR "MBL associated serine protease 2" OR mbs1 OR mcm4 OR "minichromosome maintenance complex component 4" OR mefv OR mkl1 OR mogs OR "mannosyl-oligosaccharide glucosidase" OR "mannosyl oligosaccharide glucosidase" OR msh6 OR "muts homolog 6" OR msn OR moesin OR mst1 OR "macrophage stimulating 1" OR mthfd1 OR "methylenetetrahydrofolate dehydrogenase" OR mvk OR "Mevalonate Kinase" OR myd88 OR mysm1 OR nbas OR nbs1 OR ncf1 OR ncf2 OR ncf4 OR "neutrophil cytosolic factor 1" OR "neutrophil cytosolic factor 2" OR "neutrophil cytosolic factor 4" OR ncstn OR nicastrin OR nfat5 OR "nuclear factor of activated T-cells 5" OR "nuclear factor of activated T cells 5" OR NFE2L2 OR nfkb1 OR nfkb2 OR "nuclear factor kappa b subunit 1" OR "nuclear factor kappa b subunit 2" OR NFKBIA OR "nfkb inhibitor alpha" OR NHEJ1 OR "nonhomologous end-joining factor 1" OR "nonhomologous end joining factor 1" OR nik OR "nfkb inducing kinase" OR "nf-kb inducing kinase" OR "nf kb inducing kinase" OR NLRC4 OR "nlf family CARD domain containing 4" OR nlrp1 OR nlrp12 OR nlrp3 OR "nlr family pyrin domain containing 12" OR "nlr family pyrin domain containing 3" OR "nlr family pyrin domain containing 1" OR nod2 OR "Nucleotide Binding Oligomerization Domain Containing 2" OR nola2 OR nola3 OR "nucleolar protein family A member 2" OR "nucleolar protein family A member 3" OR nras OR nsmce3 OR oas1 OR "ORAI-I" OR "ORAI I" OR ostm1 OR otulin OR ox40 OR "tumor necrosis factor superfamily member 4 " OR "TNF receptor superfamily member 4 " OR "TNF superfamily member 4" OR p22phox OR p40phox OR p47phox OR p67phox OR "Neutrophil cytosol factor P22" OR "Neutrophil cytosol factor P40" OR "Neutrophil cytosol factor P47" OR "Neutrophil cytosol factor P67" OR palb2 OR parn OR "poly A-specific ribonuclease" OR "poly A specific ribonuclease" OR "polyA specific ribonuclease" OR pepd OR pgm3 OR "Phosphoglucomutase 3" OR PI3KR1 OR "Phosphoinositide-3-Kinase Regulatory Subunit 1" OR "Phosphoinositide 3 Kinase Regulatory Subunit 1" OR PIK3CD OR PIK3R1 OR PLCG2 OR "phospholipase C gamma 2" OR PLEKHM1 OR PMS2 OR "PMS1 Homolog 2" OR pnp OR "Purine Nucleoside Phosphorylase" OR pola1 OR pold1OR pold2 OR pole1 OR pole2 OR "DNA polymerase epsilon" OR polr31 OR polr3c OR polr3f OR prf1 OR "perforin 1" OR "PRKC Delta" OR "protein kinase c delta" OR PRKCD OR PRKCDC OR psen OR psenen OR PSMB8 OR "Proteasome 20S Subunit Beta 8" OR psmg2 OR PSTPIP1 OR "Proline Serine Threonine Phosphatase Interacting Protein 1 " OR pten OR ptprc OR RAB27A OR rac2 OR "Rac 2" OR "Rac Family Small GTPase 2" OR rad51 OR rag OR rag1 OR rag2 OR "recombination activating gene 1" OR "recombination activating 1" OR "recombination activating gene 2" OR "recombination activating 2" OR RANBP2 OR RAND51C OR RASGRP1 OR RBCK1 OR "RANBP2-type" OR "RANBP2 type" OR RECQL3 OR "RECQ Protein-Like 3" OR "RECQ Protein Like 3" OR rel OR rela OR RelB OR RFWD3 OR RFX5 OR "Regulatory Factor X5" OR RFXANK OR "Regulatory Factor X Associated" OR RFXAP OR rhoh OR RIPK1 OR RMRP OR "RNA component of mitochondrial RNA processing endoribonuclease" OR RNASEH2A OR RNASEH2B OR RNASEH2C OR "Ribonuclease H2 Subunit A" OR "Ribonuclease H2 Subunit B" OR "Ribonuclease H2 Subunit C" OR RNF168 OR RNF31 OR "ring finger protein 168" OR "ring finger protein 31" OR RNU4ATAC OR ROBLD3 OR "Roadblock domain containing 3" OR rorc OR "RAR Related Orphan Receptor C" OR rpsa OR "Ribosomal Protein SA" OR rtel1 OR "Regulator of Telomere Elongation Helicase 1" OR samd9 OR samd9l OR samhd1 OR sbds OR SEC61A1 OR SEMA3E OR "semaphorin 3E" OR SERPING1 OR SH2D1A OR "SH2 Domain Containing 1A" OR SH3BP2 OR "SH3 Domain Binding Protein 2" OR SH3KBP1 OR SKIV2L OR SLC29A3 OR SLC35C1 OR SLC37A4 OR "Solute Carrier Family 29 Member 3" OR "Solute Carrier Family 35 Member C1" OR "Solute Carrier Family 37 Member A4" OR SLC39A7 OR SLC46A1 OR "Solute Carrier Family 39 Member A7" OR "Solute Carrier Family 46 Member A1" OR SLC7A7 OR "Solute Carrier Family 7 Member A7" OR slx4 OR SMARCAL1 OR SMARCD2 OR snx10 OR SP110 OR SPINK5 OR "Serine Peptidase Inhibitor Kazal Type 5" OR SPPL2a OR SRP54 OR SRP72 OR "STAT 1" OR stat1 OR "STAT 2" OR stat2 OR "STAT 3" OR stat3 OR "STAT 5b" OR stat5b OR "STAT 5" OR stat5 OR "Signal Transducer and Activator of Transcription 5b" OR "Signal Transducer and Activator of Transcription 5" OR "Signal Transducer and Activator of Transcription 1" OR "Signal Transducer and Activator of Transcription 2" OR "Signal Transducer and Activator of Transcription 3" OR "stim-1" OR stim1 OR "Stromal Interaction Molecule 1" OR stk4 OR stn1 OR stx11 OR "Syntaxin 11" OR stxbp2 OR "syntaxin binding protein 2" OR tap1 OR tap2 OR "transporter 1" OR "transporter 2" OR tapbp OR "tap binding protein" OR taz OR tafazzin OR tbk1 OR "tank binding kinase 1" OR tbx1 OR "t box transcription factor 1" OR tcf3 OR "transcription factor 3" OR TCIRG1 OR tcn2 OR "Transcobalamin 2" OR "tcr-alpha" OR "tcr alpha" OR "t cell receptor alpha" OR terc OR "Telomerase RNA Component" OR tert OR "Telomerase Reverse Transcriptase" OR TFRC OR TGFB1 OR TGFBR1 OR TGFBR2 OR thbd OR Thrombomodulin OR ticam1 OR tinf2 OR "TRF1-interacting nuclear factor 2" OR "TRF1 interacting nuclear factor 2" OR "TERF1-interacting nuclear factor 2" OR "TERF1 interacting nuclear factor 2" OR Thymotaxin OR tirap OR tlr3 OR "toll like receptor 3" OR tmc6 OR tmc8 OR "Transmembrane Channel Like 6" OR "Transmembrane Channel Like 8" OR tmem173 OR "Transmembrane Protein 173" OR TNFAIP3 OR TNFRSF1 OR "TNF Receptor Superfamily Member" OR TNFRSF11A OR TNFRSF13B OR TNFRSF13C OR TNFRSF1A OR TNFRSF4 OR TNFRSF6 OR TNFRSF9 OR TNFSF11 OR TNFSF12 OR TNFSF6 OR TOP2B OR TP53 OR tpp1 OR tpp2 OR "Tripeptidyl Peptidase 1" OR "Tripeptidyl Peptidase 2" OR trac OR traf3 OR "TNF Receptor Associated Factor 3" OR TRAF3IP2 OR trex1 OR "Three Prime Repair Exonuclease 1" OR trim22 OR trnt1 OR "TRNA Nucleotidyl Transferase 1" OR ttc37 OR "Tetratricopeptide Repeat Domain 37" OR ttc7a OR "Tetratricopeptide Repeat Domain 7A" OR txb1 OR tyk2 OR "Tyrosine Kinase 2" OR ube2t OR unc13d OR "unc-13 homolog D" OR "unc 13 homolog D" OR UNC93B1 OR "unc-93 homolog b1" OR "unc 93 homolog b1" OR ung OR "Uracil DNA Glycosylase" OR usb1 OR usp18 OR VPS13B OR "Vacuolar Protein Sorting 13 Homolog B" OR vps45 OR vps45a OR "Vacuolar Protein Sorting 45 A" OR "was gene" OR "was protein" OR "WASP Actin Nucleation Promoting Factor" OR wdr1 OR WIPF1 OR WRAP53 OR XIAP OR XRCC1 OR XRCC9 OR "ZAP-70" OR zap70 OR "Zeta Chain Of T Cell Receptor Associated Protein Kinase 70" OR ZBTB24 OR "BTB Domain Containing 24" OR ZNF341 OR "interleukin-2 receptor subunit gamma" OR "interleukin 2 receptor subunit gamma") adj2 (delet* OR mutat* OR gene* OR autoimmun* OR deficien* OR immunodeficien* )).ti,ab. OR ((moebius) adj2 (syndrom*)).ti,ab. OR (("X-Linked" OR "X Linked") adj2 (Agammaglobulinemia OR inhibitor OR cgd OR Lymphoproliferative OR myelodysplasia OR neutropenia OR "reticulate pigmentary" OR autoimmun* OR deficien* OR immunodeficien*)).ti,ab. OR ((gene* OR primary OR inborn* OR congenital*) adj2 (autoimmun* OR deficien* OR immunodeficien*)).ti,ab.  AND  (autoimmun* OR immunodeficien* OR deficien* ).ti,ab.  )  OR  (("inborn errors" OR "inborn error").ti,ab. AND (immun* OR autoimmun* OR deficien* OR immunodeficien*).ti,ab.)  )  AND  (coronavir* OR corona virus* OR corona pandemic* OR betacoronavir* OR covid19 OR covid OR nCoV OR novel CoV OR CoV 2 OR CoV2 OR sarscov2 OR sars2 OR 2019nCoV OR wuhan virus* OR NCOV19 OR solidarity trial OR operation warp speed OR COVAX OR "ACT-Accelerator" OR BNT162b2 OR comirnaty OR "mRNA-1273" OR CoviShield OR AZD1222 OR Sputnik V OR CoronaVac OR "BBIBP-CorV" OR "Ad26.CoV2.S" OR "JNJ-78436735" OR Ad26COVS1 OR VAC31518 OR EpiVacCorona OR Convidicea OR "Ad5-nCoV" OR Covaxin OR CoviVac OR ZF2001 OR "NVX-CoV2373" OR "ZyCoV-D" OR CIGB 66 OR CVnCoV OR "INO-4800" OR "VIR-7831" OR "UB-612" OR BNT162 OR Soberana 1 OR Soberana 2 OR "B.1.1.7" OR "VOC 202012/01" OR "VOC202012/01" OR "VUI 202012/01" OR "VUI202012/01" OR "501Y.V1" OR UK Variant OR Kent Variant OR "VOC 202102/02" OR "VOC202102/02" OR "B.1.351" OR "VOC 202012/02" OR "VOC202012/02" OR "20H/501.V2" OR "20H/501Y.V2" OR "501Y.V2" OR "501.V2" OR South African Variant OR "B.1.1.28.1" OR "B.1.1.28" OR "B.1.1.248" OR "VOC 202101/02" OR "VOC202101/02" OR "VUI202101/02" OR "VUI 202101/02" OR "501Y.V3" OR brazil Variant OR "P.1 variant" OR "P.1 lineage" OR "Lineage P.1" OR "B.1.427" OR "B.1.429" OR "20C/S:452R" OR "CAL.20C" OR "CAL.20C/L452R" OR "20C/L452R" OR "20-C variant" OR "CAVUI1" OR "GH/451R.V1" OR "B.1.526" OR "20C/S:484K" OR "B.1.1.28.3" OR P3 variant OR "P.3 Strain" OR "Lineage P.3" OR "P.3 Lineage" OR "PHL-B.1.1.28" OR "VUI-21MAR-02" OR "B.1.1.28.2" OR "20B/S.484K" OR "P.2 variant" OR "P.2 strain" OR "P.2 Lineage" OR GR clade OR "VUI 202101/01" OR "VUI202101/01" OR "B.1.177" OR "20A.EU1" OR "20A.EU2" OR Variant 20A OR "B.1.525" OR "G/484K.V3" OR "20A/S:484K" OR "UK1188" OR "B.1.616" OR "20C/ B1" OR clade 20C OR "B.1.617" OR "VUI-21APR-01" OR "B.1.618" OR "VUI-202102/01" OR "VUI-21FEB-01" OR D614G OR N501Y OR A570D OR P681H OR K417N OR E484K OR "K417N/T" OR L452R OR S477N OR D253G OR T951 OR A701V OR 8477H OR V30L OR A220V OR T445C OR C6286T OR C26801G OR E484L OR D66H OR Y144V OR Q677H OR D215G OR 484K OR P681R OR D651G OR E484Q OR F888L OR F565L OR V1176F OR F157S OR L452R OR D614G OR T781I OR T859N OR D950H OR L5F OR K417T OR L18F OR T20N OR P26S OR R190S OR H655Y OR T1027I OR A701V OR K417N OR S494P OR T716I OR S982A OR D1118H OR K1191N).mp. OR (sars AND cov).ti,ab. OR ((wuhan OR hubei OR huanan) AND (severe acute respiratory OR pneumonia*) AND outbreak*).mp. OR Coronavirus Infection/ OR Coronavirus/ OR betacoronavirus/ OR exp coronavirus disease 2019  Limits: English, humans, 2019 | 02/28/2022 | 311 articles  - 140 duplicates  =171 articles |
| PubMed | (((((((((((11q23 OR 11q23del OR acd OR ace OR "angiotensin converting enzyme" OR acp5 OR "acid phosphate 5" OR actb OR ada OR ada2 OR adam17 OR tace OR "adenosine deaminase" OR adar1 OR aicda OR "AID enzyme" OR "activation induced cytidine deaminase" OR "activation-induced cytidine deaminase" OR apeced OR "aire autoimmune regulator" OR ak2 OR "Adenylate kinase 2" OR "Adenylate kinase-2" OR alpi OR "intestinal alkaline phosphatase" OR ap1s3 OR "adaptor related protein complex 1" OR ap3b1 "adaptor related protein complex 3" OR ap3d1 OR "APOL-I" OR "APOL I" OR "Apolipoprotein L" OR arhgef1 OR arpc1b OR atm OR "ataxia telangiectasia mutated" OR atp6ap1 OR b2m OR "beta 2-Microglobulin" OR "beta 2 Microglobulin" OR bach2 OR baff OR "B-Cell Activation factor" OR "B Cell Activation factor" OR "B-Cell Activating factor" OR "B Cell Activating factor" OR bcl10 OR bcl11 OR bcl11b OR bcli10 OR blm OR blnk OR "B Cell linker" OR bloc1s6 OR "biogenesis of lysosomal organelles complex 1" OR brca1 OR brca2 OR brip1 OR btk OR "B Cell linker" OR "Bruton tyrosine kinase" OR c1qa OR c1qb OR c1qc OR "Complement C1q" OR "Complement C1s" OR "Complement activating enzyme" OR "Complement system protein" OR c1r OR c1s OR c2 OR c3 OR c4 OR c4a OR c4b OR c5 OR c6 OR c7 OR c8 OR c8a OR c8b OR c8g OR c9 OR card11 OR "Caspase recruitment domain" OR card14 OR card15 OR card9 OR carmil1 OR carmil2 OR casp8 OR "Caspase Eight" OR "Caspase 8" OR "Caspase-8" OR casp10 OR "Caspase ten" OR "Caspase 10" OR "Caspase-10" OR ccbe1 OR "calcium-binding EGF domains" OR "calcium binding EGF domains" OR cd16 OR cd19 OR cd20 OR cd21 OR cd25 OR cd27 OR cd3* OR cd3d OR cd3e OR cd3g OR cd3z OR cd40 OR cd40lg OR cd45 OR cd46 OR cd55 OR cd59 OR cd70 OR cd79a OR cd79b OR cd79 OR cd8 OR cd81 OR cd8a OR cdca7 OR cebpe OR "C-EBP epsilon" OR "CEBP epsilon" OR "C EBP epsilon" OR "CCAAT enhancer binding protein" OR cfb OR cfd OR cfh OR cfhr1 OR cfhr2 OR cfhr3 OR cfhr4 OR cfhr5 OR cfi OR cfp OR cftr OR "complement factor b" OR "complement factor d" OR "complement factor h" OR "complement factor i" OR "complement factor p" OR "cystic fibrosis transmembrane conductance regulator" OR chd7 OR "chromodomain helicase DNA binding protein 7" OR cib1 OR ciita OR "class II transactivator" OR "Class II Major Histocompatibility Complex Transactivator" OR clbp OR clcn7 OR "chloride channel 7" OR clpb OR coh1 OR copa OR "COPI Coat Complex Subunit Alpha" OR "Coronin-1A" OR "Coronin 1A" OR coro1a OR csf2ra OR csf3r OR csf2rb OR "colony stimulating factor 2" OR "colony stimulating factor 3" OR ctc1 "conserved telomere maintenance component 1" OR ctla4 OR "cytotoxic T lymphocyte associated protein 4" OR ctps1 OR "ctp synthase 1" OR ctsc OR "cathepsin C" OR cxcr4 OR cxcra OR cyba OR cybb OR cybc1 OR "cytochrome b-245" OR dbr1 OR dclre1b OR dclre1c OR "DNA cross-link repair" OR "DNA cross link repair" OR def6 OR "Del1op13-p14" OR del1op13 OR dkc1 OR "Dyskerin Pseudouridine Synthase" OR dnajc21 OR dnase1l3 OR dnase2 OR dnmt3b OR "DNA methyltransferase 3B" OR "DNA methyltransferase 3 beta" OR dock2 OR dock8 OR "dedicator of cytokinesis" OR efl1 OR "euphorbia factor L1" OR elane OR "elastase neutrophil expressed" OR epg5 OR "ectopic P-granules autophagy protein 5" OR "ectopic P granules autophagy protein 5" OR erbb2ip OR ercc4 OR ercc6l2 OR extl3 OR faap24 OR fadd OR "Fas-Associated Death Domain" OR "Fas Associated Death Domain" OR fanca OR fancb OR fancc OR fancd2 OR fance OR fanf OR fanci OR fancl OR fancm OR "Fanconi Anemia Complementation Group" OR DKCA6 OR "Coats plus syndrome due to CTC1 deficiency" OR DKCX1 OR EVI1 OR "MECOM deficiency" OR DKCB2 OR DKCB1 OR DKCB6 OR DKCA4 OR DKCB5 OR "Ataxia Pancytopenia Syndrome" OR BMFS1 OR "SRP72-deficiency" OR "Coats plus syndrome due to STN1 deficiency" OR DKCA1 OR DKCA2 OR DKCB4 OR DKCA3 OR BMFS5 OR DKCB3 OR fas OR faslg OR fat4 OR fcgr3a OR "Fc gamma receptor" OR fcho1 OR fcn3 OR "ficolin 3" OR fremt1 OR "fermitin 1" OR fermt3 OR "fermitin 3" OR foxn1 OR foxp3 OR "forkhead box P3" OR fpr1 OR "Formyl Peptide Receptor 1" OR g6pc3 OR "Glucosephosphate Dehydrogenase" OR "glucose 6 phosphatase catalytic" OR g6pt1 OR "gata-2" OR "gata 2" OR gata2 OR "gata binding protein 2" OR gfi1 OR "growth factor independent 1" OR gins1 OR havcr2 OR hax1 OR "HS1-associated protein X-1" OR "HS1 associated protein X 1" OR "HS1 associated protein X1" OR "HCLS1-associated protein X-1" OR " HCLS1 associated protein X 1" OR " HCLS1 associated protein X1" OR hells OR homx OR hmox1 OR hyou1 OR icos OR "Inducible T-Cell Co-Stimulator" OR "Inducible T Cell Costimulator" OR icoslg OR ifih1 OR "Interferon Induced Helicase c domain" OR ifnar1 OR ifnar2 OR ifngr1 OR ifngr2 OR ifnyr1 OR ifnyr2 OR "interferon gamma receptor" OR "inf gamma r1" OR "inf gamma r2" OR ighm OR igkc OR "immunoglobulin kappa constant" OR igll1 OR "immunoglobulin lambda-like polypeptide 1" OR "immunoglobulin lambda like polypeptide 1" OR ikbkb OR ikbkg OR "I kappa b kinase" OR "inhibitor of nuclear factor b kinase" OR ikzf1 OR il10 OR "IL-10" OR "IL 10" OR "interleukin 10" OR "interleukin-10" OR il10ra OR "IL-10RA" OR "IL 10RA" OR "interleukin 10RA" OR "interleukin-10RA" OR il10rb OR "IL-10RB" OR "IL 10RB" OR "interleukin 10RB" OR "interleukin-10RB" OR il12r OR "IL-12R" OR "IL 12R" OR "interleukin 12R" OR "interleukin-12R" OR il12rb OR "IL-12RB" OR "IL 12RB" OR "interleukin 12RB" OR "interleukin-12RB" OR il12rb1 OR "IL-12RB1" OR "IL 12RB1" OR "interleukin 12RB1" OR "interleukin-12RB1" OR il12rb2 OR "IL-12RB2" OR "IL 12RB2" OR "interleukin 12RB2" OR "interleukin-12RB2" OR il17f OR "IL-17F" OR "IL 17F" OR "interleukin 17F" OR "interleukin-17F" OR il17ra OR "IL-17RA" OR "IL 17RA" OR "interleukin 17RA" OR "interleukin-17RA" OR il17rc OR "IL-17RC" OR "IL 17RC" OR "interleukin 17RC" OR "interleukin-17RC" OR il18bp OR "IL-18BP" OR "IL 18BP" OR "interleukin 18BP" OR "interleukin-18BP" OR il1rn OR "IL-1RN" OR "IL 1RN" OR "interleukin 1RN" OR "interleukin-1RN" OR il23r OR "IL-23R" OR "IL 23R" OR "interleukin 23R" OR "interleukin-23R" OR il2i OR "IL-2I" OR "IL 2I" OR "interleukin 2I" OR "interleukin-2I" OR il2ir OR "IL-2IR" OR "IL 2IR" OR "interleukin 2IR" OR "interleukin-2IR" OR il2ira OR "IL-2IRA" OR "IL 2IRA" OR "interleukin 2IRA" OR "interleukin-2IRA" OR il2irb OR "IL-2IRB" OR "IL 2IRB" OR "interleukin 2IRB" OR "interleukin-2IRB" OR il2irg OR "IL-2IRG" OR "IL 2IRG" OR "interleukin 2IRG" OR "interleukin-2IRG" OR il36rn OR "IL-36RN" OR "IL 36RN" OR "interleukin 36RN" OR "interleukin-36RN" OR il6r OR "IL-6R" OR "IL 6R" OR "interleukin 6R" OR "interleukin-6R" OR il6st OR "IL-6ST" OR "IL 6ST" OR "interleukin 6ST" OR "interleukin-6ST" OR ilr7 OR "IL-R7" OR "IL R7" OR "interleukin R7" OR "interleukin- R7" OR ino80 OR irak1 OR "IRAK-1" OR "IRAK 1" OR "interleukin 1" OR "interleukin-1" OR irak4 OR "IRAK-4" OR "IRAK 4" OR "interleukin 4" OR "interleukin-4" OR irf2bp2 OR irf3 OR irf4 OR irf7 OR irf8 OR irf9 OR "interferon regulatory factor 2" OR "interferon regulatory factor 3" OR "interferon regulatory factor 4" OR "interferon regulatory factor 7" OR "interferon regulatory factor 8" OR "interferon regulatory factor 9" OR isg15 OR "itchy homolog e3" OR "itchy e3" OR itgam OR "integrin subunit alpha" OR "integrin subunit beta" OR itgb2 OR itk OR "il2 inducible t cell kinase" OR jagn1 OR "jagunal homolg 1" OR jak1 OR jak3 OR "janus kinase 1" OR "janus kinase 3" OR kdm6a OR kindlin3 OR kmt2a OR kmt2d OR kras OR lamtor2 OR lat OR lck OR "leukocyte c terminal src kinase" OR "leukocyte c-terminal src kinase" OR lig1 OR "ligase I" OR lig4 OR "ligase IV" OR lpin2 OR "lipin 2" OR lrba OR "lipopolysaccharide responsive beige like anchor protein " OR "lps responsive beige like anchor protein " OR lyst OR "lysosomal trafficking regulator" OR mad2l2 OR magt1 OR "magnesium transporter 1" OR malt1 OR "Mannan Binding Lectin" OR "Mannose Binding Lectin" OR map3k14 OR "mitogen-activated protein kinase 14" OR masp2 OR "MBL-associated serine protease 2" OR "MBL associated serine protease 2" OR mbs1 OR mcm4 OR "minichromosome maintenance complex component 4" OR mefv OR mkl1 OR mogs OR "mannosyl-oligosaccharide glucosidase" OR "mannosyl oligosaccharide glucosidase" OR msh6 OR "muts homolog 6" OR msn OR moesin OR mst1 OR "macrophage stimulating 1" OR mthfd1 OR "methylenetetrahydrofolate dehydrogenase" OR mvk OR "Mevalonate Kinase" OR myd88 OR mysm1 OR nbas OR nbs1 OR ncf1 OR ncf2 OR ncf4 OR "neutrophil cytosolic factor 1" OR "neutrophil cytosolic factor 2" OR "neutrophil cytosolic factor 4" OR ncstn OR nicastrin OR nfat5 OR "nuclear factor of activated T-cells 5" OR "nuclear factor of activated T cells 5" OR nfe2l2 OR nfkb1 OR nfkb2 OR "nuclear factor kappa b subunit 1" OR "nuclear factor kappa b subunit 2" OR nfkbia OR "nfkb inhibitor alpha" OR nhej1 OR "nonhomologous end-joining factor 1" OR "nonhomologous end joining factor 1" OR nik OR "nfkb inducing kinase" OR "nf-kb inducing kinase" OR "nf kb inducing kinase" OR nlrc4 OR "nlf family CARD domain containing 4" OR nlrp1 OR nlrp12 OR nlrp3 OR "nlr family pyrin domain containing 12" OR "nlr family pyrin domain containing 3" OR "nlr family pyrin domain containing 1" OR nod2 OR "Nucleotide Binding Oligomerization Domain Containing 2" OR nola2 OR nola3 OR "nucleolar protein family A member 2" OR "nucleolar protein family A member 3" OR nras OR nsmce3 OR oas1 OR "ORAI-I" OR "ORAI I" OR ostm1 OR otulin OR ox40 OR "tumor necrosis factor superfamily member 4 " OR "TNF receptor superfamily member 4 " OR "TNF superfamily member 4" OR p22phox OR p40phox OR p47phox OR p67phox OR "Neutrophil cytosol factor P22" OR "Neutrophil cytosol factor P40" OR "Neutrophil cytosol factor P47" OR "Neutrophil cytosol factor P67" OR palb2 OR parn OR "poly A-specific ribonuclease" OR "poly A specific ribonuclease" OR "polyA specific ribonuclease" OR pepd OR pgm3 OR "Phosphoglucomutase 3" OR pi3kr1 OR "Phosphoinositide-3-Kinase Regulatory Subunit 1" OR "Phosphoinositide 3 Kinase Regulatory Subunit 1" OR pik3cd OR pik3r1 OR plcg2 OR "phospholipase C gamma 2" OR plekhm1 OR pms2 OR "PMS1 Homolog 2" OR pnp OR "Purine Nucleoside Phosphorylase" OR pola1 OR pold1 OR pold2 OR pole1 OR pole2 OR "DNA polymerase epsilon" OR polr31 OR polr3c OR polr3f OR prf1 OR "perforin 1" OR "PRKC Delta" OR "protein kinase c delta" OR prkcd OR prkcdc OR psen OR psenen OR psmb8 OR "Proteasome 20S Subunit Beta 8" OR psmg2 OR pstpip1 OR "Proline Serine Threonine Phosphatase Interacting Protein 1 " OR pten OR ptprc OR rab27a OR rac2 OR "Rac 2" OR "Rac Family Small GTPase 2" OR rad51 OR rag OR rag1 OR rag2 OR "recombination activating gene 1" OR "recombination activating 1" OR "recombination activating gene 2" OR "recombination activating 2" OR ranbp2 OR rand51c OR rasgrp1 OR rbck1 OR "RANBP2-type" OR "RANBP2 type" OR recql3 OR "RECQ Protein-Like 3" OR "RECQ Protein Like 3" OR rel OR rela OR relb OR rfwd3 OR rfx5 OR "Regulatory Factor X5" OR rfxank OR "Regulatory Factor X Associated" OR rfxap OR rhoh OR ripk1 OR rmrp OR "RNA component of mitochondrial RNA processing endoribonuclease" OR rnaseh2a OR rnaseh2b OR rnaseh2c OR "Ribonuclease H2 Subunit A" OR "Ribonuclease H2 Subunit B" OR "Ribonuclease H2 Subunit C" OR rnf168 OR rnf31 OR "ring finger protein 168" OR "ring finger protein 31" OR rnu4atac OR robld3 OR "Roadblock domain containing 3" OR rorc OR "RAR Related Orphan Receptor C" OR rpsa OR "Ribosomal Protein SA" OR rtel1 OR "Regulator of Telomere Elongation Helicase 1" OR samd9 OR samd9l OR samhd1 OR sbds OR sec61a1 OR sema3e OR "semaphorin 3E" OR serping1 OR sh2d1a OR "SH2 Domain Containing 1A" OR sh3bp2 OR "SH3 Domain Binding Protein 2" OR sh3kbp1 OR skiv2l OR slc29a3 OR slc35c1 OR slc37a4 OR "Solute Carrier Family 29 Member 3" OR "Solute Carrier Family 35 Member C1" OR "Solute Carrier Family 37 Member A4" OR slc39a7 OR slc46a1 OR "Solute Carrier Family 39 Member A7" OR "Solute Carrier Family 46 Member A1" OR slc7a7 OR "Solute Carrier Family 7 Member A7" OR slx4 OR smarcal1 OR smarcd2 OR snx10 OR sp110 OR spink5 OR "Serine Peptidase Inhibitor Kazal Type 5" OR sppl2a OR srp54 OR srp72 OR "STAT 1" OR stat1 OR "STAT 2" OR stat2 OR "STAT 3" OR stat3 OR "STAT 5b" OR stat5b OR "STAT 5" OR stat5 OR "Signal Transducer and Activator of Transcription 5b" OR "Signal Transducer and Activator of Transcription 5" OR "Signal Transducer and Activator of Transcription 1" OR "Signal Transducer and Activator of Transcription 2" OR "Signal Transducer and Activator of Transcription 3" OR "stim-1" OR stim1 OR "Stromal Interaction Molecule 1" OR stk4 OR stn1 OR stx11 OR "Syntaxin 11" OR stxbp2 OR "syntaxin binding protein 2" OR tap1 OR tap2 OR "transporter 1" OR "transporter 2" OR tapbp OR "tap binding protein" OR taz OR tafazzin OR tbk1 OR "tank binding kinase 1" OR tbx1 OR "t box transcription factor 1" OR tcf3 OR "transcription factor 3" OR tcirg1 OR tcn2 OR "Transcobalamin 2" OR "tcr-alpha" OR "tcr alpha" OR "t cell receptor alpha" OR terc OR "Telomerase RNA Component" OR tert OR "Telomerase Reverse Transcriptase" OR tfrc OR tgfb1 OR tgfbr1 OR tgfbr2 OR thbd OR thrombomodulin OR ticam1 OR tinf2 OR "TRF1-interacting nuclear factor 2" OR "TRF1 interacting nuclear factor 2" OR "TERF1-interacting nuclear factor 2" OR "TERF1 interacting nuclear factor 2" OR thymotaxin OR tirap OR tlr3 OR "toll like receptor 3" OR tmc6 OR tmc8 OR "Transmembrane Channel Like 6" OR "Transmembrane Channel Like 8" OR tmem173 OR "Transmembrane Protein 173" OR tnfaip3 OR tnfrsf1 OR "TNF Receptor Superfamily Member" OR tnfrsf11a OR tnfrsf13b OR tnfrsf13c OR tnfrsf1a OR tnfrsf4 OR tnfrsf6 OR tnfrsf9 OR tnfsf11 OR tnfsf12 OR tnfsf6 OR top2b OR tp53 OR tpp1 OR tpp2 OR "Tripeptidyl Peptidase 1" OR "Tripeptidyl Peptidase 2" OR trac OR traf3 OR "TNF Receptor Associated Factor 3" OR traf3ip2 OR trex1 OR "Three Prime Repair Exonuclease 1" OR trim22 OR trnt1 OR "TRNA Nucleotidyl Transferase 1" OR ttc37 OR "Tetratricopeptide Repeat Domain 37" OR ttc7a OR "Tetratricopeptide Repeat Domain 7A" OR txb1 OR tyk2 OR "Tyrosine Kinase 2" OR ube2t OR unc13d OR "unc-13 homolog D" OR "unc 13 homolog D" OR unc93b1 OR "unc-93 homolog b1" OR "unc 93 homolog b1" OR ung OR "Uracil DNA Glycosylase" OR usb1 OR usp18 OR vps13b OR "Vacuolar Protein Sorting 13 Homolog B" OR vps45 OR vps45a OR "Vacuolar Protein Sorting 45 A" OR "was gene" OR "was protein" OR "WASP Actin Nucleation Promoting Factor" OR wdr1 OR wipf1 OR wrap53 OR xiap OR xrcc1 OR xrcc9 OR "ZAP-70" OR zap70 OR "Zeta Chain Of T Cell Receptor Associated Protein Kinase 70" OR zbtb24 OR "BTB Domain Containing 24" OR znf341 OR "interleukin-2 receptor subunit gamma" OR "interleukin 2 receptor subunit gamma" OR "moebius syndrome" OR "X-Linked" OR "X Linked") AND (autoimmun* OR immunodeficien* OR deficien*)) OR ("inborn errors" OR "inborn error")) AND (immun* OR autoimmun* OR deficien* OR immunodeficien*)) AND (coronavir* OR corona virus* OR corona pandemic* OR betacoronavir* OR covid19 OR covid OR nCoV OR novel CoV OR CoV 2 OR CoV2 OR sarscov2 OR sars2 OR 2019nCoV OR wuhan virus* OR NCOV19 OR solidarity trial OR operation warp speed OR COVAX OR "ACT-Accelerator" OR BNT162b2 OR comirnaty OR "mRNA-1273" OR CoviShield OR AZD1222 OR Sputnik V OR CoronaVac OR "BBIBP-CorV" OR "Ad26.CoV2.S" OR "JNJ-78436735" OR Ad26COVS1 OR VAC31518 OR EpiVacCorona OR Convidicea OR "Ad5-nCoV" OR Covaxin OR CoviVac OR ZF2001 OR "NVX-CoV2373" OR "ZyCoV-D" OR CIGB 66 OR CVnCoV OR "INO-4800" OR "VIR-7831" OR "UB-612" OR BNT162 OR Soberana 1 OR Soberana 2 OR "B.1.1.7" OR "VOC 202012/01" OR "VOC202012/01" OR "VUI 202012/01" OR "VUI202012/01" OR "501Y.V1" OR UK Variant OR Kent Variant OR "VOC 202102/02" OR "VOC202102/02" OR "B.1.351" OR "VOC 202012/02" OR "VOC202012/02" OR "20H/501.V2" OR "20H/501Y.V2" OR "501Y.V2" OR "501.V2" OR South African Variant OR "B.1.1.28.1" OR "B.1.1.28" OR "B.1.1.248" OR "VOC 202101/02" OR "VOC202101/02" OR "VUI202101/02" OR "VUI 202101/02" OR "501Y.V3" OR brazil Variant OR "P.1 variant" OR "P.1 lineage" OR "Lineage P.1" OR "B.1.427" OR "B.1.429" OR "20C/S:452R" OR "CAL.20C" OR "CAL.20C/L452R" OR "20C/L452R" OR "20-C variant" OR "CAVUI1" OR "GH/451R.V1" OR "B.1.526" OR "20C/S:484K" OR "B.1.1.28.3" OR P3 variant OR "P.3 Strain" OR "Lineage P.3" OR "P.3 Lineage" OR "PHL-B.1.1.28" OR "VUI-21MAR-02" OR "B.1.1.28.2" OR "20B/S.484K" OR "P.2 variant" OR "P.2 strain" OR "P.2 Lineage" OR GR clade OR "VUI 202101/01" OR "VUI202101/01" OR "B.1.177" OR "20A.EU1" OR "20A.EU2" OR Variant 20A OR "B.1.525" OR "G/484K.V3" OR "20A/S:484K" OR "UK1188" OR "B.1.616" OR "20C/ B1" OR clade 20C OR "B.1.617" OR "VUI-21APR-01" OR "B.1.618" OR "VUI-202102/01" OR "VUI-21FEB-01" OR D614G OR N501Y OR A570D OR P681H OR K417N OR E484K OR "K417N/T" OR L452R OR S477N OR D253G OR T951 OR A701V OR 8477H OR V30L OR A220V OR T445C OR C6286T OR C26801G OR E484L OR D66H OR Y144V OR Q677H OR D215G OR 484K OR P681R OR D651G OR E484Q OR F888L OR F565L OR V1176F OR F157S OR L452R OR D614G OR T781I OR T859N OR D950H OR L5F OR K417T OR L18F OR T20N OR P26S OR R190S OR H655Y OR T1027I OR A701V OR K417N OR S494P OR T716I OR S982A OR D1118H OR K1191N)) OR (sars AND cov)) OR (wuhan OR hubei OR huanan)) AND (severe acute respiratory OR pneumonia*)) AND (outbreak*)) OR (Coronavirus Infections)) OR (Coronavirus)) OR (betacoronavirus)  Limits: English, 2019- | 02/28/2022 | 417 articles  -67 duplicates  =350 articles |
| CINAHL | ((  TI,AB ( 11q23 OR 11q23del OR acd OR ace OR "angiotensin converting enzyme" OR acp5 OR "acid phosphate 5" OR actb OR ada OR ada2 OR adam17 OR tace OR "adenosine deaminase" OR adar1 OR aicda OR "AID enzyme" OR "activation induced cytidine deaminase" OR "activation-induced cytidine deaminase" OR apeced OR "aire autoimmune regulator" OR ak2 OR "Adenylate kinase 2" OR "Adenylate kinase-2" OR alpi OR "intestinal alkaline phosphatase" OR ap1s3 OR "adaptor related protein complex 1" OR ap3b1 "adaptor related protein complex 3" OR ap3d1 OR "APOL-I" OR "APOL I" OR "Apolipoprotein L" OR arhgef1 OR arpc1b OR atm OR "ataxia telangiectasia mutated" OR atp6ap1 OR b2m OR "beta 2-Microglobulin" OR "beta 2 Microglobulin" OR bach2 OR baff OR "B-Cell Activation factor" OR "B Cell Activation factor" OR "B-Cell Activating factor" OR "B Cell Activating factor" OR bcl10 OR bcl11 OR bcl11b OR bcli10 OR blm OR blnk OR "B Cell linker" OR bloc1s6 OR "biogenesis of lysosomal organelles complex 1" OR brca1 OR brca2 OR brip1 OR btk OR "B Cell linker" OR "Bruton tyrosine kinase" OR c1qa OR c1qb OR c1qc OR "Complement C1q" OR "Complement C1s" OR "Complement activating enzyme" OR "Complement system protein" OR c1r OR c1s OR c2 OR c3 OR c4 OR c4a OR c4b OR c5 OR c6 OR c7 OR c8 OR c8a OR c8b OR c8g OR c9 OR card11 OR "Caspase recruitment domain" OR card14 OR card15 OR card9 OR carmil1 OR carmil2 OR casp8 OR "Caspase Eight" OR "Caspase 8" OR "Caspase-8" OR casp10 OR "Caspase ten" OR "Caspase 10" OR "Caspase-10" OR ccbe1 OR "calcium-binding EGF domains" OR "calcium binding EGF domains" OR cd16 OR cd19 OR cd20 OR cd21 OR cd25 OR cd27 OR cd3* OR cd3d OR cd3e OR cd3g OR cd3z OR cd40 OR cd40lg OR cd45 OR cd46 OR cd55 OR cd59 OR cd70 OR cd79a OR cd79b OR cd79 OR cd8 OR cd81 OR cd8a OR cdca7 OR cebpe OR "C-EBP epsilon" OR "CEBP epsilon" OR "C EBP epsilon" OR "CCAAT enhancer binding protein" OR cfb OR cfd OR cfh OR cfhr1 OR cfhr2 OR cfhr3 OR cfhr4 OR cfhr5 OR cfi OR cfp OR cftr OR "complement factor b" OR "complement factor d" OR "complement factor h" OR "complement factor i" OR "complement factor p" OR "cystic fibrosis transmembrane conductance regulator" OR chd7 OR "chromodomain helicase DNA binding protein 7" OR cib1 OR ciita OR "class II transactivator" OR "Class II Major Histocompatibility Complex Transactivator" OR clbp OR clcn7 OR "chloride channel 7" OR clpb OR coh1 OR copa OR "COPI Coat Complex Subunit Alpha" OR "Coronin-1A" OR "Coronin 1A" OR coro1a OR csf2ra OR csf3r OR csf2rb OR "colony stimulating factor 2" OR "colony stimulating factor 3" OR ctc1 "conserved telomere maintenance component 1" OR ctla4 OR "cytotoxic T lymphocyte associated protein 4" OR ctps1 OR "ctp synthase 1" OR ctsc OR "cathepsin C" OR cxcr4 OR cxcra OR cyba OR cybb OR cybc1 OR "cytochrome b-245" OR dbr1 OR dclre1b OR dclre1c OR "DNA cross-link repair" OR "DNA cross link repair" OR def6 OR "Del1op13-p14" OR del1op13 OR dkc1 OR "Dyskerin Pseudouridine Synthase" OR dnajc21 OR dnase1l3 OR dnase2 OR dnmt3b OR "DNA methyltransferase 3B" OR "DNA methyltransferase 3 beta" OR dock2 OR dock8 OR "dedicator of cytokinesis" OR efl1 OR "euphorbia factor L1" OR elane OR "elastase neutrophil expressed" OR epg5 OR "ectopic P-granules autophagy protein 5" OR "ectopic P granules autophagy protein 5" OR erbb2ip OR ercc4 OR ercc6l2 OR extl3 OR faap24 OR fadd OR "Fas-Associated Death Domain" OR "Fas Associated Death Domain" OR fanca OR fancb OR fancc OR fancd2 OR fance OR fanf OR fanci OR fancl OR fancm OR "Fanconi Anemia Complementation Group" OR DKCA6 OR "Coats plus syndrome due to CTC1 deficiency" OR DKCX1 OR EVI1 OR "MECOM deficiency" OR DKCB2 OR DKCB1 OR DKCB6 OR DKCA4 OR DKCB5 OR "Ataxia Pancytopenia Syndrome" OR BMFS1 OR "SRP72-deficiency" OR "Coats plus syndrome due to STN1 deficiency" OR DKCA1 OR DKCA2 OR DKCB4 OR DKCA3 OR BMFS5 OR DKCB3 OR fas OR faslg OR fat4 OR fcgr3a OR "Fc gamma receptor" OR fcho1 OR fcn3 OR "ficolin 3" OR fremt1 OR "fermitin 1" OR fermt3 OR "fermitin 3" OR foxn1 OR foxp3 OR "forkhead box P3" OR fpr1 OR "Formyl Peptide Receptor 1" OR g6pc3 OR "Glucosephosphate Dehydrogenase" OR "glucose 6 phosphatase catalytic" OR g6pt1 OR "gata-2" OR "gata 2" OR gata2 OR "gata binding protein 2" OR gfi1 OR "growth factor independent 1" OR gins1 OR havcr2 OR hax1 OR "HS1-associated protein X-1" OR "HS1 associated protein X 1" OR "HS1 associated protein X1" OR "HCLS1-associated protein X-1" OR " HCLS1 associated protein X 1" OR " HCLS1 associated protein X1" OR hells OR homx OR hmox1 OR hyou1 OR icos OR "Inducible T-Cell Co-Stimulator" OR "Inducible T Cell Costimulator" OR icoslg OR ifih1 OR "Interferon Induced Helicase c domain" OR ifnar1 OR ifnar2 OR ifngr1 OR ifngr2 OR ifnyr1 OR ifnyr2 OR "interferon gamma receptor" OR "inf gamma r1" OR "inf gamma r2" OR ighm OR igkc OR "immunoglobulin kappa constant" OR igll1 OR "immunoglobulin lambda-like polypeptide 1" OR "immunoglobulin lambda like polypeptide 1" OR ikbkb OR ikbkg OR "I kappa b kinase" OR "inhibitor of nuclear factor b kinase" OR ikzf1 OR il10 OR "IL-10" OR "IL 10" OR "interleukin 10" OR "interleukin-10" OR il10ra OR "IL-10RA" OR "IL 10RA" OR "interleukin 10RA" OR "interleukin-10RA" OR il10rb OR "IL-10RB" OR "IL 10RB" OR "interleukin 10RB" OR "interleukin-10RB" OR il12r OR "IL-12R" OR "IL 12R" OR "interleukin 12R" OR "interleukin-12R" OR il12rb OR "IL-12RB" OR "IL 12RB" OR "interleukin 12RB" OR "interleukin-12RB" OR il12rb1 OR "IL-12RB1" OR "IL 12RB1" OR "interleukin 12RB1" OR "interleukin-12RB1" OR il12rb2 OR "IL-12RB2" OR "IL 12RB2" OR "interleukin 12RB2" OR "interleukin-12RB2" OR il17f OR "IL-17F" OR "IL 17F" OR "interleukin 17F" OR "interleukin-17F" OR il17ra OR "IL-17RA" OR "IL 17RA" OR "interleukin 17RA" OR "interleukin-17RA" OR il17rc OR "IL-17RC" OR "IL 17RC" OR "interleukin 17RC" OR "interleukin-17RC" OR il18bp OR "IL-18BP" OR "IL 18BP" OR "interleukin 18BP" OR "interleukin-18BP" OR il1rn OR "IL-1RN" OR "IL 1RN" OR "interleukin 1RN" OR "interleukin-1RN" OR il23r OR "IL-23R" OR "IL 23R" OR "interleukin 23R" OR "interleukin-23R" OR il2i OR "IL-2I" OR "IL 2I" OR "interleukin 2I" OR "interleukin-2I" OR il2ir OR "IL-2IR" OR "IL 2IR" OR "interleukin 2IR" OR "interleukin-2IR" OR il2ira OR "IL-2IRA" OR "IL 2IRA" OR "interleukin 2IRA" OR "interleukin-2IRA" OR il2irb OR "IL-2IRB" OR "IL 2IRB" OR "interleukin 2IRB" OR "interleukin-2IRB" OR il2irg OR "IL-2IRG" OR "IL 2IRG" OR "interleukin 2IRG" OR "interleukin-2IRG" OR il36rn OR "IL-36RN" OR "IL 36RN" OR "interleukin 36RN" OR "interleukin-36RN" OR il6r OR "IL-6R" OR "IL 6R" OR "interleukin 6R" OR "interleukin-6R" OR il6st OR "IL-6ST" OR "IL 6ST" OR "interleukin 6ST" OR "interleukin-6ST" OR ilr7 OR "IL-R7" OR "IL R7" OR "interleukin R7" OR "interleukin- R7" OR ino80 OR irak1 OR "IRAK-1" OR "IRAK 1" OR "interleukin 1" OR "interleukin-1" OR irak4 OR "IRAK-4" OR "IRAK 4" OR "interleukin 4" OR "interleukin-4" OR irf2bp2 OR irf3 OR irf4 OR irf7 OR irf8 OR irf9 OR "interferon regulatory factor 2" OR "interferon regulatory factor 3" OR "interferon regulatory factor 4" OR "interferon regulatory factor 7" OR "interferon regulatory factor 8" OR "interferon regulatory factor 9" OR isg15 OR "itchy homolog e3" OR "itchy e3" OR itgam OR "integrin subunit alpha" OR "integrin subunit beta" OR itgb2 OR itk OR "il2 inducible t cell kinase" OR jagn1 OR "jagunal homolg 1" OR jak1 OR jak3 OR "janus kinase 1" OR "janus kinase 3" OR kdm6a OR kindlin3 OR kmt2a OR kmt2d OR kras OR lamtor2 OR lat OR lck OR "leukocyte c terminal src kinase" OR "leukocyte c-terminal src kinase" OR lig1 OR "ligase I" OR lig4 OR "ligase IV" OR lpin2 OR "lipin 2" OR lrba OR "lipopolysaccharide responsive beige like anchor protein " OR "lps responsive beige like anchor protein " OR lyst OR "lysosomal trafficking regulator" OR mad2l2 OR magt1 OR "magnesium transporter 1" OR malt1 OR "Mannan Binding Lectin" OR "Mannose Binding Lectin" OR map3k14 OR "mitogen-activated protein kinase 14" OR masp2 OR "MBL-associated serine protease 2" OR "MBL associated serine protease 2" OR mbs1 OR mcm4 OR "minichromosome maintenance complex component 4" OR mefv OR mkl1 OR mogs OR "mannosyl-oligosaccharide glucosidase" OR "mannosyl oligosaccharide glucosidase" OR msh6 OR "muts homolog 6" OR msn OR moesin OR mst1 OR "macrophage stimulating 1" OR mthfd1 OR "methylenetetrahydrofolate dehydrogenase" OR mvk OR "Mevalonate Kinase" OR myd88 OR mysm1 OR nbas OR nbs1 OR ncf1 OR ncf2 OR ncf4 OR "neutrophil cytosolic factor 1" OR "neutrophil cytosolic factor 2" OR "neutrophil cytosolic factor 4" OR ncstn OR nicastrin OR nfat5 OR "nuclear factor of activated T-cells 5" OR "nuclear factor of activated T cells 5" OR nfe2l2 OR nfkb1 OR nfkb2 OR "nuclear factor kappa b subunit 1" OR "nuclear factor kappa b subunit 2" OR nfkbia OR "nfkb inhibitor alpha" OR nhej1 OR "nonhomologous end-joining factor 1" OR "nonhomologous end joining factor 1" OR nik OR "nfkb inducing kinase" OR "nf-kb inducing kinase" OR "nf kb inducing kinase" OR nlrc4 OR "nlf family CARD domain containing 4" OR nlrp1 OR nlrp12 OR nlrp3 OR "nlr family pyrin domain containing 12" OR "nlr family pyrin domain containing 3" OR "nlr family pyrin domain containing 1" OR nod2 OR "Nucleotide Binding Oligomerization Domain Containing 2" OR nola2 OR nola3 OR "nucleolar protein family A member 2" OR "nucleolar protein family A member 3" OR nras OR nsmce3 OR oas1 OR "ORAI-I" OR "ORAI I" OR ostm1 OR otulin OR ox40 OR "tumor necrosis factor superfamily member 4 " OR "TNF receptor superfamily member 4 " OR "TNF superfamily member 4" OR p22phox OR p40phox OR p47phox OR p67phox OR "Neutrophil cytosol factor P22" OR "Neutrophil cytosol factor P40" OR "Neutrophil cytosol factor P47" OR "Neutrophil cytosol factor P67" OR palb2 OR parn OR "poly A-specific ribonuclease" OR "poly A specific ribonuclease" OR "polyA specific ribonuclease" OR pepd OR pgm3 OR "Phosphoglucomutase 3" OR pi3kr1 OR "Phosphoinositide-3-Kinase Regulatory Subunit 1" OR "Phosphoinositide 3 Kinase Regulatory Subunit 1" OR pik3cd OR pik3r1 OR plcg2 OR "phospholipase C gamma 2" OR plekhm1 OR pms2 OR "PMS1 Homolog 2" OR pnp OR "Purine Nucleoside Phosphorylase" OR pola1 OR pold1 OR pold2 OR pole1 OR pole2 OR "DNA polymerase epsilon" OR polr31 OR polr3c OR polr3f OR prf1 OR "perforin 1" OR "PRKC Delta" OR "protein kinase c delta" OR prkcd OR prkcdc OR psen OR psenen OR psmb8 OR "Proteasome 20S Subunit Beta 8" OR psmg2 OR pstpip1 OR "Proline Serine Threonine Phosphatase Interacting Protein 1 " OR pten OR ptprc OR rab27a OR rac2 OR "Rac 2" OR "Rac Family Small GTPase 2" OR rad51 OR rag OR rag1 OR rag2 OR "recombination activating gene 1" OR "recombination activating 1" OR "recombination activating gene 2" OR "recombination activating 2" OR ranbp2 OR rand51c OR rasgrp1 OR rbck1 OR "RANBP2-type" OR "RANBP2 type" OR recql3 OR "RECQ Protein-Like 3" OR "RECQ Protein Like 3" OR rel OR rela OR relb OR rfwd3 OR rfx5 OR "Regulatory Factor X5" OR rfxank OR "Regulatory Factor X Associated" OR rfxap OR rhoh OR ripk1 OR rmrp OR "RNA component of mitochondrial RNA processing endoribonuclease" OR rnaseh2a OR rnaseh2b OR rnaseh2c OR "Ribonuclease H2 Subunit A" OR "Ribonuclease H2 Subunit B" OR "Ribonuclease H2 Subunit C" OR rnf168 OR rnf31 OR "ring finger protein 168" OR "ring finger protein 31" OR rnu4atac OR robld3 OR "Roadblock domain containing 3" OR rorc OR "RAR Related Orphan Receptor C" OR rpsa OR "Ribosomal Protein SA" OR rtel1 OR "Regulator of Telomere Elongation Helicase 1" OR samd9 OR samd9l OR samhd1 OR sbds OR sec61a1 OR sema3e OR "semaphorin 3E" OR serping1 OR sh2d1a OR "SH2 Domain Containing 1A" OR sh3bp2 OR "SH3 Domain Binding Protein 2" OR sh3kbp1 OR skiv2l OR slc29a3 OR slc35c1 OR slc37a4 OR "Solute Carrier Family 29 Member 3" OR "Solute Carrier Family 35 Member C1" OR "Solute Carrier Family 37 Member A4" OR slc39a7 OR slc46a1 OR "Solute Carrier Family 39 Member A7" OR "Solute Carrier Family 46 Member A1" OR slc7a7 OR "Solute Carrier Family 7 Member A7" OR slx4 OR smarcal1 OR smarcd2 OR snx10 OR sp110 OR spink5 OR "Serine Peptidase Inhibitor Kazal Type 5" OR sppl2a OR srp54 OR srp72 OR "STAT 1" OR stat1 OR "STAT 2" OR stat2 OR "STAT 3" OR stat3 OR "STAT 5b" OR stat5b OR "STAT 5" OR stat5 OR "Signal Transducer and Activator of Transcription 5b" OR "Signal Transducer and Activator of Transcription 5" OR "Signal Transducer and Activator of Transcription 1" OR "Signal Transducer and Activator of Transcription 2" OR "Signal Transducer and Activator of Transcription 3" OR "stim-1" OR stim1 OR "Stromal Interaction Molecule 1" OR stk4 OR stn1 OR stx11 OR "Syntaxin 11" OR stxbp2 OR "syntaxin binding protein 2" OR tap1 OR tap2 OR "transporter 1" OR "transporter 2" OR tapbp OR "tap binding protein" OR taz OR tafazzin OR tbk1 OR "tank binding kinase 1" OR tbx1 OR "t box transcription factor 1" OR tcf3 OR "transcription factor 3" OR tcirg1 OR tcn2 OR "Transcobalamin 2" OR "tcr-alpha" OR "tcr alpha" OR "t cell receptor alpha" OR terc OR "Telomerase RNA Component" OR tert OR "Telomerase Reverse Transcriptase" OR tfrc OR tgfb1 OR tgfbr1 OR tgfbr2 OR thbd OR thrombomodulin OR ticam1 OR tinf2 OR "TRF1-interacting nuclear factor 2" OR "TRF1 interacting nuclear factor 2" OR "TERF1-interacting nuclear factor 2" OR "TERF1 interacting nuclear factor 2" OR thymotaxin OR tirap OR tlr3 OR "toll like receptor 3" OR tmc6 OR tmc8 OR "Transmembrane Channel Like 6" OR "Transmembrane Channel Like 8" OR tmem173 OR "Transmembrane Protein 173" OR tnfaip3 OR tnfrsf1 OR "TNF Receptor Superfamily Member" OR tnfrsf11a OR tnfrsf13b OR tnfrsf13c OR tnfrsf1a OR tnfrsf4 OR tnfrsf6 OR tnfrsf9 OR tnfsf11 OR tnfsf12 OR tnfsf6 OR top2b OR tp53 OR tpp1 OR tpp2 OR "Tripeptidyl Peptidase 1" OR "Tripeptidyl Peptidase 2" OR trac OR traf3 OR "TNF Receptor Associated Factor 3" OR traf3ip2 OR trex1 OR "Three Prime Repair Exonuclease 1" OR trim22 OR trnt1 OR "TRNA Nucleotidyl Transferase 1" OR ttc37 OR "Tetratricopeptide Repeat Domain 37" OR ttc7a OR "Tetratricopeptide Repeat Domain 7A" OR txb1 OR tyk2 OR "Tyrosine Kinase 2" OR ube2t OR unc13d OR "unc-13 homolog D" OR "unc 13 homolog D" OR unc93b1 OR "unc-93 homolog b1" OR "unc 93 homolog b1" OR ung OR "Uracil DNA Glycosylase" OR usb1 OR usp18 OR vps13b OR "Vacuolar Protein Sorting 13 Homolog B" OR vps45 OR vps45a OR "Vacuolar Protein Sorting 45 A" OR "was gene" OR "was protein" OR "WASP Actin Nucleation Promoting Factor" OR wdr1 OR wipf1 OR wrap53 OR xiap OR xrcc1 OR xrcc9 OR "ZAP-70" OR zap70 OR "Zeta Chain Of T Cell Receptor Associated Protein Kinase 70" OR zbtb24 OR "BTB Domain Containing 24" OR znf341 OR "interleukin-2 receptor subunit gamma" OR "interleukin 2 receptor subunit gamma" OR "moebius syndrome" OR "X-Linked" OR "X Linked" )  AND  TI,AB( autoimmun* OR immunodeficien* OR deficien* )  )  OR  TI,AB ( ( "inborn errors" OR "inborn error" ) AND ( immun* OR autoimmun* OR deficien* OR immunodeficien* ) )  )  AND  TI,AB,SU( ( coronavir* OR "corona virus" OR "corona pandemic" OR betacoronavir* OR covid19 OR covid OR ncov OR "CoV 2" OR cov2 OR sarscov2 OR sars2 OR 2019ncov OR "novel CoV" OR "wuhan virus" OR NCOV19 OR "solidarity trial" OR "operation warp speed" OR COVAX OR "ACT-Accelerator" OR BNT162b2 OR comirnaty OR "mRNA-1273" OR CoviShield OR AZD1222 OR "Sputnik V" OR CoronaVac OR "BBIBP-CorV" OR "Ad26.CoV2.S" OR "JNJ-78436735" OR Ad26COVS1 OR VAC31518 OR EpiVacCorona OR Convidicea OR "Ad5-nCoV" OR Covaxin OR CoviVac OR ZF2001 OR "NVX-CoV2373" OR "ZyCoV-D" OR "CIGB 66" OR CVnCoV OR "INO-4800" OR "VIR-7831" OR "UB-612" OR "BNT162" OR "Soberana 1" OR "Soberana 2 " OR "B.1.1.7" OR "VOC 202012/01" OR "VOC202012/01" OR "VUI 202012/01" OR "VUI202012/01" OR "501Y.V1" OR "UK Variant" OR "Kent Variant" OR "VOC 202102/02" OR "VOC202102/02" OR "B.1.351" OR "VOC 202012/02" OR "VOC202012/02" OR "20H/501.V2" OR "20H/501Y.V2" OR "501Y.V2" OR "501.V2" OR "South African Variant" OR "B.1.1.28.1" OR "B.1.1.28" OR "B.1.1.248" OR "VOC 202101/02" OR "VOC202101/02" OR "VUI202101/02" OR "VUI 202101/02" OR "501Y.V3" OR "brazil Variant" OR "P.1 variant" OR "P.1 lineage" OR "Lineage P.1" OR "B.1.427" OR "B.1.429" OR "20C/S:452R" OR "CAL.20C" OR "CAL.20C/L452R" OR "20C/L452R" OR "20-C variant" OR CAVUI1 OR "GH/451R.V1" OR "B.1.526" OR "20C/S:484K" OR "B.1.1.28.3" OR "P3 variant" OR "P.3 Strain" OR "Lineage P.3" OR "P.3 Lineage" OR "PHL-B.1.1.28" OR "VUI-21MAR-02" OR "B.1.1.28.2" OR "20B/S.484K" OR "P.2 variant" OR "P.2 strain" OR "P.2 Lineage" OR "GR clade" OR "VUI 202101/01" OR "VUI202101/01" OR "B.1.177" OR "20A.EU1" OR "20A.EU2" OR "Variant 20A" OR "B.1.525" OR "G/484K.V3" OR "20A/S:484K" OR UK1188 OR "B.1.616" OR "20C/ B1" OR "clade 20C" OR "B.1.617" OR "VUI-21APR-01" OR "B.1.618" OR "VUI-202102/01" OR "VUI-21FEB-01" OR D614G OR N501Y OR A570D OR P681H OR K417N OR E484K OR "K417N/T" OR L452R OR S477N OR D253G OR T951 OR A701V OR 8477H OR V30L OR A220V OR T445C OR C6286T OR C26801G OR E484L OR D66H OR Y144V OR Q677H OR D215G OR 484K OR P681R OR D651G OR E484Q OR F888L OR F565L OR V1176F OR F157S OR L452R OR D614G OR T781I OR T859N OR D950H OR L5F OR K417T OR L18F OR T20N OR P26S OR R190S OR H655Y OR T1027I OR A701V OR K417N OR S494P OR T716I OR S982A OR D1118H OR K1191N) OR (sars AND cov) OR ( ( wuhan OR hubei OR huanan ) AND ( "severe acute respiratory" OR pneumonia* ) AND ( outbreak* ) ) )  Limits: English, 2019-, peer-reviewed | 02/28/2022 | 83 articles  -31 duplicates  =52 articles |
| Wiley online library | "11q23 OR 11q23del OR acd OR ace OR "angiotensin converting enzyme" OR acp5 OR "acid phosphate 5" OR actb OR ada OR ada2 OR adam17 OR tace OR "adenosine deaminase" OR adar1 OR aicda OR "AID enzyme" OR "activation induced cytidine deaminase" OR "activation-induced cytidine deaminase" OR apeced OR "aire autoimmune regulator" OR ak2 OR "Adenylate kinase 2" OR "Adenylate kinase-2" OR alpi OR "intestinal alkaline phosphatase" OR ap1s3 OR "adaptor related protein complex 1" OR ap3b1 "adaptor related protein complex 3" OR ap3d1 OR "APOL-I" OR "APOL I" OR "Apolipoprotein L" OR arhgef1 OR arpc1b OR atm OR "ataxia telangiectasia mutated" OR atp6ap1 OR b2m OR "beta 2-Microglobulin" OR "beta 2 Microglobulin" OR bach2 OR baff OR "B-Cell Activation factor" OR "B Cell Activation factor" OR "B-Cell Activating factor" OR "B Cell Activating factor" OR bcl10 OR bcl11 OR bcl11b OR bcli10 OR blm OR blnk OR "B Cell linker" OR bloc1s6 OR "biogenesis of lysosomal organelles complex 1" OR brca1 OR brca2 OR brip1 OR btk OR "B Cell linker" OR "Bruton tyrosine kinase" OR c1qa OR c1qb OR c1qc OR "Complement C1q" OR "Complement C1s" OR "Complement activating enzyme" OR "Complement system protein" OR c1r OR c1s OR c2 OR c3 OR c4 OR c4a OR c4b OR c5 OR c6 OR c7 OR c8 OR c8a OR c8b OR c8g OR c9 OR card11 OR "Caspase recruitment domain" OR card14 OR card15 OR card9 OR carmil1 OR carmil2 OR casp8 OR "Caspase Eight" OR "Caspase 8" OR "Caspase-8" OR casp10 OR "Caspase ten" OR "Caspase 10" OR "Caspase-10" OR ccbe1 OR "calcium-binding EGF domains" OR "calcium binding EGF domains" OR cd16 OR cd19 OR cd20 OR cd21 OR cd25 OR cd27 OR cd3* OR cd3d OR cd3e OR cd3g OR cd3z OR cd40 OR cd40lg OR cd45 OR cd46 OR cd55 OR cd59 OR cd70 OR cd79a OR cd79b OR cd79 OR cd8 OR cd81 OR cd8a OR cdca7 OR cebpe OR "C-EBP epsilon" OR "CEBP epsilon" OR "C EBP epsilon" OR "CCAAT enhancer binding protein" OR cfb OR cfd OR cfh OR cfhr1 OR cfhr2 OR cfhr3 OR cfhr4 OR cfhr5 OR cfi OR cfp OR cftr OR "complement factor b" OR "complement factor d" OR "complement factor h" OR "complement factor i" OR "complement factor p" OR "cystic fibrosis transmembrane conductance regulator" OR chd7 OR "chromodomain helicase DNA binding protein 7" OR cib1 OR ciita OR "class II transactivator" OR "Class II Major Histocompatibility Complex Transactivator" OR clbp OR clcn7 OR "chloride channel 7" OR clpb OR coh1 OR copa OR "COPI Coat Complex Subunit Alpha" OR "Coronin-1A" OR "Coronin 1A" OR coro1a OR csf2ra OR csf3r OR csf2rb OR "colony stimulating factor 2" OR "colony stimulating factor 3" OR ctc1 "conserved telomere maintenance component 1" OR ctla4 OR "cytotoxic T lymphocyte associated protein 4" OR ctps1 OR "ctp synthase 1" OR ctsc OR "cathepsin C" OR cxcr4 OR cxcra OR cyba OR cybb OR cybc1 OR "cytochrome b-245" OR dbr1 OR dclre1b OR dclre1c OR "DNA cross-link repair" OR "DNA cross link repair" OR def6 OR "Del1op13-p14" OR del1op13 OR dkc1 OR "Dyskerin Pseudouridine Synthase" OR dnajc21 OR dnase1l3 OR dnase2 OR dnmt3b OR "DNA methyltransferase 3B" OR "DNA methyltransferase 3 beta" OR dock2 OR dock8 OR "dedicator of cytokinesis" OR efl1 OR "euphorbia factor L1" OR elane OR "elastase neutrophil expressed" OR epg5 OR "ectopic P-granules autophagy protein 5" OR "ectopic P granules autophagy protein 5" OR erbb2ip OR ercc4 OR ercc6l2 OR extl3 OR faap24 OR fadd OR "Fas-Associated Death Domain" OR "Fas Associated Death Domain" OR fanca OR fancb OR fancc OR fancd2 OR fance OR fanf OR fanci OR fancl OR fancm OR "Fanconi Anemia Complementation Group" OR DKCA6 OR "Coats plus syndrome due to CTC1 deficiency" OR DKCX1 OR EVI1 OR "MECOM deficiency" OR DKCB2 OR DKCB1 OR DKCB6 OR DKCA4 OR DKCB5 OR "Ataxia Pancytopenia Syndrome" OR BMFS1 OR "SRP72-deficiency" OR "Coats plus syndrome due to STN1 deficiency" OR DKCA1 OR DKCA2 OR DKCB4 OR DKCA3 OR BMFS5 OR DKCB3 OR fas OR faslg OR fat4 OR fcgr3a OR "Fc gamma receptor" OR fcho1 OR fcn3 OR "ficolin 3" OR fremt1 OR "fermitin 1" OR fermt3 OR "fermitin 3" OR foxn1 OR foxp3 OR "forkhead box P3" OR fpr1 OR "Formyl Peptide Receptor 1" OR g6pc3 OR "Glucosephosphate Dehydrogenase" OR "glucose 6 phosphatase catalytic" OR g6pt1 OR "gata-2" OR "gata 2" OR gata2 OR "gata binding protein 2" OR gfi1 OR "growth factor independent 1" OR gins1 OR havcr2 OR hax1 OR "HS1-associated protein X-1" OR "HS1 associated protein X 1" OR "HS1 associated protein X1" OR "HCLS1-associated protein X-1" OR " HCLS1 associated protein X 1" OR " HCLS1 associated protein X1" OR hells OR homx OR hmox1 OR hyou1 OR icos OR "Inducible T-Cell Co-Stimulator" OR "Inducible T Cell Costimulator" OR icoslg OR ifih1 OR "Interferon Induced Helicase c domain" OR ifnar1 OR ifnar2 OR ifngr1 OR ifngr2 OR ifnyr1 OR ifnyr2 OR "interferon gamma receptor" OR "inf gamma r1" OR "inf gamma r2" OR ighm OR igkc OR "immunoglobulin kappa constant" OR igll1 OR "immunoglobulin lambda-like polypeptide 1" OR "immunoglobulin lambda like polypeptide 1" OR ikbkb OR ikbkg OR "I kappa b kinase" OR "inhibitor of nuclear factor b kinase" OR ikzf1 OR il10 OR "IL-10" OR "IL 10" OR "interleukin 10" OR "interleukin-10" OR il10ra OR "IL-10RA" OR "IL 10RA" OR "interleukin 10RA" OR "interleukin-10RA" OR il10rb OR "IL-10RB" OR "IL 10RB" OR "interleukin 10RB" OR "interleukin-10RB" OR il12r OR "IL-12R" OR "IL 12R" OR "interleukin 12R" OR "interleukin-12R" OR il12rb OR "IL-12RB" OR "IL 12RB" OR "interleukin 12RB" OR "interleukin-12RB" OR il12rb1 OR "IL-12RB1" OR "IL 12RB1" OR "interleukin 12RB1" OR "interleukin-12RB1" OR il12rb2 OR "IL-12RB2" OR "IL 12RB2" OR "interleukin 12RB2" OR "interleukin-12RB2" OR il17f OR "IL-17F" OR "IL 17F" OR "interleukin 17F" OR "interleukin-17F" OR il17ra OR "IL-17RA" OR "IL 17RA" OR "interleukin 17RA" OR "interleukin-17RA" OR il17rc OR "IL-17RC" OR "IL 17RC" OR "interleukin 17RC" OR "interleukin-17RC" OR il18bp OR "IL-18BP" OR "IL 18BP" OR "interleukin 18BP" OR "interleukin-18BP" OR il1rn OR "IL-1RN" OR "IL 1RN" OR "interleukin 1RN" OR "interleukin-1RN" OR il23r OR "IL-23R" OR "IL 23R" OR "interleukin 23R" OR "interleukin-23R" OR il2i OR "IL-2I" OR "IL 2I" OR "interleukin 2I" OR "interleukin-2I" OR il2ir OR "IL-2IR" OR "IL 2IR" OR "interleukin 2IR" OR "interleukin-2IR" OR il2ira OR "IL-2IRA" OR "IL 2IRA" OR "interleukin 2IRA" OR "interleukin-2IRA" OR il2irb OR "IL-2IRB" OR "IL 2IRB" OR "interleukin 2IRB" OR "interleukin-2IRB" OR il2irg OR "IL-2IRG" OR "IL 2IRG" OR "interleukin 2IRG" OR "interleukin-2IRG" OR il36rn OR "IL-36RN" OR "IL 36RN" OR "interleukin 36RN" OR "interleukin-36RN" OR il6r OR "IL-6R" OR "IL 6R" OR "interleukin 6R" OR "interleukin-6R" OR il6st OR "IL-6ST" OR "IL 6ST" OR "interleukin 6ST" OR "interleukin-6ST" OR ilr7 OR "IL-R7" OR "IL R7" OR "interleukin R7" OR "interleukin- R7" OR ino80 OR irak1 OR "IRAK-1" OR "IRAK 1" OR "interleukin 1" OR "interleukin-1" OR irak4 OR "IRAK-4" OR "IRAK 4" OR "interleukin 4" OR "interleukin-4" OR irf2bp2 OR irf3 OR irf4 OR irf7 OR irf8 OR irf9 OR "interferon regulatory factor 2" OR "interferon regulatory factor 3" OR "interferon regulatory factor 4" OR "interferon regulatory factor 7" OR "interferon regulatory factor 8" OR "interferon regulatory factor 9" OR isg15 OR "itchy homolog e3" OR "itchy e3" OR itgam OR "integrin subunit alpha" OR "integrin subunit beta" OR itgb2 OR itk OR "il2 inducible t cell kinase" OR jagn1 OR "jagunal homolg 1" OR jak1 OR jak3 OR "janus kinase 1" OR "janus kinase 3" OR kdm6a OR kindlin3 OR kmt2a OR kmt2d OR kras OR lamtor2 OR lat OR lck OR "leukocyte c terminal src kinase" OR "leukocyte c-terminal src kinase" OR lig1 OR "ligase I" OR lig4 OR "ligase IV" OR lpin2 OR "lipin 2" OR lrba OR "lipopolysaccharide responsive beige like anchor protein " OR "lps responsive beige like anchor protein " OR lyst OR "lysosomal trafficking regulator" OR mad2l2 OR magt1 OR "magnesium transporter 1" OR malt1 OR "Mannan Binding Lectin" OR "Mannose Binding Lectin" OR map3k14 OR "mitogen-activated protein kinase 14" OR masp2 OR "MBL-associated serine protease 2" OR "MBL associated serine protease 2" OR mbs1 OR mcm4 OR "minichromosome maintenance complex component 4" OR mefv OR mkl1 OR mogs OR "mannosyl-oligosaccharide glucosidase" OR "mannosyl oligosaccharide glucosidase" OR msh6 OR "muts homolog 6" OR msn OR moesin OR mst1 OR "macrophage stimulating 1" OR mthfd1 OR "methylenetetrahydrofolate dehydrogenase" OR mvk OR "Mevalonate Kinase" OR myd88 OR mysm1 OR nbas OR nbs1 OR ncf1 OR ncf2 OR ncf4 OR "neutrophil cytosolic factor 1" OR "neutrophil cytosolic factor 2" OR "neutrophil cytosolic factor 4" OR ncstn OR nicastrin OR nfat5 OR "nuclear factor of activated T-cells 5" OR "nuclear factor of activated T cells 5" OR nfe2l2 OR nfkb1 OR nfkb2 OR "nuclear factor kappa b subunit 1" OR "nuclear factor kappa b subunit 2" OR nfkbia OR "nfkb inhibitor alpha" OR nhej1 OR "nonhomologous end-joining factor 1" OR "nonhomologous end joining factor 1" OR nik OR "nfkb inducing kinase" OR "nf-kb inducing kinase" OR "nf kb inducing kinase" OR nlrc4 OR "nlf family CARD domain containing 4" OR nlrp1 OR nlrp12 OR nlrp3 OR "nlr family pyrin domain containing 12" OR "nlr family pyrin domain containing 3" OR "nlr family pyrin domain containing 1" OR nod2 OR "Nucleotide Binding Oligomerization Domain Containing 2" OR nola2 OR nola3 OR "nucleolar protein family A member 2" OR "nucleolar protein family A member 3" OR nras OR nsmce3 OR oas1 OR "ORAI-I" OR "ORAI I" OR ostm1 OR otulin OR ox40 OR "tumor necrosis factor superfamily member 4 " OR "TNF receptor superfamily member 4 " OR "TNF superfamily member 4" OR p22phox OR p40phox OR p47phox OR p67phox OR "Neutrophil cytosol factor P22" OR "Neutrophil cytosol factor P40" OR "Neutrophil cytosol factor P47" OR "Neutrophil cytosol factor P67" OR palb2 OR parn OR "poly A-specific ribonuclease" OR "poly A specific ribonuclease" OR "polyA specific ribonuclease" OR pepd OR pgm3 OR "Phosphoglucomutase 3" OR pi3kr1 OR "Phosphoinositide-3-Kinase Regulatory Subunit 1" OR "Phosphoinositide 3 Kinase Regulatory Subunit 1" OR pik3cd OR pik3r1 OR plcg2 OR "phospholipase C gamma 2" OR plekhm1 OR pms2 OR "PMS1 Homolog 2" OR pnp OR "Purine Nucleoside Phosphorylase" OR pola1 OR pold1 OR pold2 OR pole1 OR pole2 OR "DNA polymerase epsilon" OR polr31 OR polr3c OR polr3f OR prf1 OR "perforin 1" OR "PRKC Delta" OR "protein kinase c delta" OR prkcd OR prkcdc OR psen OR psenen OR psmb8 OR "Proteasome 20S Subunit Beta 8" OR psmg2 OR pstpip1 OR "Proline Serine Threonine Phosphatase Interacting Protein 1 " OR pten OR ptprc OR rab27a OR rac2 OR "Rac 2" OR "Rac Family Small GTPase 2" OR rad51 OR rag OR rag1 OR rag2 OR "recombination activating gene 1" OR "recombination activating 1" OR "recombination activating gene 2" OR "recombination activating 2" OR ranbp2 OR rand51c OR rasgrp1 OR rbck1 OR "RANBP2-type" OR "RANBP2 type" OR recql3 OR "RECQ Protein-Like 3" OR "RECQ Protein Like 3" OR rel OR rela OR relb OR rfwd3 OR rfx5 OR "Regulatory Factor X5" OR rfxank OR "Regulatory Factor X Associated" OR rfxap OR rhoh OR ripk1 OR rmrp OR "RNA component of mitochondrial RNA processing endoribonuclease" OR rnaseh2a OR rnaseh2b OR rnaseh2c OR "Ribonuclease H2 Subunit A" OR "Ribonuclease H2 Subunit B" OR "Ribonuclease H2 Subunit C" OR rnf168 OR rnf31 OR "ring finger protein 168" OR "ring finger protein 31" OR rnu4atac OR robld3 OR "Roadblock domain containing 3" OR rorc OR "RAR Related Orphan Receptor C" OR rpsa OR "Ribosomal Protein SA" OR rtel1 OR "Regulator of Telomere Elongation Helicase 1" OR samd9 OR samd9l OR samhd1 OR sbds OR sec61a1 OR sema3e OR "semaphorin 3E" OR serping1 OR sh2d1a OR "SH2 Domain Containing 1A" OR sh3bp2 OR "SH3 Domain Binding Protein 2" OR sh3kbp1 OR skiv2l OR slc29a3 OR slc35c1 OR slc37a4 OR "Solute Carrier Family 29 Member 3" OR "Solute Carrier Family 35 Member C1" OR "Solute Carrier Family 37 Member A4" OR slc39a7 OR slc46a1 OR "Solute Carrier Family 39 Member A7" OR "Solute Carrier Family 46 Member A1" OR slc7a7 OR "Solute Carrier Family 7 Member A7" OR slx4 OR smarcal1 OR smarcd2 OR snx10 OR sp110 OR spink5 OR "Serine Peptidase Inhibitor Kazal Type 5" OR sppl2a OR srp54 OR srp72 OR "STAT 1" OR stat1 OR "STAT 2" OR stat2 OR "STAT 3" OR stat3 OR "STAT 5b" OR stat5b OR "STAT 5" OR stat5 OR "Signal Transducer and Activator of Transcription 5b" OR "Signal Transducer and Activator of Transcription 5" OR "Signal Transducer and Activator of Transcription 1" OR "Signal Transducer and Activator of Transcription 2" OR "Signal Transducer and Activator of Transcription 3" OR "stim-1" OR stim1 OR "Stromal Interaction Molecule 1" OR stk4 OR stn1 OR stx11 OR "Syntaxin 11" OR stxbp2 OR "syntaxin binding protein 2" OR tap1 OR tap2 OR "transporter 1" OR "transporter 2" OR tapbp OR "tap binding protein" OR taz OR tafazzin OR tbk1 OR "tank binding kinase 1" OR tbx1 OR "t box transcription factor 1" OR tcf3 OR "transcription factor 3" OR tcirg1 OR tcn2 OR "Transcobalamin 2" OR "tcr-alpha" OR "tcr alpha" OR "t cell receptor alpha" OR terc OR "Telomerase RNA Component" OR tert OR "Telomerase Reverse Transcriptase" OR tfrc OR tgfb1 OR tgfbr1 OR tgfbr2 OR thbd OR thrombomodulin OR ticam1 OR tinf2 OR "TRF1-interacting nuclear factor 2" OR "TRF1 interacting nuclear factor 2" OR "TERF1-interacting nuclear factor 2" OR "TERF1 interacting nuclear factor 2" OR thymotaxin OR tirap OR tlr3 OR "toll like receptor 3" OR tmc6 OR tmc8 OR "Transmembrane Channel Like 6" OR "Transmembrane Channel Like 8" OR tmem173 OR "Transmembrane Protein 173" OR tnfaip3 OR tnfrsf1 OR "TNF Receptor Superfamily Member" OR tnfrsf11a OR tnfrsf13b OR tnfrsf13c OR tnfrsf1a OR tnfrsf4 OR tnfrsf6 OR tnfrsf9 OR tnfsf11 OR tnfsf12 OR tnfsf6 OR top2b OR tp53 OR tpp1 OR tpp2 OR "Tripeptidyl Peptidase 1" OR "Tripeptidyl Peptidase 2" OR trac OR traf3 OR "TNF Receptor Associated Factor 3" OR traf3ip2 OR trex1 OR "Three Prime Repair Exonuclease 1" OR trim22 OR trnt1 OR "TRNA Nucleotidyl Transferase 1" OR ttc37 OR "Tetratricopeptide Repeat Domain 37" OR ttc7a OR "Tetratricopeptide Repeat Domain 7A" OR txb1 OR tyk2 OR "Tyrosine Kinase 2" OR ube2t OR unc13d OR "unc-13 homolog D" OR "unc 13 homolog D" OR unc93b1 OR "unc-93 homolog b1" OR "unc 93 homolog b1" OR ung OR "Uracil DNA Glycosylase" OR usb1 OR usp18 OR vps13b OR "Vacuolar Protein Sorting 13 Homolog B" OR vps45 OR vps45a OR "Vacuolar Protein Sorting 45 A" OR "was gene" OR "was protein" OR "WASP Actin Nucleation Promoting Factor" OR wdr1 OR wipf1 OR wrap53 OR xiap OR xrcc1 OR xrcc9 OR "ZAP-70" OR zap70 OR "Zeta Chain Of T Cell Receptor Associated Protein Kinase 70" OR zbtb24 OR "BTB Domain Containing 24" OR znf341 OR "interleukin-2 receptor subunit gamma" OR "interleukin 2 receptor subunit gamma" OR "moebius syndrome" OR "X-Linked" OR "X Linked"" anywhere and "autoimmun* OR immunodeficien* OR deficien*" anywhere or ""inborn errors" OR "inborn error" AND immun* OR autoimmun* OR deficien* OR immunodeficien*" anywhere and "coronavir* OR "corona virus" OR "corona pandemic" OR betacoronavir* OR covid19 OR covid OR ncov OR "CoV 2" OR cov2 OR sarscov2 OR sars2 OR 2019ncov OR "novel CoV" OR "wuhan virus" OR NCOV19 OR "solidarity trial" OR "operation warp speed" OR COVAX OR "ACT-Accelerator" OR BNT162b2 OR comirnaty OR "mRNA-1273" OR CoviShield OR AZD1222 OR "Sputnik V" OR CoronaVac OR "BBIBP-CorV" OR "Ad26.CoV2.S" OR "JNJ-78436735" OR Ad26COVS1 OR VAC31518 OR EpiVacCorona OR Convidicea OR "Ad5-nCoV" OR Covaxin OR CoviVac OR ZF2001 OR "NVX-CoV2373" OR "ZyCoV-D" OR "CIGB 66" OR CVnCoV OR "INO-4800" OR "VIR-7831" OR "UB-612" OR "BNT162" OR "Soberana 1" OR "Soberana 2 " OR "B.1.1.7" OR "VOC 202012/01" OR "VOC202012/01" OR "VUI 202012/01" OR "VUI202012/01" OR "501Y.V1" OR "UK Variant" OR "Kent Variant" OR "VOC 202102/02" OR "VOC202102/02" OR "B.1.351" OR "VOC 202012/02" OR "VOC202012/02" OR "20H/501.V2" OR "20H/501Y.V2" OR "501Y.V2" OR "501.V2" OR "South African Variant" OR "B.1.1.28.1" OR "B.1.1.28" OR "B.1.1.248" OR "VOC 202101/02" OR "VOC202101/02" OR "VUI202101/02" OR "VUI 202101/02" OR "501Y.V3" OR "brazil Variant" OR "P.1 variant" OR "P.1 lineage" OR "Lineage P.1" OR "B.1.427" OR "B.1.429" OR "20C/S:452R" OR "CAL.20C" OR "CAL.20C/L452R" OR "20C/L452R" OR "20-C variant" OR CAVUI1 OR "GH/451R.V1" OR "B.1.526" OR "20C/S:484K" OR "B.1.1.28.3" OR "P3 variant" OR "P.3 Strain" OR "Lineage P.3" OR "P.3 Lineage" OR "PHL-B.1.1.28" OR "VUI-21MAR-02" OR "B.1.1.28.2" OR "20B/S.484K" OR "P.2 variant" OR "P.2 strain" OR "P.2 Lineage" OR "GR clade" OR "VUI 202101/01" OR "VUI202101/01" OR "B.1.177" OR "20A.EU1" OR "20A.EU2" OR "Variant 20A" OR "B.1.525" OR "G/484K.V3" OR "20A/S:484K" OR UK1188 OR "B.1.616" OR "20C/ B1" OR "clade 20C" OR "B.1.617" OR "VUI-21APR-01" OR "B.1.618" OR "VUI-202102/01" OR "VUI-21FEB-01" OR D614G OR N501Y OR A570D OR P681H OR K417N OR E484K OR "K417N/T" OR L452R OR S477N OR D253G OR T951 OR A701V OR 8477H OR V30L OR A220V OR T445C OR C6286T OR C26801G OR E484L OR D66H OR Y144V OR Q677H OR D215G OR 484K OR P681R OR D651G OR E484Q OR F888L OR F565L OR V1176F OR F157S OR L452R OR D614G OR T781I OR T859N OR D950H OR L5F OR K417T OR L18F OR T20N OR P26S OR R190S OR H655Y OR T1027I OR A701V OR K417N OR S494P OR T716I OR S982A OR D1118H OR K1191N" anywhere or "sars AND cov" anywhere or "wuhan OR hubei OR huanan" anywhere and ""severe acute respiratory" OR pneumonia*" anywhere and "outbreak*"  Limits: 2019- | 02/28/2022 | 38 articles  -17 duplicates  =21 articles |
| Scopus | ( ( ( TITLE-ABS-KEY ( 11q23 OR 11q23del OR acd OR ace OR "angiotensin converting enzyme" OR acp5 OR "acid phosphate 5" OR actb OR ada OR ada2 OR adam17 OR tace OR "adenosine deaminase" OR adar1 OR aicda OR "AID enzyme" OR "activation induced cytidine deaminase" OR "activation-induced cytidine deaminase" OR apeced OR "aire autoimmune regulator" OR ak2 OR "Adenylate kinase 2" OR "Adenylate kinase-2" OR alpi OR "intestinal alkaline phosphatase" OR ap1s3 OR "adaptor related protein complex 1" OR ap3b1 "adaptor related protein complex 3" OR ap3d1 OR "APOL-I" OR "APOL I" OR "Apolipoprotein L" OR arhgef1 OR arpc1b OR atm OR "ataxia telangiectasia mutated" OR atp6ap1 OR b2m OR "beta 2-Microglobulin" OR "beta 2 Microglobulin" OR bach2 OR baff OR "B-Cell Activation factor" OR "B Cell Activation factor" OR "B-Cell Activating factor" OR "B Cell Activating factor" OR bcl10 OR bcl11 OR bcl11b OR bcli10 OR blm OR blnk OR "B Cell linker" OR bloc1s6 OR "biogenesis of lysosomal organelles complex 1" OR brca1 OR brca2 OR brip1 OR btk OR "B Cell linker" OR "Bruton tyrosine kinase" OR c1qa OR c1qb OR c1qc OR "Complement C1q" OR "Complement C1s" OR "Complement activating enzyme" OR "Complement system protein" OR c1r OR c1s OR c2 OR c3 OR c4 OR c4a OR c4b OR c5 OR c6 OR c7 OR c8 OR c8a OR c8b OR c8g OR c9 OR card11 OR "Caspase recruitment domain" OR card14 OR card15 OR card9 OR carmil1 OR carmil2 OR casp8 OR "Caspase Eight" OR "Caspase 8" OR "Caspase-8" OR casp10 OR "Caspase ten" OR "Caspase 10" OR "Caspase-10" OR ccbe1 OR "calcium-binding EGF domains" OR "calcium binding EGF domains" OR cd16 OR cd19 OR cd20 OR cd21 OR cd25 OR cd27 OR cd3* OR cd3d OR cd3e OR cd3g OR cd3z OR cd40 OR cd40lg OR cd45 OR cd46 OR cd55 OR cd59 OR cd70 OR cd79a OR cd79b OR cd79 OR cd8 OR cd81 OR cd8a OR cdca7 OR cebpe OR "C-EBP epsilon" OR "CEBP epsilon" OR "C EBP epsilon" OR "CCAAT enhancer binding protein" OR cfb OR cfd OR cfh OR cfhr1 OR cfhr2 OR cfhr3 OR cfhr4 OR cfhr5 OR cfi OR cfp OR cftr OR "complement factor b" OR "complement factor d" OR "complement factor h" OR "complement factor i" OR "complement factor p" OR "cystic fibrosis transmembrane conductance regulator" OR chd7 OR "chromodomain helicase DNA binding protein 7" OR cib1 OR ciita OR "class II transactivator" OR "Class II Major Histocompatibility Complex Transactivator" OR clbp OR clcn7 OR "chloride channel 7" OR clpb OR coh1 OR copa OR "COPI Coat Complex Subunit Alpha" OR "Coronin-1A" OR "Coronin 1A" OR coro1a OR csf2ra OR csf3r OR csf2rb OR "colony stimulating factor 2" OR "colony stimulating factor 3" OR ctc1 "conserved telomere maintenance component 1" OR ctla4 OR "cytotoxic T lymphocyte associated protein 4" OR ctps1 OR "ctp synthase 1" OR ctsc OR "cathepsin C" OR cxcr4 OR cxcra OR cyba OR cybb OR cybc1 OR "cytochrome b-245" OR dbr1 OR dclre1b OR dclre1c OR "DNA cross-link repair" OR "DNA cross link repair" OR def6 OR "Del1op13-p14" OR del1op13 OR dkc1 OR "Dyskerin Pseudouridine Synthase" OR dnajc21 OR dnase1l3 OR dnase2 OR dnmt3b OR "DNA methyltransferase 3B" OR "DNA methyltransferase 3 beta" OR dock2 OR dock8 OR "dedicator of cytokinesis" OR efl1 OR "euphorbia factor L1" OR elane OR "elastase neutrophil expressed" OR epg5 OR "ectopic P-granules autophagy protein 5" OR "ectopic P granules autophagy protein 5" OR erbb2ip OR ercc4 OR ercc6l2 OR extl3 OR faap24 OR fadd OR "Fas-Associated Death Domain" OR "Fas Associated Death Domain" OR fanca OR fancb OR fancc OR fancd2 OR fance OR fanf OR fanci OR fancl OR fancm OR "Fanconi Anemia Complementation Group" OR DKCA6 OR "Coats plus syndrome due to CTC1 deficiency" OR DKCX1 OR EVI1 OR "MECOM deficiency" OR DKCB2 OR DKCB1 OR DKCB6 OR DKCA4 OR DKCB5 OR "Ataxia Pancytopenia Syndrome" OR BMFS1 OR "SRP72-deficiency" OR "Coats plus syndrome due to STN1 deficiency" OR DKCA1 OR DKCA2 OR DKCB4 OR DKCA3 OR BMFS5 OR DKCB3 OR fas OR faslg OR fat4 OR fcgr3a OR "Fc gamma receptor" OR fcho1 OR fcn3 OR "ficolin 3" OR fremt1 OR "fermitin 1" OR fermt3 OR "fermitin 3" OR foxn1 OR foxp3 OR "forkhead box P3" OR fpr1 OR "Formyl Peptide Receptor 1" OR g6pc3 OR "Glucosephosphate Dehydrogenase" OR "glucose 6 phosphatase catalytic" OR g6pt1 OR "gata-2" OR "gata 2" OR gata2 OR "gata binding protein 2" OR gfi1 OR "growth factor independent 1" OR gins1 OR havcr2 OR hax1 OR "HS1-associated protein X-1" OR "HS1 associated protein X 1" OR "HS1 associated protein X1" OR "HCLS1-associated protein X-1" OR " HCLS1 associated protein X 1" OR " HCLS1 associated protein X1" OR hells OR homx OR hmox1 OR hyou1 OR icos OR "Inducible T-Cell Co-Stimulator" OR "Inducible T Cell Costimulator" OR icoslg OR ifih1 OR "Interferon Induced Helicase c domain" OR ifnar1 OR ifnar2 OR ifngr1 OR ifngr2 OR ifnyr1 OR ifnyr2 OR "interferon gamma receptor" OR "inf gamma r1" OR "inf gamma r2" OR ighm OR igkc OR "immunoglobulin kappa constant" OR igll1 OR "immunoglobulin lambda-like polypeptide 1" OR "immunoglobulin lambda like polypeptide 1" OR ikbkb OR ikbkg OR "I kappa b kinase" OR "inhibitor of nuclear factor b kinase" OR ikzf1 OR il10 OR "IL-10" OR "IL 10" OR "interleukin 10" OR "interleukin-10" OR il10ra OR "IL-10RA" OR "IL 10RA" OR "interleukin 10RA" OR "interleukin-10RA" OR il10rb OR "IL-10RB" OR "IL 10RB" OR "interleukin 10RB" OR "interleukin-10RB" OR il12r OR "IL-12R" OR "IL 12R" OR "interleukin 12R" OR "interleukin-12R" OR il12rb OR "IL-12RB" OR "IL 12RB" OR "interleukin 12RB" OR "interleukin-12RB" OR il12rb1 OR "IL-12RB1" OR "IL 12RB1" OR "interleukin 12RB1" OR "interleukin-12RB1" OR il12rb2 OR "IL-12RB2" OR "IL 12RB2" OR "interleukin 12RB2" OR "interleukin-12RB2" OR il17f OR "IL-17F" OR "IL 17F" OR "interleukin 17F" OR "interleukin-17F" OR il17ra OR "IL-17RA" OR "IL 17RA" OR "interleukin 17RA" OR "interleukin-17RA" OR il17rc OR "IL-17RC" OR "IL 17RC" OR "interleukin 17RC" OR "interleukin-17RC" OR il18bp OR "IL-18BP" OR "IL 18BP" OR "interleukin 18BP" OR "interleukin-18BP" OR il1rn OR "IL-1RN" OR "IL 1RN" OR "interleukin 1RN" OR "interleukin-1RN" OR il23r OR "IL-23R" OR "IL 23R" OR "interleukin 23R" OR "interleukin-23R" OR il2i OR "IL-2I" OR "IL 2I" OR "interleukin 2I" OR "interleukin-2I" OR il2ir OR "IL-2IR" OR "IL 2IR" OR "interleukin 2IR" OR "interleukin-2IR" OR il2ira OR "IL-2IRA" OR "IL 2IRA" OR "interleukin 2IRA" OR "interleukin-2IRA" OR il2irb OR "IL-2IRB" OR "IL 2IRB" OR "interleukin 2IRB" OR "interleukin-2IRB" OR il2irg OR "IL-2IRG" OR "IL 2IRG" OR "interleukin 2IRG" OR "interleukin-2IRG" OR il36rn OR "IL-36RN" OR "IL 36RN" OR "interleukin 36RN" OR "interleukin-36RN" OR il6r OR "IL-6R" OR "IL 6R" OR "interleukin 6R" OR "interleukin-6R" OR il6st OR "IL-6ST" OR "IL 6ST" OR "interleukin 6ST" OR "interleukin-6ST" OR ilr7 OR "IL-R7" OR "IL R7" OR "interleukin R7" OR "interleukin- R7" OR ino80 OR irak1 OR "IRAK-1" OR "IRAK 1" OR "interleukin 1" OR "interleukin-1" OR irak4 OR "IRAK-4" OR "IRAK 4" OR "interleukin 4" OR "interleukin-4" OR irf2bp2 OR irf3 OR irf4 OR irf7 OR irf8 OR irf9 OR "interferon regulatory factor 2" OR "interferon regulatory factor 3" OR "interferon regulatory factor 4" OR "interferon regulatory factor 7" OR "interferon regulatory factor 8" OR "interferon regulatory factor 9" OR isg15 OR "itchy homolog e3" OR "itchy e3" OR itgam OR "integrin subunit alpha" OR "integrin subunit beta" OR itgb2 OR itk OR "il2 inducible t cell kinase" OR jagn1 OR "jagunal homolg 1" OR jak1 OR jak3 OR "janus kinase 1" OR "janus kinase 3" OR kdm6a OR kindlin3 OR kmt2a OR kmt2d OR kras OR lamtor2 OR lat OR lck OR "leukocyte c terminal src kinase" OR "leukocyte c-terminal src kinase" OR lig1 OR "ligase I" OR lig4 OR "ligase IV" OR lpin2 OR "lipin 2" OR lrba OR "lipopolysaccharide responsive beige like anchor protein " OR "lps responsive beige like anchor protein " OR lyst OR "lysosomal trafficking regulator" OR mad2l2 OR magt1 OR "magnesium transporter 1" OR malt1 OR "Mannan Binding Lectin" OR "Mannose Binding Lectin" OR map3k14 OR "mitogen-activated protein kinase 14" OR masp2 OR "MBL-associated serine protease 2" OR "MBL associated serine protease 2" OR mbs1 OR mcm4 OR "minichromosome maintenance complex component 4" OR mefv OR mkl1 OR mogs OR "mannosyl-oligosaccharide glucosidase" OR "mannosyl oligosaccharide glucosidase" OR msh6 OR "muts homolog 6" OR msn OR moesin OR mst1 OR "macrophage stimulating 1" OR mthfd1 OR "methylenetetrahydrofolate dehydrogenase" OR mvk OR "Mevalonate Kinase" OR myd88 OR mysm1 OR nbas OR nbs1 OR ncf1 OR ncf2 OR ncf4 OR "neutrophil cytosolic factor 1" OR "neutrophil cytosolic factor 2" OR "neutrophil cytosolic factor 4" OR ncstn OR nicastrin OR nfat5 OR "nuclear factor of activated T-cells 5" OR "nuclear factor of activated T cells 5" OR nfe2l2 OR nfkb1 OR nfkb2 OR "nuclear factor kappa b subunit 1" OR "nuclear factor kappa b subunit 2" OR nfkbia OR "nfkb inhibitor alpha" OR nhej1 OR "nonhomologous end-joining factor 1" OR "nonhomologous end joining factor 1" OR nik OR "nfkb inducing kinase" OR "nf-kb inducing kinase" OR "nf kb inducing kinase" OR nlrc4 OR "nlf family CARD domain containing 4" OR nlrp1 OR nlrp12 OR nlrp3 OR "nlr family pyrin domain containing 12" OR "nlr family pyrin domain containing 3" OR "nlr family pyrin domain containing 1" OR nod2 OR "Nucleotide Binding Oligomerization Domain Containing 2" OR nola2 OR nola3 OR "nucleolar protein family A member 2" OR "nucleolar protein family A member 3" OR nras OR nsmce3 OR oas1 OR "ORAI-I" OR "ORAI I" OR ostm1 OR otulin OR ox40 OR "tumor necrosis factor superfamily member 4 " OR "TNF receptor superfamily member 4 " OR "TNF superfamily member 4" OR p22phox OR p40phox OR p47phox OR p67phox OR "Neutrophil cytosol factor P22" OR "Neutrophil cytosol factor P40" OR "Neutrophil cytosol factor P47" OR "Neutrophil cytosol factor P67" OR palb2 OR parn OR "poly A-specific ribonuclease" OR "poly A specific ribonuclease" OR "polyA specific ribonuclease" OR pepd OR pgm3 OR "Phosphoglucomutase 3" OR pi3kr1 OR "Phosphoinositide-3-Kinase Regulatory Subunit 1" OR "Phosphoinositide 3 Kinase Regulatory Subunit 1" OR pik3cd OR pik3r1 OR plcg2 OR "phospholipase C gamma 2" OR plekhm1 OR pms2 OR "PMS1 Homolog 2" OR pnp OR "Purine Nucleoside Phosphorylase" OR pola1 OR pold1 OR pold2 OR pole1 OR pole2 OR "DNA polymerase epsilon" OR polr31 OR polr3c OR polr3f OR prf1 OR "perforin 1" OR "PRKC Delta" OR "protein kinase c delta" OR prkcd OR prkcdc OR psen OR psenen OR psmb8 OR "Proteasome 20S Subunit Beta 8" OR psmg2 OR pstpip1 OR "Proline Serine Threonine Phosphatase Interacting Protein 1 " OR pten OR ptprc OR rab27a OR rac2 OR "Rac 2" OR "Rac Family Small GTPase 2" OR rad51 OR rag OR rag1 OR rag2 OR "recombination activating gene 1" OR "recombination activating 1" OR "recombination activating gene 2" OR "recombination activating 2" OR ranbp2 OR rand51c OR rasgrp1 OR rbck1 OR "RANBP2-type" OR "RANBP2 type" OR recql3 OR "RECQ Protein-Like 3" OR "RECQ Protein Like 3" OR rel OR rela OR relb OR rfwd3 OR rfx5 OR "Regulatory Factor X5" OR rfxank OR "Regulatory Factor X Associated" OR rfxap OR rhoh OR ripk1 OR rmrp OR "RNA component of mitochondrial RNA processing endoribonuclease" OR rnaseh2a OR rnaseh2b OR rnaseh2c OR "Ribonuclease H2 Subunit A" OR "Ribonuclease H2 Subunit B" OR "Ribonuclease H2 Subunit C" OR rnf168 OR rnf31 OR "ring finger protein 168" OR "ring finger protein 31" OR rnu4atac OR robld3 OR "Roadblock domain containing 3" OR rorc OR "RAR Related Orphan Receptor C" OR rpsa OR "Ribosomal Protein SA" OR rtel1 OR "Regulator of Telomere Elongation Helicase 1" OR samd9 OR samd9l OR samhd1 OR sbds OR sec61a1 OR sema3e OR "semaphorin 3E" OR serping1 OR sh2d1a OR "SH2 Domain Containing 1A" OR sh3bp2 OR "SH3 Domain Binding Protein 2" OR sh3kbp1 OR skiv2l OR slc29a3 OR slc35c1 OR slc37a4 OR "Solute Carrier Family 29 Member 3" OR "Solute Carrier Family 35 Member C1" OR "Solute Carrier Family 37 Member A4" OR slc39a7 OR slc46a1 OR "Solute Carrier Family 39 Member A7" OR "Solute Carrier Family 46 Member A1" OR slc7a7 OR "Solute Carrier Family 7 Member A7" OR slx4 OR smarcal1 OR smarcd2 OR snx10 OR sp110 OR spink5 OR "Serine Peptidase Inhibitor Kazal Type 5" OR sppl2a OR srp54 OR srp72 OR "STAT 1" OR stat1 OR "STAT 2" OR stat2 OR "STAT 3" OR stat3 OR "STAT 5b" OR stat5b OR "STAT 5" OR stat5 OR "Signal Transducer and Activator of Transcription 5b" OR "Signal Transducer and Activator of Transcription 5" OR "Signal Transducer and Activator of Transcription 1" OR "Signal Transducer and Activator of Transcription 2" OR "Signal Transducer and Activator of Transcription 3" OR "stim-1" OR stim1 OR "Stromal Interaction Molecule 1" OR stk4 OR stn1 OR stx11 OR "Syntaxin 11" OR stxbp2 OR "syntaxin binding protein 2" OR tap1 OR tap2 OR "transporter 1" OR "transporter 2" OR tapbp OR "tap binding protein" OR taz OR tafazzin OR tbk1 OR "tank binding kinase 1" OR tbx1 OR "t box transcription factor 1" OR tcf3 OR "transcription factor 3" OR tcirg1 OR tcn2 OR "Transcobalamin 2" OR "tcr-alpha" OR "tcr alpha" OR "t cell receptor alpha" OR terc OR "Telomerase RNA Component" OR tert OR "Telomerase Reverse Transcriptase" OR tfrc OR tgfb1 OR tgfbr1 OR tgfbr2 OR thbd OR thrombomodulin OR ticam1 OR tinf2 OR "TRF1-interacting nuclear factor 2" OR "TRF1 interacting nuclear factor 2" OR "TERF1-interacting nuclear factor 2" OR "TERF1 interacting nuclear factor 2" OR thymotaxin OR tirap OR tlr3 OR "toll like receptor 3" OR tmc6 OR tmc8 OR "Transmembrane Channel Like 6" OR "Transmembrane Channel Like 8" OR tmem173 OR "Transmembrane Protein 173" OR tnfaip3 OR tnfrsf1 OR "TNF Receptor Superfamily Member" OR tnfrsf11a OR tnfrsf13b OR tnfrsf13c OR tnfrsf1a OR tnfrsf4 OR tnfrsf6 OR tnfrsf9 OR tnfsf11 OR tnfsf12 OR tnfsf6 OR top2b OR tp53 OR tpp1 OR tpp2 OR "Tripeptidyl Peptidase 1" OR "Tripeptidyl Peptidase 2" OR trac OR traf3 OR "TNF Receptor Associated Factor 3" OR traf3ip2 OR trex1 OR "Three Prime Repair Exonuclease 1" OR trim22 OR trnt1 OR "TRNA Nucleotidyl Transferase 1" OR ttc37 OR "Tetratricopeptide Repeat Domain 37" OR ttc7a OR "Tetratricopeptide Repeat Domain 7A" OR txb1 OR tyk2 OR "Tyrosine Kinase 2" OR ube2t OR unc13d OR "unc-13 homolog D" OR "unc 13 homolog D" OR unc93b1 OR "unc-93 homolog b1" OR "unc 93 homolog b1" OR ung OR "Uracil DNA Glycosylase" OR usb1 OR usp18 OR vps13b OR "Vacuolar Protein Sorting 13 Homolog B" OR vps45 OR vps45a OR "Vacuolar Protein Sorting 45 A" OR "was gene" OR "was protein" OR "WASP Actin Nucleation Promoting Factor" OR wdr1 OR wipf1 OR wrap53 OR xiap OR xrcc1 OR xrcc9 OR "ZAP-70" OR zap70 OR "Zeta Chain Of T Cell Receptor Associated Protein Kinase 70" OR zbtb24 OR "BTB Domain Containing 24" OR znf341 OR "interleukin-2 receptor subunit gamma" OR "interleukin 2 receptor subunit gamma" OR "moebius syndrome" OR "X-Linked" OR "X Linked" ) AND TITLE-ABS-KEY ( autoimmun* OR immunodeficien* OR deficien* ) ) OR ( TITLE-ABS-KEY ( "inborn errors" OR "inborn error" ) AND TITLE-ABS-KEY ( immun* OR autoimmun* OR deficien* OR immunodeficien* ) ) ) ) AND ( TITLE-ABS-KEY ( coronavir* OR "corona virus" OR "corona pandemic" OR betacoronavir* OR covid19 OR covid OR ncov OR "CoV 2" OR cov2 OR sarscov2 OR sars2 OR 2019ncov OR "novel CoV" OR "wuhan virus" OR ncov19 OR "solidarity trial" OR "operation warp speed" OR covax OR "ACT-Accelerator" OR bnt162b2 OR comirnaty OR "mRNA-1273" OR covishield OR azd1222 OR "Sputnik V" OR coronavac OR "BBIBP-CorV" OR "Ad26.CoV2.S" OR "JNJ-78436735" OR ad26covs1 OR vac31518 OR epivaccorona OR convidicea OR "Ad5-nCoV" OR covaxin OR covivac OR zf2001 OR "NVX-CoV2373" OR "ZyCoV-D" OR "CIGB 66" OR cvncov OR "INO-4800" OR "VIR-7831" OR "UB-612" OR "BNT162" OR "Soberana 1" OR "Soberana 2 " OR "B.1.1.7" OR "VOC 202012/01" OR "VOC202012/01" OR "VUI 202012/01" OR "VUI202012/01" OR "501Y.V1" OR "UK Variant" OR "Kent Variant" OR "VOC 202102/02" OR "VOC202102/02" OR "B.1.351" OR "VOC 202012/02" OR "VOC202012/02" OR "20H/501.V2" OR "20H/501Y.V2" OR "501Y.V2" OR "501.V2" OR "South African Variant" OR "B.1.1.28.1" OR "B.1.1.28" OR "B.1.1.248" OR "VOC 202101/02" OR "VOC202101/02" OR "VUI202101/02" OR "VUI 202101/02" OR "501Y.V3" OR "brazil Variant" OR "P.1 variant" OR "P.1 lineage" OR "Lineage P.1" OR "B.1.427" OR "B.1.429" OR "20C/S:452R" OR "CAL.20C" OR "CAL.20C/L452R" OR "20C/L452R" OR "20-C variant" OR cavui1 OR "GH/451R.V1" OR "B.1.526" OR "20C/S:484K" OR "B.1.1.28.3" OR "P3 variant" OR "P.3 Strain" OR "Lineage P.3" OR "P.3 Lineage" OR "PHL-B.1.1.28" OR "VUI-21MAR-02" OR "B.1.1.28.2" OR "20B/S.484K" OR "P.2 variant" OR "P.2 strain" OR "P.2 Lineage" OR "GR clade" OR "VUI 202101/01" OR "VUI202101/01" OR "B.1.177" OR "20A.EU1" OR "20A.EU2" OR "Variant 20A" OR "B.1.525" OR "G/484K.V3" OR "20A/S:484K" OR uk1188 OR "B.1.616" OR "20C/ B1" OR "clade 20C" OR "B.1.617" OR "VUI-21APR-01" OR "B.1.618" OR "VUI-202102/01" OR "VUI-21FEB-01" OR d614g OR n501y OR a570d OR p681h OR k417n OR e484k OR "K417N/T" OR l452r OR s477n OR d253g OR t951 OR a701v OR 8477h OR v30l OR a220v OR t445c OR c6286t OR c26801g OR e484l OR d66h OR y144v OR q677h OR d215g OR 484k OR p681r OR d651g OR e484q OR f888l OR f565l OR v1176f OR f157s OR l452r OR d614g OR t781i OR t859n OR d950h OR l5f OR k417t OR l18f OR t20n OR p26s OR r190s OR h655y OR t1027i OR a701v OR k417n OR s494p OR t716i OR s982a OR d1118h OR k1191n ) OR TITLE-ABS-KEY ( sars AND cov ) OR ( TITLE-ABS-KEY ( wuhan OR hubei OR huanan ) AND TITLE-ABS-KEY ( "severe acute respiratory" OR pneumonia* ) AND TITLE-ABS-KEY ( outbreak* ) ) ) AND ( LIMIT-TO ( PUBYEAR , 2023 ) OR LIMIT-TO ( PUBYEAR , 2019 ) ) AND ( LIMIT-TO ( LANGUAGE , "English" ) ) | 02/28/2022 | 168 articles  -53  duplicates  =115 articles |
| Nature | (11q23 OR 11q23del OR acd OR ace OR "angiotensin converting enzyme" OR acp5 OR "acid phosphate 5" OR actb OR ada OR ada2 OR adam17 OR tace OR "adenosine deaminase" OR adar1 OR aicda OR "AID enzyme" OR "activation induced cytidine deaminase" OR "activation-induced cytidine deaminase" OR apeced OR "aire autoimmune regulator" OR ak2 OR "Adenylate kinase 2" OR "Adenylate kinase-2" OR alpi OR "intestinal alkaline phosphatase" OR ap1s3 OR "adaptor related protein complex 1" OR ap3b1 "adaptor related protein complex 3" OR ap3d1 OR "APOL-I" OR "APOL I" OR "Apolipoprotein L" OR arhgef1 OR arpc1b OR atm OR "ataxia telangiectasia mutated" OR atp6ap1 OR b2m OR "beta 2-Microglobulin" OR "beta 2 Microglobulin" OR bach2 OR baff OR "B-Cell Activation factor" OR "B Cell Activation factor" OR "B-Cell Activating factor" OR "B Cell Activating factor" OR bcl10 OR bcl11 OR bcl11b OR bcli10 OR blm OR blnk OR "B Cell linker" OR bloc1s6 OR "biogenesis of lysosomal organelles complex 1" OR brca1 OR brca2 OR brip1 OR btk OR "B Cell linker" OR "Bruton tyrosine kinase" OR c1qa OR c1qb OR c1qc OR "Complement C1q" OR "Complement C1s" OR "Complement activating enzyme" OR "Complement system protein" OR c1r OR c1s OR c2 OR c3 OR c4 OR c4a OR c4b OR c5 OR c6 OR c7 OR c8 OR c8a OR c8b OR c8g OR c9 OR card11 OR "Caspase recruitment domain" OR card14 OR card15 OR card9 OR carmil1 OR carmil2 OR casp8 OR "Caspase Eight" OR "Caspase 8" OR "Caspase-8" OR casp10 OR "Caspase ten" OR "Caspase 10" OR "Caspase-10" OR ccbe1 OR "calcium-binding EGF domains" OR "calcium binding EGF domains" OR cd16 OR cd19 OR cd20 OR cd21 OR cd25 OR cd27 OR cd3* OR cd3d OR cd3e OR cd3g OR cd3z OR cd40 OR cd40lg OR cd45 OR cd46 OR cd55 OR cd59 OR cd70 OR cd79a OR cd79b OR cd79 OR cd8 OR cd81 OR cd8a OR cdca7 OR cebpe OR "C-EBP epsilon" OR "CEBP epsilon" OR "C EBP epsilon" OR "CCAAT enhancer binding protein" OR cfb OR cfd OR cfh OR cfhr1 OR cfhr2 OR cfhr3 OR cfhr4 OR cfhr5 OR cfi OR cfp OR cftr OR "complement factor b" OR "complement factor d" OR "complement factor h" OR "complement factor i" OR "complement factor p" OR "cystic fibrosis transmembrane conductance regulator" OR chd7 OR "chromodomain helicase DNA binding protein 7" OR cib1 OR ciita OR "class II transactivator" OR "Class II Major Histocompatibility Complex Transactivator" OR clbp OR clcn7 OR "chloride channel 7" OR clpb OR coh1 OR copa OR "COPI Coat Complex Subunit Alpha" OR "Coronin-1A" OR "Coronin 1A" OR coro1a OR csf2ra OR csf3r OR csf2rb OR "colony stimulating factor 2" OR "colony stimulating factor 3" OR ctc1 "conserved telomere maintenance component 1" OR ctla4 OR "cytotoxic T lymphocyte associated protein 4" OR ctps1 OR "ctp synthase 1" OR ctsc OR "cathepsin C" OR cxcr4 OR cxcra OR cyba OR cybb OR cybc1 OR "cytochrome b-245" OR dbr1 OR dclre1b OR dclre1c OR "DNA cross-link repair" OR "DNA cross link repair" OR def6 OR "Del1op13-p14" OR del1op13 OR dkc1 OR "Dyskerin Pseudouridine Synthase" OR dnajc21 OR dnase1l3 OR dnase2 OR dnmt3b OR "DNA methyltransferase 3B" OR "DNA methyltransferase 3 beta" OR dock2 OR dock8 OR "dedicator of cytokinesis" OR efl1 OR "euphorbia factor L1" OR elane OR "elastase neutrophil expressed" OR epg5 OR "ectopic P-granules autophagy protein 5" OR "ectopic P granules autophagy protein 5" OR erbb2ip OR ercc4 OR ercc6l2 OR extl3 OR faap24 OR fadd OR "Fas-Associated Death Domain" OR "Fas Associated Death Domain" OR fanca OR fancb OR fancc OR fancd2 OR fance OR fanf OR fanci OR fancl OR fancm OR "Fanconi Anemia Complementation Group" OR DKCA6 OR "Coats plus syndrome due to CTC1 deficiency" OR DKCX1 OR EVI1 OR "MECOM deficiency" OR DKCB2 OR DKCB1 OR DKCB6 OR DKCA4 OR DKCB5 OR "Ataxia Pancytopenia Syndrome" OR BMFS1 OR "SRP72-deficiency" OR "Coats plus syndrome due to STN1 deficiency" OR DKCA1 OR DKCA2 OR DKCB4 OR DKCA3 OR BMFS5 OR DKCB3 OR fas OR faslg OR fat4 OR fcgr3a OR "Fc gamma receptor" OR fcho1 OR fcn3 OR "ficolin 3" OR fremt1 OR "fermitin 1" OR fermt3 OR "fermitin 3" OR foxn1 OR foxp3 OR "forkhead box P3" OR fpr1 OR "Formyl Peptide Receptor 1" OR g6pc3 OR "Glucosephosphate Dehydrogenase" OR "glucose 6 phosphatase catalytic" OR g6pt1 OR "gata-2" OR "gata 2" OR gata2 OR "gata binding protein 2" OR gfi1 OR "growth factor independent 1" OR gins1 OR havcr2 OR hax1 OR "HS1-associated protein X-1" OR "HS1 associated protein X 1" OR "HS1 associated protein X1" OR "HCLS1-associated protein X-1" OR " HCLS1 associated protein X 1" OR " HCLS1 associated protein X1" OR hells OR homx OR hmox1 OR hyou1 OR icos OR "Inducible T-Cell Co-Stimulator" OR "Inducible T Cell Costimulator" OR icoslg OR ifih1 OR "Interferon Induced Helicase c domain" OR ifnar1 OR ifnar2 OR ifngr1 OR ifngr2 OR ifnyr1 OR ifnyr2 OR "interferon gamma receptor" OR "inf gamma r1" OR "inf gamma r2" OR ighm OR igkc OR "immunoglobulin kappa constant" OR igll1 OR "immunoglobulin lambda-like polypeptide 1" OR "immunoglobulin lambda like polypeptide 1" OR ikbkb OR ikbkg OR "I kappa b kinase" OR "inhibitor of nuclear factor b kinase" OR ikzf1 OR il10 OR "IL-10" OR "IL 10" OR "interleukin 10" OR "interleukin-10" OR il10ra OR "IL-10RA" OR "IL 10RA" OR "interleukin 10RA" OR "interleukin-10RA" OR il10rb OR "IL-10RB" OR "IL 10RB" OR "interleukin 10RB" OR "interleukin-10RB" OR il12r OR "IL-12R" OR "IL 12R" OR "interleukin 12R" OR "interleukin-12R" OR il12rb OR "IL-12RB" OR "IL 12RB" OR "interleukin 12RB" OR "interleukin-12RB" OR il12rb1 OR "IL-12RB1" OR "IL 12RB1" OR "interleukin 12RB1" OR "interleukin-12RB1" OR il12rb2 OR "IL-12RB2" OR "IL 12RB2" OR "interleukin 12RB2" OR "interleukin-12RB2" OR il17f OR "IL-17F" OR "IL 17F" OR "interleukin 17F" OR "interleukin-17F" OR il17ra OR "IL-17RA" OR "IL 17RA" OR "interleukin 17RA" OR "interleukin-17RA" OR il17rc OR "IL-17RC" OR "IL 17RC" OR "interleukin 17RC" OR "interleukin-17RC" OR il18bp OR "IL-18BP" OR "IL 18BP" OR "interleukin 18BP" OR "interleukin-18BP" OR il1rn OR "IL-1RN" OR "IL 1RN" OR "interleukin 1RN" OR "interleukin-1RN" OR il23r OR "IL-23R" OR "IL 23R" OR "interleukin 23R" OR "interleukin-23R" OR il2i OR "IL-2I" OR "IL 2I" OR "interleukin 2I" OR "interleukin-2I" OR il2ir OR "IL-2IR" OR "IL 2IR" OR "interleukin 2IR" OR "interleukin-2IR" OR il2ira OR "IL-2IRA" OR "IL 2IRA" OR "interleukin 2IRA" OR "interleukin-2IRA" OR il2irb OR "IL-2IRB" OR "IL 2IRB" OR "interleukin 2IRB" OR "interleukin-2IRB" OR il2irg OR "IL-2IRG" OR "IL 2IRG" OR "interleukin 2IRG" OR "interleukin-2IRG" OR il36rn OR "IL-36RN" OR "IL 36RN" OR "interleukin 36RN" OR "interleukin-36RN" OR il6r OR "IL-6R" OR "IL 6R" OR "interleukin 6R" OR "interleukin-6R" OR il6st OR "IL-6ST" OR "IL 6ST" OR "interleukin 6ST" OR "interleukin-6ST" OR ilr7 OR "IL-R7" OR "IL R7" OR "interleukin R7" OR "interleukin- R7" OR ino80 OR irak1 OR "IRAK-1" OR "IRAK 1" OR "interleukin 1" OR "interleukin-1" OR irak4 OR "IRAK-4" OR "IRAK 4" OR "interleukin 4" OR "interleukin-4" OR irf2bp2 OR irf3 OR irf4 OR irf7 OR irf8 OR irf9 OR "interferon regulatory factor 2" OR "interferon regulatory factor 3" OR "interferon regulatory factor 4" OR "interferon regulatory factor 7" OR "interferon regulatory factor 8" OR "interferon regulatory factor 9" OR isg15 OR "itchy homolog e3" OR "itchy e3" OR itgam OR "integrin subunit alpha" OR "integrin subunit beta" OR itgb2 OR itk OR "il2 inducible t cell kinase" OR jagn1 OR "jagunal homolg 1" OR jak1 OR jak3 OR "janus kinase 1" OR "janus kinase 3" OR kdm6a OR kindlin3 OR kmt2a OR kmt2d OR kras OR lamtor2 OR lat OR lck OR "leukocyte c terminal src kinase" OR "leukocyte c-terminal src kinase" OR lig1 OR "ligase I" OR lig4 OR "ligase IV" OR lpin2 OR "lipin 2" OR lrba OR "lipopolysaccharide responsive beige like anchor protein " OR "lps responsive beige like anchor protein " OR lyst OR "lysosomal trafficking regulator" OR mad2l2 OR magt1 OR "magnesium transporter 1" OR malt1 OR "Mannan Binding Lectin" OR "Mannose Binding Lectin" OR map3k14 OR "mitogen-activated protein kinase 14" OR masp2 OR "MBL-associated serine protease 2" OR "MBL associated serine protease 2" OR mbs1 OR mcm4 OR "minichromosome maintenance complex component 4" OR mefv OR mkl1 OR mogs OR "mannosyl-oligosaccharide glucosidase" OR "mannosyl oligosaccharide glucosidase" OR msh6 OR "muts homolog 6" OR msn OR moesin OR mst1 OR "macrophage stimulating 1" OR mthfd1 OR "methylenetetrahydrofolate dehydrogenase" OR mvk OR "Mevalonate Kinase" OR myd88 OR mysm1 OR nbas OR nbs1 OR ncf1 OR ncf2 OR ncf4 OR "neutrophil cytosolic factor 1" OR "neutrophil cytosolic factor 2" OR "neutrophil cytosolic factor 4" OR ncstn OR nicastrin OR nfat5 OR "nuclear factor of activated T-cells 5" OR "nuclear factor of activated T cells 5" OR nfe2l2 OR nfkb1 OR nfkb2 OR "nuclear factor kappa b subunit 1" OR "nuclear factor kappa b subunit 2" OR nfkbia OR "nfkb inhibitor alpha" OR nhej1 OR "nonhomologous end-joining factor 1" OR "nonhomologous end joining factor 1" OR nik OR "nfkb inducing kinase" OR "nf-kb inducing kinase" OR "nf kb inducing kinase" OR nlrc4 OR "nlf family CARD domain containing 4" OR nlrp1 OR nlrp12 OR nlrp3 OR "nlr family pyrin domain containing 12" OR "nlr family pyrin domain containing 3" OR "nlr family pyrin domain containing 1" OR nod2 OR "Nucleotide Binding Oligomerization Domain Containing 2" OR nola2 OR nola3 OR "nucleolar protein family A member 2" OR "nucleolar protein family A member 3" OR nras OR nsmce3 OR oas1 OR "ORAI-I" OR "ORAI I" OR ostm1 OR otulin OR ox40 OR "tumor necrosis factor superfamily member 4 " OR "TNF receptor superfamily member 4 " OR "TNF superfamily member 4" OR p22phox OR p40phox OR p47phox OR p67phox OR "Neutrophil cytosol factor P22" OR "Neutrophil cytosol factor P40" OR "Neutrophil cytosol factor P47" OR "Neutrophil cytosol factor P67" OR palb2 OR parn OR "poly A-specific ribonuclease" OR "poly A specific ribonuclease" OR "polyA specific ribonuclease" OR pepd OR pgm3 OR "Phosphoglucomutase 3" OR pi3kr1 OR "Phosphoinositide-3-Kinase Regulatory Subunit 1" OR "Phosphoinositide 3 Kinase Regulatory Subunit 1" OR pik3cd OR pik3r1 OR plcg2 OR "phospholipase C gamma 2" OR plekhm1 OR pms2 OR "PMS1 Homolog 2" OR pnp OR "Purine Nucleoside Phosphorylase" OR pola1 OR pold1 OR pold2 OR pole1 OR pole2 OR "DNA polymerase epsilon" OR polr31 OR polr3c OR polr3f OR prf1 OR "perforin 1" OR "PRKC Delta" OR "protein kinase c delta" OR prkcd OR prkcdc OR psen OR psenen OR psmb8 OR "Proteasome 20S Subunit Beta 8" OR psmg2 OR pstpip1 OR "Proline Serine Threonine Phosphatase Interacting Protein 1 " OR pten OR ptprc OR rab27a OR rac2 OR "Rac 2" OR "Rac Family Small GTPase 2" OR rad51 OR rag OR rag1 OR rag2 OR "recombination activating gene 1" OR "recombination activating 1" OR "recombination activating gene 2" OR "recombination activating 2" OR ranbp2 OR rand51c OR rasgrp1 OR rbck1 OR "RANBP2-type" OR "RANBP2 type" OR recql3 OR "RECQ Protein-Like 3" OR "RECQ Protein Like 3" OR rel OR rela OR relb OR rfwd3 OR rfx5 OR "Regulatory Factor X5" OR rfxank OR "Regulatory Factor X Associated" OR rfxap OR rhoh OR ripk1 OR rmrp OR "RNA component of mitochondrial RNA processing endoribonuclease" OR rnaseh2a OR rnaseh2b OR rnaseh2c OR "Ribonuclease H2 Subunit A" OR "Ribonuclease H2 Subunit B" OR "Ribonuclease H2 Subunit C" OR rnf168 OR rnf31 OR "ring finger protein 168" OR "ring finger protein 31" OR rnu4atac OR robld3 OR "Roadblock domain containing 3" OR rorc OR "RAR Related Orphan Receptor C" OR rpsa OR "Ribosomal Protein SA" OR rtel1 OR "Regulator of Telomere Elongation Helicase 1" OR samd9 OR samd9l OR samhd1 OR sbds OR sec61a1 OR sema3e OR "semaphorin 3E" OR serping1 OR sh2d1a OR "SH2 Domain Containing 1A" OR sh3bp2 OR "SH3 Domain Binding Protein 2" OR sh3kbp1 OR skiv2l OR slc29a3 OR slc35c1 OR slc37a4 OR "Solute Carrier Family 29 Member 3" OR "Solute Carrier Family 35 Member C1" OR "Solute Carrier Family 37 Member A4" OR slc39a7 OR slc46a1 OR "Solute Carrier Family 39 Member A7" OR "Solute Carrier Family 46 Member A1" OR slc7a7 OR "Solute Carrier Family 7 Member A7" OR slx4 OR smarcal1 OR smarcd2 OR snx10 OR sp110 OR spink5 OR "Serine Peptidase Inhibitor Kazal Type 5" OR sppl2a OR srp54 OR srp72 OR "STAT 1" OR stat1 OR "STAT 2" OR stat2 OR "STAT 3" OR stat3 OR "STAT 5b" OR stat5b OR "STAT 5" OR stat5 OR "Signal Transducer and Activator of Transcription 5b" OR "Signal Transducer and Activator of Transcription 5" OR "Signal Transducer and Activator of Transcription 1" OR "Signal Transducer and Activator of Transcription 2" OR "Signal Transducer and Activator of Transcription 3" OR "stim-1" OR stim1 OR "Stromal Interaction Molecule 1" OR stk4 OR stn1 OR stx11 OR "Syntaxin 11" OR stxbp2 OR "syntaxin binding protein 2" OR tap1 OR tap2 OR "transporter 1" OR "transporter 2" OR tapbp OR "tap binding protein" OR taz OR tafazzin OR tbk1 OR "tank binding kinase 1" OR tbx1 OR "t box transcription factor 1" OR tcf3 OR "transcription factor 3" OR tcirg1 OR tcn2 OR "Transcobalamin 2" OR "tcr-alpha" OR "tcr alpha" OR "t cell receptor alpha" OR terc OR "Telomerase RNA Component" OR tert OR "Telomerase Reverse Transcriptase" OR tfrc OR tgfb1 OR tgfbr1 OR tgfbr2 OR thbd OR thrombomodulin OR ticam1 OR tinf2 OR "TRF1-interacting nuclear factor 2" OR "TRF1 interacting nuclear factor 2" OR "TERF1-interacting nuclear factor 2" OR "TERF1 interacting nuclear factor 2" OR thymotaxin OR tirap OR tlr3 OR "toll like receptor 3" OR tmc6 OR tmc8 OR "Transmembrane Channel Like 6" OR "Transmembrane Channel Like 8" OR tmem173 OR "Transmembrane Protein 173" OR tnfaip3 OR tnfrsf1 OR "TNF Receptor Superfamily Member" OR tnfrsf11a OR tnfrsf13b OR tnfrsf13c OR tnfrsf1a OR tnfrsf4 OR tnfrsf6 OR tnfrsf9 OR tnfsf11 OR tnfsf12 OR tnfsf6 OR top2b OR tp53 OR tpp1 OR tpp2 OR "Tripeptidyl Peptidase 1" OR "Tripeptidyl Peptidase 2" OR trac OR traf3 OR "TNF Receptor Associated Factor 3" OR traf3ip2 OR trex1 OR "Three Prime Repair Exonuclease 1" OR trim22 OR trnt1 OR "TRNA Nucleotidyl Transferase 1" OR ttc37 OR "Tetratricopeptide Repeat Domain 37" OR ttc7a OR "Tetratricopeptide Repeat Domain 7A" OR txb1 OR tyk2 OR "Tyrosine Kinase 2" OR ube2t OR unc13d OR "unc-13 homolog D" OR "unc 13 homolog D" OR unc93b1 OR "unc-93 homolog b1" OR "unc 93 homolog b1" OR ung OR "Uracil DNA Glycosylase" OR usb1 OR usp18 OR vps13b OR "Vacuolar Protein Sorting 13 Homolog B" OR vps45 OR vps45a OR "Vacuolar Protein Sorting 45 A" OR "was gene" OR "was protein" OR "WASP Actin Nucleation Promoting Factor" OR wdr1 OR wipf1 OR wrap53 OR xiap OR xrcc1 OR xrcc9 OR "ZAP-70" OR zap70 OR "Zeta Chain Of T Cell Receptor Associated Protein Kinase 70" OR zbtb24 OR "BTB Domain Containing 24" OR znf341 OR "interleukin-2 receptor subunit gamma" OR "interleukin 2 receptor subunit gamma" OR "moebius syndrome" OR "X-Linked" OR "X Linked")  AND  (autoimmun* OR immunodeficien* OR deficien*)  OR  ("inborn errors" OR "inborn error" AND immun* OR autoimmun* OR deficien* OR immunodeficien*)  AND  (coronavir* OR "corona virus" OR "corona pandemic" OR betacoronavir* OR covid19 OR covid OR ncov OR "CoV 2" OR cov2 OR sarscov2 OR sars2 OR 2019ncov OR "novel CoV" OR "wuhan virus" OR NCOV19 OR "solidarity trial" OR "operation warp speed" OR COVAX OR "ACT-Accelerator" OR BNT162b2 OR comirnaty OR "mRNA-1273" OR CoviShield OR AZD1222 OR "Sputnik V" OR CoronaVac OR "BBIBP-CorV" OR "Ad26.CoV2.S" OR "JNJ-78436735" OR Ad26COVS1 OR VAC31518 OR EpiVacCorona OR Convidicea OR "Ad5-nCoV" OR Covaxin OR CoviVac OR ZF2001 OR "NVX-CoV2373" OR "ZyCoV-D" OR "CIGB 66" OR CVnCoV OR "INO-4800" OR "VIR-7831" OR "UB-612" OR "BNT162" OR "Soberana 1" OR "Soberana 2 " OR "B.1.1.7" OR "VOC 202012/01" OR "VOC202012/01" OR "VUI 202012/01" OR "VUI202012/01" OR "501Y.V1" OR "UK Variant" OR "Kent Variant" OR "VOC 202102/02" OR "VOC202102/02" OR "B.1.351" OR "VOC 202012/02" OR "VOC202012/02" OR "20H/501.V2" OR "20H/501Y.V2" OR "501Y.V2" OR "501.V2" OR "South African Variant" OR "B.1.1.28.1" OR "B.1.1.28" OR "B.1.1.248" OR "VOC 202101/02" OR "VOC202101/02" OR "VUI202101/02" OR "VUI 202101/02" OR "501Y.V3" OR "brazil Variant" OR "P.1 variant" OR "P.1 lineage" OR "Lineage P.1" OR "B.1.427" OR "B.1.429" OR "20C/S:452R" OR "CAL.20C" OR "CAL.20C/L452R" OR "20C/L452R" OR "20-C variant" OR CAVUI1 OR "GH/451R.V1" OR "B.1.526" OR "20C/S:484K" OR "B.1.1.28.3" OR "P3 variant" OR "P.3 Strain" OR "Lineage P.3" OR "P.3 Lineage" OR "PHL-B.1.1.28" OR "VUI-21MAR-02" OR "B.1.1.28.2" OR "20B/S.484K" OR "P.2 variant" OR "P.2 strain" OR "P.2 Lineage" OR "GR clade" OR "VUI 202101/01" OR "VUI202101/01" OR "B.1.177" OR "20A.EU1" OR "20A.EU2" OR "Variant 20A" OR "B.1.525" OR "G/484K.V3" OR "20A/S:484K" OR UK1188 OR "B.1.616" OR "20C/ B1" OR "clade 20C" OR "B.1.617" OR "VUI-21APR-01" OR "B.1.618" OR "VUI-202102/01" OR "VUI-21FEB-01" OR D614G OR N501Y OR A570D OR P681H OR K417N OR E484K OR "K417N/T" OR L452R OR S477N OR D253G OR T951 OR A701V OR 8477H OR V30L OR A220V OR T445C OR C6286T OR C26801G OR E484L OR D66H OR Y144V OR Q677H OR D215G OR 484K OR P681R OR D651G OR E484Q OR F888L OR F565L OR V1176F OR F157S OR L452R OR D614G OR T781I OR T859N OR D950H OR L5F OR K417T OR L18F OR T20N OR P26S OR R190S OR H655Y OR T1027I OR A701V OR K417N OR S494P OR T716I OR S982A OR D1118H OR K1191N" anywhere or "sars AND cov" anywhere or "wuhan OR hubei OR huanan" anywhere and ""severe acute respiratory" OR pneumonia*) AND (outbreak*)  Limits: 2019- | 02/28/2022 | 21 articles  -4 duplicates  =17 articles |

Table Supplementary 2: Inborn errors of immunity in each inborn error of immunity class

| Inborn Errors of Immunity Classes | Inborn Errors of Immunity |
| --- | --- |
| Cellular and humoral immunodeficiencies | - ADA Deficiency, AR - IL-7Ra Deficiency, AR - AK2 Deficiency (Reticular Dysgenesis), AR - IL-21 Deficiency, AR - B2M (MHC Class I Deficiency), AR - IL-21R Deficiency, AR - BCL10 Deficiency, AR - ITK Deficiency, AR - CARD11 Deficiency, AR - JAK3 Deficiency, AR - CD27 Deficiency, AR - LCK Deficiency, AR - CD3d, AR - LRBA Deficiency, AR - CD3e, AR - MAGT1 Deficiency, XL - CD3g, AR - MALT1 Deficiency, AR - CD3z Deficiency, AR - MST1 Deficiency, AR - CD8 Deficiency, AR - NIK Deficiency, AR - CD40 Deficiency, AR - Omenn Syndrome, AR - CD40 Ligand Deficiency, XL - OX40 Deficiency, AR - CD45 Deficiency, AR - RAG1 Deficiency, AR - Cernunnos/XLF Deficiency, AR - RAG2 Deficiency, AR - CIITA (MHC Class II Deficiency), AR - RFX5 (MHC Class II Deficiency), AR - Coronin-1A Deficiency, AR - RFXAP (MHC Class II Deficiency), AR - CTPS1 Deficiency, AR - RFXANK (MHC Class II Deficiency), AR - DCLRE1C Deficiency (Artemis), AR - RhoH Deficiency, AR - DNA Ligase IV Deficiency, AR - TAP1 (MHC Class I Deficiency), AR - DNA PKcs Deficiency, AR - TAP2 (MHC Class I Deficiency), AR - DOCK2 Deficiency, AR - TAPBP (MHC Class I Deficiency), AR - DOCK8 Deficiency, AR - TCRa Deficiency, AR - gc Deficiency, XL - ZAP-70 Deficiency, AR - ICOS Deficiency, AR |
| Combined immunodeficiencies with associated or syndromic features | - ATM Deficiency (Ataxia-Telangiectasia-AT), AR - PMS2 Deficiency, AR - CCBE1 Deficiency (Hennekam-lymphangiectasia-lymphedema Syndrome), AR - PNP Deficiency, AR - CHD7 Deficiency (CHARGE Syndrome, de novo HS or AD) - POLE1 Deficiency (FILS Syndrome), AR - SEMA3E Deficiency (CHARGE Syndrome, de novo HS or AD) - RECQL3 Deficiency (Bloom Syndrome, AR) - CHARGE Syndrome, unknown - RMRP Deficiency (Cartilage Hair Hypoplasia , AR) - Chrom 22q11.2 Deletion Syndrome (de novo HS or AD) - RNF168 Deficiency (RIDDLE Syndrome), AR - Complete DiGeorge Syndrome - SLC46A1/PCFT Deficiency, AR - DiGeorge Syndrome, unknown - SMARCAL1 Deficiency (Schimke Immunoosseous dysplasia, SIOD), AR - DNMT3B Deficiency (ICF1), AR - SPINK5 Deficiency (Comel-Netherton Syndrome , AR) - Dyskeratosis Congenita (TERC, TINF2, AD) - STAT3 Deficiency (AD-HIES) - Dyskeratosis Congenita (NOLA2, NOLA3, DCLRE1B, PARN, AR) STAT5b Deficiency, AR - Dyskeratosis Congenita (TERT, RTEL1, TPP1, AD/AR) - STIM-1 Deficiency, AR - Dyskeratosis Congenita (DKC1, XL) - TBX1 Deficiency, HS - EPG5 Deficiency (Vici Syndrome), AR - TCN2 Deficiency, AR - HOIL1 Deficiency, AR - TTC7A Deficiency (ID with multiple intestinal atresias), AR - HOIP Deficiency, AR - SP110 Deficiency (VODI Syndrome) - IKBA GOF (Ectodermal Dysplasia with ID/EDA-ID, AD) - WAS Deficiency (Wiskott-Aldrich Syndrome), XL - MCM4 Deficiency, AR - WIP Deficiency, AR - MTHFD1 Deficiency, AR - Winged Helix Deficiency (Nude), AR - NBS1 Deficiency (Nijmegen Breakage Syndrome), AR - ZBTB24 Deficiency (ICF2), AR - NEMO/IKBKG Deficiency (Ectodermal Dysplasia with ID/EDA-ID, XL) |
| Predominantly antibody deficiencies | - AID Deficiency, AR - INO80, AR - BAFF Receptor Deficiency, AR - m Heavy Chain Deficiency, AR - BLNK Deficiency, AR - MOGS Deficiency, AR - BTK Deficiency, XL - MSH6 Deficiency, AR - CARD11 Deficiency (AD, GOF) - NFKB2 Deficiency (AD) - CD19 Deficiency, AR - PI3KR1 Deficiency (AD, LOF) - CD20 Deficiency, AR - PI3KR1 Deficiency (AR) - CD21 Deficiency, AR - PI3K-d Activated (AD, GOF) - CD81 Deficiency, AR - Specific Antibody Deficiency (normal Ig and B cells), AR - Common Variable Immunodeficiency (CVID), AR - TACI Deficiency, AD/AR - E47 Deficiency, AD - Thymoma with ID, AR - Hypogammaglobulinemia of Infancy, Transient (normal B cells), AR - TRNT1 Deficiency, AR - Iga Deficiency, AR - TTC37 Deficiency, AR - Ig Deficiency, AR - TWEAK Deficiency, AD - IgA with IgG Subclass Deficiency, AR - UNG Deficiency, AR - IgG Subclass Deficiency, Isolated, AR - 5 Deficiency, AR - Ig Heavy Chain Mutations and Deletions, AR |
| Immune dysregulatory diseases | - ADA2 Deficiency, AR - UNC13D Deficiency (FHL3), AR - ADAR1 Deficiency (Aicardi-Goutieres Syndrome, AGS6) - AD STXBP2 Deficiency (FHL5), AR/AD - ALPS-CASP8 (CASP8, AR) - NFAT5 Deficiency (Haploinsufficiency, AD) - ALPS-CASP10 (CASP10, AD) - PRF1 Deficiency (FHL2), AR - ALPS-FAS (TNFRSF6, AD/AR) - PRKCd Deficiency, AR - ALPS-FASLG (TNFSF6, AR) - RNASEH2A Deficiency (Aicardi-Goutieres Syndrome, AGS4), AR - AIRE Deficiency (APECED, APS-1), AR - RNASEH2B Deficiency (Aicardi-Goutieres Syndrome, AGS2), AR - CD25 Deficiency, AR - RNASEH2C Deficiency (Aicardi-Goutieres Syndrome, AGS3), AR - LYST Deficiency (Chediak-Higashi Syndrome), AR - SAMHD1 Deficiency (Aicardi-Goutieres Syndrome, AGS5), AR - CTLA4 Deficiency, AD - SH2D1A Deficiency (XLP1) - FADD Deficiency, AR - ACP5 Deficiency (SPENCD Syndrome, AR) - FOXP3 Deficiency (IPEX Syndrome, XL) - STAT3 Deficiency (AD, GOF) - RAB27A Deficiency (Griscelli Syndrome Type 2), AR - STX11 Deficieny (FHL4), AR - AP3B1 Deficiency (Hermansky-Pudlak Syndrome Type 2) - TMEM173 Deficiency (STING-associated vasculopahty, infantile-onset), AR - PLDN Deficiency (Hermansky-Pudlak Syndrome Type 9), AR - TPP2 Deficiency (Tripeptidyl-Peptidase II), AR - IFIH1 Deiciency (Aicardi-Goutieres Syndrome 7, AGS7), AR - TREX1 Deficiency (Aicardi-Goutieres Syndrome, AGS1), AR/AD - IL-10 Deficiency, AR - XIAP Deficiency (XLP2) - IL-10Ra Deficiency, AR |
| Phagocytic diseases | - b-Actin Deficiency, AD - GATA-2 Deficiency, AD - C/EBPE Deficiency (Specific Granule Deficiency), AR - GFI1 Deficiency (SCN2), AD - CGD, AR (p22phox Deficiency) - G&PT1 Deficiency (Glycogen Storage Disease Type 1b), AR - CGD, AR (p40phox Deficiency) - HAX1 deficiency (Kostmann Disease, SCN3), AR - CGD, AR (p47phox Deficiency) - ITGB2 Deficiency (Leukocyte Adhesion Deficiency Type 1, LAD1), AR - CGD, AR (p67phox Deficiency) - JAGN1 Deficiency (Poikiloderma with neutropenia, Clericuzio-type), AR - CGD, XL (gp91 phox Deficiency) - KINDLIN3 Deficiency (Leukocyte Adhesion Deficiency Type 3, LAD3), AR - CLBP Deficiency (3-Methylglutaconic Aciduria), AR - Rac 2 Deficiency, AD - COH1 Deficiency (Cohen Syndrome), AR - ROBLD3/LAMTOR2 Deficiency (p14), AR - CSF2RA Deficiency (Pulmonary Alveolar Proteinosis), AR - SBDS Deficiency (Shwachman-Diamond Syndrome), AR - CTSC Deficiency (Papillon-Lefèvre Syndrome), AR - SLC35C1 Deficiency (Leukocyte Adhesion Deficiency Type 2, LAD2), AR - Cyclic Neutropenia (ELANE, AD) - TAZZ Deficiency (Barth Syndrome), XL - Elastase Deficiency (SCN1), AD - VPS45A Deficiency (SCN5), AR - FPR1 Deficiency (Localized Juvenile Periodontitis), AR - WAS GOF (X-linked Neutropenia/Myelodysplasia) - G6PC3 Deficiency (SCN4), AR |
| Innate immunodeficiencies | - ACT1 Deficiency, AR - ISG15 Deficiency (MSMD, AR) - APOL-I Deficiency (Trypanosomiasis, AD) - Macrophage gp91phox Deficiency (MSMD, XL) - CARD9 Deficiency, AR - MyD88 Deficiency, AR - CD16 Deficiency, AR - RORc Deficiency (MSMD, AR) - CXCR4 Gain-of-Function (WHIM Syndrome, AD) - RPSA Deficiency (Isolated Congenital Asplenia, AD) - EVER1 Deficiency (Epidermodysplasia Verruciformis 1), AR - STAT1 Deficiency (MSMD, AD) - EVER2 Deficiency (Epidermodysplasia Verruciformis 2), AR - STAT 1 Deficiency (Predisposition to severe viral infection, AR) - IFNγR1 Deficiency (MSMD, AD) - STAT 1 GOF (Chronic Mucocutaneous Candidiasis/CMC, AD) - IFNγR1 Deficiency (MSMD, AR) - STAT 2 Deficiency, AR - IFNγR2 Deficiency (MSMD, AR) - TBK1 Deficiency (Herpes Simplex Encephalitis-HSE, AD) - IL12/IL23RB1 Deficiency (MSMD, AR) - TLR3 Deficiency (Herpes Simplex Encephalitis-HSE, AD/AR) - IL-12p40 (MSMD, AR) - TRAF3 Deficiency (Herpes Simplex Encephalitis-HSE, AD) - IL-17F Deficiency (Chronic Mucocutaneous Candidiasis-CMC, AD) TRIF Deficiency (Herpes Simplex Encephalitis-HSE, AD/AR) - IL-17RA Deficiency (Chronic Mucocutaneous Candidiasis-CMC, AR) Tyk2 Deficiency (MSMD, AR) - IL-17RC Deficiency (Chronic Mucocutaneous Candidiasis-CMC, AR) UNC93B1 Deficiency (Herpes Simplex Encephalitis-HSE, AR) - IRAK-4 Deficiency, AR |
| Autoinflammatory diseases | - ADAM17 Deficiency, AR - NLRC4 GOF (Familial Cold Autoinflammatory Syndrome 4, AD) - CARD14 Deficiency (CARD14 Mediated Psoriasis-CAMPS, AD) - NOD2/CARD15 Deficiency (Blau Syndrome, AD) - COPA Deficiency (Autoimmune Interstitial lung, joint and kidney Disease, AD) - PLCG2 Deficiency (Familial Cold Autoinflammatory Syndrome 3-PLAID, AD) - IL-1RN Deficiency (DIRA, AR) - PLCG2 Deficiency (APLAID Syndrome, AD) - IL-36RN Deficiency (DITRA, AR) - PLCG2 Deficiency (PLAID Syndrome, AD) - LPIN2 Deficiency (Majeed Syndrome, AR) - PSMB8 Deficiency (CANDLE Syndrome, AR) - MEFV Deficiency (Familial Mediterranean Fever-FMF, AD/AR) - PSTPIP1 Deficiency (PAPA Syndrome, AD) - MVK Deficiency (Hyper IgD Syndrome, AR) - SH3BP2 Deficiency (Cherubism, AD) - NLRP12 Deficiency (Familial Cold Autoinflammatory Syndrome 2, AD) - SLC29A3 Deficiency (AR) - NLRP3 Deficiency (Familial Cold Autoinflammatory Syndrome 1, AD) - TNFRSF1 Deficiency (TNF Receptor-Associated Periodic Syndrome-TRAPS, AD) - NLRP3/CIAS1/PYPAF1 Deficiency (Muckle-Wells Syndrome, AD) |
| Complement deficiencies | - Atypical Hemolytic Uremic Syndrome, AD - CD46 Deficiency, AD - C1S Deficiency (C1s), AR - CD59 Deficiency, AR - C1QA Deficiency (C1 Inhibitor), AD - CFB Deficiency (Factor B), AD - C1QB Deficiency (C1q A, B and C, AR) - CFD Deficiency (Factor D), AR - C1QC Deficiency (C1r), AR - CFI Deficiency (Factor I), AR - C2 Deficiency, AR - CFH Deficiency (Factor H), AR/AD - C3 Deficiency (GOF, AD) - CFP Deficiency (Properdin), XL - C3 Deficiency (LOF, AR) - DFHR 1 to 5 Deficiencies (Factor H-related Protein, AD/AR) - C4 Deficiency (A and B, AR) - FCN3 Deficiency (Ficolin 3), AR - C5 Deficiency, AR - ITGAM Deficiency (Complement Receptor 3-CR3, AR) - C6 Deficiency, AR - MASP2 Deficiency, AR - C7 Deficiency, AR - THBD Deficiency (Thrombomodulin), AD - C8 Deficiency (a, b and γ, AR) |
| Bone marrow failure | - DKCA6, AD - Fanconi Anemia Type S, AR - Fanconi Anemia Type D1, AR - Fanconi Anemia Type J, AR - Coats plus syndrome due to CTC1 deficiency, AR - DKCX1, XL - Fanconi Anemia Type Q, AR - Fanconi Anemia Type A, AR - Fanconi Anemia Type B, XLR - Fanconi Anemia Type C, AR - Fanconi Anemia Type D2, AR - Fanconi Anemia Type E, AR - Fanconi Anemia Type F, AR - Fanconi Anemia Type I, AR - Fanconi Anemia Type L, AR - Fanconi Anemia Type M, AR - Fanconi Anemia Type V, AR - EVI1, MECOM deficiency, AD - DKCB2, AR - DKCB1, AR - Fanconi Anemia Type N, AR - DKCB6, AR - Fanconi Anemia Type R, AR - Fanconi Anemia Type O, AR - Fanconia Anemia Type W, AR - DKCA4, AD - DKCB5, AR - SAMD9 (GOF), AD GOF - Ataxia Pancytopenia Syndrome, AD - Fanconi Anemia Type P, AR - BMFS1 (SRP72-deficiency), AD - Coats plus syndrome due to STN1 deficiency, AR - DKCA1, AD - DKCA2, AD - DKCB4, AR - DKCA3, AD - BMFS5, AD - Fanconi Anemia Type T, AR - DKCB3, AR - Fanconi Anemia Type U, AR - Fanconi Anemia Type G, AR |
| Phenocopies of primary immunodeficiencies | - AIRE AutoAB (IL-17/IL-22) - KRAS (RALD, GOF) - AutoAB (Complement Factor H) - NLRP3 (Cryopyrinopathy) - AutoAB (CI Inhibitor) - NRAS (RALD, GOF) - AutoAB (GM-CSF) - TNFRSF6 (ALPS-SFAS) - AutoAB (IFN gamma) |
