# Supplementary material for: Severity of SARS-CoV-2 infection in children with inborn errors of immunity (primary immunodeficiencies): a systematic review
Source: Allergy Asthma Clin Immunol. 2023 Aug 9;19:69. doi: 10.1186/s13223-023-00831-1 (PMC10413516; doi:10.1186/s13223-023-00831-1)
Supplement: Supplementary file 2 — Additional file 2: Table S3. Summary of the characteristics of the included studies with evidence on IEIs and COVID-19 in pediatric patients (n = 116 studies), 2020-2022. [file 13223_2023_831_MOESM2_ESM.docx]

Table Supplementary 3: Summary of the characteristics of the included studies with evidence on IEIs and COVID-19 in pediatric patients (n = 116 studies), 2020-2022

| **Author, year, study location** | **Study design, setting** | **Age (months)^a^** | **Male, n (%)** | **Ethnicity^b^** | **Identified IEIs** | **Main genetic cause of IEIs** | **Other potential modifiers in immunity-related pathways**  **OR**  **Specific allele change** | **IEIs mode of inheritance** | **COVID-19 severity**  **If experienced MIS-C**  **AND**  **Comorbidities, n** | **Laboratory findings** | **IEIs treatment at SARS-CoV-2 infection, n** | **Admitted to ICU, n** | **Mechanical ventilation, n** | **ARDS, n** | **Assessment of study risk of bias (tool used; finding); and treatment outcome** |
| --- | --- | --- | --- | --- | --- | --- | --- | --- | --- | --- | --- | --- | --- | --- | --- |
| **IUIS IEIs category: Cellular and humoral immunodeficiencies (Group I)** | | | | | | | | | | | | | | | |
| Gelzo et al. 2022 ([62](#_ENREF_62)), Italy | Retrospective cohort, single centre | 60, 144 and 60 | 2 (66.7) | 3 Whites (Caucasians) | 3 CID (DOCK8 deficiencies) | DOCK8 deficiency (n=3) | c.3031C>T p (n=1)  c.689G>A (n=1)  c.1000T>C (n=1) | 3 ARs | 3 Not reported  3 Yes MIS-C  AND  3 No comorbidities | 3 Not reported | 3 Not reported | 3 Not reported | 3 Not reported | 3 Not reported | (NOS, 7)  3 outcome was not reported |
| Vagrecha et a. 2022 ([125](#_ENREF_125)), United States | Retrospective cohort, single centre | 48, 120 and 24 | 3 (100) | 2 Hispanics  1 Black | 3 CID (DOCK8 deficiencies) | DOCK8 deficiency (n=3) | c.2695C>T (n=1)  c.2060C>T (n=1)  c.1193G>A (n=1) | 3 ARs | 3 Not reported  3 Yes MIS-C  AND  3 No comorbidities | 3 Not possible to extract | 3 Not possible to extract | 3 Not possible to extract | 3 Not possible to extract | 3 Not possible to extract | (NOS, 7)  3 outcome was not reported |
| Marcus et al. 2021 ([92](#_ENREF_92)), Israel | Retrospective cohort, multi-centre | 3 and 180 | 0 (0) | 2 Jews | 2 CIDs (RelB deficiencies) | RELB deficiencies (n=2) | 2 NOPMiIrPwD | 2 ARs | 1 Asymptomatic  1 Mild  2 No MIS-C  AND  2 No comorbidities | 2 Not reported | 1 IVIG | 0 | 0 | 0 | (NOS, 7)  2 survived |
| Marcus et al. 2022 ([91](#_ENREF_91)), Israel | Retrospective case report, multi-centre | 192 | 0 (0) | 1 Jewish | 1 CID (RelB deficiency) | RELB deficiency (n=1) | 1 NOPMiIrPwD | 1 AR | 1 Mild  1 Not reported  AND  1 No comorbidities | 1 Not reported | 1 Casirivimab/imdevimab | 0 | 0 | 0 | (Modified NOS, high)  1 survived |
| Abolhassani et al. 2022 ([19](#_ENREF_19)), Iran | Prospective cohort, single centre | 108 | 1 (100) | 1 Persian | 1 CID (PIGA deficiency) | PIGA deficiency (n=1) | IFN pathway genes: IFNA8 (n=1)  IL-1 activation pathway genes: TNFRSF11A (n=1) | 1 X-linked | 1 Severe  1 No MIS-C  AND  1 Not reported | 1 Low serum IgA and IgM levels | 1 Antibiotics  1 IVIG  1 Convalescent plasma  1 TPN | 1 | 0 | 0 | (NOS, 8)  1 survived |
| Abolhassani et al. 2022 ([19](#_ENREF_19)), Iran | Prospective cohort, single centre | 108 | 1 (100) | 1 Persian | 1 CID (Ikaros deficiency) | IKZF1 (n=1) | Lymphocyte development/epigenetic: CLEC16A (n=1)  I IFN pathway genes: IRF2BPL (n=1)  IL-1 activation pathway genes: TNFRSF11A (n=1) | 1 AD | 1 Critical  1 No MIS-C  AND  1 Not reported | 1 Not reported | 1 Antibiotics  1 IVIG  1 Antiplatelets  1 ACEIs  1 Amiodarone  1 TPN  1 CPR | 1 | 1 | 1 | (NOS, 8)  1 died (not reported if COVID-19-related) |
| Karakoc Aydiner et al. 2022 ([73](#_ENREF_73)), Turkey | Prospective cohort, multi-centre | 55 | 1 (100) | 1 White (Caucasian) | 1 CID (MODP1 deficiency) | MODP1 (n=1) | 1 NOPMiIrPwD | 1 AR | 1 Mild  1 No MIS-C  AND  1 Atopic dermatitis | 1 High CRP  1 High D-dimer | 1 No treatment | 0 | 0 | 0 | (NOS, 7)  1 survived |
| Conti et al. 2022 ([46](#_ENREF_46)), Italy | Retrospective cohort, single centre | 108 and 156 | 1 (50) | 2 Whites (Caucasians) | 2 CIDs (Idiopathic CD4+ lymphocytopenia) | 2 NSGDwR | 2 NOPMiIrPwD | 2 Unknowns | 2 Mild  2 No MIS-C  AND  1 Truncus arteriosus | 2 Not reported | 1 Casirivimab/imdevimab | 0 | 0 | 0 | (NOS, 8)  2 survived |
| Giardino et al. 2022 ([63](#_ENREF_63)), Italy | Retrospective cohort, multi-centre | 36 | 1 (100) | 1 White (Caucasian) | 1 CID (Idiopathic CD4+ lymphocytopenia) | 1 NSGDwR | 1 NOPMiIrPwD | 1 Unknown | 1 Mild  1 No MIS-C  AND  1 CHD  1 Epilepsy  1 Developmental delay | 1 Not reported | 1 No treatment | 0 | 0 | 0 | (NOS, 7)  1 survived |
| Delavari et al. 2021 ([48](#_ENREF_48)), Iran | Prospective cohort, multi-centre | 144 | 1 (100) | 1 Persian | 1 CID (MST1 deficiency) | STK4 (n=1) | 1 NOPMiIrPwD | 1 AR | 1 Critical  1 Yes MIS-C  AND  1 Meningitis  1 Cardiorespiratory arrest  1 AIHA  1 ITP  1 Seizure  1 Neurological disorder | 1 Lymphopenia  1 High ESR  1 High CRP | 1 Acyclovir  1 Antibiotics  1 Steroids | 1 | 1 | 1 | (NOS, 7)  1 died (COVID-19-related) |
| Giardino et al. 2022 ([63](#_ENREF_63)), Italy | Retrospective cohort, multi-centre | 180 | 1 (100) | 1 White (Caucasian) | 1 CID (NK reduction) | 1 NSGDwR | 1 NOPMiIrPwD | 1 Unknown | 1 Mild  1 No MIS-C  AND  1 Ulcerative colitis | 1 Not reported | 1 No treatment | 0 | 0 | 0 | (NOS, 7)  1 survived |
| Ramanathan et al. 2022 ([111](#_ENREF_111)), United Kingdom | Retrospective case report, single centre | 156 | 0 (0) | 1 White (Caucasian) | 1 CID (MHC class II deficiency) | RFXANK (n=1) | c.362A>T p (n=1) | 1 AR | 1 Mild  1 No MIS-C  AND  1 Post HSCT | 1 Not reported | 1 Valganciclovir  1 HSCT  1 Antibiotics  1 Remdesivir  1 Transfer of maternal CD45RO^+^ memory cells  1 Regdanvimab | 0 | 0 | 0 | (Modified NOS, high)  1 survived |
| Marcus et al. 2021 ([92](#_ENREF_92)), Israel | Retrospective cohort, multi-centre | 204 | 1 (100) | 1 Jew | 1 CID (CD40 deficiency) | CD40 (TNFRSF5) (n=1) | 1 NOPMiIrPwD | 1 X-linked | 1 Mild  1 No MIS-C  AND  1 Hypogammaglobulinemia | 1 Not reported | 1 IVIG | 0 | 0 | 0 | (NOS, 7)  1 survived |
| Mohanty et al. 2021 ([99](#_ENREF_99)), India | Retrospective case report, single centre | 36 | 1 (100) | 1 Indian | 1 CID (CD40 deficiency) | CD40 (TNFRSF5) (n=1) | CD40c.366_366delinTCA (n=1) | 1 X-linked | 1 Asymptomatic  1 No MIS-C  AND  1 No comorbidities | 1 Low serum IgG, IgA and IgM levels  1 Lymphocytosis | 1 IVIG | 0 | 0 | 0 | (Modified NOS, high)  1 survived |
| Meyts et al. 2021 ([95](#_ENREF_95)), 10 countries | Retrospective cohort, multi-centre | Age group: 156-216 (n=1) | 0 (0) | 1 White (Caucasian) | 1 CID (ZAP70 deficiency) | ZAP70 deficiency (n=1) | 1 NOPMiIrPwD | 1 AR | 1 Mild  1 No MIS-C  AND  1 CLD  1 B-cell lymphoma | 1 Not reported | 1 No treatment | 0 | 0 | 0 | (NOS, 8)  1 survived |
| Vagrecha et a. 2022 ([125](#_ENREF_125)), United States | Retrospective cohort, single centre | 24 | 1 (100) | 1 White (Caucasian) | 1 CID (ZAP70 deficiency) | ZAP70 deficiency (n=1) | c.1153C>T (n=1) | 1 AR | 1 Not reported  1 Yes MIS-C  AND  1 No comorbidities | 1 Not possible to extract | 1 Not possible to extract | 1 Not possible to extract | 1 Not possible to extract | 1 Not possible to extract | (NOS, 7)  1 outcome was not reported |
| Abolhassani et al. 2022 ([19](#_ENREF_19)), Iran | Prospective cohort, single centre | 84 | 1 (100) | 1 Persian | 1 CID (subcategory was not reported) | 1 NSGDwR | IFN pathway genes: STAT4 (n=1) | 1 Unknown | 1 Critical  1 No MIS-C  AND  1 Not reported | 1 Not reported | 1 Antibiotics  1 IVIG  1 Antiplatelets  1 ACEIs  1 Amiodarone  1 TPN  1 CPR | 1 | 1 | 1 | (NOS, 8)  1 died (not reported if COVID-19-related) |
| Conti et al. 2022 ([46](#_ENREF_46)), Italy | Retrospective cohort, single centre | 144, 180, 192 and 84 | 0 (0) | 4 Whites (Caucasians) | 4 CIDs (subcategories were not reported) | 4 NSGDwR | 4 NOPMiIrPwD | 4 Unknowns | 3 Mild  1 Moderate  4 No MIS-C  AND  1 CLDs  1 Burkitt lymphoma  1 Autism  1 Neurodevelopmental delay | 4 Not reported | 1 Sotrovimab  1 Casirivimab/imdevimab  1 IVIG  1 SCIg  1 Antibiotics | 0 | 0 | 0 | (NOS, 8)  4 survived |
| Delavari et al. 2021 ([48](#_ENREF_48)), Iran | Prospective cohort, multi-centre | 11 | 1 (100) | 1 Persian | 1 CID (subcategory was not reported) | 1 NSGDwR | 1 NOPMiIrPwD | 1 Unknown | 1 Moderate  1 Yes MIS-C  AND  1 Autoimmune hypothyroidism | 1 Lymphopenia  1 High ESR  1 High CRP | 1 Antibiotics  1 IVIG  1 Hydroxychloroquine | 1 | 0 | 0 | (NOS, 7)  1 survived |
| Giardino et al. 2022 ([63](#_ENREF_63)), Italy | Retrospective cohort, multi-centre | 195 | 1 (100) | 1 White (Caucasian) | 1 CID (subcategory was not reported) | 1 NSGDwR | 1 NOPMiIrPwD | 1 Unknown | 1 Asymptomatic  1 No MIS-C  AND  1 Lung nodule  1 Cognitive disability | 1 Not reported | 1 No treatment | 0 | 0 | 0 | (NOS, 7)  1 survived |
| [Goudouris](https://scholar.google.com/citations?user=nyuqBh4AAAAJ&hl=en&oi=sra) et al. 2021 ([65](#_ENREF_65)), Brazil | Retrospective cohort, multi-centre | 76, 212, 6, 124 and 175 | 0 (0) | 5 Hispanics | 5 CIDs (subcategories were not reported) | 5 NSGDwR | 5 NOPMiIrPwD | 5 ARs | 2 Asymptomatic  2 Mild  1 Moderate  5 No MIS-C  AND  3 Obesity  2 Down syndrome  1 CHD | 5 Not reported | 1 IVIG  4 No treatment | 1 | 0 | 0 | (NOS, 8)  5 survived |
| Karimi et al. 2021 ([74](#_ENREF_74)), Iran | Retrospective cohort, multi- centre | 1, 13, 33, 62, 86, 6, 51, 16, and 19 | 6 (66.7) | 9 Persians | 9 CIDs (subcategories were not reported) | 9 NSGDwR | 9 NOPMiIrPwD | 9 ARs | 5 Mild  4 Moderate  9 Not reported    AND  4 Cardiorespiratory arrest  2 Septic shock  2 DIC  1 Pulmonary haemorrhage  1 Seizures  1 Bronchiectasis  1 Heart disease  1 Hydrocephalus  1 Renal disorder  1 Allergic colitis  1 Autoimmunity | 9 Not possible to extract | 9 Antibiotics  8 IVIG  4 Chloroquine  1 Valganciclovir  1 Naproxen  1 Antiplatelets  1 Acyclovir | 4 | 4 | 4 | (NOS, 6)  5 survived  4 died (COVID-19-related) |
| Karakoc Aydiner et al. 2022 ([73](#_ENREF_73)), Turkey | Prospective cohort, multi-centre | 199, 100, 147, and 163 | 4 (100) | 4 Whites (Caucasians) | 4 CIDs (subcategories were not reported) | 4 NSGDwR | 4 NOPMiIrPwD | 4 Unknowns | 3 Mild  1 Moderate  3 No MIS-C  1 Yes MIS-C  AND  1 Multi-organ failure  1 Bronchiectasis  1 Allergic rhinitis | 1 Neutropenia  1 Leukopenia  2 High CRP  1 High LDH  1 High D-dimer  1 High Troponine I  1 Hypoalbuminemia | 4 Antibiotics  3 IVIG  1 SCIg | 1 | 1 | 1 | (NOS, 7)  3 survived  1 died (COVID-19-related) |
| Meyts et al. 2021 ([95](#_ENREF_95)), 10 countries | Retrospective cohort, multi-centre | Age groups: 0-24 (n=4), 36-144 (n=5), and 156-216 (n=2) | 7 (70) | 9 Whites (Caucasians)  2 Hispanics | 11 CIDs (subcategories were not reported) | 11 NSGDwR | 11 NOPMiIrPwD | 11 Unknowns | 3 Asymptomatic  8 Mild  11 No MIS-C  AND  3 CLDs  2 CHDs  3 HLHs  2 Eczema  2 Down syndrome  1 B-cell lymphoma  1 Post HSCT  1 Heart disease  1 Gastrointestinal disease  2 Tracheostomy  2 Chronic ventilations  1 Cognitive disability  1 Sepsis | 11 Not reported | 11 Antibiotics  2 Steroids  3 Antivirals  6 IVIG  1 Heparin  1 Chloroquine  1 Lopinavir/ritonavir  1 Pentamidine  1 Thrombopoietin receptor agonist  1 Remdesivir  1 Convalescent plasma  1 Tocilizumab  1 Granulocyte colony-stimulating factor  4 Oxygen supplementation  1 Antifungals  1 Gene therapy | 2 | 2 | 2 | (NOS, 8)  9 survived  2 still in ICU (not COVID-19-related) |
| Moazzen et al. 2021 ([98](#_ENREF_98)), Iran | Retrospective cohort, single centre | 48 | 1 (100) | 1 Persian | 1 CID (subcategory was not reported) | 1 NSGDwR | 1 NOPMiIrPwD | 1 Unknown | 1 Mild  1 No MIS-C  AND  1 CLD | 1 Not reported | 1 Antibiotics  1 Diuretics  1 Sildenafil  1 IVIG  1 Steroids | 1 | 1 | 1 | (NOS, 6)  1 died (not COVID-19-related) |
| Peeler et al. 2021 ([104](#_ENREF_104)), United States | Retrospective case report, single centre | 22 | 0 (0) | 1 White (Caucasian) | 1 CID (subcategory was not reported) | 1 NSGDwR | 1 NOPMiIrPwD | 1 Unknown | 1 Severe  1 Yes MIS-C  AND  1 Hypothyroidism 1 Prematurity  1 Tracheostomy  1 69 XXX triploidy  1 Polymicrogyria  1 Epilepsy  1 Repaired atrial septal defect  1 *Enterococcus faecalis* coinfection  1 *Pseudomonas aeruginosa* coinfection  1 Hypotension  1 Tachycardia  1 Multi-organ failure | 1 Raised liver enzymes  1 High LDH  1 High ferritin  1 Lymphopenia  1 Elevated PT  1 Elevated PTT  1 High D-dimer  1 High fibrinogen  1 High interleukin-2  1 High interleukin-6  1 High interleukin-10 | 1 Oxygen supplementation  1 IV inotropes  1 Antibiotics  1 Metronidazole  1 Nitric oxide  1 Sedation  1 Neuromuscular blockade  1 Hydroxychloroquine  1 Remdesivir  1 Steroids  1 Anakinra | 1 | 1 | 1 | (Modified NOS, high)  1 survived |
| Conti et al. 2022 ([46](#_ENREF_46)), Italy | Retrospective cohort, single centre | 72 | 1 (100) | 1 White (Caucasian) | 1 SCID (Common gamma chain SCID, CD132 deficiency) | IL2RG (n=1) | 1 NOPMiIrPwD | 1 X-linked | 1 Mild  1 No MIS-C  AND  1 Post HSCT | 1 Not reported | 1 No treatment | 0 | 0 | 0 | (NOS, 8)  1 survived |
| [Goudouris](https://scholar.google.com/citations?user=nyuqBh4AAAAJ&hl=en&oi=sra) et al. 2021 ([65](#_ENREF_65)), Brazil | Retrospective cohort, multi-centre | 72 and 99 | 2 (100) | 2 Hispanics | 2 SCIDs (Common gamma chain SCID, CD132 deficiency) | IL2RG (n=2) | 2 NOPMiIrPwD | 2 X-linked | 2 Asymptomatic  2 No MIS-C  AND  2 No comorbidities | 2 Not reported | 2 No treatment | 0 | 0 | 0 | (NOS, 8)  2 survived |
| Pieniawska-Śmiech et al. 2021 ([108](#_ENREF_108)), Poland | Retrospective cohort, single centre | 72 | 1 (100) | 1 White (Caucasian) | 1 SCID (Common gamma chain SCID, CD132 deficiency) | IL2RG (n=1) | 1 NOPMiIrPwD | 1 X-linked | 1 Mild  1 No MIS-C  AND  1 No comorbidities | 1 Normal laboratory parameters | 1 No treatment | 0 | 0 | 0 | (NOS, 8)  1 survived |
| van Oers et al. 2021 ([126](#_ENREF_126)), United States | Retrospective case report, single centre | An infant (0 to 2) | 1 (100) | 1 White (Caucasian) | 1 SCID (Common gamma chain SCID, CD132 deficiency) | IL2RG (n=1) | c.758-2A>G (n=1) | 1 X-linked | 1 Moderate  1 No MIS-C  AND  1 Hepatitis | 1 Raised liver enzymes  1 High D-dimer  1 Elevated PTT  1 High ferritin  1 Raised procalcitonin  1 High interleukin-10  1 Lymphopenia  1 Neutropenia | 1 HSCT  1 Convalescent plasma  1 IVIG  1 Steroids | 0 | 0 | 0 | (Modified NOS, high)  1 survived |
| Abolhassani et al. 2022 ([19](#_ENREF_19)), Iran | Prospective cohort, single centre | 24 | 1 (100) | 1 Persian | 1 SCID (IL7Ra deficiency) | IL7Ra deficiency (n=1) | Lymphocyte development/epigenetic: KMT2D (n=1) | 1 AR | 1 Critical  1 No MIS-C  AND  1 Not reported | 1 Not reported | 1 IVIG  1 Antibiotics  1 TPN  1 CPR | 1 | 1 | 1 | (NOS, 8)  1 died (not reported if COVID-19-related) |
| Hariharan et al. 2022 ([69](#_ENREF_69)), India | Retrospective case report, single centre | 60 | 0 (0) | 1 Indian | 1 SCID (IL7Ra deficiency) | IL7Ra deficiency (n=1) | IL−7Ra (n=1) | 1 AR | 1 Mild  1 Yes MIS-C  AND  1 Tuberculosis infection  1 Pallor  1 Aneurysm (left main coronary artery) | 1 High ESR  1 Leukocytosis  1 Hyponatremia  1 Hypoalbuminemia  1 Low serum IgM and IgA levels  1 Reduced T-cell markers  1 Raised liver enzymes | 1 Antitubercular drugs  1 IVIG  1 Anticoagulant | 0 | 0 | 0 | (Modified NOS, high)  1 survived |
| Karakoc Aydiner et al. 2022 ([73](#_ENREF_73)), Turkey | Prospective cohort, multi-centre | 50 | 0 (0) | 1 White (Caucasian) | 1 SCID (IL7Ra deficiency) | IL7Ra deficiency (n=1) | 1 NOPMiIrPwD | 1 AR | 1 Asymptomatic  1 No MIS-C  AND  1 Post HSCT | 3 Neutropenia  1 High LDH | 1 No treatment | 0 | 0 | 0 | (NOS, 7)  1 survived |
| Al-Saud et al. 2021 ([31](#_ENREF_31)), Saudi Arabia | Retrospective case report, single centre | 8 | 1 (100) | 1 Arab | 1 SCID (JAK3 deficiency) | JAK3 deficiency (n=1) | Cys227Profs*49 and Arg-103Cys (n=1) | 1 AR | 1 Moderate  1 No MIS-C  AND  1 *Pneumocystis jirovecii* coinfection  1 *Candida albicans* coinfection | 1 Lymphopenia  1 Low T cells  1 Low NK cells | 1 IVIG  1 Antibiotics  1 Steroids  1 Favipiravir  1 Heparin  1 Zinc  1 Antifungals  1 Oxygen supplementation | 1 | 1 | 1 | (Modified NOS, high)  1 survived |
| Conti et al. 2022 ([46](#_ENREF_46)), Italy | Retrospective cohort, single centre | 216 | 0 (0) | 1 White (Caucasian) | 1 SCID (JAK3 deficiency) | JAK3 deficiency (n=1) | 1 NOPMiIrPwD | 1 AR | 1 Mild  1 No MIS-C  AND  1 Post HSCT | 1 Not reported | 1 No treatment | 0 | 0 | 0 | (NOS, 8)  1 survived |
| Zamperlini-Netto et al. 2021 ([131](#_ENREF_131)), Brazil | Retrospective case report, single centre | 17 | 0 (0) | 1 White (Caucasian) | 1 SCID (JAK3 deficiency) | JAK3 deficiency (n=1) | 1 NOPMiIrPwD | 1 AR | 1 Mild  1 No MIS-C  AND  1 Adenitis due to tuberculosis vaccine  1 Post HSCT | 1 Not reported | 1 Antitubercular drugs  1 Antibiotics  1 Steroids  1 Rituximab  1 Cyclosporin  1 Hydroxychloroquine  1 IVIG | 0 | 0 | 0 | (Modified NOS, high)  1 survived |
| Delavari et al. 2021 ([48](#_ENREF_48)), Iran | Prospective cohort, multi-centre | 6 | 1 (100) | 1 Persian | 1 SCID (Omenn syndrome) | PAX1 deficiency (n=1) | 1 NOPMiIrPwD | 1 AR | 1 Moderate  1 Yes MIS-C  AND  1 Heart failure  1 Adenitis due to tuberculosis vaccine  1 Seizures | 1 Low WBCs  1 Lymphopenia  1 Thrombocytosis  1 High ESR  1 High CRP | 1 Antitubercular drugs  1 Vitamin B6  1 Antibiotics | 1 | 1 | 1 | (NOS, 7)  1 died (not COVID-19-related) |
| Moazzen et al. 2021 ([98](#_ENREF_98)), Iran | Retrospective cohort, single centre | 3 | 1 (100) | 1 Persian | 1 SCID (Omenn syndrome) | PAX1 deficiency (n=1) | 1 NOPMiIrPwD | 1 AR | 1 Moderate  1 No MIS-C  AND  1 Cardiomegaly  1 Seizure | 1 Lymphopenia  1 Elevated cardiac enzymes | 1 IV inotropes  1 Diuretics  1 Antibiotics  1 Antiepileptics  1 IVIG | 1 | 1 | 1 | (NOS, 6)  1 died (COVID-19-related) |
| Gabryszewski et al. 2021 ([60](#_ENREF_60)), United States | Retrospective case report, single centre | 14 | 0 (0) | 1 White (Caucasian) | 1 SCID (DCLRE1C (Artemis) deficiency) | DCLRE1C (n=1) | c.632G>C (n=1) | 1 AR | 1 Mild  1 No MIS-C  AND  1 No comorbidities | 1 Low NK cells  1 Lymphopenia  1 Near-absent B cells  1 Elevated CD4-to-CD8 ratio  1 Low serum IgA, IgG and IgE levels | 1 No treatment | 0 | 0 | 0 | (Modified NOS, high)  1 survived |
| Vagrecha et a. 2022 ([125](#_ENREF_125)), United States | Retrospective cohort, single centre | 96 | 0 (0) | 1 Hispanic | 1 SCID (DCLRE1C (Artemis) deficiency) | DCLRE1C (n=1) | c.1733A>G (n=1) | 1 AR | 1 Not reported  1 Yes MIS-C  AND  1 No comorbidities | 1 Not possible to extract | 1 Not possible to extract | 1 Not possible to extract | 1 Not possible to extract | 1 Not possible to extract | (NOS, 7)  1 outcome was not reported |
| Abolhassani et al. 2022 ([19](#_ENREF_19)), Iran | Prospective cohort, single centre | 5 | 1 (100) | 1 Persians | 1 SCID (PIGA deficiency) | PIGA deficiency (n=1) | Lymphocyte development/epigenetic: TCF3 (n=1) | 1 AR | 1 Critical  1 No MIS-C  AND  1 Not reported | 1 Low serum IgA, IgM and IgG levels | 1 IVIG  1 Antibiotics  1 TPN  1 CPR  1 Steroids  1 Vitamin B6 | 1 | 1 | 1 | (NOS, 8)  1 died (not reported if COVID-19-related) |
| Castano-Jaramillo et al. 2021 ([40](#_ENREF_40)), Mexico | Retrospective cohort, multi-centre | 5 | 1 (100) | 1 Hispanic | 1 SCID (CD3D deficiency) | CD3D deficiency (n=1) | 1 NOPMiIrPwD | 1 AR | 1 Moderate  1 No MIS-C  AND  1 GERD  1 Myocarditis | 1 Normal laboratory parameters | 1 IVIG | 0 | 0 | 0 | (NOS, 8)  1 survived |
| Conti et al. 2022 ([46](#_ENREF_46)), Italy | Retrospective cohort, single centre | 36 and 24 | 2 (100) | 2 Whites (Caucasians) | 2 SCIDs (ADA deficiencies) | ADA deficiency (n=2) | 2 NOPMiIrPwD | 2 ARs | 1 Mild  1 Moderate  2 No MIS-C  AND  1 AIHA  1 Hypothyroidism | 2 Not reported | 1 Sotrovimab | 0 | 0 | 0 | (NOS, 8)  2 survived |
| Giardino et al. 2022 ([63](#_ENREF_63)), Italy | Retrospective cohort, multi-centre | 33 | 0 (0) | 1 White (Caucasian) | 1 SCID (NBAS deficiency) | NBAS deficiency (n=1) | 1 NOPMiIrPwD | 1 AR | 1 Moderate  1 No MIS-C  AND  1 Autoimmune enteropathy  1 Hepatitis  1 Ichthyosiform dermatosis  1 Congenital hypothyroidism  1 Growth hormone deficiency  1 Developmental delay  1 Pulmonary hypertension  1 Nodules  1 Interstitial lung disease | 1 Not reported | 1 Acyclovir  1 IVIG  1 Dupilumab  1 Steroids | 0 | 0 | 0 | (NOS, 7)  1 survived |
| [Goudouris](https://scholar.google.com/citations?user=nyuqBh4AAAAJ&hl=en&oi=sra) et al. 2021 ([65](#_ENREF_65)), Brazil | Retrospective cohort, multi-centre | 42 | 1 (100) | 1 Hispanic | 1 SCID (SCID-class II HLA) | SCID-class II HLA (n=1) | 1 NOPMiIrPwD | 1 AR | 1 Mild  1 No MIS-C  AND  1 Bronchiectasis | 1 Not reported | 1 No treatment | 0 | 0 | 0 | (NOS, 8)  1 survived |
| Vagrecha et a. 2022 ([125](#_ENREF_125)), United States | Retrospective cohort, single centre | 24 | 1 (100) | 1 Black | 1 SCID (RAG1 deficiency) | RAG1 (n=1) | c.2237T>G (n=1) | 1 AR | 1 Not reported  1 Yes MIS-C  AND  1 No comorbidities | 1 Not possible to extract | 1 Not possible to extract | 1 Not possible to extract | 1 Not possible to extract | 1 Not possible to extract | (NOS, 7)  1 outcome was not reported |
| Babaei et al. 2022 ([34](#_ENREF_34)), Iran | Retrospective cohort, single centre | 5 | 0 (0) | 1 Persian | 1 SCID (subcategory was not reported) | 1 NSGDwR | 1 NOPMiIrPwD | 1 AR | 1 Moderate  1 No MIS-C  AND  1 Adenitis due to tuberculosis vaccine  1 Dilated cardiomyopathy  1 Ventricular septal defect  1 Seizures | 1 Not reported | 1 IVIG  1 Acyclovir  1 Antibiotics 1 Antifungals  1 Steroids | 1 | 1 | 1 | (NOS, 7)  1 died (not COVID-19-related) |
| Delavari et al. 2021 ([48](#_ENREF_48)), Iran | Prospective cohort, multi-centre | 10, 20 and 8 | 2 (66.7) | 3 Persians | 3 SCIDs (subcategories were not reported) | 3 NSGDwR | 3 NOPMiIrPwD | 3 ARs | 3 Severe  3 Yes MIS-C  AND  3 Not reported  1 Adenitis due to tuberculosis vaccine | 2 Low WBCs  3 Lymphopenia  3 Low Hb  3 Thrombocytopenia  3 High ESR  2 Low CD3, CD4 and CD8 counts | 3 Antibiotics  3 IVIG  1 Hydroxychloroquine | 3 | 3 | 3 | (NOS, 7)  3 died (COVID-19-related) |
| Giardino et al. 2022 ([63](#_ENREF_63)), Italy | Retrospective cohort, multi-centre | 180 | 0 (0) | 1 White (Caucasian) | 1 SCID (subcategory was not reported) | 1 NSGDwR | 1 NOPMiIrPwD | 1 AR | 1 Asymptomatic  1 No MIS-C  AND  1 CLD  1 Cerebral atrophy  1 Arthritis  1 Scoliosis  1 Cognitive disability | 1 Not reported | 1 HSCT | 0 | 0 | 0 | (NOS, 7)  1 survived |
| [Goudouris](https://scholar.google.com/citations?user=nyuqBh4AAAAJ&hl=en&oi=sra) et al. 2021 ([65](#_ENREF_65)), Brazil | Retrospective cohort, multi-centre | 8, 25, 26 and 158 | 2 (50) | 4 Hispanics | 4 SCIDs (subcategories were not reported) | 4 NSGDwR | 1 NOPMiIrPwD | 4 Unknowns | 3 Asymptomatic  1 Mild  4 No MIS-C  AND  1 Bronchiectasis  3 No comorbidities | 4 Not reported | 4 No treatment | 0 | 0 | 0 | (NOS, 8)  4 survived |
| Karakoc Aydiner et al. 2022 ([73](#_ENREF_73)), Turkey | Prospective cohort, multi-centre | 8 and 7 | 2 (100) | 2 Whites (Caucasians) | 2 SCIDs (subcategories were not reported) | 2 NSGDwR | 2 NOPMiIrPwD | 2 ARs | 1 Mild  1 Critical  2 Yes MIS-C  AND  1 Multi-organ failure | 2 Neutropenia  1 High CRP  1 High LDH  1 High D-dimer | 2 Antibiotics  2 IVIG  1 Antifungals  1 Tacrolimus | 1 | 1 | 1 | (NOS, 7)  1 survived  1 died (COVID-19-related) |
| Karimi et al. 2021 ([74](#_ENREF_74)), Iran | Retrospective cohort, multi- centre | 6.5 and 6 | 1 (50) | 2 Persians | 2 SCIDs (subcategories were not reported) | 2 NSGDwR | 2 NOPMiIrPwD | 2 ARs | 1 Moderate  1 Severe  2 Not reported  AND  1 Septic shock  1 DIC  1 Ventricular septal defect  1 Dilated cardiomyopathy  1 Seizures | 2 Not possible to extract | 2 IVIG  2 Antibiotics  2 Antifungals  1 Ganciclovir  1 Acyclovir | 1 | 1 | 1 | (NOS, 6)  1 survived  1 died (COVID-19-related) |
| Kołtan et al. 2022 ([81](#_ENREF_81)), Poland | Retrospective cohort, multi-centre | 4 Not possible to extract | 4 Not possible to extract | 4 Whites (Caucasians) | 4 SCIDs (subcategories were not reported) | 4 NSGDwR | 4 NOPMiIrPwD | 4 ARs | 1 Asymptomatic  2 Mild  1 Moderate  3 No MIS-C  1 Yes MIS-C  AND  1 Post HSCT  3 Not possible to extract | 4 Not reported | 4 Not possible to extract | 0 | 0 | 0 | (NOS, 7)  4 survived |
| Moazzen et al. 2021 ([98](#_ENREF_98)), Iran | Retrospective cohort, single centre | 8, 8 and 48 | 3 (100) | 3 Persians | 3 SCIDs (subcategories were not reported) | 3 NSGDwR | 3 NOPMiIrPwD | 3 ARs | 2 Moderate  1 Mild  3 No MIS-C  AND  3 No comorbidities | 1 Lymphopenia  1 High CRP  1 Anaemia  1 Thrombocytopenia | 3 Antibiotics  3 IVIG  3 Vitamin C  1 RBCs transfusions  1 Platelet transfusions | 1 | 0 | 0 | (NOS, 6)  3 survived |
| **IUIS IEIs category: Combined immunodeficiencies with associated or syndromic features (Group II)** | | | | | | | | | | | | | | | |
| Conti et al. 2022 ([46](#_ENREF_46)), Italy | Retrospective cohort, single centre | 4, 12, 12, 36, 72, 96, 84, 120, 168 and 192 | 4 (40) | 10 Whites (Caucasians) | 10 Thymic defects with additional congenital anomalies (DiGeorge syndromes) | Large (3Mb) deletion of 22q11.2 (n=10) | 10 NOPMiIrPwD | 10 ADs | 8 Mild  2 Moderate  10 No MIS-C  AND  7 Cardiopathy  1 Evan’s syndrome  1 Percutaneous endoscopic gastrostomy for dysphagia  1 GERD | 10 Not reported | 2 Antibiotics  1 Antiplatelets  1 SCIg  1 Mycophenolate mofetil  1 Vitamin D  1 Iron  1 Steroids  1 Sotrovimab  1 Banlavimib/etesemivab | 0 | 0 | 0 | (NOS, 8)  10 survived |
| Fallatah et al. 2021 ([56](#_ENREF_56)), United States | Retrospective case report, single centre | 156 and 144 | 1 (50) | 2 Hispanics | 2 Thymic defects with additional congenital anomalies (DiGeorge syndromes) | Large (3Mb) deletion of 22q11.2 (n=2) | 2 NOPMiIrPwD | 2 ADs | 2 Asymptomatic  2 No MIS-C  AND  1 Headache  1 Obesity  2 CHDs  1 Ventriculoperitoneal shunt | 1 Hypogammaglobulinemia  1 Lymphopenia  1 Low serum IgM level | 2 No treatment | 0 | 0 | 0 | (Modified NOS, low)  2 survived |
| Giardino et al. 2022 ([63](#_ENREF_63)), Italy | Retrospective cohort, multi-centre | 12, 165, 84, 18, 204, 111, 138, 19, 127, and 182 | 3 (30) | 10 Whites (Caucasians) | 10 Thymic defects with additional congenital anomalies (DiGeorge syndromes) | Large (3Mb) deletion of 22q11.2 (n=10) | 10 NOPMiIrPwD | 10 ADs | 2 Asymptomatic  7 Mild  1 Moderate  10 No MIS-C  AND  2 CHDs  2 Hypoparathyroidism  2 Interventricular defects  2 Cognitive disabilities  1 Autoimmune hypothyroidism  1 Hypocalcaemia  1 Congenital hypothyroidism  1 Autoimmune thyroiditis  1 Hyperthyroidism  1 Developmental delay  1 Minimal left to right interatrial shunt  1 Nephrocalcinosis  1 Obesity  1 Bicuspid aortic valve  1 Left aortic arch | 10 Not reported | 1 Thymus transplantation  1 SCIg  2 Antibiotics  1 Antifungals  1 Acyclovir | 0 | 0 | 0 | (NOS, 7)  10 survived |
| [Goudouris](https://scholar.google.com/citations?user=nyuqBh4AAAAJ&hl=en&oi=sra) et al. 2021 ([65](#_ENREF_65)), Brazil | Retrospective cohort, multi-centre | 8 | 1 (100) | 1 Hispanic | 1 Thymic defect with additional congenital anomalies (DiGeorge syndromes) | Large (3Mb) deletion of 22q11.2 (n=1) | 1 NOPMiIrPwD | 1 AD | 1 Severe  1 No MIS-C  AND  1 CHD  1 Arterial hypertension  1 Bacterial pneumonia | 1 Not reported | 1 IVIG | 1 | 0 | 0 | (NOS, 8)  1 survived |
| Kołtan et al. 2022 ([81](#_ENREF_81)), Poland | Retrospective cohort, multi-centre | 4 Not possible to extract | 4 Not possible to extract | 4 Whites (Caucasians) | 4 Thymic defects with additional congenital anomalies (DiGeorge syndromes) | Large (3Mb) deletion of 22q11.2 (n=4) | 4 NOPMiIrPwD | 4 ADs | 3 Mild  1 Asymptomatic  4 No MIS-C  AND  4 Not possible to extract | 4 Not reported | 4 Not possible to extract | 0 | 0 | 0 | (NOS, 7)  4 survived |
| Marcus et al. 2021 ([92](#_ENREF_92)), Israel | Retrospective cohort, multi-centre | 18 | 1 (100) | 1 Jewish | 1 Thymic defect with additional congenital anomalies (DiGeorge syndromes) | Large (3Mb) deletion of 22q11.2 (n=1) | 1 NOPMiIrPwD | 1 AD | 1 Asymptomatic  1 No MIS-C  AND  1 No comorbidities | 1 Hypogammaglobulinemia  1 Low T-cell receptor excision circles level | 1 IVIG  1 Antibiotics | 0 | 0 | 0 | (NOS, 7)  1 survived |
| Meyts et al. 2021 ([95](#_ENREF_95)), 10 countries | Retrospective cohort, multi-centre | Age group: 0-24 (n=1) | 1 (100) | 1 Hispanic | 1 Thymic defect with additional congenital anomalies (DiGeorge syndromes) | Large (3Mb) deletion of 22q11.2 (n=1) | 1 NOPMiIrPwD | 1 AD | 1 Mild  1 No MIS-C  AND  1 CLD  1 Tracheostomy  1 Chronic ventilation | 1 Not reported | 1 Antibiotics  1 IVIG  1 Oxygen supplementation | 0 | 0 | 0 | (NOS, 8)  1 survived |
| Milito et al. 2021 ([97](#_ENREF_97)), Italy | Retrospective cohort, multi-centre | Age group: <216 (n=10) | 10 Not possible to extract | 10 Whites (Caucasians) | 10 Thymic defects with additional congenital anomalies (DiGeorge syndromes) | Large (3Mb) deletion of 22q11.2 (n=10) | 10 NOPMiIrPwD | 10 ADs | 3 Asymptomatic  6 Mild  1 Severe  10 No MIS-C  AND  10 Not possible to extract | 10 Not reported | 10 Not reported | 0 | 0 | 0 | (NOS, 7)  10 survived |
| Pieniawska-Śmiech et al. 2021 ([108](#_ENREF_108)), Poland | Retrospective cohort, single centre | 18 | 1 (100) | 1 White (Caucasian) | 1 Thymic defect with additional congenital anomalies (DiGeorge syndromes) | Large (3Mb) deletion of 22q11.2 (n=1) | 1 NOPMiIrPwD | 1 AD | 1 Mild  1 No MIS-C  AND  1 No comorbidities | 1 Normal laboratory parameters | 1 No treatment | 0 | 0 | 0 | (NOS, 8)  1 survived |
| Babaei et al. 2022 ([34](#_ENREF_34)), Iran | Retrospective cohort, single centre | 124 | 0 (0) | 1 Persian | 1 DNA repair defect (Ataxia-telangiectasia) | ATM (n=1) | 1 NOPMiIrPwD | 1 AR | 1 Asymptomatic  1 No MIS-C  AND  1 No comorbidities | 1 Not reported | 1 IVIG | 0 | 0 | 0 | (NOS, 7)  1 survived |
| Conti et al. 2022 ([46](#_ENREF_46)), Italy | Retrospective cohort, single centre | 108 and 168 | 1 (50) | 2 Whites (Caucasians) | 2 DNA repair defects (Ataxias-telangiectasias) | ATM (n=2) | 2 NOPMiIrPwD | 2 ARs | 2 Mild  2 No MIS-C  AND  1 No comorbidities | 2 Not reported | 2 SCIg | 0 | 0 | 0 | (NOS, 8)  2 survived |
| Delavari et al. 2021 ([48](#_ENREF_48)), Iran | Prospective cohort, multi-centre | 206 | 1 (100) | 1 Persian | 1 DNA repair defect (Ataxia-telangiectasia) | ATM (n=1) | 1 NOPMiIrPwD | 1 AR | 1 Mild  1 No MIS-C  AND  1 No comorbidities | 1 High ESR  1 High CRP | 1 Antibiotics  1 IVIG | 0 | 0 | 0 | (NOS, 7)  1 survived |
| Esenboga et al. 2021 ([52](#_ENREF_52)), Turkey | Retrospective cohort, single centre | 180 and 120 | 1 (50) | 2 Whites (Caucasians) | 2 DNA repair defects (Ataxias-telangiectasias) | ATM (n=1)  1 NSGDwR | 2 NOPMiIrPwD | 2 ARs | 2 Mild  2 No MIS-C  AND  1 GERD  1 No comorbidities | 2 Lymphopenia  1 Low serum IgG and IgA levels  1 Low serum IgM level | 2 Antibiotics 1 IVIG  1 Favipiravir  1 Antiplatelets | 0 | 0 | 0 | (NOS, 7)  2 survived |
| Gelzo et al. 2022 ([62](#_ENREF_62)), Italy | Retrospective cohort, single centre | 168, 24 and 12 | 2 (66.7) | 3 Whites (Caucasians) | 3 DNA repair defects (Ataxias-telangiectasias) | ATM (n=3) | c.5262G>T (n=1)  c.1595G>A (n=1)  c.1229T>C (n=1) | 3 ARs | 3 Not reported  3 Yes MIS-C  AND  3 No comorbidities | 1 Thrombocytopenia  1 Anaemia | 3 Not reported | 3 Not reported | 3 Not reported | 3 Not reported | (NOS, 7)  3 outcome was not reported |
| Giardino et al. 2022 ([63](#_ENREF_63)), Italy | Retrospective cohort, multi-centre | 156 | 1 (100) | 1 White (Caucasian) | 1 DNA repair defect (Ataxia-telangiectasia) | ATM (n=1) | 1 NOPMiIrPwD | 1 AR | 1 Asymptomatic  1 No MIS-C  AND  1 No comorbidities | 1 Not reported | 1 SCIg | 0 | 0 | 0 | (NOS, 7)  1 survived |
| Gordon et al. 2022 ([64](#_ENREF_64)), United States | Prospective cohort, single centre | 60 and 108 | 0 (0) | 2 White (Caucasian) | 2 DNA repair defects (Ataxias-telangiectasias) | ATM (n=2) | 2 NOPMiIrPwD | 2 ARs | 2 Mild  2 No MIS-C  AND  1 CLD | 1 Low WBCs  2 Neutropenia  2 Lymphopenia | 2 No treatment | 0 | 0 | 0 | (NOS, 7)  2 survived |
| Karakoc Aydiner et al. 2022 ([73](#_ENREF_73)), Turkey | Prospective cohort, multi-centre | 80 | 0 (0) | 1 White (Caucasian) | 1 DNA repair defect (Ataxia-telangiectasia) | ATM (n=1) | 1 NOPMiIrPwD | 1 AR | 1 Mild  1 No MIS-C  AND  1 IBD | 1 Neutropenia  1 High CRP  1 Raised procalcitonin  1 High ferritin  1 High LDH  1 High D-dimer | 1 Antibiotics  1 IVIG | 0 | 0 | 0 | (NOS, 7)  1 survived |
| Karimi et al. 2021 ([74](#_ENREF_74)), Iran | Retrospective cohort, multi- centre | 112 and 7 | 2 (100) | 2 Persians | 2 DNA repair defects (Ataxias-telangiectasias) | ATM (n=2) | 2 NOPMiIrPwD | 2 ARs | 1 Mild  1 Moderate  1 Not reported  AND  2 No comorbidities | 2 Not possible to extract | 2 Antibiotics  2 IVIG  1 Antifungals  1 Steroids | 0 | 0 | 0 | (NOS, 6)  2 survived |
| Kołtan et al. 2022 ([81](#_ENREF_81)), Poland | Retrospective cohort, multi-centre | 2 Not possible to extract | 2 Not possible to extract | 2 Whites (Caucasians) | 2 DNA repair defects (Ataxias-telangiectasias) | ATM (n=2) | 2 NOPMiIrPwD | 2 ARs | 2 Mild  2 No MIS-C  AND  2 Not possible to extract | 2 Not reported | 2 Not possible to extract | 0 | 0 | 0 | (NOS, 7)  2 survived |
| Milito et al. 2021 ([97](#_ENREF_97)), Italy | Retrospective cohort, multi-centre | Age group: <216 (n=1) | 1 Not possible to extract | 1 Whites (Caucasians) | 1 DNA repair defect (Ataxia-telangiectasia) | ATM (n=1) | 1 NOPMiIrPwD | 1 AR | 1 Mild  1 No MIS-C  AND  1 Not possible to extract | 1 Not reported | 1 Not reported | 0 | 0 | 0 | (NOS, 7)  1 survived |
| Pieniawska-Śmiech et al. 2021 ([108](#_ENREF_108)), Poland | Retrospective cohort, single centre | 132 | 1 (100) | 1 White (Caucasian) | 1 DNA repair defect (Ataxia-telangiectasia) | ATM (n=1) | 1 NOPMiIrPwD | 1 AR | 1 Mild  1 No MIS-C  AND  1 No comorbidities | 1 Normal laboratory parameters | 1 No treatment | 0 | 0 | 0 | (NOS, 8)  1 survived |
| Sherkat et al. 2021 ([117](#_ENREF_117)), Iran | Retrospective cohort, single centre | 168 | 0 (0) | 1 Persian | 1 DNA repair defect (Ataxia-telangiectasia) | ATM (n=1) | 1 NOPMiIrPwD | 1 AR | 1 Mild  1 No MIS-C  AND  1 No comorbidities | 1 Not reported | 1 Hydroxychloroquine  1 Famotidine  1 Promethazine | 0 | 0 | 0 | (NOS, 7)  1 survived |
| Shields et al. 2022 ([118](#_ENREF_118)), United Kingdom | Retrospective cohort, multi-centre | Age group: <216 (n=4) | 1 (25) | 4 Whites (Caucasians) | 4 DNA repair defects (Ataxias-telangiectasias) | ATM (n=4) | 4 NOPMiIrPwD | 4 ARs | 4 Not reported  4 Not reported  AND  4 No comorbidities | 4 Not possible to extract | 4 Not possible to extract | 0 | 0 | 0 | (NOS, 8)  4 survived |
| Topal et al. 2022 ([124](#_ENREF_124)), Turkey | Retrospective cohort, single centre | 144 | 1 Gender was not reported | 1 White (Caucasian) | 1 DNA repair defect (Ataxia-telangiectasia) | ATM (n=1) | 1 NOPMiIrPwD | 1 AR | 1 Moderate  1 No MIS-C  AND  1 CLD  1 Previous non-Hodgkin lymphoma | 1 Not reported | 1 Oxygen supplementation | 0 | 0 | 0 | (NOS, 7)  1 survived |
| Castano-Jaramillo et al. 2021 ([40](#_ENREF_40)), Mexico | Retrospective cohort, multi-centre | 36 | 1 (100) | 1 Hispanic | 1 Immunodeficiency with congenital thrombocytopenia (Wiskott-Aldrich syndrome) | Wiskott-Aldrich syndrome protein deficiency (n=1) | 1 NOPMiIrPwD | 1 X-linked | 1 Moderate  1 No MIS-C  AND  1 *Cytomegalovirus* coinfection compromising lung, CNS and bone marrow  1 IBD  1 Post HSCT  1 CLD | 1 Not reported | 1 SCIg | 1 | 1 | 1 | (NOS, 8)  1 died (not COVID-19-related) |
| Cenciarelli et al. 2020 ([5](#_ENREF_5)), Italy | Retrospective case report, single centre | 18 | 1 (100) | 1 White (Caucasian) | 1 Immunodeficiency with congenital thrombocytopenia (Wiskott-Aldrich syndrome) | Wiskott-Aldrich syndrome protein deficiency (n=1) | 1 NOPMiIrPwD | 1 X-linked | 1 Mild  1 No MIS-C  AND  1 Vasculitis  1 Eczema | 1 Thrombocytopenia | 1 Hydroxychloroquine  1 Lopinavir/ritonavir  1 IVIG  1 Antibiotics | 0 | 0 | 0 | (Modified NOS, high)  1 survived |
| Conti et al. 2022 ([46](#_ENREF_46)), Italy | Retrospective cohort, single centre | 18 | 1 (100) | 1 White (Caucasian) | 1 Immunodeficiency with congenital thrombocytopenia (Wiskott-Aldrich syndrome) | Wiskott-Aldrich syndrome protein deficiency (n=1) | 1 NOPMiIrPwD | 1 X-linked | 1 Mild  1 No MIS-C  AND  1 No comorbidities | 1 Not reported | 1 Lopinavir/ritonavir  1 Gene therapy | 0 | 0 | 0 | (NOS, 8)  1 survived |
| Delavari et al. 2021 ([48](#_ENREF_48)), Iran | Prospective cohort, multi-centre | 5 | 1 (100) | 1 Persian | 1 Immunodeficiency with congenital thrombocytopenia (Wiskott-Aldrich syndrome) | Wiskott-Aldrich syndrome protein deficiency (n=1) | 1 NOPMiIrPwD | 1 X-linked | 1 Moderate  1 No MIS-C  AND    1 Microcytic thrombocytopenia | 1 Low Hb  1 Thrombocytopenia  1 High ESR  1 High CRP | 1 Antibiotics  1 IVIG  1 Oxygen supplementation | 0 | 0 | 0 | (NOS, 7)  1 survived |
| Giardino et al. 2022 ([63](#_ENREF_63)), Italy | Retrospective cohort, multi-centre | 16, 205 and 120 | 3 (100) | 3 Whites (Caucasians) | 3 Immunodeficiencies with congenital thrombocytopenia (Wiskott-Aldrich syndrome) | Wiskott-Aldrich syndrome protein deficiency (n=3) | 3 NOPMiIrPwD | 3 X-linked | 3 Asymptomatic  3 No MIS-C  AND  1 Autoinflammatory vasculitis  1 Scoliosis  1 Kaposi-like haemangioendothelioma | 3 Not reported | 2 Gene therapy  1 No treatment | 0 | 0 | 0 | (NOS, 7)  3 survived |
| [Goudouris](https://scholar.google.com/citations?user=nyuqBh4AAAAJ&hl=en&oi=sra) et al. 2021 ([65](#_ENREF_65)), Brazil | Retrospective cohort, multi-centre | 151 | 1 (100) | 1 Hispanic | 1 Immunodeficiency with congenital thrombocytopenia (Wiskott-Aldrich syndrome) | Wiskott-Aldrich syndrome protein deficiency (n=1) | 1 NOPMiIrPwD | 1 X-linked | 1 Asymptomatic  1 No MIS-C  AND  1 No comorbidities | 1 Not reported | 1 No treatment | 0 | 0 | 0 | (NOS, 8)  1 survived |
| Kołtan et al. 2022 ([81](#_ENREF_81)), Poland | Retrospective cohort, multi-centre | 2 Not possible to extract | 2 Not possible to extract | 2 Whites (Caucasians) | 2 Immunodeficiencies with congenital thrombocytopenia (Wiskott-Aldrich syndrome) | Wiskott-Aldrich syndrome protein deficiency (n=2) | 2 NOPMiIrPwD | 2 X-linked | 1 Asymptomatic  1 Mild  2 No MIS-C  AND  2 Not possible to extract | 2 Not reported | 2 Not possible to extract | 0 | 0 | 0 | (NOS, 7)  2 survived |
| Meyts et al. 2021 ([95](#_ENREF_95)), 10 countries | Retrospective cohort, multi-centre | Age groups: 0-24 (n=1) and 36-144 (n=1) | 2 (100) | 2 Whites (Caucasians) | 2 Immunodeficiencies with congenital thrombocytopenia (Wiskott-Aldrich syndrome) | Wiskott-Aldrich syndrome protein deficiency (n=2) | 2 NOPMiIrPwD | 2 X-linked | 1 Asymptomatic  1 Mild  2 No MIS-C  AND  1 *Cytomegalovirus* coinfection  1 Encephalitis  1 Post HSCT  1 HLH  1 Sepsis | 2 Not reported | 1 Gene therapy  2 Antibiotics  1 Antivirals  2 IVIG  2 Steroids  1 Lopinavir/ritonavir  1  1 Remdesivir  1 Convalescent plasma  1 Tocilizumab  1 Pentamidine  1 Thrombopoietin agonist | 1 | 1 | 1 | (NOS, 8)  1 survived  1 still in ICU (not COVID-19-related) |
| Mohanty et al. 2021 ([99](#_ENREF_99)), India | Retrospective case report, single centre | 17 | 1 (100) | 1 Indian | 1 Immunodeficiency with congenital thrombocytopenia (Wiskott-Aldrich syndrome) | Wiskott-Aldrich syndrome protein deficiency (n=1) | C.663_664insC (n=1) | 1 X-linked | 1 Mild  1 No MIS-C  AND  1 No comorbidities | 1 Low serum IgA level | 1 No treatment | 0 | 0 | 0 | (Modified NOS, high)  1 survived |
| Topal et al. 2022 ([124](#_ENREF_124)), Turkey | Retrospective cohort, single centre | 132 | 1 Gender was not reported | 1 White (Caucasian) | 1 Immunodeficiency with congenital thrombocytopenia (Wiskott-Aldrich syndrome) | Wiskott-Aldrich syndrome protein deficiency (n=1) | 1 NOPMiIrPwD | 1 X-linked | 1 Asymptomatic  1 No MIS-C  AND  1 No comorbidities | 1 Not reported | 1 No treatment | 0 | 0 | 0 | (NOS, 7)  1 survived |
| Kołtan et al. 2022 ([81](#_ENREF_81)), Poland | Retrospective cohort, multi-centre | 6 Not possible to extract | 6 Not possible to extract | 6 Whites (Caucasians) | 6 DNA repair defects (Nijmegen breakage syndromes) | NBS1 (n=6) | 6 NOPMiIrPwD | 6 ARs | 1 Asymptomatic  3 Mild  2 Moderate  6 No MIS-C  AND  6 Not possible to extract | 6 Not reported | 6 Not possible to extract | 0 | 0 | 0 | (NOS, 7)  6 survived |
| Pieniawska-Śmiech et al. 2021 ([108](#_ENREF_108)), Poland | Retrospective cohort, single centre | 108, 42 and 78 | 2 (66.7) | 3 Whites (Caucasians) | 3 DNA repair defects (Nijmegen breakage syndromes) | NBS1 (n=3) | 3 NOPMiIrPwD | 3 ARs | 3 Mild  3 No MIS-C  AND  3 No comorbidities | 3 Normal laboratory parameters | 3 No treatment | 0 | 0 | 0 | (NOS, 8)  3 survived |
| Abolhassani et al. 2022 ([19](#_ENREF_19)), Iran | Prospective cohort, single centre | 24 and 156 | 2 (100) | 2 Persians | 2 DNA repair defects (Immunodeficiencies with centromeric instability and facial anomalies, ICF1) | DNMT3B (n=2) | Lymphocyte development/epigenetic: BCL11B (n=1)  IFN pathway genes: DDX58, TREX1 (n=1)  IL-1 activation pathway genes: IL1RL2 and NLRP1 (n=1)  NF-kB pathway genes: NLRP12 (n=1) | 2 ARs | 2 Severe  2 No MIS-C  AND  2 Not reported | 2 Low serum IgA, IgM and IgG levels | 2 Antibiotics  2 IVIG  1 Oxygen supplementation  1 TPN  1 CPR  1 ACEIs  1 Antiplatelets  1 Amiodarone | 0 | 0 | 0 | (NOS, 8)  2 survived |
| Karakoc Aydiner et al. 2022 ([73](#_ENREF_73)), Turkey | Prospective cohort, multi-centre | 31 | 1 (100) | 1 White (Caucasian) | 1 DNA repair defect (Immunodeficiency with centromeric instability and facial anomalies, ICF1) | DNMT3B (n=1) | 1 NOPMiIrPwD | 1 AR | 1 Mild  1 No MIS-C  AND  1 Bronchiectasis | 1 High CRP  1 High D-dimer | 1 Antibiotics  1 IVIG | 0 | 0 | 0 | (NOS, 7)  1 survived |
| Delavari et al. 2021 ([48](#_ENREF_48)), Iran | Prospective cohort, multi-centre | 130 and 152 | 1 (50) | 2 Persians | 2 DNA repair defects (Immunodeficiencies with centromeric instability and facial anomalies, ICF1) | DNMT3B (n=2) | 2 NOPMiIrPwD | 2 ARs | 1 Moderate  1 Severe  2 Yes MIS-C  AND  2 Not reported | 2 High ESR  2 High CRP | 2 Antibiotics 2 IVIG | 1 | 1 | 1 | (NOS, 7)  1 survived  1 died (not reported if COVID-19-related) |
| Babaei et al. 2022 ([34](#_ENREF_34)), Iran | Retrospective cohort, single centre | 18 | 1 (100) | 1 Persian | 1 DNA repair defect (ICF syndrome) | 1 NSGDwR | 1 NOPMiIrPwD | 1 AR | 1 Mild  1 No MIS-C  AND  1 No comorbidities | 1 Not reported | 1 IVIG  1 Acyclovir  1 Antibiotics  1 Steroids | 1 | 1 | 1 | (NOS, 7)  1 survived |
| Castano-Jaramillo et al. 2021 ([40](#_ENREF_40)), Mexico | Retrospective cohort, multi-centre | 9 | 1 (100) | 1 Hispanic | 1 Immunodeficiency with congenital thrombocytopenia (ARPC1B deficiency) | ARPC1B deficiency (n=1) | 1 NOPMiIrPwD | 1 AR | 1 Mild  1 No MIS-C  AND  1 *Pseudomonas aeruginosa* coinfection  1 Septic shock | 1 Not reported | 1 IVIG | 0 | 0 | 0 | (NOS, 8)  1 survived |
| Meyts et al. 2021 ([95](#_ENREF_95)), 10 countries | Retrospective cohort, multi-centre | Age group: 0-24 (n=1) | 1 (100) | 1 White (Caucasian) | 1 Immunodeficiency with congenital thrombocytopenia (ARPC1B deficiency) | ARPC1B deficiency (n=1) | 1 NOPMiIrPwD | 1 AR | 1 Mild  1 No MIS-C  AND  1 Eczema | 1 Not reported | 1 Oxygen supplementation  1 Antibiotics  1 IVIG | 0 | 0 | 0 | (NOS, 8)  1 survived |
| Castano‐Jaramillo et al. 2021 ([39](#_ENREF_39)), Mexico | Retrospective case report, single centre | 8 | 1 (100) | 1 Hispanic | 1 Immunodeficiency with congenital thrombocytopenia (ARPC1B deficiency) | ARPC1B deficiency (n=1) | p.Glu300fs (n=1) | 1 AR | 1 Severe  1 No MIS-C    AND  1 Type I IFN deficiency  1 *Pseudomonas aeruginosa* coinfection  1 Dark discoloration  1 Rectal bleeding  1 Eczema  1 Septic shock | 1 Lymphopenia  1 Leukocytosis  1 Neutrophilia  1 Eosinophilia  1 Thrombocytopenia  1 Elevated PTT | 1 IV fluids  1 Antibiotics  1 Antifungals  1 IVIG | 0 | 0 | 0 | (Modified NOS, high)  1 survived |
| David et al. 2022 ([47](#_ENREF_47)), Israel | Retrospective cohort, single centre | 60, 48 and 6 | 2 (66.7) | 3 Jews | 3 Hypoparathyroidism-retardation-dysmorphism syndromes (subcategories were not reported) | TBCE (n=3) | c.155_166del (n=3) | 3 ARs | 3 Not reported  3 Not reported  AND  1 *Klebsiella oxytoca* bacteraemia  1 *Respiratory syncytial virus* coinfection  1 *Adenovirus* coinfection  1 *Parainfluenza* coinfection  1 Human metapneumovirus coinfection  1 Hypoparathyroidism  1 Severe progressive developmental delay  1 Adrenal insufficiency  1 Severe growth retardation | 1 High total IgA and IgE levels  1 Reduced frequency of naive B cells  1 Increased frequency of CD21lowCD27- B cells  1 Reduced terminally differentiated effector memory CD8,  1 Reduced inverted CD4/CD8 ratio  1 Impaired phytohemagglutinin-induced lymphocyte proliferation | 3 Antibiotics | 2 | 2 | 2 | (NOS, 7)  1 survived  2 died (not COVID-19-related) |
| Babaei et al. 2022 ([34](#_ENREF_34)), Iran | Retrospective cohort, single centre | 32 | 0 (0) | 1 Persian | 1 Calcium channel defects (STIM1 deficiency) | STIM1 deficiency (n=1) | 1 NOPMiIrPwD | 1 AR | 1 Moderate  1 No MIS-C  AND  1 Sepsis  1 Cardiorespiratory arrest  1 AIHA  1 ITP  1 Viscosities  1 Nephrotic syndrome  1 Myopathy | 1 Not reported | 1 IVIG  1 Antibiotics | 1 | 1 | 1 | (NOS, 7)  1 died (not COVID-19-related) |
| Karimi et al. 2021 ([74](#_ENREF_74)), Iran | Retrospective cohort, multi- centre | 31 | 0 (0) | 1 Persian | 1 Calcium channel defects (STIM1 deficiency) | STIM1 deficiency (n=1) | 1 NOPMiIrPwD | 1 AR | 1 Moderate  1 Not reported  AND  1 AIHA  1 Vasculitis  1 Nephrotic syndrome  1 Myopathy | 1 Thrombocytopenia | 1 IVIG  1 Steroids  1 Antibiotics | 0 | 0 | 0 | (NOS, 6)  1 survived |
| Alkan et al. 2021 ([30](#_ENREF_30)), Turkey | Retrospective case report, single centre | 78 | 1 (100) | 1 White (Caucasian) | 1 EDA-ID caused by hypomorphic mutations in encoding the nuclear factor κB essential modulator (NEMO) protein | IKBKG (n=1) | 1 NOPMiIrPwD | 1 X-linked | 1 Severe  1 Yes MIS-C  AND  1 No commodities | 1 Low WBCs  1 Low Hb  1 High ESR  1 High CRP  1 High interleukin-6 | 1 Antibiotics  1 Oxygen supplementation  1 Hydroxychloroquine  1 Favipiravir  1 IVIG  1 Convalescent plasma | 0 | 0 | 0 | (Modified NOS, high)  1 survived |
| Karakoc Aydiner et al. 2022 ([73](#_ENREF_73)), Turkey | Prospective cohort, multi-centre | 76 | 1 (100) | 1 White (Caucasian) | 1 EDA-ID caused by hypomorphic mutations in encoding the nuclear factor κB essential modulator (NEMO) protein | IKBKG (n=1) | 1 NOPMiIrPwD | 1 X-linked | 1 Mild  1 No MIS-C  AND  1 IBD | 1 High ESR  1 High CRP  1 High LDH  1 High D-dimer  1 High fibrinogen  1 High interleukin-6 | 1 Antibiotics | 0 | 0 | 0 | (NOS, 7)  1 survived |
| Giardino et al. 2022 ([63](#_ENREF_63)), Italy | Retrospective cohort, multi-centre | 144 | 0 (0) | 1 White (Caucasian) | 1 Other CIDs with syndromic features (Kabuki syndrome) | KDM6A deficiency (n=1) | 1 NOPMiIrPwD | 1 X-linked | 1 Mild  1 No MIS-C  AND  1 Cerebral lymphoproliferation | 1 Not reported | 1 Mycophenolate mofetil | 0 | 0 | 0 | (NOS, 7)  1 survived |
| Pieniawska-Śmiech et al. 2021 ([108](#_ENREF_108)), Poland | Retrospective cohort, single centre | 48 | 0 (0) | 1 White (Caucasian) | 1 Other CIDs with syndromic features (Kabuki syndrome) | KDM6A deficiency (n=1) | 1 NOPMiIrPwD | 1 X-linked | 1 Mild  1 No MIS-C  AND  1 No comorbidities | 1 Low serum IgG levels | 1 No treatment | 0 | 0 | 0 | (NOS, 8)  1 survived |
| Khalid et al. 2021 ([76](#_ENREF_76)), United States | Retrospective case report, single centre | 96 | 1 (100) | 1 White (Caucasian) | 1 Hyper IgE Syndromes (PGM3 deficiency) | PGM3 deficiency (n=1) | 1 NOPMiIrPwD | 1 AR | 1 Mild  1 No MIS-C  AND  1 No comorbidities | 1 Lymphopenia  1 Neutropenia | 1 No treatment | 0 | 0 | 0 | (Modified NOS, high)  1 survived |
| Meyts et al. 2021 ([95](#_ENREF_95)), 10 countries | Retrospective cohort, multi-centre | Age groups: 36-144 (n=1) | 1 (100) | 1 White (Caucasian) | 1 Hyper IgE Syndromes (PGM3 deficiency) | PGM3 deficiency (n=1) | 1 NOPMiIrPwD | 1 AR | 1 Mild  1 No MIS-C  AND  1 Neutropenia  1 Eczema  1 Cognitive disability | 1 Not reported | 1 Lopinavir/ritonavir | 0 | 0 | 0 | (NOS, 8)  1 survived |
| Gelzo et al. 2022 ([62](#_ENREF_62)), Italy | Retrospective cohort, single centre | 120 and 108 | 2 (100) | 2 Whites (Caucasians) | 2 DNA repair defects (MCM4 deficiencies) | MCM4 (n=2) | c.2461C>T (n=1)  c.848T>C (n=1) | 2 ARs | 2 Not reported  2 Yes MIS-C  AND  2 No comorbidities | 2 Not reported | 2 Not reported | 2 Not reported | 2 Not reported | 2 Not reported | (NOS, 7)  2 outcome was not reported |
| Conti et al. 2022 ([46](#_ENREF_46)), Italy | Retrospective cohort, single centre | 72 | 1 (100) | 1 White (Caucasian) | 1 DNA repair defect (Bloom syndrome) | BLM (n=1) | 1 NOPMiIrPwD | 1 AR | 1 Mild  1 No MIS-C  AND  1 Short stature | 1 Not reported | 1 No treatment | 0 | 0 | 0 | (NOS, 8)  1 survived |
| Conti et al. 2022 ([46](#_ENREF_46)), Italy | Retrospective cohort, single centre | 96 | 1 (100) | 1 White (Caucasian) | 1 Immuno-osseous dysplasia (Schimke immuno-osseous dysplasia) | SMARCAL1 (n=1) | 1 NOPMiIrPwD | 1 AR | 1 Moderate  1 No MIS-C  AND  1 Epilepsy  1 Ischemic/haemorrhagic neurological event  1 Post HSCT  1 Neurodevelopmental delay  1 Kidney transplantation | 1 Not reported | 1 No treatment | 0 | 0 | 0 | (NOS, 8)  1 survived |
| Vagrecha et a. 2022 ([125](#_ENREF_125)), United States | Retrospective cohort, single centre | 36 | 0 (0) | 1 Hispanic | 1 Calcium channel defect (ORAI-1 deficiency) | ORAI1 deficiency (n=1) | c.763G>A (n=1) | 1 AR | 1 Not reported  1 Yes MIS-C  AND  1 No comorbidities | 1 Not possible to extract | 1 Not possible to extract | 1 Not possible to extract | 1 Not possible to extract | 1 Not possible to extract | (NOS, 7)  1 outcome was not reported |
| Abolhassani et al. 2022 ([19](#_ENREF_19)), Iran | Prospective cohort, single centre | 24 | 1 (100) | 1 Persian | 1 Thymic defect with additional congenital anomalies (TBX1 deficiency) | TBX1 (n=1) | DNA repair gene defect: MSH6, MCM4, and MCM10 (n=1) | 1 AD | 1 Severe  1 Yes MIS-C  AND  1 Not reported | 1 Low serum IgM and IgA level | 1 Antibiotics  1 IVIG  1 ACEIs  1 Antiplatelets  1 Biological agents  1 TPN | 1 | 0 | 0 | (NOS, 8)  1 survived |
| Delavari et al. 2021 ([48](#_ENREF_48)), Iran | Prospective cohort, multi-centre | 72 | 0 (0) | 1 Persian | 1 Hyper IgM syndrome (subcategory was not reported) | 1 NSGDwR | 1 NOPMiIrPwD | 1 AR | 1 Mild  1 No MIS-C  AND  1 Granulomatous inflammatory process in bone marrow aspiration | 1 Low Hb  1 Thrombocytopenia  1 High ESR  1 High CRP | 1 Hydroxychloroquine  1 Antibiotics  1 Oxygen supplementation | 0 | 0 | 0 | (NOS, 7)  1 survived |
| [Goudouris](https://scholar.google.com/citations?user=nyuqBh4AAAAJ&hl=en&oi=sra) et al. 2021 ([65](#_ENREF_65)), Brazil | Retrospective cohort, multi-centre | 184 and 198 | 2 (100) | 2 Hispanics | 2 Hyper IgM syndromes (subcategories were not reported) | 2 NSGDwR | 2 NOPMiIrPwD | 2 Unknowns | 1 Mild  1 Critical  2 No MIS-C  AND  1 Pleural edema  1 Diabetes mellitus type II  1 Arterial hypertension  1 Obesity | 2 Not reported | 2 IVIG | 1 | 1 | 1 | (NOS, 8)  1 survived  1 died (COVID-19-related) |
| Karimi et al. 2021 ([74](#_ENREF_74)), Iran | Retrospective cohort, multi- centre | 107 | 1 (100) | 1 Persian | 1 Hyper IgM syndrome (subcategory was not reported) | 1 NSGDwR | 1 NOPMiIrPwD | 1 Unknown | 1 Mild  1 Not reported  AND  1 Seizures | 1 Not possible to extract | 1 IVIG  1 Antibiotics  1 Antifungals | 0 | 0 | 0 | (NOS, 6)  1 survived |
| Marcus et al. 2021 ([92](#_ENREF_92)), Israel | Retrospective cohort, multi-centre | 204 | 1 (100) | 1 Jew | 1 Hyper IgM syndrome (subcategory was not reported) | 1 NSGDWR | 1 NOPMiIrPwD | 1 Unknown | 1 Mild  1 No MIS-C  AND  1 No comorbidities | 1 Not reported | 1 No treatment | 0 | 0 | 0 | (NOS, 7)  1 survived |
| Safarirad et al. 2021 ([112](#_ENREF_112)), Iran | Retrospective case report, single centre | 84 | 0 (0) | 1 Persian | 1 Hyper IgM syndrome (subcategory was not reported) | 1 NSGDwR | 1 NOPMiIrPwD | 1 Unknown | 1 Moderate  1 No MIS-C  AND  1 No comorbidities | 1 Anaemia  1 Thrombocytopenia  1 High IgM level  1 Low RBCs  1 Low Hb  1 Lymphopenia  1 High ESR | 1 Antibiotics 1 Chloroquine | 0 | 0 | 0 | (Modified NOS, moderate)  1 survived |
| Theophanous et al. 2021 ([123](#_ENREF_123)), United States | Retrospective case report, single centre | 72 | 1 (100) | 1 White (Caucasian) | 1 Hyper IgM syndrome (subcategory was not reported) | 1 NSGDwR | 1 NOPMiIrPwD | 1 Unknown | 1 Asymptomatic  1 No MIS-C  AND  1 Bell’s palsy  1 Unilateral facial droop  1 Inability to close the right eye  1 Right sided mouth droop with drooling  1 Prematurity  1 Chromosome 17 and 19 deletions  1 Submucosal cleft palate  1 Tachycardia  1 Atrial and ventricular septal defects  1 Hypospadias  1 Asthma  1 Obstructive sleep apnoea  1 Gastrostomy tube feeding | 1 High WBCs | 1 IVIG  1 Acyclovir  1 Lubricating eye drops | 0 | 0 | 0 | (Modified NOS, moderate)  1 survived |
| Babaei et al. 2022 ([34](#_ENREF_34)), Iran | Retrospective cohort, single centre | 204 | 0 (0) | 1 Persian | 1 Hyper IgE syndrome (subcategory was not reported) | 1 NSGDwR | 1 NOPMiIrPwD | 1 AR | 1 Severe  1 Yes MIS-C  AND  1 Pneumonitis  1 Arthritis  1 Eczema | 1 Not reported | 1 IVIG  1 Antibiotics  1 Cetirizine | 1 | 1 | 1 | (NOS, 7)  1 died (COVID-19-related) |
| Conti et al. 2022 ([46](#_ENREF_46)), Italy | Retrospective cohort, single centre | 84, 120, 132 and 132 | 2 (50) | 4 Whites (Caucasians) | 4 Hyper IgE syndromes (subcategories were not reported) | STAT3 (n=4) | 4 NOPMiIrPwD | 4 ARs | 4 Mild  4 No MIS-C  AND  1 Eczema  1 Thrombosis | 4 Not reported | 2 Dupilumab  1 SCIg  1 IVIG  1 Antibiotics  1 Casirivimab/imdevimab  1 Banlavimib/etesemivab | 0 | 0 | 0 | (NOS, 8)  4 survived |
| **IUIS IEIs category: Predominantly antibody deficiencies (Group III)** | | | | | | | | | | | | | | | |
| Gelzo et al. 2022 ([62](#_ENREF_62)), Italy | Retrospective cohort, single centre | 168 | 1 (100) | 1 White (Caucasian) | 1 CVID (TACI deficiency) | TNFRSF13B (n=1) | c.260T>A, p (n=1) | 1 AD | 1 Not reported  1 Yes MIS-C  AND  1 No comorbidities | 1 Not reported | 1 Not reported | 1 Not reported | 1 Not reported | 1 Not reported | (NOS, 7)  1 outcome was not reported |
| Schmidt et al. 2021 ([114](#_ENREF_114)), Germany | Retrospective case report, single centre | 42 | 0 (0) | 1 White (Caucasian) | 1 CVID (TACI deficiency) | TNFRSF13B deficiency (n=1) | rs34557412 (n=1) | 1 AD | 1 Severe  1 Yes MIS-C  AND  1 Arthritis  1 Anaemia  1 Recurrent episodes of exanthema and systemic inflammation  1 DIC  1 Acute cardiac insufficiency  1 Acute heart failure  1 Hypotension  1 Heart failure  1 Venous infarction  1 Thrombosis  1 Intracranial haemorrhage  1 Aspiration pneumonia  1 DIC  1 Seizures  1 Multi-organ failure | 1 High WBCs  1 Increased lactate  1 Metabolic acidosis  1 Elevated creatinine  1 Raised liver enzymes | 1 Steroids  1 Methotrexate  1 Inhaled nitric oxide  1 Vasopressors  1 IV inotropes  1 Sodium bicarbonate  1 ECMO | 1 | 1 | 1 | (Modified NOS, high)  1 died (COVID-19-related) |
| Vagrecha et a. 2022 ([125](#_ENREF_125)), United States | Retrospective cohort, single centre | 120 | 1 (100) | 1 Hispanic | 1 CVID (TACI deficiency) | TNFRSF13B (n=1) | c.178C>T (n=1) | 1 AD | 1 Not reported  1 Yes MIS-C  AND  1 No comorbidities | 1 Not possible to extract | 1 Not possible to extract | 1 Not possible to extract | 1 Not possible to extract | 1 Not possible to extract | (NOS, 7)  1 outcome was not reported |
| Karakoc Aydiner et al. 2022 ([73](#_ENREF_73)), Turkey | Prospective cohort, multi-centre | 84 | 0 (0) | 1 White (Caucasian) | 1 CVID (TACI deficiency) | TACI deficiency (n=1) | 1 NOPMiIrPwD | 1 AR | 1 Mild  1 No MIS-C  AND  1 Bronchiectasis | 1 Lymphopenia | 1 IVIG  1 Antibiotics | 0 | 0 | 0 | (NOS, 7)  1 survived |
| Abolhassani et al. 2022 ([19](#_ENREF_19)), Iran | Prospective cohort, single centre | 120 | 0 (0) | 1 Persian | 1 CVID (NFKB1 deficiency) | NFKB1 (n=1) | Lymphocyte development/epigenetic: SAMD9 (n=1)  IFN pathway genes: IFNA8 (n=1) | 1 AD | 1 Critical  1 No MIS-C  AND  1 Not reported | 1 Not reported | 1 Antibiotics  1 IVIG  1 ACEIs  1 TPN  1 CPR  1 Convalescent plasma  1 Biological agents | 1 | 1 | 1 | (NOS, 8)  1 died (not reported if COVID-19-related) |
| Abraham et al. 2021 ([27](#_ENREF_27)), United States | Retrospective case report, single centre | 204 | 1 (100) | 1 White (Caucasian) | 1 CVID (NFKB2 deficiency) | NFKB2 (n=1) | Ser866Asn (n=1) | 1 AD | 1 Severe  1 Yes MIS-C  AND  1 Psoriasis  1 Multiple nevi | 1 Primary hypogammaglobulinemia  1 Low B cells  1 Lymphopenia  1 Hyponatremia  1 High D-dimer  1 High ferritin  1 High CRP  1 Raised procalcitonin  1 Raised liver enzymes | 1 Antibiotics  1 Antifungals 1 IVIG  1 Remdesivir  1 Steroids  1 Tocilizumab  1 Oxygen supplementation  1 IV inotropes  1 Convalescent plasma | 1 | 1 | 1 | (Modified NOS, high)  1 survived |
| Bodansky et al. 2022 ([36](#_ENREF_36)), United States | Retrospective cohort, multi- centre | An adolescent (120 to 216) | 0 (0) | 1 White (Caucasian) | 1 CVID (NFKB2 deficiency) | NFKB2 deficiency (n=1) | p.Thr684Pro (n=1) | 1 AD | 1 Severe  1 Yes MIS-C  AND  1 Type I IFN deficiency  1 Left ventricular dysfunction | 1 Lymphopenia  1 Low serum IgG and IgA levels  1 Panhypogammaglobulinemia  1 Low CD3 –CD56+  1 Low unswitched memory, % CD19+ | 1 IV inotropes | 1 | 1 | 1 | (NOS, 7)  1 survived |
| Meyts et al. 2021 ([95](#_ENREF_95)), 10 countries | Retrospective cohort, multi-centre | Age groups: 156-216 (n=1) | 1 (100) | 1 White (Caucasian) | 1 CVID (NFKB2 deficiency) | NFKB2 deficiency (n=1) | 2 NOPMiIrPwD | 1 AD | 1 Moderate  1 No MIS-C  AND  1 Psoriasis  1 Sepsis  1 HLH | 1 Not reported | 1 Antibiotics  1 Steroids  1 Tocilizumab  1 Remdesivir  1 Convalescent plasma | 1 | 0 | 0 | (NOS, 8)  1 survived |
| Szafron et al. 2020 ([122](#_ENREF_122)), United States | Retrospective case report, single centre | 168 | 0 (0) | 1 White (Caucasian) | 1 CVID (NFKB2 deficiency) | NFKB2 deficiency (n=1) | p.R853x (n=1) | 1 AD | 1 Moderate  1 No MIS-C  AND  1 *Cytomegalovirus* coinfection  1 Arthritis  1 Eczema  1 Hepatitis | 1 Not reported | 1 Oxygen supplementation  1 Steroids  1 Convalescent plasma  1 Anakinra  1 Remdesivir  1 Ganciclovir 1 Foscarnet | 0 | 0 | 1 | (Modified NOS, low)  1 survived |
| Abolhassani et al. 2022 ([19](#_ENREF_19)), Iran | Prospective cohort, single centre | 192 | 1 (100) | 1 Persian | 1 CVID (PIK3CD deficiency) | PIK3CD (n=1) | Lymphocyte development/epigenetic: NOTCH1 (n=1)  IL-1 activation pathway genes: MAP3K6 (n=1)  NF-kB pathway genes: CARD14 (n=1) | 1 AD | 1 Severe  1 No MIS-C  AND  1 Not reported | 1 Low serum IgA and IgG levels | 1 Antibiotics 1 IVIG  1 Steroids  1 Antiplatelets  1 TPN | 1 | 0 | 0 | (NOS, 8)  1 survived |
| [Goudouris](https://scholar.google.com/citations?user=nyuqBh4AAAAJ&hl=en&oi=sra) et al. 2021 ([65](#_ENREF_65)), Brazil | Retrospective cohort, multi-centre | 157 | 1 (100) | 1 Hispanic | 1 CVID (PIK3CD deficiency) | PIK3CD (n=1) | 1 NOPMiIrPwD | 1 AD | 1 Asymptomatic  1 No MIS-C  AND  1 Diabetes mellitus type II | 1 Not reported | 1 No treatment | 0 | 0 | 0 | (NOS, 8)  1 survived |
| Milito et al. 2021 ([97](#_ENREF_97)), Italy | Retrospective cohort, multi-centre | Age group: <216 (n=1) | 1 Not possible to extract | 1 Whites (Caucasians) | 1 CVID (PIK3CD deficiency) | PIK3CD (n=1) | 1 NOPMiIrPwD | 1 AD | 1 Asymptomatic  1 No MIS-C  AND  1 Not possible to extract | 1 Not reported | 1 Not reported | 0 | 0 | 0 | (NOS, 7)  1 survived |
| Abolhassani et al. 2022 ([19](#_ENREF_19)), Iran | Prospective cohort, single centre | 120 | 1 (100) | 1 Persian | 1 CVID (SH3KBP1 deficiency) | SH3KBP1 (n=1) | Lymphocyte development/epigenetic: BLK (n=1)  IL-1 activation pathway genes: PSTPIP1 (n=1)  NF-kB pathway genes: NOD2 (n=1) | 1 X-linked | 1 Severe  1 No MIS-C  AND  1 Not reported | 1 Not reported | 1 Antibiotics  1 IVIG  1 Steroids  1 Antiplatelets  1 ACEIs  1 Amiodarone  1 TPN  1 CPR | 1 | 1 | 1 | (NOS, 8)  1 died (not reported if COVID-19-related) |
| Ahanchian et al. 2021 ([28](#_ENREF_28)), Iran | Retrospective case report, single centre | 96 | 1 (100) | 1 Persian | 1 CVID (subcategory was not reported) | 1 NSGDwR | 1 NOPMiIrPwD | 1 AR | 1 Mild  1 No MIS-C  AND  1 No comorbidities | 1 Low serum IgM level  1 Low serum IgA level | 1 IVIG  1 Aminoglycoside  1 Bronchodilators  1 Antibiotics  1 Hydroxychloroquine  1 Oxygen supplementation | 0 | 0 | 0 | (Modified NOS, high)  1 survived |
| Babaei et al. 2022 ([34](#_ENREF_34)), Iran | Retrospective cohort, single centre | 72 | 0 (0) | 1 Persian | 1 CVID (subcategory was not reported) | 1 NSGDwR | 1 NOPMiIrPwD | 1 AR | 1 Mild  1 No MIS-C  AND  1 No comorbidities | 1 Anaemia  2 Not reported | 1 IVIG | 0 | 0 | 0 | (NOS, 7)  1 survived |
| Castano-Jaramillo et al. 2021 ([40](#_ENREF_40)), Mexico | Retrospective cohort, multi-centre | 180 and 108 | 2 (100) | 2 Hispanics | 2 CVIDs (subcategories were not reported) | 2 NSGDwR | 2 NOPMiIrPwD | 2 Unknowns | 2 Moderate  2 No MIS-C  AND  1 AIHA  1 Epilepsy  1 Bronchiectasis | 1 Thrombocytopenia  1 Neutropenia | 2 IVIG  1 Steroids  1 Ivermectin  1 Cyclosporin  1 Tocilizumab | 1 | 0 | 1 | (NOS, 8)  2 survived |
| Conti et al. 2022 ([46](#_ENREF_46)), Italy | Retrospective cohort, single centre | 24, 120, 168,  180,  204  and 204 | 4 (66.7) | 6 Whites (Caucasians) | 6 CVIDs (subcategories were not reported) | SALL4 (n=1)  5 NSGDwR | 6 NOPMiIrPwD | 6 ARs | 5 Mild  1 Moderate  6 No MIS-C  AND  1 Thrombophilia  1 Evan’s syndrome  1 CLD  1 Osteochondrosis | 6 Not reported | 2 Sotrovimab  1 Casirivimab/imdevimab | 0 | 0 | 0 | (NOS, 8)  6 survived |
| Deyà-Martínez et al. 2021 ([50](#_ENREF_50)), Spain | Prospective cohort, single centre | 168 and 204 | 0 (0) | 2 Whites (Caucasians) | 2 CVIDs (subcategories were not reported) | 2 NSGDwR | 2 NOPMiIrPwD | 2 ARs | 2 Asymptomatic  2 No MIS-C  AND  2 No comorbidities | 2 Not reported | 2 No treatment | 0 | 0 | 0 | (NOS, 7)  2 survived |
| Giardino et al. 2022 ([63](#_ENREF_63)), Italy | Retrospective cohort, multi-centre | 188, 171 and 156 | 2 (66.7) | 3 Whites (Caucasians) | 3 CVIDs (subcategories were not reported) | 3 NSGDwR | 3 NOPMiIrPwD | 3 ARs | 2 Mild  1 Asymptomatic  3 No MIS-C  AND  1 CHD  1 Tricuspid atresia  1 Hypoplastic right ventricle  1 Interventricular defect  1 Pulmonary artery stenosis  1 Congestive hepatopathy (post-Fontan operation) | 3 Not reported | 3 IVIG | 0 | 0 | 0 | (NOS, 7)  3 survived |
| Gordon et al. 2022 ([64](#_ENREF_64)), United States | Prospective cohort, single centre | 180 | 0 (0) | 1 White (Caucasian) | 1 CVID (subcategory was not reported) | 1 NSGDwR | 1 NOPMiIrPwD | 1 Unknown | 1 Moderate  1 No MIS-C  AND  1 CLD  1 Chromosome 18q deletion | 1 Neutrophilia  1 High WBCs  1 Lymphopenia  1 High CRP | 1 Remdesivir  1 Steroids  1 Oxygen supplementation | 1 | 0 | 0 | (NOS, 7)  1 survived |
| [Goudouris](https://scholar.google.com/citations?user=nyuqBh4AAAAJ&hl=en&oi=sra) et al. 2021 ([65](#_ENREF_65)), Brazil | Retrospective cohort, multi-centre | 99, 112 and 139 | 3 (100) | 3 Hispanics | 3 CVIDs (subcategories were not reported) | 3 NSGDwR | 3 NOPMiIrPwD | 3 Unknowns | 2 Asymptomatic  1 Mild  3 No MIS-C  AND  1 Autoimmune thrombocytopenia | 3 Not reported | 3 IVIG | 0 | 0 | 0 | (NOS, 8)  3 survived |
| Greenmyer et al. 2022 ([66](#_ENREF_66)), United States | Retrospective cohort, single centre | 216 | 1 (100) | 1 White (Caucasian) | 1 CVID (subcategory was not reported) | 1 NSGDwR | 1 NOPMiIrPwD | 1 Unknown | 1 Mild  1 No MIS-C  AND  1 Chromosome 18q deletion | 1 Low CD3 and CD4 counts  1 Low serum IgA, IgG and IgM levels | 1 No treatment | 0 | 0 | 0 | (NOS, 7)  1 survived |
| Karimi et al. 2021 ([74](#_ENREF_74)), Iran | Retrospective cohort, multi- centre | 179, 81 and 139 | 1 (33.3) | 3 Persians | 3 CVIDs (subcategories were not reported) | 3 NSGDwR | 3 NOPMiIrPwD | 3 Unknowns | 2 Mild  1 Moderate  3 Not reported  AND  1 Autoimmunity  1 Bronchiectasis  1 CLD | 3 Not possible to extract | 3 Antibiotics  2 Antifungals  2 IVIG | 0 | 0 | 0 | (NOS, 6)  3 survived |
| Kinoshita et al. 2021 ([80](#_ENREF_80)), United States | Retrospective cohort, single centre | 132 | 1 (100) | 1 White (Caucasian) | 1 CVID (subcategory was not reported) | 1 NSGDwR | 1 NOPMiIrPwD | 1 Unknown | 1 Mild  1 No MIS-C  AND  1 Celiac disease | 1 Low B cells  1 Low memory B cells | 1 IVIG | 0 | 0 | 0 | (NOS, 7)  1 survived |
| Meyts et al. 2021 ([95](#_ENREF_95)), 10 countries | Retrospective cohort, multi-centre | Age groups: 156-216 (n=1) | 1 (100) | 1 Hispanic | 1 CVID (subcategory was not reported) | 1 NSGDwR | 2 NOPMiIrPwD | 1 Unknown | 1 Asymptomatic  1 No MIS-C  AND  1 CLD | 1 Not reported | 1 No treatment | 0 | 0 | 0 | (NOS, 8)  1 survived |
| Milito et al. 2021 ([97](#_ENREF_97)), Italy | Retrospective cohort, multi-centre | Age group: <216 (n=7) | 7 Not possible to extract | 7 Whites (Caucasians) | 7 CVIDs (subcategories were not reported) | 7 NSGDwR | 7 NOPMiIrPwD | 7 Unknowns | 5 Mild  2 Asymptomatic  7 No MIS-C  AND  7 Not possible to extract | 7 Not reported | 7 Not reported | 0 | 0 | 0 | (NOS, 7)  7 survived |
| Topal et al. 2022 ([124](#_ENREF_124)), Turkey | Retrospective cohort, single centre | 180, 216, 84, 84, 168, 204, 108, and 168 | 8 Genders were not reported | 8 Whites (Caucasians) | 8 CVIDs (subcategories were not reported) | 8 NSGDwR | 8 NOPMiIrPwD | 8 Unknowns | 6 Mild  1 Asymptomatic  1 Moderate  8 No MIS-C  AND  1 IBD  1 Chronic anaemia  1 Macrothrombocytopenia  1 Hypothyroidism  2 Short stature  2 Microcephaly  1 Pituitary microadenoma  1 Mental retardation  1 Fanconi aplastic anaemia  1 ITP  1 Asthma  1 Previous adenotonsillectomy  1 Obesity  1 Hypertension  1 Factor deficiency | 8 Not reported | 7 No treatment  1 Oxygen supplementation | 0 | 0 | 0 | (NOS, 7)  8 survived |
| Abolhassani et al. 2022 ([19](#_ENREF_19)), Iran | Prospective cohort, single centre | 156 | 1 (100) | 1 Persian | 1 Agammaglobulinemia (X-linked agammaglobulinemia) | BTK deficiency (n=1) | DNA repair gene defect: CHD7 (n=1)  IFN pathway genes: STAT6, IFNLR1 and IL28R (n=1)  NF-kB pathway genes: NOD2 (n=1) | 1 X-linked | 1 Severe  1 No MIS-C  AND  1 Not reported | 1 Low serum IgA, IgM and IgG levels | 1 IVIG  1 Antibiotics  1 Biological agents  1 Convalescent plasma  1 TPN  1 Oxygen supplementation | 0 | 0 | 1 | (NOS, 8)  1 survived |
| Babaei et al. 2022 ([34](#_ENREF_34)), Iran | Retrospective cohort, single centre | 22 and 27 | 2 (100) | 2 Persians | 2 Agammaglobulinemias (X-linked agammaglobulinemias) | BTK deficiency (n=2) | 2 NOPMiIrPwD | 2 X-linked | 2 Mild  2 No MIS-C  AND  1 Poliomyelitis  1 Developmental dysplasia of the hip | 2 Not reported | 2 IVIG  1 Acyclovir  2 Antibiotics  1 Steroids | 0 | 0 | 0 | (NOS, 7)  2 survived |
| Castano-Jaramillo et al. 2021 ([40](#_ENREF_40)), Mexico | Retrospective cohort, multi-centre | 156, 156 and 204 | 3 (100) | 3 Hispanics | 3 Agammaglobulinemias (X-linked agammaglobulinemias) | BTK deficiency (n=3) | 3 NOPMiIrPwD | 3 X-linked | 3 Mild  3 No MIS-C  AND  1 Secondary hemophagocytic syndrome  1 Pulmonary haemorrhage | 3 Not reported | 1 IVIG  1 Steroids  1 Cyclosporine  1 Etoposide | 1 | 1 | 1 | (NOS, 8)  2 survived  1 died (not COVID-19-related) |
| Chappell et al. 2022 ([42](#_ENREF_42)), United Kingdom | Prospective cohort, multi-centre | 204 | 1 (100) | 1 White (Caucasian) | 1 Agammaglobulinemia (X-linked agammaglobulinemia) | 1 NSGDwR | 1 NOPMiIrPwD | 1 X-linked | 1 Mild  1 No MIS-C  AND  1 No comorbidities | 1 Not reported | 1 IVIG  1 Antibiotics  1 Remdesivir | 0 | 0 | 0 | (NOS, 7)  1 survived |
| Conti et al. 2022 ([46](#_ENREF_46)), Italy | Retrospective cohort, single centre | 156 | 1 (100) | 1 White (Caucasian) | 1 Agammaglobulinemia (X-linked agammaglobulinemia) | BTK deficiency (n=1) | 1 NOPMiIrPwD | 1 X-linked | 1 Moderate  1 No MIS-C  AND  1 Cardiopathy | 1 Not reported | 1 SCIg  1 Sotrovimab | 0 | 0 | 0 | (NOS, 8)  1 survived |
| Devassikutty et al. 2021 ([49](#_ENREF_49)), India | Retrospective case report, single centre | 216 | 1 (100) | 1 Indian | 1 Agammaglobulinemia (X-linked agammaglobulinemia) | BTK deficiency (n=1) | rs128621194 (n=1) | 1 X-linked | 1 Mild  1 No MIS-C  AND  1 Arthritis  1 Decortication for empyema thoracis  1 Short stature  1 Delayed puberty | 1 Low serum IgA level  1 High CD3 cells | 1 Antibiotics  1 IVIG | 0 | 0 | 0 | (NOS, 6)  1 survived |
| Deyà-Martínez et al. 2021 ([50](#_ENREF_50)), Spain | Prospective cohort, single centre | 96 | 1 (100) | 1 White (Caucasian) | 1 Agammaglobulinemia (X-linked agammaglobulinemia) | BTK deficiency (n=1) | 1 NOPMiIrPwD | 1 X-linked | 1 Asymptomatic  1 No MIS-C  AND  1 No comorbidities | 1 Not reported | 1 No treatment | 0 | 0 | 0 | (NOS, 7)  1 survived |
| Esenboga et al. 2021 ([52](#_ENREF_52)), Turkey | Retrospective cohort, single centre | 216, 45 and 54 | 1 (33.3) | 3 Whites (Caucasians) | 3 Agammaglobulinemias (X-linked agammaglobulinemias) | BTK deficiency (n=3) | 3 NOPMiIrPwD | 3 X-linked | 2 Mild  1 Moderate  3 No MIS-C  AND  1 AIHA  1 Juvenile idiopathic arthritis  1 Hepatitis | 3 Not reported | 1 Favipiravir 2 Antibiotics 1 Heparin  3 IVIG  1 Methotrexate  1 Azathioprine  1 Oxygen supplementation | 0 | 0 | 0 | (NOS, 7)  3 survived |
| Giardino et al. 2022 ([63](#_ENREF_63)), Italy | Retrospective cohort, multi-centre | 82, 66 and 216 | 3 (100) | 3 Whites (Caucasians) | 3 Agammaglobulinemias (X-linked agammaglobulinemias) | BTK deficiency (n=3) | 3 NOPMiIrPwD | 3 X-linked | 2 Mild  1 Asymptomatic  3 No MIS-C  AND  3 No comorbidities | 3 Not reported | 2 SCIg  1 IVIG | 0 | 0 | 0 | (NOS, 7)  3 survived |
| [Goudouris](https://scholar.google.com/citations?user=nyuqBh4AAAAJ&hl=en&oi=sra) et al. 2021 ([65](#_ENREF_65)), Brazil | Retrospective cohort, multi-centre | 31, 64, 117, 132, and 181 | 5 (100) | 5 Hispanics | 5 Agammaglobulinemias (X-linked agammaglobulinemias) | BTK deficiency (n=3)  2 NSGDwR | 5 NOPMiIrPwD | 5 X-linked | 4 Mild  1 Critical  5 No MIS-C  AND  1 Bacterial pneumonia  1 Bronchiectasis  1 Obesity | 5 Not reported | 2 IVIG  1 SCIg | 1 | 1 | 1 | (NOS, 8)  4 survived  1 died (COVID-19-related) |
| Ho et al. 2021 ([71](#_ENREF_71)), United States | Retrospective cohort, single centre | 120 | 1 (100) | 1 White (Caucasian) | 1 Agammaglobulinemia (X-linked agammaglobulinemia) | BTK deficiency (n=1) | 1 NOPMiIrPwD | 1 X-linked | 1 Mild  1 No MIS-C  AND  1 No comorbidities | 1 High CRP  1 High fibrinogen  1 High interleukin-6 | 1 Oxygen supplementation  1 Convalescent plasma | 0 | 0 | 0 | (NOS, 7)  1 survived |
| Jin et al. 2020 ([8](#_ENREF_8)), United States | Retrospective case report, single centre | 120 | 1 (100) | 1 White (Caucasian) | 1 Agammaglobulinemia (X-linked agammaglobulinemia) | BTK deficiency (n=1) | 1 NOPMiIrPwD | 1 X-linked | 1 Severe  1 No MIS-C  AND  1 Hereditary spherocytosis | 1 Leukopenia  1 Thrombocytopenia  1 Lymphocytosis  1 Anaemia  1 High ESR  1 High CRP | 1 Antibiotics  1 Platelet transfusions  1 Oxygen supplementation  1 Bronchodilators  1 IVIG  1 Heparin  1 Remdesivir 1 Convalescent plasma | 0 | 0 | 0 | (Modified NOS, high)  1 survived |
| Karakoc Aydiner et al. 2022 ([73](#_ENREF_73)), Turkey | Prospective cohort, multi-centre | 93 | 1 (100) | 1 White (Caucasian) | 1 Agammaglobulinemia (X-linked agammaglobulinemia) | BTK deficiency (n=1) | 1 NOPMiIrPwD | 1 X-linked | 1 Asymptomatic  1 No MIS-C  AND  1 No comorbidities | 1 High CRP | 1 IVIG  1 Antibiotics | 0 | 0 | 0 | (NOS, 7)  1 survived |
| Karimi et al. 2021 ([74](#_ENREF_74)), Iran | Retrospective cohort, multi- centre | 163 | 1 (100) | 1 Persian | 1 Agammaglobulinemia (X-linked agammaglobulinemia) | BTK deficiency (n=1) | 1 NOPMiIrPwD | 1 X-linked | 1 Moderate  1 Not reported  AND  1 No comorbidities | 1 Not possible to extract | 1 IVIG  1 Antibiotics | 0 | 0 | 0 | (NOS, 6)  1 survived |
| Lang-Meli et al. 2022 ([85](#_ENREF_85)), Germany | Retrospective case-series, multi-centre | 132 and 156 | 2 (100) | 2 Whites (Caucasians) | 2 Agammaglobulinemias (X-linked agammaglobulinemias) | BTK deficiency (n=2) | 2 NOPMiIrPwD | 2 X-linked | 1 Mild  1 Moderate  2 No MIS-C  AND  2 No comorbidities | 2 Low serum IgM and IgA levels | 2 IVIG  2 Antibiotics  2 Remdesivir  2 Convalescent plasma | 0 | 0 | 0 | (Modified NOS, high)  2 survived |
| Marcus et al. 2021 ([92](#_ENREF_92)), Israel | Retrospective cohort, multi-centre | 72 and 60 | 2 (100) | 2 Jews | 2 Agammaglobulinemias (X-linked agammaglobulinemias) | BTK deficiency (n=2) | 2 NOPMiIrPwD | 2 X-linked | 1 Asymptomatic  1 Mild  2 No MIS-C  AND  2 Agammaglobulinemia | 2 Not reported | 2 IVIG | 0 | 0 | 0 | (NOS, 7)  2 survived |
| Marcus et al. 2022 ([91](#_ENREF_91)), Israel | Retrospective case report, multi-centre | 210, 156 and 114 | 3 (100) | 3 Jews | 3 Agammaglobulinemias (X-linked agammaglobulinemias) | BTK deficiency (n=3) | 3 NOPMiIrPwD | 3 X-linked | 1 Mild  1 Moderate  1 Severe  3 Not reported  AND  1 Diabetes mellitus type I | 3 Not reported | 1 IVIG  2 Antibiotics 1 Insulin  2 IV fluids  1 Heparin  1 Convalescent plasma | 0 | 0 | 0 | (Modified NOS, high)  3 survived |
| Meyts et al. 2021 ([95](#_ENREF_95)), 10 countries | Retrospective cohort, multi-centre | Age groups: 36-144 (n=2) | 2 (100) | 2 Whites (Caucasians) | 2 Agammaglobulinemias (X-linked agammaglobulinemias) | BTK deficiency (n=2) | 2 NOPMiIrPwD | 2 X-linked | 1 Asymptomatic  1 Moderate  2 No MIS-C  AND  1 Hereditary spherocytosis | 2 Not reported | 2 Antibiotics  1 Remdesivir  1 Heparin  1 Convalescent plasma  1 Steroids  1 IVIG  1 Oxygen supplementation | 0 | 0 | 0 | (NOS, 8)  2 survived |
| Milito et al. 2021 ([97](#_ENREF_97)), Italy | Retrospective cohort, multi-centre | Age group: <216 (n=5) | 5 Not possible to extract | 5 Whites (Caucasians) | 5 Agammaglobulinemias (X-linked agammaglobulinemias) | BTK deficiency (n=5) | 5 NOPMiIrPwD | 5 X-linked | 2 Asymptomatic  3 Mild  5 No MIS-C  AND  5 Not possible to extract | 5 Not reported | 5 Not reported | 0 | 0 | 0 | (NOS, 7)  5 survived |
| Pereira et al. 2021 ([105](#_ENREF_105)), United Kingdom | Retrospective case report, single centre | 204 | 1 (100) | 1 White (Caucasian) | 1 Agammaglobulinemia (X-linked agammaglobulinemia) | BTK deficiency (n=1) | 1 NOPMiIrPwD | 1 X-linked | 1 Severe  1 No MIS-C  AND  1 No comorbidities | 1 High CRP  1 High D-dimer  1 High ESR  1 Low Hb | 1 Remdesivir  1 Antibiotics  1 IVIG | 0 | 0 | 0 | (Modified NOS, high)  1 survived |
| Sherkat et al. 2021 ([117](#_ENREF_117)), Iran | Retrospective cohort, single centre | 192 | 1 (100) | 1 Persian | 1 Agammaglobulinemia (X-linked agammaglobulinemia) | BTK deficiency (n=1) | 1 NOPMiIrPwD | 1 X-linked | 1 Moderate  1 No MIS-C  AND  1 Pulmonary aspergillosis  1 Bacterial pneumonia | 1 Not reported | 1 Oxygen supplementation  1 Hydroxychloroquine  1 Antibiotics  1 Antifungals  1 Famotidine  1 Promethazine  1 Steroids  1 Remdesivir | 0 | 0 | 1 | (NOS, 7)  1 survived |
| Speletas et al. 2022 ([120](#_ENREF_120)), Greece | Retrospective case report, single centre | 180 and 216 | 2 (100) | 2 Whites (Caucasians) | 2 Agammaglobulinemias (X-linked agammaglobulinemias) | BTK deficiency (n=2) | c.1700C>T, p.A567V (n=1)  c.519_insCTGCATTGAGA (n=1) | 2 X-linked | 1 Mild  1 No MIS-C  AND  2 No comorbidities | 2 Normal laboratory parameters | 2 No treatment | 0 | 0 | 0 | (Modified NOS, high)  2 survived |
| Westreich 2021 ([129](#_ENREF_129)), United States | Retrospective case report, single centre | 144 | 1 (100) | 1 White (Caucasian) | 1 Agammaglobulinemia (X-linked agammaglobulinemia) | BTK deficiency (n=1) | A523V (n=1) | 1 X-linked | 1 Moderate  1 No MIS-C  AND  1 No comorbidities | 1 Normal laboratory parameters | 1 Antibiotics  1 IVIG | 0 | 0 | 0 | (Modified NOS, high)  1 survived |
| Yadav et al. 2022 ([130](#_ENREF_130)), India | Retrospective case report, single centre | 120 | 1 (100) | 1 Indian | 1 Agammaglobulinemia (X-linked agammaglobulinemia) | 1 NSGDwR | 1 NOPMiIrPwD | 1 X-linked | 1 Mild  1 No MIS-C  AND  1 No comorbidities | 1 Normal laboratory parameters | 1 No treatment | 0 | 0 | 0 | (Modified NOS, high)  1 survived |
| Kuster et al. 2022 ([83](#_ENREF_83)), United States | Retrospective case report, single centre | 216 | 0 (0) | 1 White (Caucasian) | 1 Agammaglobulinemia (E47 transcription factor deficiency) | TCF3 deficiency (n=1) | 1 NOPMiIrPwD | 1 AD | 1 Mild  1 No MIS-C  AND  1 No comorbidities | 1 Not reported | 1 No treatment | 0 | 0 | 0 | (Modified NOS, high)  1 survived |
| Clemente et al. 2022 ([45](#_ENREF_45)), United Kingdom | Retrospective case report, single centre | 48 | 1 (100) | 1 Asian | 1 Agammaglobulinemia (PIK3R1 deficiency) | PIK3R1 deficiency (n=1) | 1 NOPMiIrPwD | 1 AD | 1 Severe  1 Yes MIS-C  AND  1 HLH  1 Acute renal impairment  1 Myopathy | 1 Increased T-cells  1 Raised serum soluble Fas ligand  1 Low I T cells  1 Low CD4+ and CD8+ T-cell receptor excision circles levels  1 Raised IgG and IgM levels  1 Lymphopenia  1 Thrombocytopenia  1 High ESR  1 High ferritin  1 High LDH  1 Elevated creatinine | 1 Remdesivir  1 Nitazoxanide  1 Steroids  1 Tocilizumab  1 Antibiotics  1 Antifungals  1 IVIG  1 Sirolimus  1 Rituximab  1 IV fluids  1 Oxygen supplementation  1 IV inotropes  1 Prone positioning  1 Neuromuscular blockade | 1 | 1 | 1 | (Modified NOS, high)  1 survived |
| Giardino et al. 2022 ([63](#_ENREF_63)), Italy | Retrospective cohort, multi-centre | 180 | 1 (100) | 1 White (Caucasian) | 1 Agammaglobulinemia (PIK3R1 deficiency) | PIK3R1 deficiency (n=1) | 1 NOPMiIrPwD | 1 AD | 1 Not reported  1 Not reported  AND  1 Crohn disease | 1 Not reported | 1 SCIg  1 Antibiotics | 0 | 0 | 0 | (NOS, 7)  1 survived |
| Esenboga et al. 2021 ([52](#_ENREF_52)), Turkey | Retrospective cohort, single centre | 72 | 1 (100) | 1 White (Caucasian) | 1 Hypogammaglobulinemia (subcategory was not reported) | 1 NSGDwR | 1 NOPMiIrPwD | 1 AD | 1 Mild  1 No MIS-C  AND  1 No comorbidities | 1 Not reported | 1 Antibiotics | 0 | 0 | 0 | (NOS, 7)  1 survived |
| Exelmans et al. 2021 ([55](#_ENREF_55)), Belgium | Retrospective case report, single centre | 120 | 1 (100) | 1 White (Caucasian) | 1 Hypogammaglobulinemia (subcategory was not reported) | 1 NSGDwR | 1 NOPMiIrPwD | 1 Unknown | 1 Mild  1 No MIS-C  AND  1 *Mycoplasma pneumoniae* coinfection  1 Down syndrome  1 Psychomotor retardation  1 Hypotonia  1 Ventricular dysfunction  1 Pulmonary hypertension  1 Pericardial effusion  1 Pulmonary embolism  1 Heart failure | 1 Lymphopenia  1 Hypercapnia  1 Hypoxemia  1 Raised liver enzymes  1 High D-dimer  1 High WBCs  1 Neutrophilia  1 Lymphocytosis  1 Thrombocytopenia  1 High CRP  1 Elevated PT  1 High Troponine I  1 High brain natriuretic peptide  1 High LDH | 1 Steroids  1 Antibiotics  1 Bronchodilators  1 Diuretics  1 Heparin  1 Oxygen supplementation | 1 | 0 | 0 | (Modified NOS, moderate)  1 survived |
| [Goudouris](https://scholar.google.com/citations?user=nyuqBh4AAAAJ&hl=en&oi=sra) et al. 2021 ([65](#_ENREF_65)), Brazil | Retrospective cohort, multi-centre | 33, 126, 163 and 189 | 1 (25) | 4 Hispanics | 4 Hypogammaglobulinemia (subcategories were not reported) | 4 NSGDwR | 4 NOPMiIrPwD | 4 Unknowns | 1 Asymptomatic  3 Mild  4 No MIS-C  AND  4 No comorbidities | 4 Not reported | 3 IVIG | 0 | 0 | 0 | (NOS, 8)  4 survived |
| Kinoshita et al. 2021 ([80](#_ENREF_80)), United States | Retrospective cohort, single centre | 132 | 1 (100) | 1 White (Caucasian) | 1 Hypogammaglobulinemia (subcategory was not reported) | 1 NSGDwR | 1 NOPMiIrPwD | 1 Unknown | 1 Mild  1 No MIS-C  AND  1 Tonsillectomy | 1 Hypogammaglobulinemia  1 Low serum IgA levels  1 Elevated absolute B cell count  1 Low normal memory B cells  1 Low non-class switched memory B cells | 1 No treatment | 0 | 0 | 0 | (NOS, 7)  1 survived |
| Kołtan et al. 2022 ([81](#_ENREF_81)), Poland | Retrospective cohort, multi-centre | 25 Not possible to extract | 25 Not possible to extract | 25 Whites (Caucasians) | 25 Hypogammaglobulinemia (subcategories were not reported) | 25 NSGDwR | 25 NOPMiIrPwD | 25 Unknowns | 17 Mild  5 Asymptomatic  2 Moderate  1 Severe  24 No MIS-C  1 Yes MIS-C  AND  25 Not possible to extract | 25 Not reported | 25 Not possible to extract | 0 | 0 | 0 | (NOS, 7)  25 survived |
| Topal et al. 2022 ([124](#_ENREF_124)), Turkey | Retrospective cohort, single centre | 36 and 48 | 2 Genders were not reported | 2 Whites (Caucasians) | 2 Hypogammaglobulinemia (subcategories were not reported) | 2 NSGDwR | 2 NOPMiIrPwD | 2 Unknowns | 2 Mild  2 No MIS-C  AND  2 No comorbidities | 2 Not reported | 1 Hydroxychloroquine  1 No treatment | 0 | 0 | 0 | (NOS, 7)  2 survived |
| Giardino et al. 2022 ([63](#_ENREF_63)), Italy | Retrospective cohort, multi-centre | 105, 108, 211 and 102 | 3 (75) | 4 Whites (Caucasians) | 4 Hypogammaglobulinemia (subcategories were not reported) | 4 NSGDwR | 4 NOPMiIrPwD | 4 Unknowns | 3 Mild  1 Asymptomatic  4 No MIS-C  AND  3 No comorbidities  1 Hypercholesterolemia | 4 Not reported | 1 Antibiotics  3 No treatment | 0 | 0 | 0 | (NOS, 7)  4 survived |
| Kołtan et al. 2022 ([81](#_ENREF_81)), Poland | Retrospective cohort, multi-centre | 7 Not possible to extract | 7 Not possible to extract | 7 Whites (Caucasians) | 7 Isotype, light chain, or functional deficiencies with generally normal numbers of B cells (Isolated IgG subclass deficiencies) | 7 NSGDwR | 7 NOPMiIrPwD | 7 Unknowns | 1 Asymptomatic  3 Mild  2 Moderates  1 Severe  7 No MIS-C  AND  7 Not possible to extract | 7 Not reported | 7 Not possible to extract | 0 | 0 | 0 | (NOS, 7)  7 survived |
| Pieniawska-Śmiech et al. 2021 ([108](#_ENREF_108)), Poland | Retrospective cohort, single centre | 30, 144, 96, 48, 84, 90, 72, 108, 18, 150, 42, 18, 102, 54, 132, 114, 24, 84, 36, 54, and 186 | 15 (71.4) | 21 Whites (Caucasians) | 21 Isotype, light chain, or functional deficiencies with generally normal numbers of B cells (Isolated IgG subclass deficiencies) | 21 NSGDwR | 21 NOPMiIrPwD | 21 Unknowns | 4 Asymptomatic  17 Mild  20 No MIS-C  1 Yes MIS-C  AND  20 No comorbidities  1 Down syndrome | 21 Low serum IgG levels | 21 No treatment | 0 | 0 | 0 | (NOS, 8)  21 survived |
| Conti et al. 2022 ([46](#_ENREF_46)), Italy | Retrospective cohort, single centre | 192 | 1 (100) | 1 White (Caucasian) | 1 Isotype, light chain, or functional deficiencies with generally normal numbers of B cells (Selective IgA deficiency) | 1 NSGDwR | 1 NOPMiIrPwD | 1 Unknown | 1 Mild  1 No MIS-C  AND  1 Celiac disease  1 ITP | 1 Not reported | 1 No treatment | 0 | 0 | 0 | (NOS, 8)  1 survived |
| Delavari et al. 2021 ([48](#_ENREF_48)), Iran | Prospective cohort, multi-centre | 96 | 1 (100) | 1 Persian | 1 Isotype, light chain, or functional deficiencies with generally normal numbers of B cells (Selective IgA deficiency) | 1 NSGDwR | 1 NOPMiIrPwD | 1 Unknown | 1 Mild  1 No MIS-C  AND  1 No comorbidities | 1 Low Hb  1 Thrombocytosis  1 High ESR  1 High CRP | 1 Antibiotics  1 Hydroxychloroquine  1 IVIG | 0 | 0 | 0 | (NOS, 7)  1 survived |
| Esenboga et al. 2021 ([52](#_ENREF_52)), Turkey | Retrospective cohort, single centre | 108 | 1 (100) | 1 White (Caucasian) | 1 Isotype, light chain, or functional deficiencies with generally normal numbers of B cells (Selective IgA deficiency) | 1 NSGDwR | 1 NOPMiIrPwD | 1 Unknown | 1 Mild  1 No MIS-C  AND  1 Asthma | 1 Not reported | 1 Antibiotics  1 Steroids | 0 | 0 | 0 | (NOS, 7)  1 survived |
| [Goudouris](https://scholar.google.com/citations?user=nyuqBh4AAAAJ&hl=en&oi=sra) et al. 2021 ([65](#_ENREF_65)), Brazil | Retrospective cohort, multi-centre | 100, 150 and 181 | 1 (33.3) | 3 Hispanics | 3 Isotype, light chain, or functional deficiencies with generally normal numbers of B cells (Selective IgA deficiencies) | 3 NSGDwR | 3 NOPMiIrPwD | 3 Unknowns | 1 Asymptomatic  2 Mild  3 No MIS-C  AND  3 No comorbidities | 3 Not reported | 3 No treatment | 0 | 0 | 0 | (NOS, 8)  3 survived |
| Kołtan et al. 2022 ([81](#_ENREF_81)), Poland | Retrospective cohort, multi-centre | 3 Not possible to extract | 3 Not possible to extract | 3 Whites (Caucasians) | 3 Isotype, light chain, or functional deficiencies with generally normal numbers of B cells (Selective IgA deficiencies) | 3 NSGDwR | 3 NOPMiIrPwD | 3 Unknowns | 3 Mild  3 No MIS-C  AND  3 Not possible to extract | 3 Not reported | 3 Not possible to extract | 0 | 0 | 0 | (NOS, 7)  3 survived |
| Topal et al. 2022 ([124](#_ENREF_124)), Turkey | Retrospective cohort, single centre | 72, 120 and 96 | 3 Genders were not reported | 3 Whites (Caucasians) | 3 Isotype, light chain, or functional deficiencies with generally normal numbers of B cells (Selective IgA deficiencies) | 3 NSGDwR | 3 NOPMiIrPwD | 3 Unknowns | 2 Mild  1 Asymptomatic  3 No MIS-C  AND  1 Hypertelorism  1 Micrognathia  1 G6PD deficiency  1 Nasal polyposis | 3 Not reported | 3 No treatment | 0 | 0 | 0 | (NOS, 7)  3 survived |
| Kołtan et al. 2022 ([81](#_ENREF_81)), Poland | Retrospective cohort, multi-centre | 2 Not possible to extract | 2 Not possible to extract | 2 Whites (Caucasians) | 2 Isotype, light chain, or functional deficiencies with generally normal numbers of B cells (Selective IgM deficiencies) | 2 NSGDwR | 2 NOPMiIrPwD | 2 Unknowns | 2 Mild  2 No MIS-C  AND  2 Not possible to extract | 2 Not reported | 2 Not possible to extract | 0 | 0 | 0 | (NOS, 7)  2 survived |
| Pieniawska-Śmiech et al. 2021 ([108](#_ENREF_108)), Poland | Retrospective cohort, single centre | 54 | 0 (0) | 1 White (Caucasian) | 1 Isotype, light chain, or functional deficiency with generally normal numbers of B cells (Selective IgM deficiency) | 1 NSGDwR | 1 NOPMiIrPwD | 1 Unknown | 1 Not reported  1 No MIS-C  AND  1 No comorbidities | 1 Normal laboratory parameters | 1 No treatment | 0 | 0 | 0 | (NOS, 8)  1 survived |
| Castano-Jaramillo et al. 2021 ([40](#_ENREF_40)), Mexico | Retrospective cohort, multi-centre | 120 | 0 (0) | 1 Hispanic | 1 Specific antibody deficiency with normal immunoglobulin and B cells levels | 1 NSGDwR | 1 NOPMiIrPwD | 1 Unknown | 1 Mild  1 No MIS-C  AND  1 Lung Coccidioidomycosis | 1 Normal laboratory parameters | 1 No treatment | 0 | 0 | 0 | (NOS, 8)  1 survived |
| [Goudouris](https://scholar.google.com/citations?user=nyuqBh4AAAAJ&hl=en&oi=sra) et al. 2021 ([65](#_ENREF_65)), Brazil | Retrospective cohort, multi-centre | 151, 158 and 159 | 0 (0) | 3 Hispanics | 3 Specific antibody deficiencies with normal immunoglobulin and B cells levels | 3 NSGDwR | 3 NOPMiIrPwD | 3 Unknowns | 2 Mild  1 Severe  3 No MIS-C  AND  1 Bacterial pneumonia  1 Down syndrome  1 CHD | 3 Not reported | 3 No treatment | 1 | 1 | 1 | (NOS, 8)  3 survived |
| Pieniawska-Śmiech et al. 2021 ([108](#_ENREF_108)), Poland | Retrospective cohort, single centre | 12 | 0 (0) | 1 White (Caucasian) | 1 Isotype, light chain, or functional deficiencies with generally normal numbers of B cells (IgG subclass deficiency with IgA deficiency) | 1 NSGDwR | 1 NOPMiIrPwD | 1 Unknown | 1 Mild  1 No MIS-C  AND  1 No comorbidities | 1 Low serum IgG level | 1 No treatment | 0 | 0 | 0 | (NOS, 8)  1 survived |
| Pieniawska-Śmiech et al. 2021 ([108](#_ENREF_108)), Poland | Retrospective cohort, single centre | 84 | 0 (0) | 1 White (Caucasian) | 1 Isotype, light chain, or functional deficiency with generally normal numbers of B cells (IgG and IgM deficiency) | 1 NSGDwR | 1 NOPMiIrPwD | 1 Unknown | 1 Asymptomatic  1 No MIS-C  AND  1 No comorbidities | 1 Normal laboratory parameters | 1 No treatment | 0 | 0 | 0 | (NOS, 8)  1 survived |
| Pieniawska-Śmiech et al. 2021 ([108](#_ENREF_108)), Poland | Retrospective cohort, single centre | 198 and 12 | 1 (50) | 2 White (Caucasian) | 2 Isotype, light chain, or functional deficiencies with generally normal numbers of B cells (IgG, IgA and IgM deficiencies) | 2 NSGDwR | 2 NOPMiIrPwD | 2 Unknowns | 1 Mild  1 No MIS-C  AND  1 No comorbidities  1 Rubinstein-Taybi syndrome | 2 Low serum IgG levels | 1 Oxygen supplementation  1 Convalescent plasma | 0 | 0 | 0 | (NOS, 8)  2 survived |
| Topal et al. 2022 ([124](#_ENREF_124)), Turkey | Retrospective cohort, single centre | 72 | 1 Gender was not reported | 1 White (Caucasian) | 1 Isotype, light chain, or functional deficiency with generally normal numbers of B cells (Partial IgA deficiency) | 1 NSGDwR | 1 NOPMiIrPwD | 1 Unknown | 1 Asymptomatic  1 No MIS-C  AND  1 No comorbidities | 1 Not reported | 1 No treatment | 0 | 0 | 0 | (NOS, 7)  1 survived |
| Topal et al. 2022 ([124](#_ENREF_124)), Turkey | Retrospective cohort, single centre | 168 | 1 Gender was not reported | 1 White (Caucasian) | 1 Isotype, light chain, or functional deficiency with generally normal numbers of B cells (Low IgM level) | 1 NSGDwR | 1 NOPMiIrPwD | 1 Unknown | 1 Mild  1 No MIS-C  AND  1 Allergic rhinitis | 1 Not reported | 1 No treatment | 0 | 0 | 0 | (NOS, 7)  1 survived |
| Castano-Jaramillo et al. 2021 ([40](#_ENREF_40)), Mexico | Retrospective cohort, multi-centre | 180 | 0 (0) | 1 Hispanic | 1 Severe reduction in serum IgG and IgA with normal/elevated IgM and normal numbers of B cells (APRIL deficiency) | TNFSF13 (n=1) | 1 NOPMiIrPwD | 1 AR | 1 Moderate  1 No MIS-C  AND  1 CLD | 1 Thrombocytopenia  1 Low cardiac ejection fraction and dilation of the left coronary artery | 1 IVIG  1 Steroids | 1 | 0 | 0 | (NOS, 8)  1 survived |
| Abolhassani et al. 2022 ([19](#_ENREF_19)), Iran | Prospective cohort, single centre | 180 | 1 (100) | 1 Persian | 1 Severe reduction in serum IgG and IgA with normal/elevated IgM and normal numbers of B cells (UNG deficiency) | UNG (n=1) | Lymphocyte development/epigenetic: KMT2A (n=1)  IL-1 activation pathway genes: TNFRSF11A (n=1) | 1 AR | 1 Severe  1 Yes MIS-C  AND  1 Not reported | 1 Low serum IgA and IgG levels | 1 Antibiotics  1 IVIG  1 ACEIs  1 Biological agents  1 Antiplatelets  1 TPN | 1 | 0 | 0 | (NOS, 8)  1 survived |
| [Goudouris](https://scholar.google.com/citations?user=nyuqBh4AAAAJ&hl=en&oi=sra) et al. 2021 ([65](#_ENREF_65)), Brazil | Retrospective cohort, multi-centre | 70 and 110 | 2 (100) | 2 Hispanics | 2 Predominantly antibody deficiency (types were not reported) | 2 NSGDwR | 1 NOPMiIrPwD | 2 Unknowns | 2 Mild  2 No MIS-C  AND  2 No comorbidities | 2 Not reported | 1 IVIG | 0 | 0 | 0 | (NOS, 8)  2 survived |
| Meyts et al. 2021 ([95](#_ENREF_95)), 10 countries | Retrospective cohort, multi-centre | Age groups: 36-144 (n=1) | 1 (100) | 1 White (Caucasian) | 1 Predominantly antibody deficiency (type was not reported) | 1 NSGDwR | 1 NOPMiIrPwD | 1 Unknown | 1 Mild  1 No MIS-C  AND  1 Lymphopenia  1 HLH  1 Cognitive disability  1 Dysmorphism  1 CHD  1 Hypercholesterolemia | 1 Not reported | 1 Antibiotics | 0 | 0 | 0 | (NOS, 8)  1 survived |
| **IUIS IEIs category: Immune dysregulatory diseases (Group IV)** | | | | | | | | | | | | | | | |
| Alhumaidan et al. 2021 ([29](#_ENREF_29)), Saudi Arabia | Retrospective case report, single centre | 2 | 0 (0) | 1 Arab | 1 FHL syndromes (HLH) | UNC13D (n=1) | 1 NOPMiIrPwD | 1 AR | 1 Severe  1 Yes MIS-C  AND  1 Atelectasis  1 Sepsis | 1 High NT-proBNP  1 High interleukin-6  1 High TNF-a  1 High ferritin | 1 Antibiotics  1 Steroids  1 Tocilizumab  1 Etoposide 1 Anakinra | 1 | 1 | 1 | (Modified NOS, high)  1 died (COVID-19-related) |
| [Goudouris](https://scholar.google.com/citations?user=nyuqBh4AAAAJ&hl=en&oi=sra) et al. 2021 ([65](#_ENREF_65)), Brazil | Retrospective cohort, multi-centre | 15 | 1 (100) | 1 Hispanic | 1 FHL syndromes (HLH) | 1 NSGDwR | 1 NOPMiIrPwD | 1 AR | 1 Asymptomatic  1 No MIS-C  AND  1 No comorbidities | 1 Not reported | 1 IVIG | 0 | 0 | 0 | (NOS, 8)  1 survived |
| Greenmyer et al. 2022 ([67](#_ENREF_67)), United States | Retrospective case report, single centre | 60 | 0 (0) | 1 White (Caucasian) | 1 FHL syndromes (HLH) | 1 NSGDwR | 1 NOPMiIrPwD | 1 AR | 1 Mild  1 No MIS-C  AND  1 HLH  1 Ascites  1 Engraftment syndrome  1 Adrenal insufficiency  1 Veno-occlusive disease  1 Transplant-associated thrombotic microangiopathy | 1 Low Hb  1 High WBCs  1 Raised liver enzymes  1 High CRP  1 High ESR  1 Raised procalcitonin  1 High ferritin  1 High triglycerides  1 Low NK cells  1 High interleukin-2 | 1 Antibiotics  1 Etoposide  1 Steroids  1 Methotrexate  1 Emapalumab  1 HSCT  1 Defibrotide 1 Eculizumab | 1 | 1 | 0 | (Modified NOS, high)  1 survived |
| Haigh et al. 2020 ([6](#_ENREF_6)), United Kingdom | Retrospective case report, single centre | 204 | 1 (100) | 1 White (Caucasian) | 1 FHL syndromes (HLH) | 1 NSGDwR | 1 NOPMiIrPwD | 1 AR | 1 Mild  1 No MIS-C  AND  1 HLH  1 Sepsis | 1 Low RBCs  1 Thrombocytopenia  1 Low WBCs  1 Hyponatraemia  1 Hypocalcaemia  1 Raised liver enzymes  1 Increased lactate  1 High CRP  1 High ferritin  1 High triglycerides  1 High fibrinogen  1 High D-dimer | 1 Anakinra  1 IVIG  1 Antibiotics | 0 | 0 | 0 | (Modified NOS, high)  1 survived |
| Kalita et al. 2021 ([72](#_ENREF_72)), India | Retrospective case report, single centre | 24 | 1 (100) | 1 Indian | 1 FHL syndromes (HLH) | 1 NSGDwR | 1 NOPMiIrPwD | 1 AR | 1 Mild  1 No MIS-C  AND  1 HLH  1 Abnormal body movements  1 Feeding intolerance  1 Decreased neck control | 1 Low Hb  1 Leukopenia  1 Thrombocytopenia  1 High ferritin  1 Raised liver enzymes  1 High CRP  1 High D-dimer  1 High interleukin-6  1 High fibrinogen  1 Increased iron | 1 Antiepileptics  1 Antibiotics  1 Steroids  1 IV fluids | 1 | 0 | 0 | (Modified NOS, low)  1 outcome was not reported |
| Kim-Hellmuth et al. 2021 ([79](#_ENREF_79)), Germany | Retrospective case report, single centre | 36 | 1 (100) | 1 Black | 1 FHL syndromes (HLH) | 1 NSGDwR | 1 NOPMiIrPwD | 1 AR | 1 Severe  1 No MIS-C  AND  1 HLH  1 Down syndrome  1 Atrioventricular septal defect  1 Pulmonary hypertension | 1 Low Hb  1 Neutrophilia  1 Raised liver enzymes  1 High CRP  1 Raised procalcitonin  1 High interleukin-6  1 Low T3 and T4 levels  1 Thrombocytopenia  1 High triglycerides  1 High ferritin  1 High fibrinogen | 1 Steroids  1 IVIG  1 Remdesivir  1 Antibiotics  1 IV inotropes  1 IV fluids  1 Oxygen supplementation  1 Prone positioning | 1 | 1 | 1 | (Modified NOS, high)  1 survived |
| Kołtan et al. 2022 ([81](#_ENREF_81)), Poland | Retrospective cohort, multi-centre | 1 Not possible to extract | 1 Not possible to extract | 1 White (Caucasian) | 1 FHL syndromes (HLH) | 1 NSGDwR | 1 NOPMiIrPwD | 1 AR | 1 Asymptomatic  1 No MIS-C  AND  1 Not possible to extract | 1 Not reported | 1 Not possible to extract | 0 | 0 | 0 | (NOS, 7)  1 survived |
| Kosmeri et al. 2022 ([82](#_ENREF_82)), Greece | Retrospective case report, single centre | 1 | 1 (100) | 1 White (Caucasian) | 1 FHL syndromes (HLH) | 1 NSGDwR | 1 NOPMiIrPwD | 1 AR | 1 Mild  1 No MIS-C  AND  1 HLH  1 Coagulase-negative *Staphylococcus* coinfection  1 Gaucher disease  1 Persistent thrombocytopenia  1 Hypotonia  1 Diminished motor activity  1 Increased muscle tone  1 Opisthotonus  1 Oculomotor apraxia  1 Jaundice  1 Acquired coagulopathy  1 Bleeding  1 Sepsis | 1 Anaemia  1 Low Hb  1 Raised liver enzymes  1 Hyperbilirubinemia  1 Platelet transfusions  1 High D-dimer  1 Low fibrinogen  1 High ferritin  1 High interleukin-10  1 High TNF-a | 1 IVIG  1 Etoposide  1 Steroids  1 Ursodeoxycholic acid  1 Antibiotics  1 Chemotherapy | 1 | 1 | 1 | (Modified NOS, high)  1 died (not COVID-19-related) |
| Mahdavi et al. 2022 ([88](#_ENREF_88)), Iran | Retrospective case report, single centre | 96 | 1 (100) | 1 Persian | 1 FHL syndromes (HLH) | 1 NSGDwR | 1 NOPMiIrPwD | 1 AR | 1 Mild  1 No MIS-C  AND  1 HLH  1 Heart failure  1 DIC  1 Myocarditis  1 Cardiomyopathy  1 Coagulopathy  1 Thrombocytosis  1 Cardiac graft rejection | 1 Leucocytosis  1 Lymphopenia  1 High WBCs  1 Anaemia  1 Low Hb  1 Thrombocytopenia  1 High ESR  1 High CRP  1 Hyperbilirubinemia  1 High LDH  1 High ferritin  1 High D-dimer  1 High triglycerides  1 High NT-proBNP  1 Elevated CD25  1 Increased lactate | 1 IVIG  1 Steroids  1 Heparin  1 Heart transplantation  1 Mycophenolate mofetil  1 Everolimus  1 Anti-thymocyte globulin  1 Antibiotics  1 Lopinavir/ritonavir  1 Rituximab | 1 | 1 | 1 | (Modified NOS, high)  1 died (not COVID-19-related) |
| Quintana-Ortega et al. 2021 ([110](#_ENREF_110)), Spain | Retrospective case report, single centre | 132 | 0 (0) | 1 White (Caucasian) | 1 FHL syndromes (HLH) | 1 NSGDwR | 1 NOPMiIrPwD | 1 AR | 1 Mild  1 No MIS-C  AND  1 CLD  1 Interstitial lung disease  1 Anti-MDA5 juvenile dermatomyositis  1 *Pneumocystis jirovecii* coinfection  1 Pneumomediastinum  1 Pneumothorax  1 Emphysema  1 Respiratory failure  1 Septic shock | 1 Not reported | 1 Antibiotics  1 Steroids  1 IVIG  1 Cyclophosphamide  1 Tofacitinib | 1 | 1 | 1 | (Modified NOS, high)  1 died (not COVID-19-related) |
| Vagrecha et a. 2022 ([125](#_ENREF_125)), United States | Retrospective cohort, single centre | 96 | 1 (100) | 1 Black | 1 FHL syndromes (HLH) | UNC13D (n=1) | c.796C>T (n=1) | 1 AR | 1 Not reported  1 Yes MIS-C  AND  1 No comorbidities | 1 Not possible to extract | 1 Not possible to extract | 1 Not possible to extract | 1 Not possible to extract | 1 Not possible to extract | (NOS, 7)  1 outcome was not reported |
| [Goudouris](https://scholar.google.com/citations?user=nyuqBh4AAAAJ&hl=en&oi=sra) et al. 2021 ([65](#_ENREF_65)), Brazil | Retrospective cohort, multi-centre | 61 | 0 (0) | 1 Hispanic | 1 FHL Syndromes with hypopigmentation (Chediak-Higashi syndrome) | LYST (n=1) | 1 NOPMiIrPwD | 1 AR | 1 Mild  1 No MIS-C  AND  1 Arterial hypertension | 1 Not reported | 1 No treatment | 0 | 0 | 0 | (NOS, 8)  1 survived |
| Lange et al. 2021 ([84](#_ENREF_84)), Germany | Retrospective case report, single centre | 1 | 0 (0) | 1 White (Caucasian) | 1 FHL Syndromes with hypopigmentation (Chediak-Higashi syndrome) | LYST (n=1) | c5023G>T (n=1) | 1 AR | 1 Moderate  1 No MIS-C  AND  1 HLH  1 Poor feeding  1 Abdominal distension  1 Pleural effusion  1 Hypopigmentation of scalp hair | 1 Anaemia  1 Low Hb  1 Thrombocytopenia  1 Low NK cells  1 Neutropenia  1 Low fibrinogen  1 High triglycerides  1 High ferritin  1 High interleukin-2 | 1 Platelet transfusion  1 Vitamin K  1 Clotting factors  1 Steroids  1 Etoposide  1 Cyclosporine  1 HSCT | 1 | 0 | 0 | (Modified NOS, high)  1 survived |
| Mohanty et al. 2021 ([99](#_ENREF_99)), India | Retrospective case report, single centre | 118 | 0 (0) | 1 Indian | 1 FHL Syndromes with hypopigmentation (Chediak-Higashi syndrome) | LYST (n=1) | c.9162G > A (n=1) | 1 AR | 1 Mild  1 No MIS-C  AND  1 No comorbidities | 1 Not reported | 1 No treatment | 0 | 0 | 0 | (Modified NOS, high)  1 survived |
| Vagrecha et a. 2022 ([125](#_ENREF_125)), United States | Retrospective cohort, single centre | 84 | 1 (100) | 1 Black | 1 FHL Syndromes with hypopigmentation (Chediak-Higashi syndrome) | LYST (n=1) | c.10669G>T (n=1) | 1 AR | 1 Not reported  1 Yes MIS-C  AND  1 No comorbidities | 1 Not possible to extract | 1 Not possible to extract | 1 Not possible to extract | 1 Not possible to extract | 1 Not possible to extract | (NOS, 7)  1 outcome was not reported |
| Abolhassani et al. 2022 ([19](#_ENREF_19)), Iran | Prospective cohort, single centre | 144 | 1 (100) | 1 Persian | 1 FHL syndromes (STXBP2 / Munc18-2 deficiency) | STXBP2 (n=1) | Lymphocyte development/epigenetic: KMT2D (n=1)  DNA repair gene defect: ATM (n=1) | 1 AR | 1 Severe  1 No MIS-C  AND  1 Not reported | 1 Low serum IgA and IgG levels | 1 Antibiotics 1 Steroids  1 TPN | 1 | 0 | 0 | (NOS, 8)  1 survived |
| Karakoc Aydiner et al. 2022 ([73](#_ENREF_73)), Turkey | Prospective cohort, multi-centre | 42 | 0 (0) | 1 White (Caucasian) | 1 FHL syndromes (STXBP2 / Munc18-2 deficiency) | STXBP2 (n=1) | 1 NOPMiIrPwD | 1 AR | 1 Moderate  1 Yes MIS-C  AND  1 IBD  1 HLH  1 Post HSCT  1 Hypertension | 1 Leukopenia  1 High CRP  1 Raised procalcitonin  1 High LDH | 1 IVIG  1 Antibiotics | 0 | 0 | 0 | (NOS, 7)  1 survived |
| Vagrecha et a. 2022 ([125](#_ENREF_125)), United States | Retrospective cohort, single centre | 36 | 0 (0) | 1 Black | 1 FHL syndromes (STXBP2 / Munc18-2 deficiency) | STXBP2 (n=1) | c.1772C>A (n=1) | 1 AR | 1 Not reported  1 Yes MIS-C  AND  1 No comorbidities | 1 Not possible to extract | 1 Not possible to extract | 1 Not possible to extract | 1 Not possible to extract | 1 Not possible to extract | (NOS, 7)  1 outcome was not reported |
| Delavari et al. 2021 ([48](#_ENREF_48)), Iran | Prospective cohort, multi-centre | 106 | 0 (0) | 1 Persian | 1 FHL Syndromes with hypopigmentation (Griscelli syndrome, type 2) | RAB27A (n=1) | 1 NOPMiIrPwD | 1 AR | 1 Severe  1 Yes MIS-C  AND  1 AIHA  1 Severe anal ulcer  1 Hodgkin’s lymphoma  1 Liver involvement  1 Pitting oedema | 1 Leukopenia  1 Low Hb  1 Thrombocytopenia | 1 Antibiotics  1 Hydroxychloroquine | 1 | 1 | 1 | (NOS, 7)  1 died (COVID-19-related) |
| Gelzo et al. 2022 ([62](#_ENREF_62)), Italy | Retrospective cohort, single centre | 60, 108, 108, 60, and 168 | 3 (60) | 5 Whites (Caucasians) | 5 FHL syndromes (Perforin deficiencies) | PRF1 (n=5) | c.272C>T (n=5) | 5 ARs | 5 Not reported  5 Yes MIS-C  AND  1 HLH | 5 Not reported | 5 Not reported | 5 Not reported | 5 Not reported | 5 Not reported | (NOS, 7)  5 outcome was not reported |
| Vagrecha et a. 2022 ([125](#_ENREF_125)), United States | Retrospective cohort, single centre | 168 | 0 (0) | 1 Black | 1 FHL syndrome (Perforin deficiency) | PRF1 (n=1) | c.1424G>A (n=1) | 1 AR | 1 Not reported  1 Yes MIS-C  AND  1 No comorbidities | 1 Not possible to extract | 1 Not possible to extract | 1 Not possible to extract | 1 Not possible to extract | 1 Not possible to extract | (NOS, 7)  1 outcome was not reported |
| Bastard et al. 2021 ([35](#_ENREF_35)), 7 countries | Retrospective cohort, multi-centre | 156, 168, 96, 192, 96, 132, 216, 180, and 120 | 5 (55.5) | 9 Whites (Caucasians) | 9 Autoimmunity with or without lymphoproliferation (APS-1) | AIRE (n=9) | R257X (n=5)  c.242T>C (n=1)  3 NOPMiIrPwD | 8 ARs  1 AD | 4 Mild  2 Moderate  3 Severe  9 No MIS-C  AND  9 CMC  7 Adrenal insufficiency  3 Enteropathy  7 Hypoparathyroidism  3 Hepatitis  2 Diabetes mellitus  1 Asthma  1 Pancreatitis  1 Nephritis  1 Transient diabetes insipidus  1 Vitiligo  1 Gastrointestinal bleeding  1 Lupus-like systemic inflammation  1 Hypothyroidism  1 Hypogonadism  1 Ovarian insufficiency | 1 Pernicious anaemia  5 High CRP  3 High LDH  2 Raised liver enzymes  3 Lymphopenia  3 High D-dimer  1 High interleukin-6  1 High ferritin | 5 Steroids  2 Tocilizumab  2 Antibiotics  2 Antifungals  2 Plasmapheresis  2 IFN-β  2 Convalescent plasma  1 IVIG  1 Ribavirin | 3 | 2 | 5 | (NOS, 7)  9 survived |
| Carpino et al. 2021 ([38](#_ENREF_38)), Italy | Retrospective case report, single centre | 168 | 0 (0) | 1 White (Caucasian) | 1 Autoimmunity with or without lymphoproliferation (APS-1) | AIRE (n=1) | p.Leu87Pro/ p.Leu323fs (n=1) | 1 AR | 1 Asymptomatic  1 No MIS-C  AND  1 Hypoparathyroidism  1 Autoimmune insulitis  1 CMC  1 Vitiligo  1 Enamel hypoplasia  1 Abdominal pain  1 Bilateral swelling of the feet  1 Hypogonadism  1 Growth impairment  1 Pubertal delay | 1 Low calcium  1 High phosphorus  1 Low parathyroid hormone  1 Raised liver enzymes | 1 No treatment | 0 | 0 | 0 | (Modified NOS, high)  1 survived |
| Karakoc Aydiner et al. 2022 ([73](#_ENREF_73)), Turkey | Prospective cohort, multi-centre | 31 | 0 (0) | 1 White (Caucasian) | 1 Autoimmunity with or without lymphoproliferation (APS-1) | AIRE (n=1) | 1 NOPMiIrPwD | 1 AR | 1 Moderate  1 Yes MIS-C  AND  1 Multi-organ failure  1 Bronchiolitis obliterans  1 Autoimmunity | 1 Leukopenia  1 High CRP  1 High ESR  1 High ferritin  1 High LDH  1 High D-dimer | 1 Antifungals  1 IVIG | 1 | 1 | 1 | (NOS, 7)  1 died (COVID-19-related) |
| Lemarquis et al. 2021 ([86](#_ENREF_86)), Sweden | Retrospective case report, single centre | 96 | 0 (0) | 1 White (Caucasian) | 1 Autoimmunity with or without lymphoproliferation (APS-1) | AIRE (n=1) | c.1616C>T (n=1) | 1 AR | 1 Severe  1 No MIS-C  AND  1 Type I IFN deficiency  1 Hypoparathyroidism  1 Vitiligo  1 Addison disease | 1 Not reported | 1 Plasmapheresis  1 IVIG  1 Steroids  1 Anticoagulant | 1 | 1 | 1 | (Modified NOS, high)  1 survived |
| Meisel et al. 2021 ([94](#_ENREF_94)), Germany | Retrospective case report, single centre | 167, 164, 186 and 170 | 1 (25) | 2 Whites (Caucasians)  2 Arabs | 4 Autoimmunity with or without lymphoproliferation (APS-1) | AIRE (n=4) | c.784delC (n=1)  c.1096-1G>A (n=1)  c.769C>T (n=1)  c.1096-1G>A (n=1) | 4 ARs | 4 Mild  4 No MIS-C  AND  3 Adrenal insufficiency  3 Hypoparathyroidism  2 Hypergonadotropic hypogonadism  1 CMC  1 Meningoencephalitis  1 Autoimmune encephalitis  1 Atrophic gastritis  1 Growth hormone deficiency  1 Unilateral parotitis  1 Retinal degeneration  1 Optical atrophy | 4 Positive autoantibodies against IFN-α and IFN-ω | 4 No treatment | 0 | 0 | 0 | (Modified NOS, high)  4 survived |
| Oza et al. 2021 ([102](#_ENREF_102)), India | Retrospective case report, single centre | 192 | 1 (100) | 1 Indian | 1 Autoimmunity with or without lymphoproliferation (APS-1) | AIRE (n=1) | 1 NOPMiIrPwD | 1 AR | 1 Severe  1 No MIS-C  AND  1 Hypoparathyroidism  1 Diabetes mellitus type 1  1 Hepatitis  1 Adrenal insufficiency  1 Liver cirrhosis  1 Portal hypertension  1 Oesophageal varices | 1 Raised liver enzymes  1 Low Hb  1 Hyperglycaemia  1 High HbA1c  1 Low vitamin D | 1 Steroids  1 Oxygen supplementation  1  1 Remdesivir  1 Heparin  1 IV inotropes  1 Antibiotics  1 Insulin  1 Vitamin D  1 Calcium  1 Propranolol 1 Azathioprine | 1 | 0 | 0 | (Modified NOS, high)  1 survived |
| Schidlowski et al. 2022 ([113](#_ENREF_113)),  Brazil | Retrospective case report, single centre | 156 and 84 | 2 (100) | 2 Whites (Caucasians) | 2 Autoimmunity with or without lymphoproliferation (APS-1) | AIRE (n=2) | c.C796T- p.R257* (n=2) | 2 ARs | 2 Severe  2 No MIS-C  AND  2 Type I IFN deficiency  1 Hypothyroidism  1 Asthma  2 Pulmonary haemorrhage  1 Emphysema  1 Tremors on movement  1 Ataxia  1 Gait disturbance  1 Urinary and faecal incontinence | 2 Lymphopenia  2 High CRP  2 High D-dimer  2 Raised liver enzymes | 2 Antibiotics  2 Steroids  1 Analgesics  1 Bronchodilators  2 Oxygen supplementation  1 Oseltamivir  1 IV inotropes  3 Heparin  1 Lorazepam 1 Haloperidol 2 Convalescent plasma  1 Midazolam 1 Morphine 1 Rocuronium 1 Ketamine | 2 | 2 | 2 | (Modified NOS, high)  2 survived |
| Deyà-Martínez et al. 2021 ([50](#_ENREF_50)), Spain | Prospective cohort, single centre | 204 | 1 (100) | 1 White (Caucasian) | 1 Autoimmune lymphoproliferative syndrome (ALPS syndrome) | 1 NSGDwR | 1 NOPMiIrPwD | 1 Unknown | 1 Mild  1 No MIS-C  AND  1 No comorbidities | 1 Not reported | 1 No treatment | 0 | 0 | 0 | (NOS, 7)  1 survived |
| Kołtan et al. 2022 ([81](#_ENREF_81)), Poland | Retrospective cohort, multi-centre | 4 Not possible to extract | 4 Not possible to extract | 4 Whites (Caucasians) | 4 Autoimmune lymphoproliferative syndromes (ALPS syndromes) | 4 NSGDwR | 4 NOPMiIrPwD | 4 Unknowns | 3 Mild  1 Asymptomatic  4 No MIS-C  AND  4 Not possible to extract | 4 Not reported | 4 Not possible to extract | 0 | 0 | 0 | (NOS, 7)  4 survived |
| Meyts et al. 2021 ([95](#_ENREF_95)), 10 countries | Retrospective cohort, multi-centre | Age groups:36-144 (n=1) and 156-216 (n=1) | 1 (50) | 2 Whites (Caucasians) | 2 Autoimmune lymphoproliferative syndromes (ALPS syndromes) | 2 NSGDwR | 2 NOPMiIrPwD | 2 Unknowns | 1 Asymptomatic  1 Mild  2 No MIS-C  AND  1 Immune thrombocytopenia  1 Jaundice | 1 Anaemia  1 Raised liver enzymes | 1 Antibiotics  1 Chloroquine | 0 | 0 | 0 | (NOS, 8)  2 survived |
| Milito et al. 2021 ([97](#_ENREF_97)), Italy | Retrospective cohort, multi-centre | Age group: <216 (n=1) | 1 Not possible to extract | 1 Whites (Caucasians) | 1 Autoimmune lymphoproliferative syndrome (ALPS syndrome) | 1 NSGDwR | 1 NOPMiIrPwD | 1 Unknown | 1 Mild  1 No MIS-C  AND  1 Not possible to extract | 1 Not reported | 1 Not reported | 0 | 0 | 0 | (NOS, 7)  1 survived |
| Yadav et al. 2022 ([130](#_ENREF_130)), India | Retrospective case report, single centre | 180 | 1 (100) | 1 Indian | 1 Autoimmune lymphoproliferative syndrome (ALPS syndrome) | 1 NSGDwR | 1 NOPMiIrPwD | 1 Unknown | 1 Mild  1 No MIS-C  AND  1 No comorbidities | 1 Thrombocytopenia  1 Neutropenia  1 Low Hb  1 Leukopenia | 1 No treatment | 0 | 0 | 0 | (Modified NOS, high)  1 survived |
| Fernández-Suárez et al. 2021 ([57](#_ENREF_57)), Peru | Retrospective case report, single centre | 144 | 1 (100) | 1 Hispanic | 1 Regulatory T cell defect (LRBA deficiency) | LRBA deficiency (n=1) | 1 NOPMiIrPwD | 1 AR | 1 Severe  1 No MIS-C  AND  1 *Stenotrophomonas maltophilia* coinfection  1 *Candida albicans* coinfection  1 Muscle atrophy and decreased strength | 1 Raised urea  1 Elevated creatinine  1 Raised liver enzymes  1 High LDH  1 High CRP  1 Lymphopenia  1 High D-dimer  1 High fibrinogen | 1 Antibiotics  1 IVIG  1 Steroids  1 Antifungals  1 Oxygen supplementation | 1 | 1 | 1 | (Modified NOS, high)  1 survived |
| Fetyan et al. 2022 ([58](#_ENREF_58)), United Arab Emirates | Retrospective case report, single centre | 168 | 1 (100) | 1 Arab | 1 Regulatory T cell defect (LRBA deficiency) | LRBA deficiency (n=1) | c.534del; p.9Asp179IIef*16 (n=1) | 1 AR | 1 Asymptomatic  1 No MIS-C  AND  1 *Cytomegalovirus* colitis  1 Septic arthritis  1 Hypothyroidism  1 Autoimmune cytopenia  1 AIHA  1 IBD  1 Atopic dermatitis  1 Arthritis  1 Splenectomy  1 Profuse rectal bleeding  1 Rectal prolapse  1 ITP  1 CLD | 1 Anaemia  1 Thrombocytopenia  1 Hypogammaglobinaemia  1 Low serum IgA, IgM and IgG levels  1 Lymphopenia  1 Leukocytosis | 1 Antibiotics 1 IVIG  1 Pentamidine  1 Foscarnet 1 Ganciclovir  1 Sirolimus  1 Mycophenolate Mofetil  1 Azathioprine 1 Rituximab | 1 | 1 | 1 | (Modified NOS, high)  1 died (not COVID-19-related) |
| Sener et al. 2022 ([115](#_ENREF_115)), Turkey | Retrospective case-series, single centre | 168 | 1 (100) | 1 Whites (Caucasians) | 1 Regulatory T cell defect (LRBA deficiency) | LRBA deficiency (n=1) | 1 NOPMiIrPwD | 1 AR | 1 Mild  1 Yes MIS-C  AND  1 Hypertension  1 Seizures  1 Juvenile idiopathic arthritis  1 IBD  1 Tubulointerstitial nephritis  1 Posterior reversible encephalopathy syndrome | 1 Low Hb  1 Leukocytosis  1 Neutrophilia  1 Thrombocytopenia  1 High ESR  1 High CRP  1 High ferritin  1 High interleukin-6  1 High Troponine I  1 High NT-proBNP | 1 IVIG  1 Steroids  1 Anakinra | 0 | 0 | 0 | (Modified NOS, high)  1 survived |
| Vagrecha et a. 2022 ([125](#_ENREF_125)), United States | Retrospective cohort, single centre | 48, 84 and 24 | 3 (100) | 3 Blacks | 3 Regulatory T cell defects (LRBA deficiency) | LRBA deficiency (n=3) | c.3407C>T (n=1)  c.4930C>G (n=1)  c.6703C>T (n=1)  c.4918G>A (n=1)  c.1161+4G>T (n=1) | 3 ARs | 3 Not reported  3 Yes MIS-C  AND  3 No comorbidities | 3 Not possible to extract | 3 Not possible to extract | 3 Not possible to extract | 3 Not possible to extract | 3 Not possible to extract | (NOS, 7)  3 outcome was not reported |
| Stockdale et al. 2021 ([121](#_ENREF_121)), United Kingdom | Retrospective case report, single centre | 180 | 0 (0) | 1 Pakistani | 1 Autoimmunity with or without lymphoproliferation (TPP2 deficiency) | TPP2 (n=1) | c.1913 + 5G > A (n=1) | 1 AR | 1 Mild  1 No MIS-C  AND  1 Asthma  1 Eczema  1 Developmental delay  1 Seizures  1 Warts  1 Recurrent paronychia  1 Epistaxis  1 Dysphagia  1 Palpitations  1 ITP | 1 Thrombocytopenia  1 Low levels of naïve (CD45RA+/CD27+) CD4 and CD8 T cells  1 Low levels of non-switched (CD19+ CD27+ IgD+ 1.21%) and class-switched (CD19+ CD27+ IgD− 2.81%) memory B cells | 1 IVIG  1 Steroids  1 Sirolimus  1 Eltrombopag | 0 | 0 | 0 | (Modified NOS, high)  1 survived |
| Shields et al. 2022 ([118](#_ENREF_118)), United Kingdom | Retrospective cohort, multi-centre | Age group: (0 to 216) (n=1) | 0 (0) | 1 White (Caucasian) | 1 Autoimmunity with or without lymphoproliferation (TPP2 deficiency) | TPP2 (n=1) | 1 NOPMiIrPwD | 1 AR | 1 Not reported  1 Not reported  AND  1 No comorbidities | 1 Not possible to extract | 1 Not possible to extract | 0 | 0 | 0 | (NOS, 8)  1 survived |
| Vagrecha et a. 2022 ([125](#_ENREF_125)), United States | Retrospective cohort, single centre | 204, 84 and 168 | 2 (66.7) | 2 Blacks  1 Asian | 3 Autoimmunity with or without lymphoproliferation (TPP2 deficiency) | TPP2 (n=3) | c.1732T>C (n=1)  c.3526C>G (n=1)  c.340A>G (n=1) | 3 ARs | 3 Not reported  3 Yes MIS-C  AND  3 No comorbidities | 3 Not possible to extract | 3 Not possible to extract | 3 Not possible to extract | 3 Not possible to extract | 3 Not possible to extract | (NOS, 7)  3 outcome was not reported |
| Chung et al. 2022 ([44](#_ENREF_44)), Hong Kong | Retrospective case report, single centre | 60 | 1 (100) | 1 Asian | 1 Susceptibility to *Epstein–Barr virus* and lymphoproliferative conditions (XLP1) | SH2D1A deficiency (n=1) | 1 NOPMiIrPwD | 1 X-linked | 1 Severe  1 No MIS-C  AND  1 Agammaglobulinemia  1 Respiratory failure  1 Multi-organ failure | 1 Low serum IgA, IgG and IgM levels  1 Increased T-cells  1 Low NK cells  1 Reversed CD4 to CD8 ratio | 1 IVIG  1 Antibiotics  1 Steroids  1 Ribavirin  1 IV inotropes | 1 | 1 | 1 | (Modified NOS, high)  1 died (COVID-19-related) |
| Esslami et al. 2021 ([54](#_ENREF_54)), Iran | Retrospective case report, single centre | 54 | 1 (100) | 1 Persian | 1 Susceptibility to *Epstein–Barr virus* and lymphoproliferative conditions (XLP1) | SH2D1A deficiency (n=1) | 1 NOPMiIrPwD | 1 X-linked | 1 Moderate  1 No MIS-C  AND  1 Post HSCT  1 Bacterial coinfections  1 DIC  1 Septic shock | 1 Not reported | 1 Steroids  1 Cyclosporine 1 Antibiotics 1 Antifungals 1 Valganciclovir  1 Tranexamic acid  1 Oseltamivir 1 Hydroxychloroquine  1 Acyclovir | 1 | 1 | 1 | (Modified NOS, high)  1 died (not COVID-19-related) |
| Kołtan et al. 2022 ([81](#_ENREF_81)), Poland | Retrospective cohort, multi-centre | 1 Not possible to extract | 1 Not possible to extract | 1 White (Caucasian) | 1 Susceptibility to *Epstein–Barr virus* and lymphoproliferative conditions (XLP1) | SH2D1A deficiency (n=1) | 1 NOPMiIrPwD | 1 X-linked | 1 Mild  1 No MIS-C  AND  1 Not possible to extract | 1 Not reported | 1 Not possible to extract | 0 | 0 | 0 | (NOS, 7)  1 survived |
| Prader et al. 2021 ([109](#_ENREF_109)), Switzerland | Retrospective case report, single centre | 72 | 1 (100) | 1 White (Caucasian) | 1 Susceptibility to *Epstein–Barr virus* and lymphoproliferative conditions (XLP1) | SH2D1A deficiency (n=1) | c.163C > T (n=1) | 1 X-linked | 1 Moderate  1 No MIS-C  AND  1 HLH  1 *Epstein-Barr virus* coinfection  1 Liver failure  1 Arthritis  1 Arthromyalgias  1 Abdominal pain  1 Hypotension  1 Ascites  1 Pulmonary oedema  1 Lactacidemia  1 Acute kidney injury  1 Coagulopathy  1 Shock | 1 Low fibrinogen  1 Anaemia  1 Low Hb  1 Thrombocytopenia  1 High ESR  1 High ferritin  1 Raised liver enzymes  1 High LDH  1 Hyponatremia  1 High D-dimer  1 Lymphopenia  1 High triglycerides  1 Positive antinuclear antibody  1 Positive anti-MDA5  1 Positive anti-SS-A | 1 Steroids  1 IVIG  1 Antibiotics  1 Hydroxychloroquine  1 Remdesivir  1 Tocilizumab  1 Oxygen supplementation  1 Cyclophosphamide  1 Anakinra  1 Anti-thymocyte globulin  1 Ciclosporin  1 Rituximab  1 Hemofiltration | 1 | 1 | 1 | (Modified NOS, high)  1 died (not COVID-19-related) |
| Chou et al. 2021 ([43](#_ENREF_43)), United States | Prospective cohort, single centre | 132 | 1 (100) | 1 White (Caucasian) | 1 Susceptibility to *Epstein–Barr virus* and lymphoproliferative conditions (XLP2) | XIAP deficiency (n=1) | 1 NOPMiIrPwD | 1 X-linked | 1 Mild  1 No MIS-C  AND  1 *Epstein-Barr virus* coinfection  1 Obesity  1 Cardiogenic shock  1 Third-degree heart block | 1 Neutrophilia  1 Lymphopenia  1 High interleukin-6  1 High interleukin-18  1 High interleukin-10  1 Elevated level of C-X-C motif chemokine ligand 9 | 1 Remdesivir  1 Anakinra  1 Steroids  1 IVIG  1 Vasopressors  1 Rituximab | 0 | 0 | 0 | (NOS, 7)  1 survived |
| [Goudouris](https://scholar.google.com/citations?user=nyuqBh4AAAAJ&hl=en&oi=sra) et al. 2021 ([65](#_ENREF_65)), Brazil | Retrospective cohort, multi-centre | 31 | 1 (100) | 1 Hispanic | 1 Susceptibility to *Epstein–Barr virus* and lymphoproliferative conditions (XLP2) | XIAP deficiency (n=1) | 1 NOPMiIrPwD | 1 X-linked | 1 Critical  1 No MIS-C  AND  1 Early-onset IBD | 1 Not reported | 1 Not reported | 1 | 1 | 1 | (NOS, 8)  1 died (COVID-19-related) |
| Meyts et al. 2021 ([95](#_ENREF_95)), 10 countries | Retrospective cohort, multi-centre | Age groups: 156-216 (n=1) | 1 (100) | 1 Hispanic | 1 Susceptibility to *Epstein–Barr virus* and lymphoproliferative conditions (XLP2) | XIAP deficiency (n=1) | 1 NOPMiIrPwD | 1 X-linked | 1 Mild  1 No MIS-C  AND  1 HLH  1 Post HSCT  1 Graft versus host disease (gut allograft)  1 Sepsis | 1 Not reported | 1 Antibiotics  1 IVIG | 1 | 1 | 1 | (NOS, 8)  1 died (not COVID-19-related) |
| Narahari et al. 2022 ([100](#_ENREF_100)), United States | Retrospective case report, single centre | 27 | 1 (100) | 1 White (Caucasian) | 1 Susceptibility to *Epstein–Barr virus* and lymphoproliferative conditions (XLP2) | XIAP deficiency (n=1) | c.978-2A > G (n=1) | 1 X-linked | 1 Mild  1 No MIS-C  AND  1 *Epstein-Barr virus* coinfection  1 Refractory IBD  1 Hypogammaglobulinemia  1 Post HSCT  1 Graft versus host disease  1 Sinusoidal obstructive syndrome | 1 Low RBCs  1 Low WBCs  1 Thrombocytopenia | 1 IVIG  1 Steroids | 0 | 0 | 0 | (Modified NOS, high)  1 survived |
| Lee et al. 2020 ([9](#_ENREF_9)), United States | Retrospective case report, single centre | 204 | 1 (100) | 1 White (Caucasian) | 1 Autoimmunity with or without lymphoproliferation (SOCS1 deficiency) | SOCS1 deficiency (n=1) | 1 NOPMiIrPwD | 1 AD | 1 Severe  1 No MIS-C  AND  1 AIHA  1 Hypogammaglobulinemia  1 ITP  1 Coagulopathy  1 Metabolic acidosis | 1 Low Hb  1 Thrombocytopenia  1 Neutropenia  1 Lymphopenia  1 Leukopenia  1 High LDH  1 High CRP  1 Raised procalcitonin  1 Elevated PT  1 Reduced number of CD3+, CD4+ and CD8+ T cells  1 Low serum IgA and IgG levels  1 Increased lactate | 1 Steroids  1 IVIG  1 Fresh frozen plasma  1 Platelet transfusions | 0 | 0 | 0 | (Modified NOS, high)  1 survived |
| Michniacki et al. 2022 ([96](#_ENREF_96)), United States | Retrospective case report, single centre | 60 | 0 (0) | 1 Indian | 1 Autoimmunity with or without lymphoproliferation (SOCS1 deficiency) | SOCS1 deficiency (n=1) | 5 mega-base chromosomal 16p deletion at 16p13.2p13.11 (n=1) | 1 AD | 1 Mild  1 No MIS-C  AND  1 Arthralgias  1 Arthritis  1 Enthesitis  1 Ambulatory difficulties  1 Limited movement  1 Pain  1 Bony overgrowth  1 Synovial thickening  1 Multiple small joints of hands and metatarsophalangeal joints  1 Abnormal gait  1 Lumbar lordosis  1 Wet purpura  1 Epistaxis  1 Haematuria  1 Melena | 1 Thrombocytopenia  1 Excessive B cell activity  1 Eosinophilia  1 Elevated IgE levels  1 Eosinophilia  1 High aldolase  1 Increased IgE levels  1 High NK cells  1 Lymphocytosis | 1 Tofacitinib  1 Steroids  1 IVIG  1 Rituximab  1 Romiplostim | 0 | 0 | 0 | (Modified NOS, high)  1 survived |
| Angelino et al. 2021 ([32](#_ENREF_32)), Italy | Retrospective case report, single centre | 204 | 1 (100) | 1 White (Caucasian) | 1 Regulatory T cell defects (CTLA4 deficiency) | CTLA4 deficiency (n=1) | c.118G>A, p.V40M (n=1) | 1 AD | 1 Asymptomatic  1 No MIS-C  AND  1 IBD  1 Diabetes mellitus type 1  1 Polyarthritis  1 Psoriasis  1 Gastric mass  1 Subtotal gastrectomy  1 Termino-lateral gastro-jejunal anastomosis | 1 Lymphopenia  1 IgA deficiency  1 Reduced CD19+ B cells  1 Decreased memory B cells  1 Increased CD21^low^ CD38^low^ B cells | 1 Steroids  1 Abatacept  1 Mesalazine  1 Lansoprazole  1 Multivitamin  1 Iron | 0 | 0 | 0 | (Modified NOS, high)  1 survived |
| Meyts et al. 2021 ([95](#_ENREF_95)), 10 countries | Retrospective cohort, multi-centre | Age groups:156-216 (n=1) | 0 (0) | 1 White (Caucasian) | 1 Regulatory T cell defects (CTLA4 deficiency) | CTLA4 deficiency (n=1) | 1 NOPMiIrPwD | 1 AD | 1 Moderate  1 No MIS-C  AND  1 Post HSCT  1 Poor graft function  1 CLD | 1 Anaemia  1 Neutropenia | 1 Chloroquine  1 Lopinavir/ritonavir  1 Tocilizumab | 0 | 0 | 0 | (NOS, 8)  1 survived |
| Karimi et al. 2021 ([74](#_ENREF_74)), Iran | Retrospective cohort, multi- centre | 216 | 0 (0) | 1 Persian | 1 Immune dysregulation with colitis (IL-10Ra deficiency) | IL10RA deficiency (n=1) | 1 NOPMiIrPwD | 1 AR | 1 Asymptomatic  1 No MIS-C  AND  1 Early-onset IBD | 1 Not possible to extract | 1 Antibiotics  1 Steroids | 0 | 0 | 0 | (NOS, 6)  1 survived |
| Vagrecha et a. 2022 ([125](#_ENREF_125)), United States | Retrospective cohort, single centre | 168 | 0 (0) | 1 Black | 1 Immune dysregulation with colitis (IL-10Ra deficiency) | IL10RA deficiency (n=1) | c.700A>G (n=1) | 1 AR | 1 Not reported  1 Yes MIS-C  AND  1 No comorbidities | 1 Not possible to extract | 1 Not possible to extract | 1 Not possible to extract | 1 Not possible to extract | 1 Not possible to extract | (NOS, 7)  1 outcome was not reported |
| Abolhassani et al. 2022 ([19](#_ENREF_19)), Iran | Prospective cohort, single centre | 180 and 192 | 1 (50) | 2 Persians | 2 Regulatory T cell defects (BACH2 deficiencies) | BACH2 (n=2) | IFN pathway genes: IFNA4, IFNA21 and DNASE2B (n=1)  IL-1 activation pathway genes: NLRP1, NLRP2, IL1RL1, and MEFV (n=1) | 2 ADs | 2 Severe  2 No MIS-C  AND  2 Not reported | 1 Low serum IgA and IgG levels  1 Low serum IgA, IgM and IgG levels | 2 Antibiotics  1 IVIG  1 Steroids  1 Convalescent plasma  2 TPN | 2 | 0 | 0 | (NOS, 8)  2 survived |
| Karakoc Aydiner et al. 2022 ([73](#_ENREF_73)), Turkey | Prospective cohort, multi-centre | 61 and 108 | 2 (100) | 2 Whites (Caucasians) | 2 Susceptibilities to *Epstein–Barr virus* and lymphoproliferative conditions (RLTPR deficiencies) | CARMIL2 (n=2) | 2 NOPMiIrPwD | 2 ARs | 1 Asymptomatic  1 Mild  2 No MIS-C  AND  2 Not reported | 1 High LDH  2 Hypoalbuminemia | 2 IVIG  2 Antibiotics | 1 | 1 | 1 | (NOS, 7)  1 survived  1 died (not COVID-19-related) |
| Abolhassani et al. 2022 ([19](#_ENREF_19)), Iran | Prospective cohort, single centre | 192 | 1 (100) | 1 Persian | 1 Regulatory T cell defects (NOTCH1 mutation) | NOTCH1 (n=1) | Other related genes: APOL1 (n=1), IL17RC (n=1), and IL23R (n=1) | 1 AR | 1 Severe  1 No MIS-C  AND  1 Not reported | 1 Low serum IgA, IgM and IgG levels | 1 Antibiotics  1 Steroids  1 Biological agents  1 TPN | 1 | 0 | 0 | (NOS, 8)  1 survived |
| Abolhassani et al. 2022 ([19](#_ENREF_19)), Iran | Prospective cohort, single centre | 156 | 0 (0) | 1 Persian | 1 Autoimmune Lymphoproliferative Syndrome (ALPS-Caspase10) | CASP10 (n=1) | Lymphocyte development/epigenetic: SRP72 (n=1)  IFN pathway genes: IFNAR2 (n=1) | 1 AR | 1 Severe  1 No MIS-C  AND  1 Not reported | 1 Low serum IgA, IgM and IgG levels | 1 Antibiotics  1 Steroids  1 Biological agents  1 TPN | 1 | 0 | 0 | (NOS, 8)  1 survived |
| Karakoc Aydiner et al. 2022 ([73](#_ENREF_73)), Turkey | Prospective cohort, multi-centre | 180 | 1 (100) | 1 White (Caucasian) | 1 Susceptibility to *Epstein–Barr virus* and lymphoproliferative conditions (CD137 deficiency) | TNFRSF9 (n=1) | 1 NOPMiIrPwD | 1 AR | 1 Moderate  1 Yes MIS-C  AND  1 HLH  1 Lymphoma | 1 Leukopenia  1 High LDH  1 High CRP  1 High D-dimer  1 Raised procalcitonin  1 High ferritin  1 High ESR  1 High fibrinogen  1 High interleukin-6 | 1 IVIG  1 Antibiotics | 1 | 1 | 1 | (NOS, 7)  1 died (COVID-19-related) |
| Abolhassani et al. 2022 ([19](#_ENREF_19)), Iran | Prospective cohort, single centre | 120 | 1 (100) | 1 Persian | 1 IL37 deficiency (subcategory was not reported) | IL37 (n=1) | Lymphocyte development/epigenetic: ERCC6L2 (n=1)  IL-1 activation pathway genes: PEPD, PRKCD and MVK (n=1) | 1 AR | 1 Critical  1 No MIS-C  AND  1 Not reported | 1 Not reported | 1 Antibiotics  1 Steroids  1 Antiplatelets  1 ACEIs  1 Amiodarone  1 TPN  1 CPR | 1 | 1 | 1 | (NOS, 8)  1 died (not reported if COVID-19-related) |
| Marrero et al. 2021 ([93](#_ENREF_93)), United States | Retrospective case report, single centre | 96 | 1 (100) | 1 Black | 1 Regulatory T cell defect (IPEX syndrome) | 1 NSGDwR | 1 NOPMiIrPwD | 1 X-linked | 1 Severe  1 No MIS-C  AND  1 *Staphylococcus epidermidis* coinfection  1 *Candida parapsilosis* coinfection  1 BK coinfection  1 *Cytomegalovirus* coinfection  1 Graft rejection after HSCT  1 Post HSCT  1 Hypotension  1 Multi-organ failure | 1 Raised procalcitonin  1 High ferritin  1 High CRP  1 High LDH  1 High D-dimer | 1 Remdesivir  1 Tocilizumab 1 Convalescent plasma  1 Nitric oxide | 1 | 1 | 1 | (Modified NOS, high)  1 died (not COVID-19-related) |
| Gelzo et al. 2022 ([62](#_ENREF_62)), Italy | Retrospective cohort, single centre | 84 | 1 (100) | 1 White (Caucasian) | 1 Autoimmunity with or without lymphoproliferation (Prolidase deficiency) | PEPD (n=1) | c.1003C>T, p (n=1) | 1 AR | 1 Not reported  1 Yes MIS-C  AND  1 No comorbidities | 1 Not reported | 1 Not reported | 1 Not reported | 1 Not reported | 1 Not reported | (NOS, 7)  1 outcome was not reported |
| Meyts et al. 2021 ([95](#_ENREF_95)), 10 countries | Retrospective cohort, multi-centre | Age groups:36-144 (n=1) | 1 (100) | 1 Hispanic | 1 Susceptibility to *Epstein–Barr virus* and lymphoproliferative conditions (PRKCD deficiency) | PRKCD deficiency (n=1) | 1 NOPMiIrPwD | 1 AR | 1 Mild  1 No MIS-C  AND  1 Autoimmunity  1 *Rhinovirus* coinfection | 1 Not reported | 1 Steroids  1 Chloroquine | 0 | 0 | 0 | (NOS, 8)  1 survived |
| Mohanty et al. 2021 ([99](#_ENREF_99)), India | Retrospective case report, single centre | 102 | 1 (100) | 1 Indian | 1 Susceptibility to *Epstein–Barr virus* and lymphoproliferative conditions (MAGT1 deficiency) | MAGT1 (n=1) | c.750del (n=1) | 1 X-linked | 1 Asymptomatic  1 No MIS-C  AND  1 No comorbidities | 1 Not reported | 1 No treatment | 0 | 0 | 0 | (Modified NOS, high)  1 survived |
| Castano-Jaramillo et al. 2021 ([40](#_ENREF_40)), Mexico | Retrospective cohort, multi-centre | 22 | 1 (100) | 1 Hispanic | 1 Immune dysregulatory disease (type was not reported) | 1 NSGDwR | 1 NOPMiIrPwD | 1 Unknown | 1 Moderate  1 No MIS-C  AND  1 *Cytomegalovirus* coinfection  1 AIHA  1 Eczema  1 Mild pericardial effusion  1 Left coronary artery dilation | 1 Suspected cow milk allergy | 1 IVIG  1 Steroids | 0 | 0 | 0 | (NOS, 8)  1 survived |
| Conti et al. 2022 ([46](#_ENREF_46)), Italy | Retrospective cohort, single centre | 48 | 1 (100) | 1 White (Caucasian) | 1 Immune dysregulatory disease (type was not reported) | 1 NSGDwR | 1 NOPMiIrPwD | 1 Unknown | 1 Moderate  1 No MIS-C  AND  1 Haemolytic uremic syndrome  1 Coagulopathy  1 Acute renal failure  1 Cerebral oedema | 1 Not reported | 1 SCIg  1 Casirivimab/imdevimab | 1 | 1 | 1 | (NOS, 8)  1 died (not COVID-19-related) |
| Esenboga et al. 2021 ([52](#_ENREF_52)), Turkey | Retrospective cohort, single centre | 156 | 0 (0) | 1 White (Caucasian) | 1 Immune dysregulatory disease (type was not reported) | 1 NSGDwR | 1 NOPMiIrPwD | 1 Unknown | 1 Mild  1 No MIS-C  AND  1 Castleman disease  1 Myasthenia gravis  1 Paraneoplastic pemphigus  1 Systemic lupus erythematosus | 1 Not reported | 1 IVIG  1 Hydroxychloroquine  1 Pyridostigmine  1 Sirolimus | 0 | 0 | 0 | (NOS, 7)  1 survived |
| Giardino et al. 2022 ([63](#_ENREF_63)), Italy | Retrospective cohort, multi-centre | 27 and 156 | 2 (100) | 2 Whites (Caucasians) | 2 Immune dysregulatory disease (types were not reported) | 2 NSGDwR | 2 NOPMiIrPwD | 2 Unknowns | 1 Asymptomatic  1 Mild  2 No MIS-C  AND  1 Diabetes mellitus type 1  1 Early-onset IBD  1 Dilated left ventricle  1 Cognitive disability  1 Bone anomalies  1 Microcephaly  1 Myopia | 1 Hypercholesterolemia 1 Not reported | 2 No treatment | 0 | 0 | 0 | (NOS, 7)  2 survived |
| **IUIS IEIs category: Phagocytic diseases (Group V)** | | | | | | | | | | | | | | | |
| Castano-Jaramillo et al. 2021 ([40](#_ENREF_40)), Mexico | Retrospective cohort, multi-centre | 192, 96, 132 and 192 | 3 (75) | 4 Hispanics | 4 Defects of respiratory burst (CGD gp91phox  1 CGD p67phox) | CYBB (n=3)  NFC2 (n=1) | 4 NOPMiIrPwD | 4 X-linked | 2 Mild  1 Moderate  3 No MIS-C  1 Yes MIS-C  AND  1 Tuberculosis  1 Hypotension  1 Septic and cardiogenic shock  1 Myocarditis  1 DIC  1 Multi-organ failure  1 Hepatic failure  1 renal failure  1 Third grade atrioventricular block | 1 DIC  1 Thrombocytopenia  1 Third grade atrioventricular block  1 Renal and hepatic failure | 2 IVIG  1 Steroids | 1 | 1 | 1 | (NOS, 8)  3 survived  1 died (not COVID-19-related) |
| Chou et al. 2021 ([43](#_ENREF_43)), United States | Prospective cohort, single centre | 192 | 1 (100) | 1 White (Caucasian) | 1 Defect of respiratory burst (CGD gp91phox) | CYBB (n=1) | 1 NOPMiIrPwD | 1 X-linked | 1 Mild  1 No MIS-C  AND  1 Haematochezia  1 Crohn's disease  1 IBD  1 Duodenal vasculitis  1 Coagulopathy  1 Compensated shock | 1 Neutrophilia  1 Lymphopenia  1 High CRP  1 Raised procalcitonin | 1 IVIG  1 Antibiotics  1 Steroids  1 Vitamin K  1 Bowel rest  1 Infliximab | 0 | 0 | 0 | (NOS, 7)  1 survived |
| Conti et al. 2022 ([46](#_ENREF_46)), Italy | Retrospective cohort, single centre | 96 | 1 (100) | 1 White (Caucasian) | 1 Defect of respiratory burst (CGD gp91phox) | CYBB (n=1) | 1 NOPMiIrPwD | 1 X-linked | 1 Mild  1 No MIS-C  AND  1 No comorbidities | 1 Not reported | 1 Antibiotics  1 Antifungals | 0 | 0 | 0 | (NOS, 8)  1 survived |
| Delavari et al. 2021 ([48](#_ENREF_48)), Iran | Prospective cohort, multi-centre | 108 | 0 (0) | 1 Persian | 1 Defect of respiratory burst (CGD gp91phox) | 1 NSGDwR | 1 NOPMiIrPwD | 1 X-linked | 1 Mild  1 No MIS-C  AND  1 No comorbidities | 1 Low Hb  1 Thrombocytosis  1 High ESR | 1 Antibiotics  1 Oxygen supplementation | 0 | 0 | 0 | (NOS, 7)  1 survived |
| Delavari et al. 2021 ([48](#_ENREF_48)), Iran | Prospective cohort, multi-centre | 216 | 1 (100) | 1 Persian | 1 Defect of respiratory burst (CGD p22phox) | CYBA (n=1) | 1 NOPMiIrPwD | 1 X-linked | 1 Moderate  1 Yes MIS-C  AND  1 Pulmonary granulomatosis lesion  1 Severe pulmonary fibrosis | 1 Low Hb  1 High ESR | 1 Antibiotics  1 Hydroxychloroquine | 0 | 0 | 0 | (NOS, 7)  1 survived |
| Esmaeilzadeh et al. 2022 ([53](#_ENREF_53)), Iran | Retrospective case report, single centre | 22 and 156 | 1 (50) | 2 Persians | 2 Defects of respiratory burst [CGD (types were not reported)] | 2 NSGDwR | 2 NOPMiIrPwD | 2 ARs | 2 Severe  1 No MIS-C  1 Yes MIS-C  AND  2 No comorbidities | 2 Anaemia  2 Elevated PT  1 Elevated PTT  1 Decreased creatinine  1 Increased serum calcium  1 High LDH  1 Low Hb  1 Leukocytosis  1 Thrombocytosis  1 High fibrinogen  1 High CRP  1 High LDH  1 High ferritin | 2 Antibiotics  2 Antifungals  1 IVIG  1 Steroids  2 Oxygen supplementation | 0 | 0 | 2 | (Modified NOS, moderate)  2 survived |
| Gelzo et al. 2022 ([62](#_ENREF_62)), Italy | Retrospective cohort, single centre | 12 and 72 | 1 (50) | 2 Whites (Caucasians) | 2 Defects of respiratory burst (CGD p47phox) | NCF1 (n=2) | c.20G>A (n=1)  c.247G>A (n=1) | 2 ARs | 2 Not reported  2 Yes MIS-C  AND  2 No comorbidities | 2 Not reported | 2 Not reported | 2 Not reported | 2 Not reported | 2 Not reported | (NOS, 7)  2 outcome was not reported |
| [Goudouris](https://scholar.google.com/citations?user=nyuqBh4AAAAJ&hl=en&oi=sra) et al. 2021 ([65](#_ENREF_65)), Brazil | Retrospective cohort, multi-centre | 20, 126 and 18 | 3 (100) | 3 Hispanics | 3 Defects of respiratory burst [(CGD (types were not reported)] | 3 NSGDwR | 3 NOPMiIrPwD | 3 ARs | 1 Asymptomatic  1 Mild  1 Moderate  3 No MIS-C  AND  1 Bacterial pneumonia  2 No comorbidities | 3 Not reported | 3 No treatment | 0 | 0 | 0 | (NOS, 8)  3 survived |
| Karimi et al. 2021 ([74](#_ENREF_74)), Iran | Retrospective cohort, multi- centre | 103 | 1 (100) | 1 Persian | 1 Defect of respiratory burst [(CGD (type was not reported)] | 1 NSGDwR | 1 NOPMiIrPwD | 1 AR | 1 Moderate  1 No MIS-C  AND  1 Liver disorder  1 Ear nose and throat disorder  1 CLD | 1 Not possible to extract | 1 Antibiotics  1 Antifungals  1 Steroids  1 Monoclonal antibody | 0 | 0 | 0 | (NOS, 6)  1 survived |
| Kołtan et al. 2022 ([81](#_ENREF_81)), Poland | Retrospective cohort, multi-centre | 1 Not possible to extract | 1 Not possible to extract | 1 White (Caucasian) | 1 Defect of respiratory burst [(CGD (type was not reported)] | 1 NSGDwR | 1 NOPMiIrPwD | 1 AR | 1 Mild  1 No MIS-C  AND  1 Not possible to extract | 1 Not reported | 1 Not possible to extract | 0 | 0 | 0 | (NOS, 7)  1 survived |
| Mantravadi et al. 2021 ([90](#_ENREF_90)), United States | Retrospective case report, single centre | 12 | 1 (100) | 1 Black | 1 Defect of respiratory burst (CGD gp91phox) | CYBB (n=1) | 1 NOPMiIrPwD | 1 X-linked | 1 Mild  1 No MIS-C  AND  1 Bacterial pneumonia  1 Tachycardia  1 HSCT | 1 Raised liver enzymes  1 Lymphopenia  1 Elevated B cell numbers  1 Raised IgG level  1 High ESR  1 High CRP  1 High WBCs  1 Neutrophilia  1 High LDH  1 High creatine kinase  1 High fibrinogen  1 High D-dimer  1 Hyponatremia | 1 Antibiotics  1 IV fluids  1 Analgesics | 1 | 0 | 0 | (Modified NOS, high)  1 survived |
| Marcus et al. 2021 ([92](#_ENREF_92)), Israel | Retrospective cohort, multi-centre | 180 | 1 (100) | 1 Jewish | 1 Defect of respiratory burst (CGD gp91phox) | CYBB (n=1) | 1 NOPMiIrPwD | 1 X-linked | 1 Asymptomatic  1 No MIS-C  AND  1 No comorbidities | 1 Not reported | 1 Antibiotics  1 Antifungals | 0 | 0 | 0 | (NOS, 7)  1 survived |
| Marcus et al. 2021 ([92](#_ENREF_92)), Israel | Retrospective cohort, multi-centre | 192 | 1 (100) | 1 Jewish | 1 Defect of respiratory burst (CGD p47phox) | NCF1 (n=1) | 1 NOPMiIrPwD | 1 AR | 1 Mild  1 No MIS-C  AND  1 ITP  1 Perianal abscess  1 Short stature | 1 Not reported | 1 Antibiotics  1 Antifungals | 0 | 0 | 0 | (NOS, 7)  1 survived |
| Meyts et al. 2021 ([95](#_ENREF_95)), 10 countries | Retrospective cohort, multi-centre | Age groups: 0-24 (n=1) and 36-144 (n=2) | 2 (33.3) | 3 Whites (Caucasians) | 3 Defects of respiratory burst [(CGD (types were not reported)] | CYBB (n=2)  NCF2 (n=1) | 3 NOPMiIrPwD | 2 X-linked  1 Autosomal recessive | 1 Asymptomatic  2 Mild  3 No MIS-C  AND  1 *Burkholderia* coinfection  1 Hyporegenerative anaemia  1 HLH  1 CLD  1 Sepsis | 3 Not reported | 2 Antibiotics  1 Steroids  1 ECMO | 1 | 1 | 1 | (NOS, 8)  2 survived  1 died (not COVID-19-related) |
| Moazzen et al. 2021 ([98](#_ENREF_98)), Iran | Retrospective cohort, single centre | 1 | 1 (100) | 1 Persian | 1 Defect of respiratory burst [(CGD (type was not reported)] | 1 NSGDwR | 1 NOPMiIrPwD | 1 Unknown | 1 Asymptomatic  1 No MIS-C  AND  1 Poor feeding  1 Irritability  1 Abdominal distention  1 Abscess formations (liver) | 1 Not reported | 1 Antibiotics  1 Acyclovir  1 Interferon gamma | 0 | 0 | 0 | (NOS, 6)  1 survived |
| Topal et al. 2022 ([124](#_ENREF_124)), Turkey | Retrospective cohort, single centre | 192 and 36 | 2 Genders were not reported | 2 Whites (Caucasians) | 2 Defects of respiratory burst [(CGD (types were not reported)] | 2 NSGDwR | 2 NOPMiIrPwD | 2 ARs | 1 Mild  1 Asymptomatic  2 No MIS-C  AND  1 Chronic ITP  1 Multiple liver abscess | 2 Not reported | 2 No treatment | 0 | 0 | 0 | (NOS, 7)  2 survived |
| Galletta et al. 2022 ([61](#_ENREF_61)), United States | Retrospective cohort, multi-centre | 192, 204, 12, 24, 60 and 144 | 6 Genders were not reported | 6 Whites (Caucasians) | 6 Congenital neutropenias (Shwachman-Diamond syndromes) | SDBS deficiency (n=4)  2 NSGDwR | 6 NOPMiIrPwD | 6 ARs | 4 Mild  2 Moderate  6 No MIS-C  AND  6 No comorbidities | 6 Not reported | 1 Steroids | 0 | 0 | 0 | (NOS, 7)  6 survived |
| Meyts et al. 2021 ([95](#_ENREF_95)), 10 countries | Retrospective cohort, multi-centre | Age groups:36-144 (n=1) | 1 (100) | 1 White (Caucasian) | 1 Congenital neutropenia (Shwachman-Diamond syndrome) | DNAJC21 deficiency (n=1) | 1 NOPMiIrPwD | 1 AR | 1 Mild  1 No MIS-C  AND  1 Exocrine pancreas insufficiency  1 Bone anomalies  1 Cognitive disability | 1 Cytopenia  1 Anaemia  1 Thrombocytopenia | 1 Antibiotics | 0 | 0 | 0 | (NOS, 8)  1 survived |
| Ouederni et al. 2021 ([101](#_ENREF_101)), Tunisia | Retrospective case report, single centre | 192 | 1 (100) | 1 Arab | 1 Congenital neutropenia (Shwachman-Diamond syndrome) | SDBS deficiency (n=1) | 1 NOPMiIrPwD | 1 AR | 1 Mild  1 No MIS-C  AND  1 Myelodysplasia  1 Lung transplantation  1 Post HSCT  1 Graft versus host disease | 1 Lymphopenia  1 Low NK cells  1 Low CD19, CD3, CD4, and CD8 cells | 1 Antibiotics  1 Antifungals  1 Acyclovir | 0 | 0 | 0 | (Modified NOS, high)  1 survived |
| Karimi et al. 2021 ([74](#_ENREF_74)), Iran | Retrospective cohort, multi- centre | 32 and 84 | 0 (0) | 2 Persians | 2 Congenital neutropenias (HAX1 deficiencies) | HAX1 deficiency (Kostmann Disease) (n=2) | 2 NOPMiIrPwD | 2 ARs | 1 Mild  1 Moderate  2 Not reported  AND  1 No comorbidities  1 Gaucher disease | 2 Not possible to extract | 2 Antibiotics  1 Antifungals | 0 | 0 | 0 | (NOS, 6)  2 survived |
| Khalili et al. 2021 ([77](#_ENREF_77)), Iran | Retrospective case report, single centre | 32 | 0 (0) | 1 Persian | 1 Congenital neutropenia (HAX1 deficiency) | HAX1 deficiency (Kostmann Disease) (n=1) | c.661delG p.1222 (n=1) | 1 AR | 1 Mild  1 No MIS-C  AND  1 Gaucher disease | 1 Lymphopenia  1 Neutropenia  1 High CRP  1 High ESR | 1 Enzyme replacement therapy  1 Antibiotics  1 Granulocyte colony-stimulating factor  1 Hydroxychloroquine | 0 | 0 | 0 | (Modified NOS, high)  1 survived |
| Topal et al. 2022 ([124](#_ENREF_124)), Turkey | Retrospective cohort, single centre | 24, 168 and 204 | 3 Genders were not reported | 3 Whites (Caucasians) | 3 Congenital neutropenias (HAX1 deficiencies) | HAX1 deficiency (Kostmann Diseases) (n=3) | c.130-131insA (n=3) | 3 ARs | 2 Mild  1 Asymptomatic  3 No MIS-C  AND  3 No comorbidities | 3 Not reported | 1 Antibiotics  1 Favipiravir  1 Acyclovir  1 No treatment | 0 | 0 | 0 | (NOS, 7)  3 survived |
| Gelzo et al. 2022 ([62](#_ENREF_62)), Italy | Retrospective cohort, single centre | 180 and 60 | 2 (100) | 2 Whites (Caucasians) | 2 Congenital neutropenias (Glycogen storage diseases type 1b) | SLC37A4 (n=2) | c.882dup, p (n=1)  c.899G>A, p (n=1) | 2 ARs | 2 Not reported  2 Yes MIS-C  AND  2 No comorbidities | 2 Not reported | 2 Not reported | 2 Not reported | 2 Not reported | 2 Not reported | (NOS, 7)  2 outcome was not reported |
| Kołtan et al. 2022 ([81](#_ENREF_81)), Poland | Retrospective cohort, multi-centre | 1 Not possible to extract | 1 Not possible to extract | 1 White (Caucasian) | 1 Congenital neutropenia (Elastase deficiency) | ELANE (n=1) | 1 NOPMiIrPwD | 1 AD | 1 Moderate  1 No MIS-C  AND  1 Not possible to extract | 1 Not reported | 1 Not possible to extract | 0 | 0 | 0 | (NOS, 7)  1 survived |
| Vignesh et al. 2021 ([127](#_ENREF_127)), India | Retrospective case report, single centre | 20 | 1 (100) | 1 Indian | 1 Congenital neutropenia (Elastase deficiency) | ELANE (n=1) | c.457G>C (n=1) | 1 AD | 1 Moderate  1 No MIS-C  AND  1 *Pseudomonas aeruginosa* coinfection  1 Ulcers | 1 Neutropenia  1 Monocytosis  1 Eosinophilia  1 Hypergammaglobulinemia | 1 Antibiotics  1 Oxygen supplementation  1 Granulocyte colony-stimulating factor  1 Surgical debridement for ulcers | 0 | 0 | 0 | (Modified NOS, high)  1 survived |
| Sherkat et al. 2021 ([117](#_ENREF_117)), Iran | Retrospective cohort, single centre | 216 | 0 (0) | 1 Persian | 1 Congenital neutropenia (JAGN1 deficiency) | JAGN1 deficiency (n=1) | 1 NOPMiIrPwD | 1 AR | 1 Moderate  1 No MIS-C  AND  1 No comorbidities | 1 Not reported | 1 Hydroxychloroquine  1 Antibiotics  1 Famotidine  1 Promethazine | 0 | 0 | 0 | (NOS, 7)  1 survived |
| Piccolo et al. 2021 ([107](#_ENREF_107)), Italy | Retrospective case report, single centre | 33 | 1 (100) | 1 White (Caucasian) | 1 Congenital neutropenia (Poikiloderma with neutropenia) | USB1 (n=1) | p.Trp81Ter (n=1) | 1 AR | 1 Mild  1 No MIS-C  AND  1 Cutaneous mastocytosis  1 Dermatitis  1 Poikiloderma  1 Motor developmental delay | 1 Neutropenia  1 High ferritin  1 High CRP  1 Neutrophilia | 1 Antibiotics | 0 | 0 | 0 | (Modified NOS, high)  1 survived |
| Abolhassani et al. 2022 ([19](#_ENREF_19)), Iran | Prospective cohort, single centre | 180 | 1 (100) | 1 Persian | 1 Defect of motility (Cystic fibrosis) | CFTR (n=1) | Lymphocyte development/epigenetic: KMT2D (n=1)  IL-1 activation pathway genes: AP1S3 (n=1) | 1 AR | 1 Critical  1 No MIS-C  AND  1 Not reported | 1 Low serum IgA and IgM levels | 1 Antibiotics  1 IVIG  1 Antiplatelets  1 ACEIs  1 Convalescent plasma  1 Amiodarone  1 TPN  1 CPR | 1 | 1 | 1 | (NOS, 8)  1 died (not reported if COVID-19-related) |
| [Goudouris](https://scholar.google.com/citations?user=nyuqBh4AAAAJ&hl=en&oi=sra) et al. 2021 ([65](#_ENREF_65)), Brazil | Retrospective cohort, multi-centre | 145 | 0 (0) | 1 Hispanic | 1 Defect of motility (Leukocyte adhesion deficiency type 3) | FERMT3 (n=1) | 1 NOPMiIrPwD | 1 AR | 1 Mild  1 No MIS-C  AND  1 No comorbidities | 1 Not reported | 1 No treatment | 0 | 0 | 0 | (NOS, 8)  1 survived |
| Meyts et al. 2021 ([95](#_ENREF_95)), 10 countries | Retrospective cohort, multi-centre | Age groups:156-216 (n=1) | 0 (0) | 1 Hispanic | 1 Other non-lymphoid defects (GATA2 deficiency) | GATA2 (n=1) | 1 NOPMiIrPwD | 1 AD | 1 Asymptomatic  1 No MIS-C  AND  1 Bone marrow hypoplasia  1 Lower limbs oedema  1 Hypotension  1 CLD | 1 Low WBCs  1 Low red blood cells  1 Thrombocytopenia | 1 Antibiotics  1 Steroids  1 IVIG | 0 | 0 | 0 | (NOS, 8)  1 survived |
| Conti et al. 2022 ([46](#_ENREF_46)), Italy | Retrospective cohort, single centre | 168 | 0 (0) | 1 White (Caucasian) | 1 Undefined leukopenia (type was not reported) | 1 NSGDwR | 1 NOPMiIrPwD | 1 Unknown | 1 Mild  1 No MIS-C  AND  1 No comorbidities | 1 Not reported | 1 Sotrovimab  1 Casirivimab/imdevimab | 0 | 0 | 0 | (NOS, 8)  1 survived |
| Esenboga et al. 2021 ([52](#_ENREF_52)), Turkey | Retrospective cohort, single centre | 15 | 1 (100) | 1 White (Caucasian) | 1 Congenital neutropenia (type was not reported) | 1 NSGDwR | 1 NOPMiIrPwD | 1 Unknown | 1 Mild  1 No MIS-C  AND  1 No comorbidities | 1 Not reported | 1 Granulocyte colony-stimulating factor  1 Favipiravir 1 Antibiotics | 0 | 0 | 0 | (NOS, 7)  1 survived |
| Giardino et al. 2022 ([63](#_ENREF_63)), Italy | Retrospective cohort, multi-centre | 18 | 0 (0) | 1 White (Caucasian) | 1 Congenital neutropenia (type was not reported) | 1 NSGDwR | 1 NOPMiIrPwD | 1 Unknown | 1 Mild  1 No MIS-C  AND  1 Pulmonary valve stenosis  1 Interatrial communication with left to right shunt  1 Corpus callosum hypoplasia  1 Left kidney with double district | 1 Not reported | 1 No treatment | 0 | 0 | 0 | (NOS, 7)  1 survived |
| Ouederni et al. 2021 ([101](#_ENREF_101)), Tunisia | Retrospective case report, single centre | 72 | 0 (0) | 1 Arab | 1 Congenital neutropenia (type was not reported) | 1 NSGDwR | 1 NOPMiIrPwD | 1 AD | 1 Mild  1 No MIS-C  AND  1 Post HSCT | 1 Neutropenia  1 Aplasia  1 Lymphopenia | 1 Antibiotics  1 Antifungals  1 Heparin  1 Acyclovir | 0 | 0 | 0 | (Modified NOS, moderate)  1 survived |
| Topal et al. 2022 ([124](#_ENREF_124)), Turkey | Retrospective cohort, single centre | 7 | 1 Gender was not reported | 1 White (Caucasian) | 1 Congenital neutropenia (type was not reported) | 1 NSGDwR | 1 NOPMiIrPwD | 1 Unknown | 1 Mild  1 No MIS-C  AND  1 Atopic dermatitis  1 Previous infantile sepsis  1 Pyoderma | 1 Not reported | 1 Antibiotics | 0 | 0 | 0 | (NOS, 7)  1 survived |
| **IUIS IEIs category: Innate immunodeficiencies (Group VI)** | | | | | | | | | | | | | | | |
| Abolhassani et al. 2022 ([26](#_ENREF_26)), Iran | Retrospective case report, single centre | 84 | 1 (100) | 1 Persian | 1 Predisposition to severe viral infection (TLR7 deficiency) | TLR7 deficiency (n=1) | L372M (n=1) | 1 X-linked | 1 Critical  1 Not reported  AND  1 Type I IFN deficiency  1 Severe seizures  1 Hypertension  1 Dilatation of the aorta  1 Renal failure | 1 Anaemia  1 Lymphopenia  1 Thrombocytopenia  1 Low serum IgA and IgG levels  1 High IgM level  1 High ESR  1 Low RBCs  1 Low Hb  1 Low haematocrit  1 Low mean corpuscular volume  1 High alpha fetoprotein | 1 IVIG  1 TPN  1 Antiplatelets  1 Steroids  1 Convalescent plasma  1 Heparin | 1 | 1 | 1 | (Modified NOS, high)  1 survived |
| Abolhassani et al. 2022 ([19](#_ENREF_19)), Iran | Prospective cohort, single centre | 96 | 1 (100) | 1 Persian | 1 Predisposition to severe viral infection (TLR7 deficiency) | TLR7 deficiency (n=1) | Lymphocyte development/epigenetic: BCL11B (n=1)  IFN pathway genes: IFNA4, STAT6, IFIH1, and RNASEH2C (n=1) | 1 X-linked | 1 Severe  1 No MIS-C  AND  1 Not reported | 1 Low serum IgA and IgG levels | 1 IVIG  1 Antibiotics  1 TPN  1 Steroids  2 Antiplatelets | 1 | 1 | 1 | (NOS, 8)  1 survived |
| Asano et al. 2021 ([33](#_ENREF_33)), 7 countries | Prospective cohort, multi-centre | 156, 84, 216 and 156 | 4 (100) | 3 Whites (Caucasians)  1 Persian | 4 Predisposition to severe viral infections (TLR7 deficiency) | TLR7 deficiency (n=4) | D244Y, L372M, I657T and H781L (n=1) | 4 X-linked | 4 Severe  4 No MIS-C  AND  4 Type I IFN deficiency  1 Granulomatous lesion  1 Broad‐based gait  1 Obesity  1 Seizure  1 Hypertension  1 Dilatation of aorta  1 Enteropathy  1 Renal failure | 1 Anaemia  1 Thrombocytopenia  1 Low Hb  1 Lymphopenia  2 High CRP | 2 Antibiotics  1 Hydroxychloroquine  1 Favipiravir  1 Chloroquine  1 Anakinra  1 ACEIs  1 Haemodialysis  1 Lung transplantation  3 Oxygen supplementation | 4 | 3 | 3 | (NOS, 7)  4 survived |
| Pessoa et al. 2021 ([106](#_ENREF_106)), Brazil | Retrospective case report, single centre | 60 | 1 (100) | 1 Hispanic | 1 Predisposition to severe viral infection (TLR7 deficiency) | TLR7 deficiency (n=1) | rs 179008 (n=1) | 1 AR | 1 Mild  1 No MIS-C  AND  1 Hepatitis  1 Asthma  1 Odynophagia  1 Abdominal pain  1 Acholic stool  1 Jaundice  1 Tiredness  1 Oedema | 1 Hyperbilirubinemia  1 High CRP  1 High D-dimer  1 Elevated PTT  1 High α1-Antitrypsin | 1 Not reported | 0 | 0 | 0 | (Modified NOS, high)  1 survived |
| Zhang et al. 2022 ([132](#_ENREF_132)), 4 countries | Retrospective cohort, multi-centre | 144 | 1 (100) | 1 White (Caucasian) | 1 Predisposition to severe viral infection (TLR7 deficiency) | TLR7 deficiency (n=1) | I174R/Y (n=1) | 1 X-linked | 1 Severe  1 No MIS-C  AND  1 Type I IFN deficiency | 1 Not reported | 1 Not reported | 0 | 0 | 0 | (NOS, 7)  1 survived |
| Bucciol et al. 2022 ([37](#_ENREF_37)), Belgium | Retrospective case report, single centre | 48 | 1 (100) | 1 White (Caucasian) | 1 TLR signalling pathway deficiency with bacterial susceptibility (MyD88 deficiency) | MYD88 (n=1) | c.196-198del-GGA (n=1) | 1 AR | 1 Severe  1 No MIS-C  AND  1 Skin abscess (chest)  1 Gut perforation 1 Surgical resection  1 Ileostomy | 1 High CRP  1 Neutrophilia  1 Lymphopenia  1 Thrombocytopenia  1 High WBCs (CSF)  1 High RBCs (CSF)  1 Increased protein (CSF)  1 Low glucose (CSF)  1 Iron deficiency  1 Elevated vitamin B12  1 IgG3 subclass deficiency  1 Partial IgA deficiency  1 Inherited congenital asplenia  1 Absence of Howell-Jolly bodies  1 Low monocytes and NK cells  1 Low effector T cells  1 Low T follicular helper cells  1 Low T helper 17 cells | 1 Antibiotics  1 IVIG  1 Oxygen supplementation | 0 | 0 | 0 | (Modified NOS, high)  1 survived |
| Deyà-Martínez et al. 2021 ([50](#_ENREF_50)), Spain | Prospective cohort, single centre | 192 | 0 (0) | 1 White (Caucasian) | 1 TLR signalling pathway deficiency with bacterial susceptibility (MyD88 deficiency) | MYD88 (n=1) | 1 NOPMiIrPwD | 1 AR | 1 Moderate  1 No MIS-C  AND  1 No comorbidities | 1 High ferritin  1 Thrombocytopenia  1 Anaemia  1 Lymphopenia  1 High D-dimer  1 High CRP | 1 Steroids  1 Heparin  1 Antibiotics | 0 | 0 | 0 | (NOS, 7)  1 survived |
| Giardino et al. 2022 ([63](#_ENREF_63)), Italy | Retrospective cohort, multi-centre | 102 | 1 (100) | 1 White (Caucasian) | 1 TLR signalling pathway deficiency with bacterial susceptibility (MyD88 deficiency) | MYD88 (n=1) | 1 NOPMiIrPwD | 1 AR | 1 Asymptomatic  1 No MIS-C  AND  1 Multiple abdominal abscess  1 Right brachio-crural hemiparesis | 1 Not reported | 1 IVIG  1 Antibiotics | 0 | 0 | 0 | (NOS, 7)  1 survived |
| Mahmood et al. 2021 ([89](#_ENREF_89)), United States | Retrospective case-series, single centre | 192, 180 and 156 | 1 (33.3) | 3 Whites (Caucasians) | 3 TLR signalling pathway deficiencies with bacterial susceptibility (MyD88 deficiency) | MYD88 (n=3) | 3 NOPMiIrPwD | 3 ARs | 2 Severe  1 Moderate  3 No MIS-C  AND  3 No comorbidities | 2 Lymphopenia | 3 Hydroxychloroquine  2 Tocilizumab 2 Remdesivir  2 Oxygen supplementation  1 ECMO | 3 | 2 | 2 | (Modified NOS, high)  3 survived |
| Milito et al. 2021 ([97](#_ENREF_97)), Italy | Retrospective cohort, multi-centre | Age group: <216 (n=1) | 1 Not possible to extract | 1 Whites (Caucasians) | 1 TLR signalling pathway deficiency with bacterial susceptibility (MyD88 deficiency) | MYD88 (n=1) | 1 NOPMiIrPwD | 1 AR | 1 Mild  1 No MIS-C  AND  1 Not possible to extract | 1 Not reported | 1 Not reported | 0 | 0 | 0 | (NOS, 7)  1 survived |
| Esenboga et al. 2021 ([52](#_ENREF_52)), Turkey | Retrospective cohort, single centre | 24 and 114 | 1 (50) | 2 Whites (Caucasians) | 2 Predisposition to mucocutaneous candidiasis (STAT1 deficiencies) | STAT1-GOF (n=1)  1 NSGDwR | 2 NOPMiIrPwD | 2 ADs | 1 Mild  1 No MIS-C  AND  1 AIHA  1 Hepatitis  1 Epilepsy  1 Mycotic calcification in the aortic wall  1 Esophagitis | 2 Lymphopenia  2 Low serum IgM levels  1 Low serum IgA and IgE levels  1 Low memory and switch memory B cells 1 Low naive T cells, increased effector memory T cells | 2 IVIG  1 Antibiotics  1 Mycophenolate mofetil  1 Favipiravir | 0 | 0 | 0 | (NOS, 7)  2 survived |
| [Goudouris](https://scholar.google.com/citations?user=nyuqBh4AAAAJ&hl=en&oi=sra) et al. 2021 ([65](#_ENREF_65)), Brazil | Retrospective cohort, multi-centre | 136 | 1 (100) | 1 Hispanic | 1 Predisposition to mucocutaneous candidiasis (STAT1 deficiency) | STAT1-GOF (n=1) | 1 NOPMiIrPwD | 1 AD | 1 Severe  1 No MIS-C  AND    1 Bronchiectasis  1 Diabetes mellitus type I | 1 Not reported | 1 No treatment | 0 | 0 | 0 | (NOS, 8)  1 survived |
| Guisado Hernández et al. 2021 ([68](#_ENREF_68)), Spain | Retrospective case report, single centre | 132 | 0 (0) | 1 White (Caucasian) | 1 Predisposition to mucocutaneous candidiasis (STAT1 deficiency) | STAT1-GOF (n=1) | c.1053G>T (n=1) | 1 AD | 1 Mild  1 No MIS-C  AND  1 Enteropathy  1 Subarachnoideal haemorrhage  1 Cerebral aneurysm | 1 Low memory B cells  1 Lymphopenia  1 High CRP  1 High interleukin-6 | 1 Ruxolitinib  1 Antibiotics  1 Antifungals  1 IVIG | 0 | 0 | 0 | (Modified NOS, high)  1 survived |
| Karakoc Aydiner et al. 2022 ([73](#_ENREF_73)), Turkey | Prospective cohort, multi-centre | 169 | 1 (100) | 1 White (Caucasian) | 1 Predisposition to severe viral infection (STAT1 deficiency) | STAT1-GOF (n=1) | 1 NOPMiIrPwD | 1 AR | 1 Moderate  1 Yes MIS-C  AND  1 CMC  1 AIHA  1 Immune thrombocytopenia | 1 Neutropenia  1 High CRP  1 High ferritin  1 High LDH  1 High D-dimer  1 High fibrinogen  1 Hypoalbuminemia | 1 IVIG  1 Antibiotics | 0 | 0 | 0 | (NOS, 7)  1 survived |
| Meyts et al. 2021 ([95](#_ENREF_95)), 10 countries | Retrospective cohort, multi-centre | Age groups:36-144 (n=1) | 0 (0) | 1 Hispanic | 1 Predisposition to mucocutaneous candidiasis (STAT1 deficiency) | STAT1-GOF (n=1) | 1 NOPMiIrPwD | 1 AD | 1 Asymptomatic  1 No MIS-C  AND  1 CLD | 1 Not reported | 1 No treatment | 0 | 0 | 0 | (NOS, 8)  1 survived |
| Vagrecha et a. 2022 ([125](#_ENREF_125)), United States | Retrospective cohort, single centre | 36 | 0 (0) | 1 Black | 1 Predisposition to mucocutaneous candidiasis (STAT1 deficiency) | STAT1-GOF (n=1) | c.1591G>A (n=1) | 1 AD | 1 Not reported  1 Yes MIS-C  AND  1 No comorbidities | 1 Not possible to extract | 1 Not possible to extract | 1 Not possible to extract | 1 Not possible to extract | 1 Not possible to extract | (NOS, 7)  1 outcome was not reported |
| Abolhassani et al. 2022 ([19](#_ENREF_19)), Iran | Prospective cohort, single centre | 36 | 0 (0) | 1 Persian | 1 Predisposition to severe viral infection (IFNAR1 deficiency) | IFNAR1 deficiency (n=1) | Lymphocyte development/epigenetic: KMT2A (n=1)  IFN pathway genes: TYK2 and PDGFRB (n=1)  IL-1 activation pathway genes: NLRC4 (n=1)  NF-kB pathway genes: NOD2 (n=1) | 1 AR | 1 Critical  1 Yes MIS-C  AND  1 Kawasaki disease | 1 Not reported | 1 Antibiotics  1 IVIG  1 ACEIs  1 Biological agents  1 Antiplatelets  1 Amiodarone  1 CPR  1 TPN | 1 | 1 | 1 | (NOS, 8)  1 died (COVID-19-related) |
| Abolhassani et al. 2022 ([25](#_ENREF_25)), Iran | Retrospective case report, single centre | 36 | 0 (0) | 1 Persian | 1 Predisposition to severe viral infection (IFNAR1 deficiency) | IFNAR1 deficiency (n=1) | H263fs (n=1) | 1 AR | 1 Critical  1 Yes MIS-C  AND  1 Anaemia  1 Severe mucormycosis of the nose and paranasal sinuses  1 Myocardial oedema  1 Hypotension  1 Bradycardia  1 Cardiac arrhythmia | 1 Anaemia  1 Leukocytosis  1 Thrombocytosis  1 Raised liver enzymes  1 Increased acute-phase reactants  1 High CRP  1 High ESR  1 High creatine kinase  1 Metabolic acidosis  1 Pyuria | 1 IVIG  1 Antiplatelets  1 Steroid  1 Antibiotics  1 Paracetamol  1 Ibuprofen  1 IV inotropes  1 ACEIs  1 Amiodarone  1 Infliximab | 1 | 1 | 1 | (Modified NOS, high)  1 died (COVID-19-related) |
| Khanmohammadi et al. 2022 ([78](#_ENREF_78)), Iran | Retrospective case report, single centre | 156 | 1 (100) | 1 Persian | 1 Predisposition to severe viral infection (IFNAR1 deficiency) | IFNAR1 deficiency (n=1) | c.674(-2b)A>G (n=1) | 1 AR | 1 Severe  1 No MIS-C  AND  1 Type I IFN deficiency  1 Severe disease caused by MMRV vaccine  1 Shortness of breath  1 Hypotension  1 Bradycardia  1 Papilledema  1 Pseudotumor cerebri | 1 High interleukin-6  1 Elevated PT  1 Elevated PTT  1 High ESR  1 High WBCs  1 Low Hb | 1 Favipiravir  1 IVIG  1 Antibiotics  1 Antifungals  1 IFN-γ  1 Oxygen supplementation  1 Remdesivir  1 Steroids  1 Antiplatelets  1 Famotidine 1 Zinc  1 Vitamin C  1 Atropine  1 Fresh frozen plasma | 1 | 0 | 1 | (Modified NOS, high)  1 survived |
| Duncan et al. 2022 ([51](#_ENREF_51)), 3 countries | Retrospective case report, multi-entre | 32, 84 and 14 | 1 (33.3) | 3 Whites (Caucasians) | 3 Predisposition to severe Viral infections (IFNAR2 deficiencies) | IFNAR2 deficiency (n=3) | c.157T>C (n=3) | 3 ARs | 1 Mild  1 Moderate  1 Severe  3 No MIS-C  AND  3 Type I IFN deficiency  3 Severe diseases caused by MMRV vaccine  1 Seizures  1 Asthma  1 Pneumomediastinum  1 Refractory vasoactive shock  1 Septic shock  1 Progressive neuromuscular disorder  1 Variable crusting (face, chest, abdomen, and all extremities)  1 Weight loss  3 Respiratory failures | 2 Not reported  1 Lymphopenia | 2 Steroids  1 IV inotropes  1 Albumin  2 Antibiotics  1 Acyclovir  1 Remdesivir  1 Oxygen supplementation | 3 | 3 | 3 | (Modified NOS, high)  1 survived  2 died (1 not COVID-19-related) and (1 COVID-19-related) |
| Zhang et al. 2022 ([132](#_ENREF_132)), 4 countries | Retrospective cohort, multi-centre | 24, 48 and 108 | 2 (66.7) | 3 Arabs | 3 Mendelian susceptibility to mycobacterial diseases (TYK2 deficiency) | TYK2 deficiency (n=3) | P216fs (n=3) | 3 ARs | 1 Moderate  2 Critical  3 No MIS-C  AND  1 *Varicella zoster virus* coinfection  1 Severe disease caused by MMRV vaccine  1 Kawasaki disease | 3 Not reported | 3 Not reported | 2 | 0 | 2 | (NOS, 7)  3 survived |
| Schmidt et al. 2021 ([114](#_ENREF_114)), Germany | Retrospective case report, single centre | 42 | 0 (0) | 1 White (Caucasian) | 1 Non-inflammasome related conditions (TBK1 deficiency) | TBK1 deficiency (n=1) | rs753802322 (n=1) | 1 AR | 1 Severe  1 Yes MIS-C  AND  1 Arthritis  1 Anaemia  1 Recurrent episodes of exanthema and systemic inflammation  1 DIC  1 Acute cardiac insufficiency  1 Acute heart failure  1 Hypotension  1 Heart failure  1 Venous infarction  1 Thrombosis  1 Intracranial haemorrhage  1 Aspiration pneumonia  1 DIC  1 Seizures  1 Multi-organ failure | 1 High WBCs  1 Increased lactate  1 Metabolic acidosis  1 Elevated creatinine  1 Raised liver enzymes | 1 Steroids  1 Methotrexate  1 Inhaled nitric oxide  1 Vasopressors  1 IV inotropes  1 Sodium bicarbonate  1 ECMO | 1 | 1 | 1 | (Modified NOS, high)  1 died (COVID-19-related) |
| Zhang et al. 2020 ([10](#_ENREF_10)), 12 countries | Retrospective cohort, multi-centre | 204 | 1 (100) | 1 White (Caucasian) | 1 Non-inflammasome related conditions (TBK1 deficiency) | TBK1 deficiency (n=1) | p.Arg308*/WT (n=1) | 1 AR | 1 Severe  1 No MIS-C  AND  1 Not reported | 1 Not reported | 1 Not reported | 0 | 0 | 0 | (NOS, 7)  1 survived |
| Kołtan et al. 2022 ([81](#_ENREF_81)), Poland | Retrospective cohort, multi-centre | 1 Not possible to extract | 1 Not possible to extract | 1 White (Caucasian) | 1 Other IEIs related to non-hematopoietic tissues (Isolated congenital asplenia) | 1 NSGDwR | 1 NOPMiIrPwD | 1 Unknown | 1 Moderate  1 No MIS-C  AND  1 Not possible to extract | 1 Not reported | 1 Not possible to extract | 0 | 0 | 0 | (NOS, 7)  1 survived |
| Pieniawska-Śmiech et al. 2021 ([108](#_ENREF_108)), Poland | Retrospective cohort, single centre | 96 | 0 (0) | 1 White (Caucasian) | 1 Other IEIs related to non-hematopoietic tissues (Isolated congenital asplenia) | 1 NSGDwR | 1 NOPMiIrPwD | 1 Unknown | 1 Mild  1 No MIS-C  AND  1 No comorbidities | 1 Lymphopenia | 1 No treatment | 0 | 0 | 0 | (NOS, 8)  1 survived |
| Ho et al. 2021 ([71](#_ENREF_71)), United States | Retrospective cohort, single centre | 12 | 1 (100) | 1 White (Caucasian) | 1 Mendelian susceptibility to mycobacterial disease (IFN-g receptor 2 deficiency) | IFNGR2 (n=1) | 1 NOPMiIrPwD | 1 AR | 1 Mild  1 No MIS-C  AND  1 *Miliary Mycobacterium avium* coinfection | 1 Lymphocytosis  1 Neutrophilia | 1 Steroids | 1 | 1 | 1 | (NOS, 7)  1 survived |
| Meyts et al. 2021 ([95](#_ENREF_95)), 10 countries | Retrospective cohort, multi-centre | Age groups:0-24 (n=1) | 1 (100) | 1 White (Caucasian) | 1 Mendelian susceptibility to mycobacterial disease (IFN-g receptor 2 deficiency) | IFNGR2 (n=1) | 1 NOPMiIrPwD | 1 AR | 1 Mild  1 No MIS-C  AND    1 *Miliary Mycobacterium avium* coinfection  1 AIHA | 1 Leukocytosis  1 Not reported | 1 Steroids | 0 | 0 | 0 | (NOS, 8)  1 survived |
| Vagrecha et a. 2022 ([125](#_ENREF_125)), United States | Retrospective cohort, single centre | 60 and 72 | 0 (0) | 1 Black  1 Hispanic | 2 Predisposition to severe viral infections (MDA5 deficiency) | IFIH1 (n=2) | c.1793G>A (n=1)  c.467A>C (n=1) | 2 ARs | 2 Not reported  2 Yes MIS-C  AND  2 No comorbidities | 2 Not possible to extract | 2 Not possible to extract | 2 Not possible to extract | 2 Not possible to extract | 2 Not possible to extract | (NOS, 7)  2 outcome was not reported |
| Abolhassani et al. 2022 ([19](#_ENREF_19)), Iran | Prospective cohort, single centre | 192 | 1 (100) | 1 Persian | 1 *Herpes simplex* encephalitis (TRIF deficiency) | TICAM1 (n=1) | DNA repair gene defect: ATM (n=1)  IFN pathway genes: RNASEH2B (n=1) | 1 AD | 1 Severe  1 No MIS-C  AND  1 Not reported | 1 Not reported | 1 Antibiotics 1 Biological agents  1 Steroids  1 Oxygen supplementation | 0 | 0 | 0 | (NOS, 8)  1 survived |
| [Goudouris](https://scholar.google.com/citations?user=nyuqBh4AAAAJ&hl=en&oi=sra) et al. 2021 ([65](#_ENREF_65)), Brazil | Retrospective cohort, multi-centre | 114 | 0 (0) | 1 Hispanic | 1 Epidermodysplasia verruciformis (Warts, Hypogammaglobulinemia, infections, Myelokathexis syndrome) | CXCR4 (n=1) | 1 NOPMiIrPwD | 1 AD | 1 Asymptomatic  1 No MIS-C  AND  1 No comorbidities | 1 Not reported | 1 No treatment | 0 | 0 | 0 | (NOS, 8)  1 survived |
| [Goudouris](https://scholar.google.com/citations?user=nyuqBh4AAAAJ&hl=en&oi=sra) et al. 2021 ([65](#_ENREF_65)), Brazil | Retrospective cohort, multi-centre | 22 | 0 (0) | 1 Hispanic | 1 TLR signalling pathway deficiency with bacterial susceptibility (IRAK4 deficiency) | IRAK4 deficiency (n=1) | 1 NOPMiIrPwD | 1 AD | 1 Moderate  1 No MIS-C  AND  1 Bacterial pneumonia  1 Atelectasis | 1 Not reported | 1 SCIg | 0 | 0 | 0 | (NOS, 8)  1 survived |
| Lévy et al. 2021 ([87](#_ENREF_87)), France | Retrospective case report, single centre | 96 | 0 (0) | 1 Arab | 1 Predisposition to severe viral infection (IRF9 deficiency) | IRF9 deficiency (n=1) | c.991G>A (n=1) | 1 AR | 1 Mild  1 No MIS-C  AND  1 No comorbidities | 1 High CRP | 1 Casirivimab/imdevimab | 0 | 0 | 0 | (Modified NOS, high)  1 survived |
| Zhang et al. 2022 ([132](#_ENREF_132)), 4 countries | Retrospective cohort, multi-centre | 144 | 1 (100) | 1 Arab | 1 Predisposition to severe viral infection (STAT2 deficiency) | STAT2 deficiency (n=1) | S613F (n=1) | 1 AR | 1 Critical  1 No MIS-C  AND  1 Severe disease caused by MMRV vaccine  1 AIHA  1 Kawasaki disease | 1 Not reported | 1 Not reported | 0 | 0 | 0 | (NOS, 7)  1 survived |
| Allen-Manzur et al. 2020 ([4](#_ENREF_4)), Mexico | Retrospective case report, single centre | 72 | 0 (0) | 1 Hispanic | 1 Mendelian susceptibility to mycobacterial disease (IL-12 and IL-23 receptor b1 chain deficiency) | IL12RB1 (n=1) | IL12RB1 (n=1) | 1 AR | 1 Mild  1 No MIS-C  AND  1 *Mycobacterium bovis* coinfection  1 Adenitis due to tuberculosis vaccine  1 Cutaneous fistula in the anterior thorax  1 Suppurative lymphadenitis | 1 Not reported | 1 Not reported | 0 | 0 | 0 | (Modified NOS, high)  1 survived |
| Abolhassani et al. 2022 ([19](#_ENREF_19)), Iran | Prospective cohort, single centre | 120 | 0 (0) | 1 Persian | 1 Defects in innate immunity (type was not reported) | 1 NSGDwR | Lymphocyte development/epigenetic: KMT2D (n=1)  DNA repair gene defect: ERBB2IP (n=1)  IL-1 activation pathway genes: PSTPIP1 (n=1) | 1 Unknown | 1 Severe  1 No MIS-C  AND  1 Not reported | 1 Low serum IgA, IgM and IgG levels | 1 Antibiotics 1 Biological agents  1 Oxygen supplementation | 0 | 0 | 0 | (NOS, 8)  1 survived |
| [Goudouris](https://scholar.google.com/citations?user=nyuqBh4AAAAJ&hl=en&oi=sra) et al. 2021 ([65](#_ENREF_65)), Brazil | Retrospective cohort, multi-centre | 75 | 0 (0) | 1 Hispanic | 1 Defect in innate immunity (type was not reported) | 1 NSGDwR | 1 NOPMiIrPwD | 1 Unknown | 1 Moderate  1 Yes MIS-C  AND  1 Pleural and pericardial oedema | 1 Not reported | 1 IVIG | 0 | 0 | 0 | (NOS, 8)  1 survived |
| Milito et al. 2021 ([97](#_ENREF_97)), Italy | Retrospective cohort, multi-centre | Age group: <216 (n=2) | 2 Not possible to extract | 2 Whites (Caucasians) | 2 Defects in innate immunity (types were not reported) | 2 NSGDwR | 2 NOPMiIrPwD | 2 Unknowns | 2 Mild  2 No MIS-C  AND  2 Not possible to extract | 2 Not reported | 2 Not reported | 0 | 0 | 0 | (NOS, 7)  2 survived |
| Moazzen et al. 2021 ([98](#_ENREF_98)), Iran | Retrospective cohort, single centre | 84 | 0 (0) | 1 Persian | 1 Mendelian susceptibility to mycobacterial disease (type was not reported) | 1 NSGDwR | 1 NOPMiIrPwD | 1 Unknown | 1 Mild  1 No MIS-C  AND  1 Seizures | 1 Not reported | 1 Antibiotics  1 Famotidine | 0 | 0 | 0 | (NOS, 6)  1 survived |
| **IUIS IEIs category: Autoinflammatory diseases (Group VII)** | | | | | | | | | | | | | | | |
| Kaya Akca et al. 2022 ([75](#_ENREF_75)), Turkey | Prospective case-control, single centre | Mean ± SD, 12.5 ± 3.7 | 12 (48) | 25 Whites (Caucasians) | 25 Defects affecting the inflammasome (FMFs) | MEFV (n=25) | M694V (n=3), M680I (n=2), and V726A (n=1)  19 NOPMiIrPwD | 25 ARs | 2 Asymptomatic  23 Mild  25 No MIS-C  AND  2 Epilepsy  2 Asthma  1 Psoriasis  1 Vesicoureteral reflux  1 Hepatic steatosis  1 Acute rheumatic fever  1 Obsessive compulsive disorder | 18 Leukocytosis  13 High ESR  9 High CRP | 11 Favipiravir  1 Hydroxychloroquine  1 Antibiotics | 0 | 0 | 0 | (NOS, 7)  25 survived |
| Gelzo et al. 2022 ([62](#_ENREF_62)), Italy | Retrospective cohort, single centre | 108 | 0 (0) | 1 White (Caucasian) | 1 Defect affecting the inflammasome (FMF) | MEFV (n=1) | c.2084A>G, p (n=1) | 1 AR | 1 Not reported  1 Yes MIS-C  AND  1 No comorbidities | 1 Not reported | 1 Not reported | 1 Not reported | 1 Not reported | 1 Not reported | (NOS, 7)  1 outcome was not reported |
| [Goudouris](https://scholar.google.com/citations?user=nyuqBh4AAAAJ&hl=en&oi=sra) et al. 2021 ([65](#_ENREF_65)), Brazil | Retrospective cohort, multi-centre | 214 | 0 (0) | 1 Hispanic | 1 Defect affecting the inflammasome (FMF) | MEFV (n=1) | 1 NOPMiIrPwD | 1 AR | 1 Mild  1 No MIS-C  AND  1 No comorbidities | 1 Not reported | 1 No treatment | 0 | 0 | 0 | (NOS, 8)  1 survived |
| Haslak et al. 2020 ([7](#_ENREF_7)), Turkey | Retrospective cohort, multi-centre | 204, 208 and 120 | 2 (66.7) | 3 Whites (Caucasians) | 3 Defects affecting the inflammasome (FMFs) | MEFV (n=3) | 3 NOPMiIrPwD | 3 ARs | 3 Asymptomatic  3 No MIS-C  AND  3 No comorbidities | 3 Not reported | 2 Antibiotics 2 Oseltamivir 2 Hydroxychloroquine | 0 | 0 | 0 | (NOS, 7)  3 survived |
| Haslak et al. 2022 ([70](#_ENREF_70)), Turkey | Retrospective cohort, multi-centre | 150 and 168 | 1 (50) | 2 Whites (Caucasians) | 2 Defects affecting the inflammasome (FMFs) | MEFV (n=2) | 2 NOPMiIrPwD | 2 ARs | 2 Asymptomatic  2 No MIS-C  AND  2 No comorbidities | 2 Not reported | 2 Colchicine | 0 | 0 | 0 | (NOS, 7)  2 survived |
| Sener et al. 2022 ([115](#_ENREF_115)), Turkey | Retrospective case-series, single centre | 36, 180 and 60 | 2 (66.7) | 3 Whites (Caucasians) | 3 Defects affecting the inflammasome (FMFs) | MEFV (n=3) | 3 NOPMiIrPwD | 3 ARs | 2 Mild  1 Moderate  3 Yes MIS-C  AND  3 No comorbidities | 3 Low Hb  1 Leukocytosis  1 Thrombocytopenia  3 High CRP  3 High ESR  3 High interleukin-6  3 High ferritin  3 High D-dimer  1 High Troponine I  2 High NT-proBNP | 3 IVIG  3 Steroids  2 Anakinra | 0 | 0 | 0 | (Modified NOS, high)  3 survived |
| Welzel et al. 2021 ([128](#_ENREF_128)), Germany | Retrospective case-series, single centre | 180 | 0 (0) | 1 Whites (Caucasians) | 1 Defect affecting the inflammasome (FMF) | MEFV (n=1) | p.M694V (n=1) | 1 AR | 1 Mild  1 No MIS-C  AND  1 No comorbidities | 1 Normal laboratory parameters | 1 Anakinra  1 Colchicine  1 Canakinumab | 0 | 0 | 0 | (Modified NOS, high)  1 survived |
| Gelzo et al. 2022 ([62](#_ENREF_62)), Italy | Retrospective cohort, single centre | 96 and 72 | 2 (100) | 2 Whites (Caucasians) | 2 Type 1 interferonopathies (Blau syndromes) | NOD2 (n=2) | c.2753C>A (n=1)  c.415C>T (n=1) | 2 ADs | 2 Not reported  2 Yes MIS-C  AND  2 No comorbidities | 2 Not reported | 2 Not reported | 2 Not reported | 2 Not reported | 2 Not reported | (NOS, 7)  2 outcome was not reported |
| Vagrecha et a. 2022 ([125](#_ENREF_125)), United States | Retrospective cohort, single centre | 60 and 108 | 2 (100) | 1 Black  1 Asian | 2 Type 1 interferonopathies (Blau syndromes) | NOD2 (n=2) | c.380C>T (n=1)  c.1390G>T (n=1) | 2 ADs | 2 Not reported  2 Yes MIS-C  AND  2 No comorbidities | 2 Not possible to extract | 2 Not possible to extract | 2 Not possible to extract | 2 Not possible to extract | 2 Not possible to extract | (NOS, 7)  2 outcome was not reported |
| Milito et al. 2021 ([97](#_ENREF_97)), Italy | Retrospective cohort, multi-centre | Age group: <216 (n=1) | 1 Not possible to extract | 1 Whites (Caucasians) | 1 Type 1 interferonopathies (Aicardi-Goutières syndrome) | 1 NSGDwR | 1 NOPMiIrPwD | 1 Unknown | 1 Mild  1 No MIS-C  AND  1 Not possible to extract | 1 Not reported | 1 Not reported | 0 | 0 | 0 | (NOS, 7)  1 survived |
| Pararajasingam et al. 2022 ([103](#_ENREF_103)), United Kingdom | Retrospective case report, single centre | 60 | 0 (0) | 1 Pakistani | 1 Type 1 interferonopathies (Aicardi-Goutières syndrome) | SAMHD1 (n=1) | c.427C>T (n=1) | 1 AR | 1 Asymptomatic  1 No MIS-C  AND  1 Severe neurological deficit  1 Spastic quadriplegia  1 Dystonia | 1 Raised liver enzymes  1 Raised amylase  1 High LDH  1 High D-dimer | 1 Steroids  1 Ruxolitinib | 0 | 0 | 0 | (Modified NOS, high)  1 survived |
| Shields et al. 2022 ([118](#_ENREF_118)), United Kingdom | Retrospective cohort, multi-centre | Age group: (0 to 216) (n=1) | 0 (0) | 1 White (Caucasian) | 1 Type 1 interferonopathies (Aicardi-Goutières syndrome) | 1 NSGDwR | 1 NOPMiIrPwD | 1 Unknown | 1 Not reported  1 Not reported  AND  1 No comorbidities | 1 Not possible to extract | 1 Not possible to extract | 0 | 0 | 0 | (NOS, 8)  1 survived |
| [Goudouris](https://scholar.google.com/citations?user=nyuqBh4AAAAJ&hl=en&oi=sra) et al. 2021 ([65](#_ENREF_65)), Brazil | Retrospective cohort, multi-centre | 22 | 1 (100) | 1 Hispanic | 1 Defect affecting the inflammasome (Familial cold autoinflammatory syndromes 1) | NLRP3 (n=1) | 1 NOPMiIrPwD | 1 AD | 1 Asymptomatic  1 No MIS-C  AND  1 No comorbidities | 1 Not reported | 1 No treatment | 0 | 0 | 0 | (NOS, 8)  1 survived |
| Welzel et al. 2021 ([128](#_ENREF_128)), Germany | Retrospective case-series, single centre | 168 and 144 | 1 (50) | 2 Whites (Caucasians) | 2 Defects affecting the inflammasome (Familial cold autoinflammatory syndromes 1) | NLRP3 (n=2) | p.Q703K (n=2) | 2 ADs | 2 Mild  2 No MIS-C  AND  2 Cryopyrin-associated periodic syndrome  2 Arthralgia/arthritis | 2 Elevated CRP  2 Elevated serum amyloid A | 2 Anakinra  2 Canakinumab | 0 | 0 | 0 | (Modified NOS, high)  2 survived |
| Karakoc Aydiner et al. 2022 ([73](#_ENREF_73)), Turkey | Prospective cohort, multi-centre | 144 | 0 (0) | 1 White (Caucasian) | 1 Type 1 interferonopathies (ADA2 deficiency) | ADA2 deficiency (n=1) | 1 NOPMiIrPwD | 1 AR | 1 Mild  1 Yes MIS-C  AND  1 Intracranial thrombosis | 1 High CRP  1 Neutropenia  1 High ferritin  1 High fibrinogen  1 High LDH  1 High D-dimer | 1 Favipiravir  1 IVIG  1 Antibiotics | 0 | 0 | 0 | (NOS, 7)  1 survived |
| Vagrecha et a. 2022 ([125](#_ENREF_125)), United States | Retrospective cohort, single centre | 84 | 1 (100) | 1 Hispanic | 1 Type 1 interferonopathies (ADA2 deficiency) | ADA2 deficiency (n=1) | c.1385T>C (n=1)  c.1474A>G (n=1) | 1 AR | 1 Not reported  1 Yes MIS-C  AND  1 No comorbidities | 1 Not possible to extract | 1 Not possible to extract | 1 Not possible to extract | 1 Not possible to extract | 1 Not possible to extract | (NOS, 7)  1 outcome was not reported |
| Abolhassani et al. 2022 ([19](#_ENREF_19)), Iran | Prospective cohort, single centre | 216 | 1 (100) | 1 Persian | 1 Defect affecting the inflammasome (NLRP1 deficiency) | NLRP1 (n=1) | Lymphocyte development/epigenetic: KMT2D (n=1)  NF-kB pathway genes: CARD14 (n=1) | 1 AR | 1 Severe  1 No MIS-C  AND  1 Not reported | 1 Not reported | 1 Antibiotics 1 Antiplatelets  1 TPN | 1 | 0 | 0 | (NOS, 8)  1 survived |
| Gelzo et al. 2022 ([62](#_ENREF_62)), Italy | Retrospective cohort, single centre | 48 | 0 (0) | 1 White (Caucasian) | 1 Defect affecting the inflammasome (NLRP1 deficiency) | NLRP1 (n=1) | c.2528+1G>C (n=1) | 1 AR | 1 Not reported  1 Yes MIS-C  AND  1 No comorbidities | 1 Not reported | 1 Not reported | 1 Not reported | 1 Not reported | 1 Not reported | (NOS, 7)  1 outcome was not reported |
| Kołtan et al. 2022 ([81](#_ENREF_81)), Poland | Retrospective cohort, multi-centre | 1 Not possible to extract | 1 Not possible to extract | 1 White (Caucasian) | 1 Non-inflammasome related conditions (TNF receptor-associated periodic syndrome) | TNFRSF1A (n=1) | 1 NOPMiIrPwD | 1 AD | 1 Moderate  1 No MIS-C  AND  1 Not possible to extract | 1 Not reported | 1 Not possible to extract | 0 | 0 | 0 | (NOS, 7)  1 survived |
| Vagrecha et a. 2022 ([125](#_ENREF_125)), United States | Retrospective cohort, single centre | 96 | 1 (100) | 1 Hispanic | 1 Non-inflammasome related conditions (TNF receptor-associated periodic syndrome) | TNFRSF1A (n=1) | c.596T>C (n=1) | 1 AD | 1 Not reported  1 Yes MIS-C  AND  1 No comorbidities | 1 Not possible to extract | 1 Not possible to extract | 1 Not possible to extract | 1 Not possible to extract | 1 Not possible to extract | (NOS, 7)  1 outcome was not reported |
| Singh et al. 2022 ([119](#_ENREF_119)), India | Retrospective case report, single centre | 60 | 0 (0) | 1 Indian | 1 Non-inflammasome related conditions (Hyperpigmentation hypertrichosis, histiocytosis-lymphadenopathy plus syndrome SLC29A3 mutation) | SLC29A3 (n=1) | 1 NOPMiIrPwD | 1 AR | 1 Mild  1 No MIS-C  AND  1 HLH  1 Cardiomegaly  1 Pallor  1 Hypertrichosis (forehead and face)  1 Poor oral hygiene | 1 Low Hb  1 Leukopenia  1 Neutropenia  1 Thrombocytopenia  1 Anaemia  1 Raised liver enzymes  1 High D-dimer  1 High ferritin  1 High interleukin-6  1 High CRP  1 High fibrinogen  1 High triglycerides | 1 Steroids  1 IVIG  1 Antibiotics | 0 | 0 | 0 | (Modified NOS, high)  1 survived |
| Vagrecha et a. 2022 ([125](#_ENREF_125)), United States | Retrospective cohort, single centre | 84 | 1 (100) | 1 Black | 1 Non-inflammasome related conditions (Hyperpigmentation hypertrichosis, histiocytosis-lymphadenopathy plus syndrome SLC29A3 mutation) | SLC29A3 (n=1) | c.1202G>A (n=1) | 1 AR | 1 Not reported  1 Yes MIS-C  AND  1 No comorbidities | 1 Not possible to extract | 1 Not possible to extract | 1 Not possible to extract | 1 Not possible to extract | 1 Not possible to extract | (NOS, 7)  1 outcome was not reported |
| Meyts et al. 2021 ([95](#_ENREF_95)), 10 countries | Retrospective cohort, multi-centre | Age groups:36-144 (n=2) | 2 (100) | 2 Whites (Caucasians) | 2 Type 1 interferonopathies (RNASEH2B deficiency) | RNASEH2B (n=2) | 2 NOPMiIrPwD | 2 ARs | 2 Asymptomatic  2 No MIS-C  AND  2 Cognitive disabilities | 2 Not reported | 2 No treatment | 0 | 0 | 0 | (NOS, 8)  2 survived |
| Kołtan et al. 2022 ([81](#_ENREF_81)), Poland | Retrospective cohort, multi-centre | 1 Not possible to extract | 1 Not possible to extract | 1 White (Caucasian) | 1 Defect affecting the inflammasome (Familial cold autoinflammatory syndrome 4) | NLRC4 (n=1) | 1 NOPMiIrPwD | 1 AD | 1 Mild  1 No MIS-C  AND  1 Not possible to extract | 1 Not reported | 1 Not possible to extract | 0 | 0 | 0 | (NOS, 7)  1 survived |
| Delavari et al. 2021 ([48](#_ENREF_48)), Iran | Prospective cohort, multi-centre | 96 | 0 (0) | 1 Persian | 1 Non-inflammasome related conditions (Deficiency of the interleukin 1 receptor antagonist) | IL1RN (n=1) | 1 NOPMiIrPwD | 1 AR | 1 Severe  1 Yes MIS-C  AND  1 Anaemia  1 Severe generalized erythroderma  1 Ulcerative colitis  1 Oedema in the right shoulder  1 Ascites  1 Femur swelling | 1 Anaemia  1 Low Hb  1 High CRP  1 High ESR | 1 Antibiotics  1 Hydroxychloroquine | 1 | 1 | 1 | (NOS, 7)  1 died (COVID-19-related) |
| Gelzo et al. 2022 ([62](#_ENREF_62)), Italy | Retrospective cohort, single centre | 120 | 0 (0) | 1 White (Caucasian) | 1 Non-inflammasome related conditions [Pyogenic sterile arthritis, pyoderma gangrenosum, acne (PAPA) syndrome, hyperzincemia and hypercalprotectinemia] | PSTPIP1 (n=1) | c.421_424delGACG, p (n=1) | 1 AD | 1 Not reported  1 Yes MIS-C  AND  1 No comorbidities | 1 Not reported | 1 Not reported | 1 Not reported | 1 Not reported | 1 Not reported | (NOS, 7)  1 outcome was not reported |
| Kołtan et al. 2022 ([81](#_ENREF_81)), Poland | Retrospective cohort, multi-centre | 1 Not possible to extract | 1 Not possible to extract | 1 White (Caucasian) | 1 Defect affecting the inflammasome (Mevalonate kinase deficiency) | MVK (n=1) | 1 NOPMiIrPwD | 1 AR | 1 Mild  1 No MIS-C  AND  1 Not possible to extract | 1 Not reported | 1 Not possible to extract | 0 | 0 | 0 | (NOS, 7)  1 survived |
| Meyts et al. 2021 ([95](#_ENREF_95)), 10 countries | Retrospective cohort, multi-centre | Age groups:36-144 (n=1) | 0 (0) | 1 Hispanic | 1 Type 1 interferonopathies (SAMHD1 deficiency) | SAMHD1 deficiency (n=1) | 1 NOPMiIrPwD | 1 AR | 1 Asymptomatic  1 No MIS-C  AND  1 Cognitive disability  1 Spastic quadriplegy  1 Epilepsy | 1 Not reported | 1 Steroids  1 Chloroquine | 0 | 0 | 0 | (NOS, 8)  1 survived |
| Shields et al. 2022 ([118](#_ENREF_118)), United Kingdom | Retrospective cohort, multi-centre | Age group: (0 to 216) (n=1) | 0 (0) | 1 White (Caucasian) | 1 Non-inflammasome related conditions (A20 deficiency) | TNFAIP3 (n=1) | 1 NOPMiIrPwD | 1 AD | 1 Not reported  1 Not reported  AND  1 No comorbidities | 1 Not possible to extract | 1 Not possible to extract | 0 | 0 | 0 | (NOS, 8)  1 survived |
| Vagrecha et a. 2022 ([125](#_ENREF_125)), United States | Retrospective cohort, single centre | 84 | 1 (100) | 1 Black | 1 Non-inflammasome related conditions (Majeed syndrome) | LPIN2 (n=1) | c.1133C>T (n=1) | 1 AR | 1 Not reported  1 Yes MIS-C  AND  1 No comorbidities | 1 Not possible to extract | 1 Not possible to extract | 1 Not possible to extract | 1 Not possible to extract | 1 Not possible to extract | (NOS, 7)  1 outcome was not reported |
| Vagrecha et a. 2022 ([125](#_ENREF_125)), United States | Retrospective cohort, single centre | 84 | 1 (100) | 1 Black | 1 Type 1 interferonopathies (STING-like disease) | TMEM173 (n=1) | c.575G>T (n=1) | 1 AR | 1 Not reported  1 Yes MIS-C  AND  1 No comorbidities | 1 Not possible to extract | 1 Not possible to extract | 1 Not possible to extract | 1 Not possible to extract | 1 Not possible to extract | (NOS, 7)  1 outcome was not reported |
| Vagrecha et a. 2022 ([125](#_ENREF_125)), United States | Retrospective cohort, single centre | 96 | 0 (0) | 1 Black | 1 Non-inflammasome related conditions (CARD14 mediated psoriasis) | CARD14 (n=1) | c.2956C>T (n=1) | 1 AD | 1 Not reported  1 Yes MIS-C  AND  1 No comorbidities | 1 Not possible to extract | 1 Not possible to extract | 1 Not possible to extract | 1 Not possible to extract | 1 Not possible to extract | (NOS, 7)  1 outcome was not reported |
| Abolhassani et al. 2022 ([19](#_ENREF_19)), Iran | Prospective cohort, single centre | 180 | 0 (0) | 1 Persian | 1 Autoinflammatory disease (type was not reported) | 1 NSGDwR | Lymphocyte development/epigenetic: PSEN1 (n=1)  DNA repair gene defect: ATM (n=1)  IL-1 activation pathway genes: NLRP1 (n=1) | 1 Unknown | 1 Severe  1 No MIS-C  AND  1 Not reported | 1 Not reported | 1 Antibiotics  1 Steroids  1 Oxygen supplementation | 1 | 0 | 0 | (NOS, 8)  1 survived |
| Castano-Jaramillo et al. 2021 ([40](#_ENREF_40)), Mexico | Retrospective cohort, multi-centre | 144 | 1 (100) | 1 Hispanic | 1 Autoinflammatory disease (type was not reported) | 1 NSGDwR | 1 NOPMiIrPwD | 1 Unknown | 1 Mild  1 No MIS-C  AND  1 Vasculitis | 1 Not reported | 1 IVIG | 0 | 1 | 1 | (NOS, 8)  1 died (not reported if COVID-19-related) |
| **IUIS IEIs category: Complement deficiencies (Group VIII)** | | | | | | | | | | | | | | | |
| Abolhassani et al. 2022 ([19](#_ENREF_19)), Iran | Prospective cohort, single centre | 180 and 132 | 2 (100) | 2 Persians | 2 Complement deficiencies (Factor H deficiencies) | CFH (n=2) | Lymphocyte development/epigenetic: CLEC16A (n=1)  IFN pathway genes: IRF5, IFNA8, TLR4 and IRF2BPL (n=1) | 2 ARs | 1 Severe  1 Critical  2 Yes MIS-C  AND  2 Type I IFN deficiency  2 Vasculitis  2 Thrombocytopenia  2 Proteinuria  2 Reduced complement component C3 | 1 Low serum IgA, IgM and IgG levels | 2 Antibiotics  2 IVIG  2 ACEIs  2 Antiplatelets  1 Biological agents  2 TPN  1 CPR | 2 | 1 | 1 | (NOS, 8)  1 survived  1 died (COVID-19-related) |
| Gelzo et al. 2022 ([62](#_ENREF_62)), Italy | Retrospective cohort, single centre | 24 | 0 (0) | 1 White (Caucasian) | 1 Complement deficiencies (Factor H deficiency) | CFH (n=1) | c.1696G>A p (n=1) | 1 AR | 1 Not reported  1 Yes MIS-C  AND  1 No comorbidities | 1 Not reported | 1 Not reported | 1 Not reported | 1 Not reported | 1 Not reported | (NOS, 7)  1 outcome was not reported |
| [Goudouris](https://scholar.google.com/citations?user=nyuqBh4AAAAJ&hl=en&oi=sra) et al. 2021 ([65](#_ENREF_65)), Brazil | Retrospective cohort, multi-centre | 23, 180 and 182 | 1 (33.3) | 3 Hispanics | 3 Complement deficiencies (C1 inhibitor deficiencies) | SERPING1 (n=3) | 3 NOPMiIrPwD | 3 ADs | 1 Asymptomatic  2 Mild  3 No MIS-C  AND  3 Hereditary angioedema  1 Obesity | 3 Not reported | 3 No treatment | 0 | 0 | 0 | (NOS, 8)  3 survived |
| Abolhassani et al. 2022 ([19](#_ENREF_19)), Iran | Prospective cohort, single centre | 120 | 1 (100) | 1 Persian | 1 Complement deficiencies (Factor H –related protein deficiencies) | CFHR1 (n=1) | IFN pathway genes: VAV1 and IKZF2 (n=1)  IL-1 activation pathway genes: NCSTN (n=1) | 1 AD | 1 Severe  1 No MIS-C  AND  1 Not reported | 1 Not reported | 1 Antibiotics  1 Biological agents  1 TPN | 1 | 0 | 0 | (NOS, 8)  1 survived |
| Gelzo et al. 2022 ([62](#_ENREF_62)), Italy | Retrospective cohort, single centre | 72 and 168 | 1 (50) | 2 Whites (Caucasians) | 2 Complement deficiencies (Ficolin 3 deficiency) | FCN3 (n=2) | c.349del, p (n=2) | 2 ARs | 2 Not reported  2 Yes MIS-C  AND  2 No comorbidities | 2 Not reported | 2 Not reported | 2 Not reported | 2 Not reported | 2 Not reported | (NOS, 7)  2 outcome was not reported |
| Gelzo et al. 2022 ([62](#_ENREF_62)), Italy | Retrospective cohort, single centre | 168 | 0 (0) | 1 White (Caucasian) | 1 Complement deficiencies (Factor I deficiency) | CFI (n=1) | c.605_607delGAA, p (n=1) | 1 AR | 1 Not reported    1 Yes MIS-C  AND  1 No comorbidities | 1 Not reported | 1 Not reported | 1 Not reported | 1 Not reported | 1 Not reported | (NOS, 7)  1 outcome was not reported |
| [Goudouris](https://scholar.google.com/citations?user=nyuqBh4AAAAJ&hl=en&oi=sra) et al. 2021 ([65](#_ENREF_65)), Brazil | Retrospective cohort, multi-centre | 201 | 0 (0) | 1 Hispanic | 1 Complement deficiencies (C3 deficiency) | C3 (n=1) | 1 NOPMiIrPwD | 1 AR | 1 Mild  1 No MIS-C  AND  1 No comorbidities | 1 Not reported | 1 No treatment | 0 | 0 | 0 | (NOS, 8)  1 survived |
| **IUIS IEIs category: Bone marrow failure (Group IX)** | | | | | | | | | | | | | | | |
| Chandar et al. 2022 ([41](#_ENREF_41)), India | Prospective cohort, single centre | 84 | 1 Gender was not reported | 1 Indian | 1 Bone marrow failure [Fanconi anaemia (type was not reported)] | 1 NSGDwR | 1 NOPMiIrPwD | 1 AR | 1 Mild  1 No MIS-C  AND  1 Post HSCT  1 Posterior reversible encephalopathy syndrome | 1 Not reported | 1 Tacrolimus | 0 | 0 | 0 | (NOS, 7)  1 survived |
| Esslami et al. 2021 ([54](#_ENREF_54)), Iran | Retrospective case report, single centre | 84 | 1 (100) | 1 Persian | 1 Bone marrow failure [Fanconi anaemia (type was not reported)] | 1 NSGDwR | 1 NOPMiIrPwD | 1 AR | 1 Moderate  1 No MIS-C  AND  1 Meningoencephalitis  1 Post HSCT  1 Graft versus host disease  1 Seizures  1 Jaw locking  1 Restlessness  1 Delirium  1 Posterior reversible encephalopathy syndrome | 1 Raised urea  1 Elevated creatinine | 1 Mycophenolate mofetil  1 Cyclosporin 1 Folic acid  1 Livergol  1 Tacrolimus  1 Levetiracetam  1 Vitamin B6  1 IVIG  1 Plasmapheresis  1 Diuretics  1 IV inotropes  1 Peritoneal dialysis | 1 | 1 | 1 | (Modified NOS, moderate)  1 died (not COVID-19-related) |
| Field et al. 2021 ([59](#_ENREF_59)), United States | Retrospective case report, single centre | 48 | 0 (0) | 1 White (Caucasian) | 1 Bone marrow failure [Fanconi anaemia (Type D1)] | BRCA2 (n=1) | 1 NOPMiIrPwD | 1 AR | 1 Asymptomatic  1 No MIS-C  AND  1 Medulloblastoma  1 Hydrocephalus  1 Brain mass  1 Surgical resection | 1 Not reported | 1 Chemotherapy | 0 | 0 | 0 | (Modified NOS, high)  1 survived |
| Sengupta et al. 2021 ([116](#_ENREF_116)), India | Retrospective case report, single centre | 60 | 0 (0) | 1 Indian | 1 Bone marrow failure [Fanconi anaemia (type was not reported)] | 1 NSGDwR | 1 NOPMiIrPwD | 1 AR | 1 Not reported  1 Not reported  AND  1 Metabolic acidosis  1 Polyuria  1 Shock  1 Renal tubular acidosis  1 Decreased urine output | 1 Low bicarbonate  1 Low potassium  1 Low phosphate  1 Proteinuria  1 Glucosuria  1 Elevated beta-2 microglobulin (urine)  1 Urinary phosphate loss | 1 IV fluids  1 Correction of bicarbonate, potassium, and phosphorus | 0 | 0 | 0 | (Modified NOS, moderate)  1 survived |
| Karimi et al. 2021 ([74](#_ENREF_74)), Iran | Retrospective cohort, multi- centre | 48 | 0 (0) | 1 Persian | 1 Bone marrow failure [Fanconi anaemia (type was not reported)] | 1 NSGDwR | 1 NOPMiIrPwD | 1 AR | 1 Mild  1 Not reported  AND  1 Adenitis due to tuberculosis vaccine | 1 Not possible to extract | 1 IVIG  1 Favipiravir | 0 | 0 | 0 | (NOS, 6)  1 survived |
| Ouederni et al. 2021 ([101](#_ENREF_101)), Tunisia | Retrospective case report, single centre | 60 | 0 (0) | 1 Arab | 1 Bone marrow failure (SAMD9 deficiency) | SAMD 9 deficiency (n=1) | 1 NOPMiIrPwD | 1 AD | 1 Moderate  1 No MIS-C  AND  1 Myelodysplasia 1 Monosomy 7 | 1 Not reported | 1 No treatment | 0 | 0 | 0 | (Modified NOS, high)  1 survived |
| Abolhassani et al. 2022 ([19](#_ENREF_19)), Iran | Prospective cohort, single centre | 204 | 0 (0) | 1 Persian | 1 Bone marrow failure (DKCA1 deficiency) | TERT (n=1) | IFN pathway genes: IRF2BPL (n=1)  IL-1 activation pathway genes: NLRP2 (n=1) | 1 AD | 1 Severe  1 No MIS-C  AND  1 Not reported | 1 Not reported | 1 Antibiotics  1 Antiplatelets  1 TPN | 1 | 0 | 0 | (NOS, 8)  1 survived |
| **IUIS IEIs category: Phenocopies of primary immunodeficiencies (Group X)** | | | | | | | | | | | | | | | |
| Topal et al. 2022 ([124](#_ENREF_124)), Turkey | Retrospective cohort, single centre | 120 | 1 Gender was not reported | 1 White (Caucasian) | 1 Nonmalignant clinical syndrome (RAS-associated autoimmune leukoproliferative disease) | SPRED1 (n=1) | c.684+50A>T (n=1) | 1 AR | 1 Mild  1 No MIS-C  AND  1 Hypertelorism  1 Secondary HLH  1 Aplastic anaemia 1 Flattened nose  1 Ptosis  1 Downslanting palpebral fissures and epicanthal folds  1 Low hairline  1 Long philtrum | 1 Not reported | 1 No treatment | 0 | 0 | 0 | (NOS, 7)  1 survived |

Abbreviations: ACEIs, angiotensin-converting enzyme inhibitors; AD, autosomal dominant; AIHA, autoimmune haemolytic anaemia; AIRE, autoimmune regulator gene; ARDs, acute respiratory distress syndrome; AR, autosomal recessive; APS-1, autoimmune polyendocrine syndromes type-1; CGD, chronic granulomatous disease; CHD, congenital heart disease; CID, combined immunodeficiency; CLD, chronic lung disease; CMC, cutaneous mucocutaneous candidiasis; CNS, central nervous system; COVID-19, coronavirus disease 2019; CPR, cardiopulmonary resuscitation; CRP, C-reactive protein; CSF, cerebrospinal fluid; CVID, common variable immunodeficiency; DIC, disseminated intravascular coagulation; ECMO, extracorporeal membrane oxygenation; EDA-ID, anhidrotic ectodermodysplasia with immunodeficiency; ESR, erythrocyte sedimentation rate; FHL syndromes, familial hemophagocytic lymphohistiocytosis; FMF, familial Mediterranean fever; G6PD, glucose-6-phosphate dehydrogenase; GERD, gastroesophageal reflux disease; GIT, gastrointestinal tract; Hb, haemoglobin; HIgM, hyper immunoglobulin M syndrome; HLH, hemophagocytic lymphohistiocytosis; HSCT, hematopoietic stem cell transplant; IBD, inflammatory bowel disease; ICF, immunodeficiency with centromeric instability and facial anomalies; ICU, intensive care unit; IEIs, inborn errors of immunity; IFN, interferons; IFNAR, interferon alpha/ beta receptor subunit; IgA, immunoglobulin A; IgG, immunoglobulin G; IgM, immunoglobulin M; IL, interleukin; IPEX, immune-dysregulation polyendocrinopathy X-linked; ITP, immune thrombocytopenic purpura; IUIS, International Union of Immunologic Societies; IV, intravenous; IVIG, intravenous immunoglobulin; LDH, lactate dehydrogenase; LRTIs, lower respiratory tract infections; MIS-C, multisystem inflammatory syndrome in children; MMRV, measles, mumps, rubella and varicella vaccine; MRSA, methicillin-resistant *Staphylococcus aureus*; NF-kB, nuclear factor kappa B; NK, natural killer; NOPMiIrPwD, no other potential modifiers in immunity-related pathways were determined; NOS, Newcastle Ottawa Scale; NSGDwR, no specific gene defect was reported; NT-proBNP, N-terminal pro b-type natriuretic peptide; NF-κB, nuclear factor kappa-light-chain-enhancer of activated B cells; PT, prothrombin time; PTT, partial thromboplastin time; RBCs, red blood cells; SAD, specific antibody deficiency; SARS-CoV-2, severe acute respiratory syndrome coronavirus 2; SCID, severe combined immunodeficiencies; SCIg, subcutaneous immunoglobulin; SD, standard deviation; TLR, toll-like receptors; TNF-a, tumor necrosis factor-alpha; TPN total parenteral nutrition; TPP2, tripeptidyl-Peptidase II; URTIs, upper respiratory tract infections; UTIs, urinary trat infections; WBCs, white blood cells; XLP, X-linked lymphoproliferative disease.

^a^Age is presented in month for each individual patient in all included studies except for one study ([75](#_ENREF_75)) [age was presented as mean ± (SD)].

^b^Patients with black ethnicity include African-American, Black African, African and Afro-Caribbean patients.
